# Microenvironmental Heterogeneity Parallels Breast Cancer Progression: A Histology–Genomic Integration Analysis

**DOI:** 10.1371/journal.pmed.1001961

**Published:** 2016-02-16

**Authors:** Rachael Natrajan, Heba Sailem, Faraz K. Mardakheh, Mar Arias Garcia, Christopher J. Tape, Mitch Dowsett, Chris Bakal, Yinyin Yuan

**Affiliations:** 1 Breast Cancer Now Toby Robins Research Centre, The Institute of Cancer Research, London, United Kingdom; 2 Division of Molecular Pathology, The Institute of Cancer Research, London, United Kingdom; 3 Division of Cancer Biology, The Institute of Cancer Research, London, United Kingdom; 4 Centre for Molecular Pathology, Royal Marsden Hospital, London, United Kingdom; 5 Academic Department of Biochemistry, Royal Marsden Hospital, London, United Kingdom; 6 Centre for Evolution and Cancer, The Institute of Cancer Research, London, United Kingdom; Harvard Medical School, UNITED STATES

## Abstract

**Background:**

The intra-tumor diversity of cancer cells is under intense investigation; however, little is known about the heterogeneity of the tumor microenvironment that is key to cancer progression and evolution. We aimed to assess the degree of microenvironmental heterogeneity in breast cancer and correlate this with genomic and clinical parameters.

**Methods and Findings:**

We developed a quantitative measure of microenvironmental heterogeneity along three spatial dimensions (3-D) in solid tumors, termed the tumor ecosystem diversity index (EDI), using fully automated histology image analysis coupled with statistical measures commonly used in ecology. This measure was compared with disease-specific survival, key mutations, genome-wide copy number, and expression profiling data in a retrospective study of 510 breast cancer patients as a test set and 516 breast cancer patients as an independent validation set. In high-grade (grade 3) breast cancers, we uncovered a striking link between high microenvironmental heterogeneity measured by EDI and a poor prognosis that cannot be explained by tumor size, genomics, or any other data types. However, this association was not observed in low-grade (grade 1 and 2) breast cancers. The prognostic value of EDI was superior to known prognostic factors and was enhanced with the addition of *TP53* mutation status (multivariate analysis test set, *p* = 9 × 10^−4^, hazard ratio = 1.47, 95% CI 1.17–1.84; validation set, *p* = 0.0011, hazard ratio = 1.78, 95% CI 1.26–2.52). Integration with genome-wide profiling data identified losses of specific genes on 4p14 and 5q13 that were enriched in grade 3 tumors with high microenvironmental diversity that also substratified patients into poor prognostic groups. Limitations of this study include the number of cell types included in the model, that EDI has prognostic value only in grade 3 tumors, and that our spatial heterogeneity measure was dependent on spatial scale and tumor size.

**Conclusions:**

To our knowledge, this is the first study to couple unbiased measures of microenvironmental heterogeneity with genomic alterations to predict breast cancer clinical outcome. We propose a clinically relevant role of microenvironmental heterogeneity for advanced breast tumors, and highlight that ecological statistics can be translated into medical advances for identifying a new type of biomarker and, furthermore, for understanding the synergistic interplay of microenvironmental heterogeneity with genomic alterations in cancer cells.

## Abstract 2

A novel approach that maps tumor microenvironment heterogeneity and couples this with genetic information to provide superior prognosis in breast cancer.

## Editors' Summary

**Background:**

The human body contains millions of cells, all of which grow, divide, and die in an orderly fashion to build tissues during early life and to replace worn-out or dying cells and repair injuries during adult life. Sometimes, however, normal cells acquire genetic changes (mutations) that allow them to divide uncontrollably and to move around the body (metastasize), resulting in cancer. Because any cell in the body can acquire the mutations needed for cancer development, there are many types of cancer. For example, breast cancer, the most common cancer in women, begins when the cells in the breast that normally make milk become altered. Moreover, different types of cancer progress and evolve differently—some cancers grow quickly and kill their “host” soon after diagnosis, whereas others can be successfully treated with drugs, surgery, or radiotherapy. The behavior of individual cancers depends both on the characteristics of the cancer cells within the tumor and on the interactions between the cancer cells and the normal stromal cells (the connective tissue cells of organs) and other cells (for example, immune cells) that surround and feed cancer cells (the tumor microenvironment).

**Why Was This Study Done?:**

Although recent studies have highlighted the importance of the tumor microenvironment for disease-related outcomes, little is known about how the heterogeneity of the tumor microenvironment—the diversity of non-cancer cells within the tumor—affects outcomes. Mathematical modeling suggests that tumors with heterogeneous and homogeneous microenvironments have different growth patterns and that heterogeneous microenvironments are more likely to be associated with aggressive cancers than homogenous microenvironments. However, the lack of methods to quantify the spatial variability and cellular composition across solid tumors has prevented confirmation of these predictions. Here, the researchers develop a computational system for quantifying microenvironmental heterogeneity in breast cancer based on tumor morphology (shape and form) in histological sections (tissue samples taken from tumors that are examined microscopically). They then use this system to analyze the associations between clinical outcomes, molecular changes, and microenvironmental heterogeneity in breast cancer.

**What Did the Researchers Do and Find?:**

The researchers used automated image analysis and statistical analysis to develop the ecosystem diversity index (EDI), a numerical measure of microenvironmental heterogeneity in solid tumors. They compared the EDI with prognosis (likely outcome), key mutations, genome-wide copy number (tumor cells often contain abnormal numbers of copies of specific genes), and expression profiling data (the expression of several key proteins is altered in tumors) in a test set of 510 samples from patients with breast cancer and in a validation set of 516 additional samples. Among high-grade breast cancers (grade 3 cancers; the grade of a cancer indicates what the cells look like; high-grade breast cancers have a poor prognosis), but not among low-grade breast cancers (grades 1 and 2), a high EDI (high microenvironmental heterogeneity) was associated with a poor prognosis. Specifically, patients with grade 3 tumors and a high EDI had a ten-year disease-specific survival rate of 51%, whereas the remaining patients with grade 3 tumors had a ten-year survival rate of 70%. Notably, the combination of a high EDI with specific DNA alterations—mutations in a gene called *TP53* and loss of genes on Chromosomes 4p14 and 5q13—improved the accuracy of prognosis among patients with grade 3 breast cancer and stratified them into subgroups with disease-specific five-year survival rates of 35%, 9%, and 32%, respectively.

**What Do These Findings Mean?:**

These findings establish a method for measuring the spatial heterogeneity of the microenvironment of solid tumors and suggest that the measurement of tumor microenvironmental heterogeneity can be coupled with information about genomic alterations to provide an accurate way to predict outcomes among patients with high-grade breast cancer. The association between EDI, specific genomic alterations, and outcomes needs to be confirmed in additional patients. However, these findings suggest that microenvironmental heterogeneity might provide an additional biomarker to help clinicians identify those patients with advanced breast cancer who have a particularly bad prognosis. The ability to identify these patients is important because it will help clinicians target aggressive treatments to individuals with a poor prognosis and avoid the overtreatment of patients whose prognosis is more favorable. Finally, and more generally, these findings describe a new way to investigate the interactions between the tumor microenvironment and genomic alterations in cancer cells.

**Additional Information:**

This list of resources contains links that can be accessed when viewing the PDF on a device or via the online version of the article at http://dx.doi.org/10.1371/journal.pmed.1001961.

- The US National Cancer Institute provides comprehensive information about cancer and its development (in English and Spanish), including detailed information about breast cancer and an online booklet for patients
- Cancer Research UK, a not-for-profit organization, provides information about cancer, including detailed information about breast cancer and a science blog on the tumor microenvironment
- Breast Cancer Now is a not-for-profit organization that provides up-to-date information about breast cancer (in English and Spanish)
- The UK National Health Service Choices website has information and personal stories about breast cancer; the not-for-profit organization Healthtalkonline also provides personal stories about dealing with breast cancer
- Wikipedia has a page about the tumor microenvironment (note that Wikipedia is a free online encyclopedia that anyone can edit; available in several languages)

## Introduction

Accumulating evidence suggests that the interactions of cancer cells and stromal cells within their microenvironment govern disease progression, metastasis, and, ultimately, the evolution of therapeutic resistance [1–3]. Recent reports have highlighted the significance of the contribution of stromal gene expression and morphological structure as powerful prognostic determinants for a number of tumor types, emphasizing the importance of the tumor microenvironment in disease-related outcomes [4–7]. In breast cancer, a number of studies have demonstrated the prognostic correlation of individual cell types, including the immune cell infiltrate that predicts response to therapy [8–10], and the high percentage of tumor stroma that predicts poor prognosis in triple-negative disease but good prognosis in estrogen receptor (ER)–positive disease [11,12]. Nevertheless, different types of cells coexist with varying degrees of heterogeneity within a tumor. This fundamental feature of human tumors and the combinatorial effects of cell types have been largely ignored, and the collective implications for clinical outcome remain elusive.

Consistent observations from mathematical models have highlighted that tumors with diverse microenvironments show growth patterns dramatically different from those of tumors with homogeneous environments [13] and are more likely to be associated with aggressive cancer phenotypes [2] that select for cell migration and eventual metastasis by allowing cancer cells to evolve more rapidly [14]. These observations highlight the need to understand the collective physiological characteristics and heterogeneity of tumor microenvironments. However, there is a lack of methods to quantify the high spatial variability and diverse cellular composition across different solid tumors. Moreover, the interplay of genomic alterations in cancer cells and microenvironmental heterogeneity and its subsequent role in treatment response have not been explored.

Our aims were (i) to develop a computational system for quantifying microenvironmental heterogeneity based on tumor morphology in routine histological sections, (ii) to define the clinical implications of microenvironmental heterogeneity, and (iii) to integrate this histology-based index with RNA gene expression and DNA copy number profiling data to identify molecular changes associated with microenvironmental heterogeneity.

## Methods

### Clinical Samples

**Fig 1:**
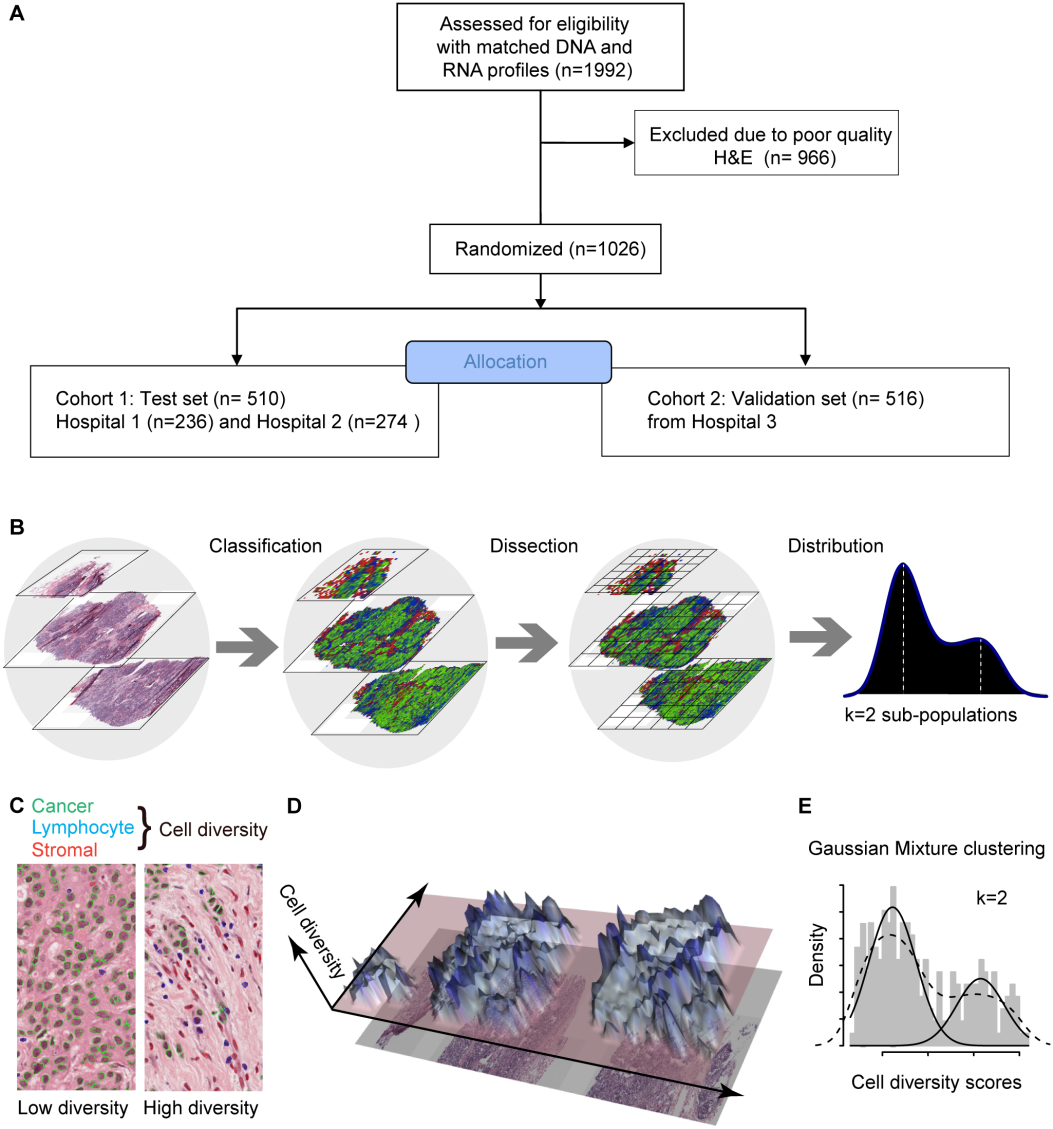
In silico tumor dissection pipeline for quantifying spatial diversity in the tumor ecosystem. (A) Flow diagram depicting the overall study design. (B) Schematic of our pipeline for quantifying spatial diversity in pathological samples. H&E sections are morphologically classified and divided into regions to be spatially scored. The number of clusters *k* in the regional scores is indicative of the number of sub-populations of cell types in the tumor regions. (C) Examples of tumor regions with low and high diversity scores using the Shannon diversity index, accounting for cancer cells (outlined in green), lymphocytes (blue), and stromal cells (red). Cell classification is automated by image analysis. (D) The 3-D landscape of cell diversity scores on an example H&E section; the *x-* and *y*-axes are the geometric axes of the image, and the *z*-axis is cell diversity computed on a region-by-region basis. (E) The distribution of regional scores in a tumor from the METABRIC study with two regional clusters identified using Gaussian mixture clustering (grey shading: histogram; dashed black line: density; solid black lines: mixture components/clusters).

Hematoxylin and eosin (H&E) sections of 1,992 untreated primary invasive breast carcinomas described in the METABRIC study [15] were quality assessed. These sections are from female patients diagnosed between 1980 and 2005 from consecutive series from five contributing hospitals in the UK and Canada with clinical annotations and matched DNA and RNA profiling data. Of these, H&E samples from two hospitals were highly fragmented, leaving in total 1,026 cases from the remaining three hospitals, which were split into a test set of 510 samples (hospital 1 and 2; Cohort 1) and an independent validation set of 516 samples (hospital 3; Cohort 2) for retrospective analysis (Fig 1A; S1 Table). On average, three sections (top, middle, and bottom) were taken from the single frozen tumor aliquot included in the METABRIC study in order to represent the morphological profile of the tumor [15,16]. Tumor sections were stained independently in different laboratories according to the hospital site. Whole-tumor section images, copy number profiled using Affymetrix SNP6, gene expression profiled using Illumina HT-12 array, and long-term follow-up data (median 68.3 mo) were obtained.

### Ethical Approval

All patients gave written consent for the use of material for research purposes. All patient specimens were obtained with appropriate ethical approval from the relevant institutional review boards (Addenbrooke’s Hospital, Cambridge, UK; Guy’s Hospital, London, UK; Nottingham, UK; Vancouver, Canada; Manitoba, Canada).

### The Ecosystem Diversity Index

To characterize the tumor ecosystem based on cell compositions, we developed a new index to be used in conjunction with our image analysis tool [16]. First, we used our automated morphological classification method [16] to identify and classify cells into cancer, lymphocyte, or stromal cell classes in H&E sections (Fig 1B). We next divided sections into smaller spatial regions and quantified the diversity of the tumor ecosystem in a tumor region *j* using the Shannon diversity index:

$$d_{j}=-\Sigma_{i}^{m}p_{i}\text{log}p_{i},$$ (1)

where *m* is the number of cell types and *p*_*i*_ is the proportion of the *i*th cell type (Fig 1B and 1C). A high value of the Shannon diversity index *d*_*j*_ reports a heterogeneous environment populated by many cell types, whilst a low value indicates a homogeneous environment (Fig 1C). Compared to other methods such as the Simpson index, the Shannon diversity index accounts for rare species and, hence, is less dominated by main species [17]. Subsequently, we derived the ecosystem diversity index (EDI) by applying unsupervised clustering that identifies the optimum number of clusters in the dataset in an unbiased manner, in order to group tumor regions and quantify the degree of spatial heterogeneity. Let *D* = *d*_1_,*d*_2_,…,*d*_*n*_ be the Shannon index for *n* regions in a tumor. We used Gaussian mixture models to fit data *D*:

$$D\;∼\Sigma_{k=1}^{K}\omega_{k}N(\mu_{k},\sigma_{k}^{2}).$$ (2)

where μ_*k*,_,$\sigma_{k}^{2}$, and ω_*k*_ are the mean, variance, and weight of a Gaussian distribution *k*, and *K* is the number of clusters. The Bayesian information criterion was then used to select the best number of clusters *K* [18]. We used *K* = 1–5 as the range of *K* to avoid small EDI groups (S1 Text). The final value of *K* thus is a measurement of heterogeneity and the score of EDI for a tumor.

### Statistical Methods and Data Analysis

Survival analysis was performed with breast-cancer-specific 10-y survival data. The Kaplan–Meier estimator was used, and the log-rank test was performed to test differences among groups. For univariate and multivariate analysis, the Cox proportional hazards regression model was fitted, and 95% confidence intervals computed to determine prognostic values; log-rank test *p* < 0.05 was considered significant. Correlation of EDI with gene expression data was computed with Pearson correlation, and *q*-values computed using tail-area-based false discovery rate correction for multiple correlation analysis [19]. Test for significant differences among groups of a single variable was carried out using ANOVA or the Kruskal–Wallis test when appropriate. Test for trend was performed using the Jonckheere–Terpstra test. Test for association between categorical variables was carried out using Fisher’s exact test. Genomic instability was calculated as the proportion of copy number aberrations in a genome based on SNP6 data, as previously described [15]. A Sweave file and data are provided for reproducing our results, and our methods are also available as an R package, “EDI” (S1 Data).

## Results

### Development of a Computational Framework for Quantifying Intra-tumor Microenvironment Heterogeneity

**Fig 2:**
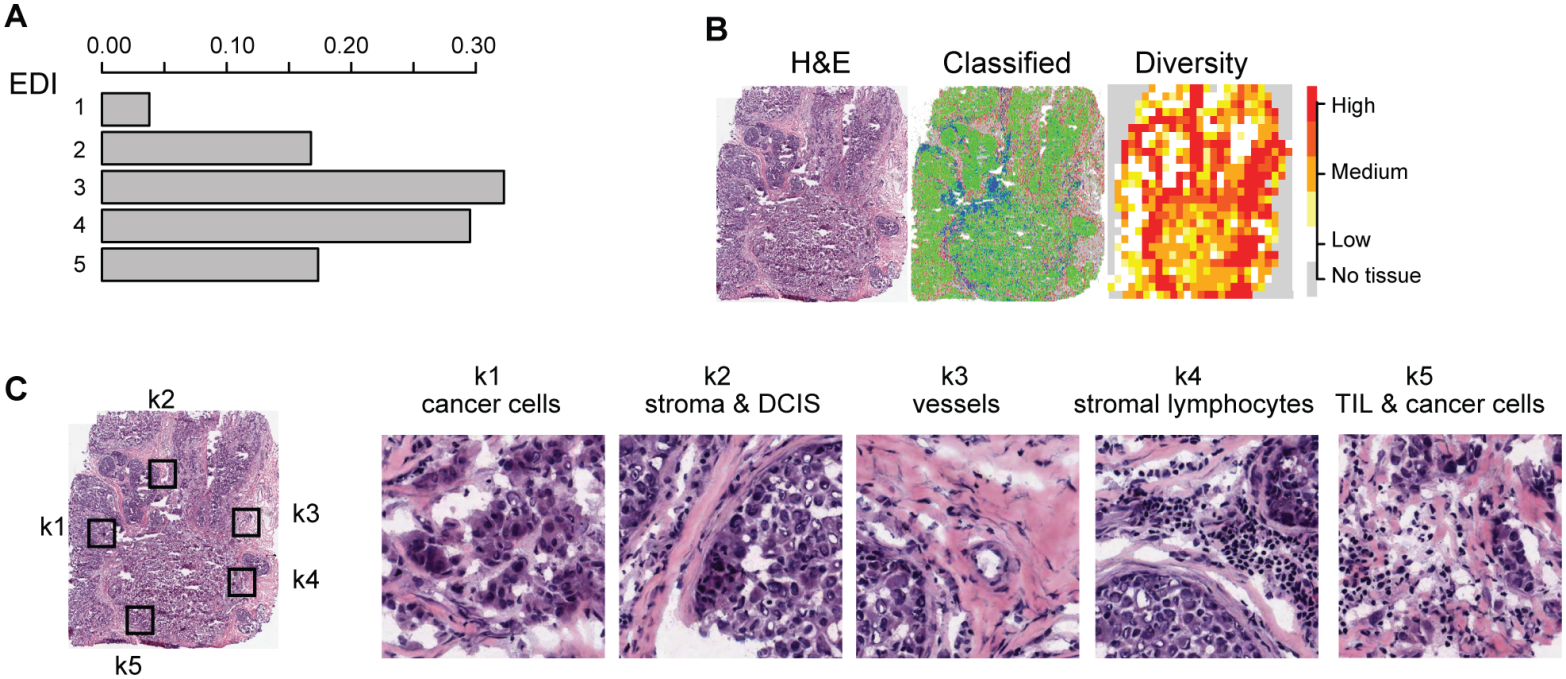
Application of EDI to 1,026 breast tumors from the METABRIC study. (A) The frequencies of EDI scores in breast tumors. (B) H&E staining, distribution of classified cells (green: cancer; blue: lymphocyte; red: stromal cells), and the heatmap of regional diversity scores for a tumor with the highest EDI score (EDI = 5). (C) Representative regions from each of the clusters k1–k5 are shown in a tumor with EDI = 5, with cluster k1 having the lowest diversity score and k5 the highest. By mapping regional clusters to the H&E image, we can begin to interpret these clusters with different cell diversity. We observed predominantly cancer cells in k1, increasingly more stromal cells and ductal in situ carcinoma cells (DCIS) in k2, and a vessel in k3. Cluster k4 features extensive stromal lymphocytes between ductal in situ carcinoma components, while k5 shows tumor-infiltrating lymphocytes (TIL) associated with invasive carcinoma cells.

To measure the spatial heterogeneity of the tumor microenvironment, we developed a fully automated computational approach to generate an index termed the ecosystem diversity index (EDI). This builds on our previously developed automated image analysis tool that identifies cancer cells, lymphocytes, and stromal cells including fibroblasts and endothelial cells, based on cell size and shape, in H&E-stained breast tumor sections [16] (Fig 1B). After cell identification, H&E sections are computationally divided into regions to examine the tumor spatial variability with statistical assessment (Methods). The Shannon diversity index [20], which measures species diversity in an ecosystem, was calculated for each dissected tumor region to define a local cellular diversity score, where a high value indicates a diverse ecosystem populated by many cell types and a low value indicates the dominance of a single cell type (Fig 1C and 1D). For each tumor, an EDI score was subsequently computed by examining the global differences in cellular diversity among local regions, using unsupervised Gaussian mixture clustering [21] of the regional diversity indices (Methods; Fig 1E). Application of this EDI measurement to H&E images of 1,026 primary breast tumors from the METABRIC study [15] (Fig 1A; Table 1) resulted in EDI scores ranging from 1 to 5, equating to the degree of tumor spatial heterogeneity for the breast tumors in this cohort, with 5 reflecting the highest degree of heterogeneity (Fig 2A). A low EDI score indicates a relatively homogeneous tumor, whereas a high EDI score suggests that diverse cancer habitats coexist in a tumor, and, hence, the tumor has a high degree of microenvironmental heterogeneity (Fig 2B and 2C). Spatial scale is a crucial parameter for EDI as a measure of spatial heterogeneity and was determined by three analytical experiments based on only histology data (S1 Text; S1 and S2 Figs).

**Table 1:** Distribution of EDI scores and clinicopathological characteristics in breast tumors.

Characteristic	Cohort 1		Cohort 2	
	EDI-Low	EDI-High	EDI-Low	EDI-High
**Number**	393	116	452	62
**Follow-up (months)**	116.9 (4.2–120)	120.0 (4.9–120)	59.6 (0.3–120)	51.8 (4.2–120)
**Age (years)**	59.3 (21.9–86.1)	59.6 (28.6–81.3)	60.7 (26.4–96.3)	63.6 (34.3–89.4)
**Tumor size**				
1 (≤2 cm)	164 (41.7%)	35 (30.2%)	149 (33.0%)	12 (19.4%)
2 (>2 cm, ≤5 cm)	209 (53.2%)	77 (66.4%)	267 (59.1%)	38 (61.3%)
3 (>5 cm)	20 (5.1%)	4 (3.4%)	33 (7.3%)	12 (19.4%)
**Node status**				
Negative (pN0)	197 (50.1%)	56 (48.3%)	212 (46.9%)	24 (38.7%)
Positive (pN1–pN3)	196 (49.9%)	60 (51.7%)	235 (52.0%)	37 (59.7%)
**Grade**				
1	46 (11.7%)	14 (12.1%)	43 (9.5%)	8 (12.9%)
2	142 (36.1%)	43 (37.1%)	163 (36.1%)	17 (27.4%)
3	194 (49.4%)	56 (48.3%)	224 (49.6%)	32 (51.6%)
**ER** *				
Positive	297 (75.6%)	83 (71.6%)	353 (78.1%)	40 (64.5%)
Negative	96 (24.4%)	33 (28.4%)	99 (21.9%)	22 (35.5%)

Data are given as median (range) or number (percent). Tumors with an EDI score of 5 are classified as EDI-high; tumors with an EDI score of 1–4 are classified EDI-low.

*Expression status defined by gene expression data. Three samples were discarded due to a small amount of tissue.

### High Intra-tumor Microenvironment Heterogeneity Identifies an Aggressive Subtype of Grade 3 Cancers

**Fig 3:**
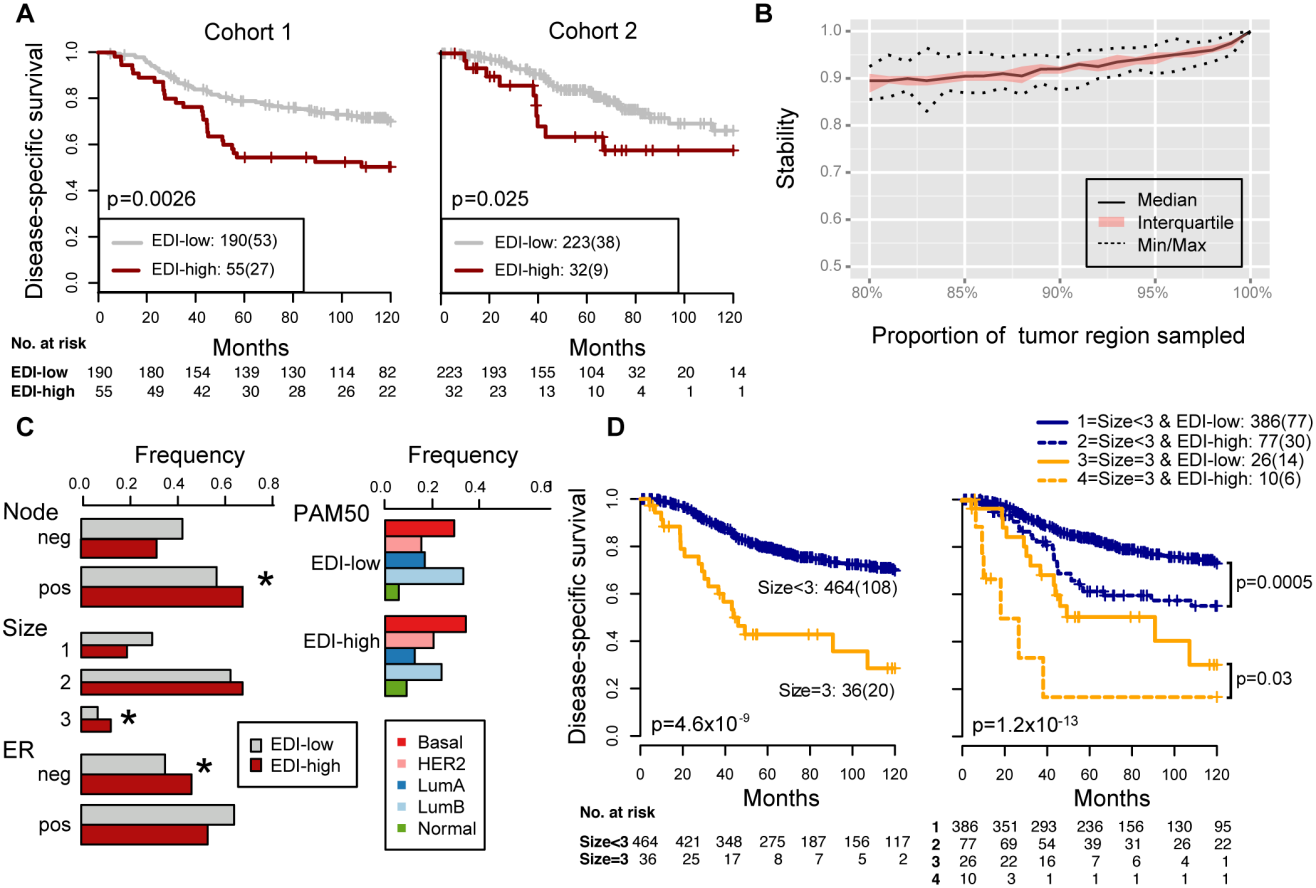
Reproducibility, stability, and independence of the EDI-high group in 507 grade 3 breast tumors. (A) Kaplan–Meier curves of disease-specific survival to illustrate the prognosis of EDI-high samples compared to other grade 3 samples in two independent patient cohorts. Shown below the graph are the number of patients (the number of disease-specific events) per group for EDI-low (grey) and EDI-high (red). (B) Agreement of the EDI subtyping between 100% data and resampling with progressively fewer tumor regions in 200 repeats. (C) Distribution of known subtypes in grade 3 tumors stratified by EDI; asterisks mark subtypes enriched in the EDI-high group. (D) Kaplan–Meier curves illustrating the duration of disease-specific survival according to tumor size (left) and improvement of stratification with the addition of EDI information (right).

To investigate the clinical implications of microenvironmental heterogeneity, we evaluated the prognostic value of EDI subtyping in two independent cohorts of breast cancer patients from the METABRIC study [15] (*n* = 510 and *n* = 516). We found that in high-grade (grade 3) breast cancers, tumors with the highest microenvironmental heterogeneity (EDI = 5, referred to subsequently as the EDI-high group, with all remaining tumors in the EDI-low group) had significantly worse disease-specific survival than the remaining patients in the test cohort (grade 3, *n* = 251, *p* = 0.0026, hazard ratio [HR] = 2.01, 95% CI 1.26–3.19; Table 2) and validation cohort (grade 3, *n* = 256, *p* = 0.025, HR = 2.24, 95% CI 1.08–4.65; Fig 3A). With the two cohorts combined, the EDI-high group accounted for 17.3% (88/507) of grade 3 breast cancers, with a 10-y disease-specific survival probability of 51% compared with 70% for the remaining grade 3 patients (Kaplan–Meier survival estimates; log-rank test of difference in survival, *p* < 0.001, HR = 2.12, 95% CI 1.44–3.13). Furthermore, the prognostic value of EDI was independent of the number of regions (multivariate survival analysis in grade 3 tumors: EDI, *p* = 0.004, HR = 1.89; number of regions, *p* = 0.26, HR = 1.29), and its correlation with prognosis remained statistically stable upon resampling with progressively fewer tumor regions built into the model (100%–80%; Figs 3B and S3), demonstrating the robustness of EDI subtyping. At variance with the results for grade 3 tumors, EDI was not associated with prognosis in low-grade tumors (grade 1 and 2, *p* = 0.42; S4 Fig). This observation was further validated using the genomic grade index (GGI) inferred from gene expression data [22], where EDI was associated with prognosis only in high-GGI tumors (S5 Fig).

**Table 2:** Univariate and multivariate analysis of the prognostic value of EDI for disease-specific survival in two cohorts of grade 3 breast cancer patients.

Analysis Group	Variable	Cohort 1 (*n* = 251)				Cohort 2 (*n* = 256)			
		Univariate Cox Regression Survival Analysis		Multivariate Cox Regression		Univariate Cox Regression Survival Analysis		Multivariate Cox Regression	
		HR (95% CI)	*p-*Value	HR (95% CI)	*p-*Value	HR (95% CI)	*p-*Value	HR (95% CI)	*p-*Value
**1**	EDI	2.01 (1.26–3.19)	0.0026**	1.74 (1.09–2.79)	0.019*	2.24 (1.08–4.65)	0.025*	2.27 (1.08–4.76)	0.029*
	Node	2.01 (1.25–3.24)	0.0034**	1.51 (0.92–2.48)	0.099	2.61 (1.29–5.26)	0.0054**	2.46 (1.17–5.15)	0.016*
	Size	2.01 (1.33–3.04)	<0.001**	2.02 (1.30–3.12)	0.0015**	3.03 (1.73–5.30)	<0.001**	2.41 (1.38–4.23)	0.0020**
	ER	0.58 (0.37–0.89)	0.013*	0.61 (0.39–0.96)	0.034*	0.55 (0.30–0.98)	0.042*	0.45 (0.24–0.82)	0.0090
**2**	EDI	2.01 (1.26–3.19)	0.0026**	2.09 (1.31–3.34)	0.0018**	2.24 (1.08–4.65)	0.025*	2.55 (1.21–5.38)	0.013*
	*TP53*	1.80 (1.15–2.81)	0.0081**	1.89 (1.20–3.00	0.0060**	1.92 (1.05–3.52)	0.029*	2.00 (1.03–3.87)	0.039*
	*APOBEC3B*	1.02 (0.85–0.21)	0.79	0.96 (0.80–1.14)	0.65	1.10 (0.87–1.39)	0.39	0.99 (0.76–1.28)	0.95
	Genomic instability	4.04 (0.59–27.27)	0.15	2.99 (0.49–18.27)	0.23	5.97 (0.48–73.93)	0.16	3.8 (0.30–47.96)	0.30
**3**	EDI + *TP53*	1.6 (1.30–1.97)	<0.001**	1.47 (1.17–1.84)	<0.001**	1.77 (1.28–2.48)	<0.001*	1.78 (1.26–2.52)	0.0011**
	Node	2.01 (1.25–3.24)	0.0034**	1.49 (0.91–1.84)	0.11	2.61 (1.29–5.26)	0.0054**	2.56 (1.22–5.35)	0.012
	Size	2.01 (1.33–3.04)	<0.001**	1.97 (1.27–3.01)	0.0023**	3.03 (1.73–5.30)	<0.001**	2.54 (1.45–4.46)	0.0011**
	ER	0.58 (0.37–0.89)	0.013*	0.75 (0.46–1.22)	0.25	0.55 (0.30–0.98)	0.042*	0.56 (0.30–1.05)	0.072

Three groups of analysis are presented. Group 1 includes EDI and classical clinical parameters that are found to be significant in univariate analysis, including lymph node status (Node), tumor size (Size), and ER status defined by microarray data. Group 2 includes EDI and cancer hallmark measures *TP53*, *APOBEC3B* expression, and genomic instability. Group 3 is a combined analysis of EDI and *TP53* mutation.

* *p* < 0.05;

** *p <* 0.01.

### High Intra-tumor Microenvironment Diversity Is Independent of Known Clinicopathological Variables in High-Grade Breast Cancer

We next performed a comprehensive comparison between EDI and know prognostic variables including lymph node status, tumor size, ER, HER2 status, molecular subtypes including PAM50 [23] and IntClust [15], and proportions of cancer, stromal cells, and lymphocytes in the tumors. Of these, only node status, tumor size, and ER status were associated with survival in grade 3 tumors in both cohorts (univariate Cox proportional hazards analysis; Table 2; S6 Fig). Also, when tested against all variables, the EDI-high group was found to be enriched with samples that are large in tumor size (>5 cm), lymph node positive, or ER negative, but not with any PAM50 or IntClust subtypes (*p <* 0.05, Fisher’s exact test; Figs 3C and S7). Although we found that the Shannon diversity index computed on the whole tumor was correlated with EDI (*p <* 0.001, Kruskal–Wallis test), it was not independently prognostic given EDI in high-grade tumors (grade 3: Shannon diversity index, *p* = 0.14, HR = 1.21, 95% CI 0.93–1.57; EDI, *p* = 0.009, HR = 1.8, 95% CI 1.15–2.80).

Subsequently, multivariate Cox proportional hazards analysis including node status, tumor size, ER status, and EDI demonstrated that EDI was independently prognostic in both cohorts of grade 3 breast cancer (Cohort 1, *p* = 0.019, HR = 1.74, 95% CI 1.09–2.79; Cohort 2, *p* = 0.029, HR = 2.27, 95% CI 1.08–4.76; Table 2).

Moreover, the addition of EDI to patient groups defined by tumor size resulted in new subgroups with substantially different disease-specific survival (EDI-high versus EDI-low within size 2: *p* = 0.004, HR = 1.96, 95% CI 1.22–3.16; size 3: *p* = 0.03, HR = 2.77, 95% CI 1.03–7.45; Fig 3D). This analysis highlighted a very aggressive tumor type with large tumor size (>5 cm) and high EDI with a 16% 5-y survival probability, whilst the rest of large size tumors have a 50% 5-y survival probability. Similarly for node status and ER status, EDI-high defined a more aggressive group within each subtype (node negative, *p* = 0.001; node positive, *p* = 0.017; ER negative, *p* = 0.008; ER positive, *p* = 0.017; S8 Fig). Thus, the prognostic value of EDI in high-grade breast cancers was independent of known clinical parameters, and EDI outperformed some well-known molecular prognostic signatures.

### High Intra-tumor Microenvironment Diversity and *TP53* Mutation Co-define an Aggressive Subset of Grade 3 Breast Cancers

**Fig 4:**
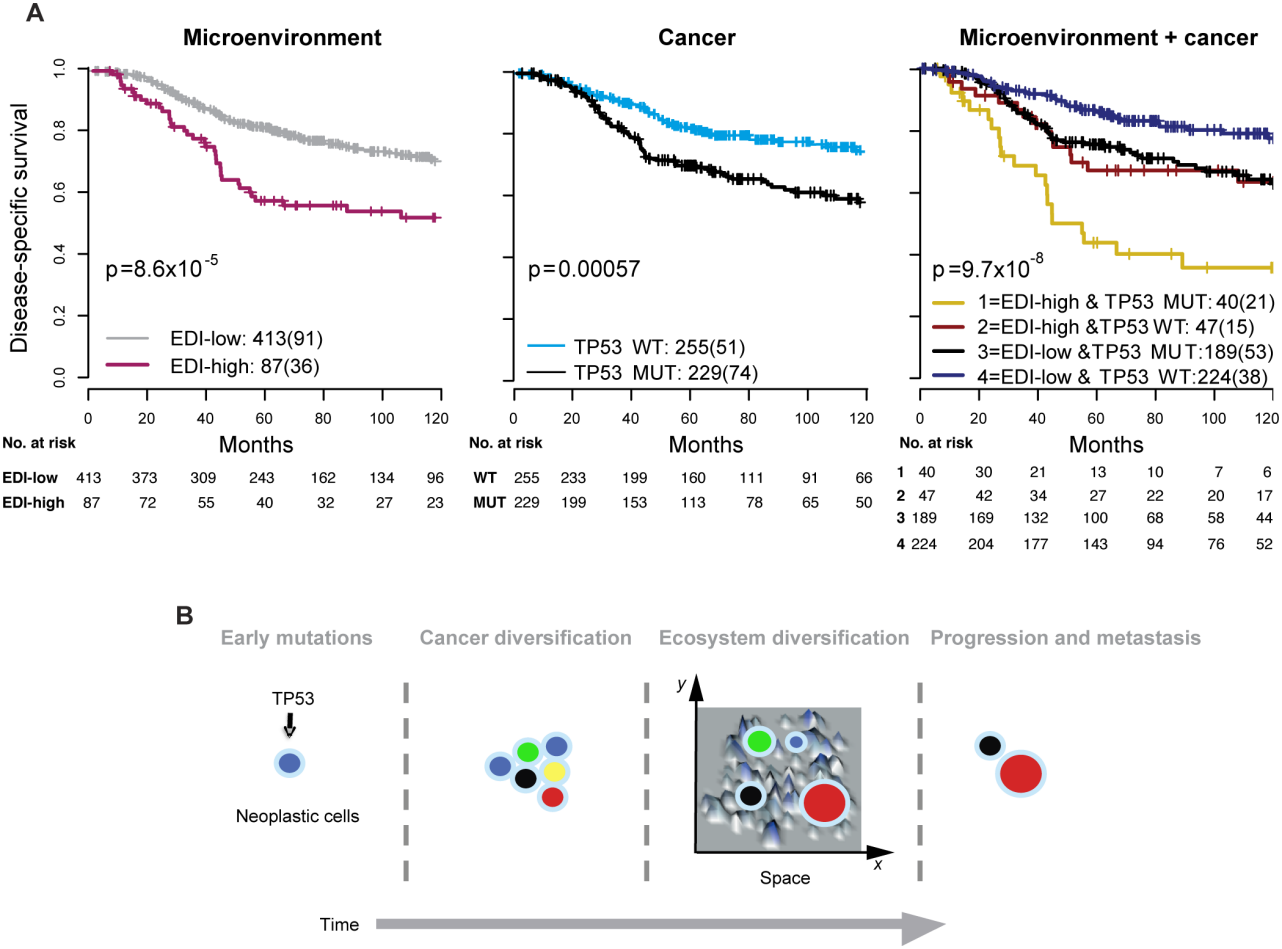
Accumulated prognostic value of microenvironmental heterogeneity and cancer *TP53* mutation in high-grade breast tumors. (A) Kaplan–Meier curves illustrating the duration of disease-specific survival according to microenvironmental heterogeneity (EDI, left panel), *TP53* mutation (middle panel), and both (right panel) in 507 grade 3 breast tumors. MUT, mutant; WT, wild-type. (B) Schematic diagram to illustrate the hypothesized temporal progression of invasive breast cancer with loss of *TP53* tumor suppressor functions as an early-stage event and spatial diversification of microenvironment as late-stage event.

We next asked whether EDI as a measure of microenvironmental heterogeneity correlated with cancer hallmarks that have previously been correlated with cancer progression, mutagenesis, and heterogeneity, including *APOBEC3B* expression [24], genomic instability [25,26], and *TP53* mutation [27,28]. Whilst we observed no significant correlation between EDI and any of these cancer hallmarks in grade 3 tumors or tumors of all grades (*p* > 0.1, ANOVA or Fisher’s exact test; S9 Fig), *TP53* mutation demonstrated a significant correlation with disease-specific survival (*p* < 0.001, HR = 1.85, 95% CI 1.29–2.65) in grade 3 tumors (Fig 4A). Taken together, EDI-high and *TP53* mutation co-defined an aggressive subtype of grade 3 cancers with a 10-y survival probability of 35%. This prognosis is significantly worse than that of the remaining grade 3 patients (Cohort 1, *p* < 0.001, HR = 2.88, 95% CI 1.66–4.99; Cohort 2, *p* = 0.001, HR = 4.2, 95% CI 1.65–10.68; Fig 4A for two cohorts combined). Interestingly, disease-specific survival for patients with either high ecosystem diversity or *TP53* mutation alone was not different (*p* = 0.74 combining two cohorts). The prognostic value of three-group stratification (low EDI and *TP53* wild-type, high EDI and *TP53* mutant, and all others) exceeded all known clinicopathological variables (multivariate analysis: Cohort 1, *p* < 0.001, HR = 1.47, 95% CI 1.17–1.84; Cohort 2, *p* = 0.0011, HR = 1.78, 95% CI 1.26–2.52; Table 2). In contrast, when tumors of all grades were considered, cancer hallmark measures were significantly associated with disease-specific survival (S10 Fig) and tumor grade (*p <* 0.001, Jonckheere–Terpstra test; S11 Fig). Therefore, EDI was independent of known cancer hallmarks and can co-define an aggressive subgroup of high-grade breast cancer together with *TP53* mutation (Fig 4B), highlighting the need to study genetic alterations in the context of the heterogeneity of the tumor microenvironment [3,29,30].

### High Intra-tumor Microenvironment Diversity Is Associated with Specific Genomic Alterations

**Fig 5:**
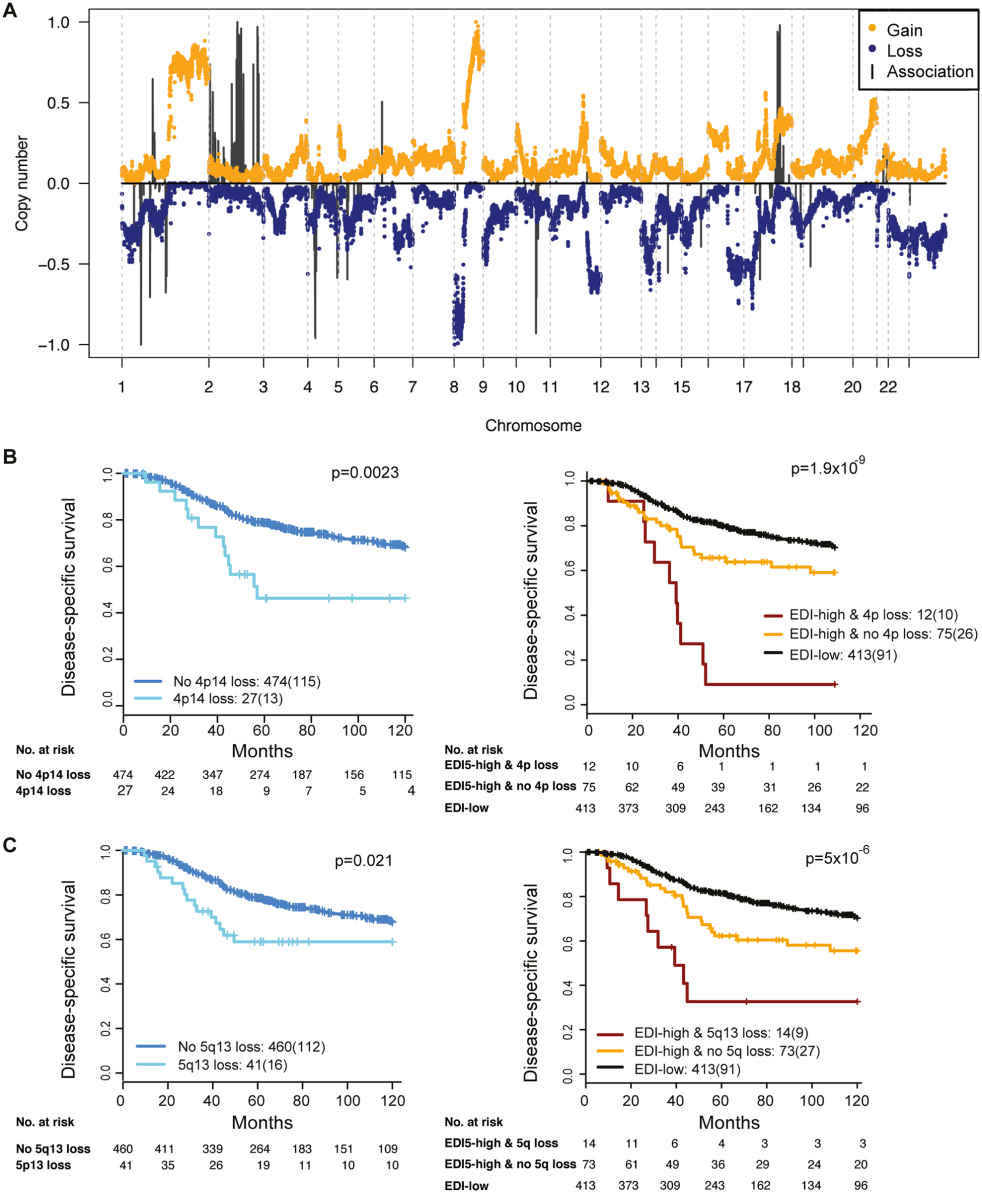
The relationship between ecological heterogeneity and cancer genomic aberrations in 507 grade 3 tumors. (A) Genome-wide copy number aberrations in grade 3 breast tumors and genomic coordinates of genes with copy number aberrations enriched in the EDI-high group. Lengths of black lines denote level of enrichment significance with copy number gains (above the horizontal line) or losses (below the horizontal line). (B) Kaplan–Meier curves illustrating the duration of disease-specific survival in grade 3 breast cancer patients according to copy number loss of the 4p14 region (left) and the EDI-high group with additional information of 4p14 copy number loss (right). (C) Kaplan–Meier curves illustrating the duration of disease-specific survival according to copy number loss of the 5q13 region (left) and the EDI-high group with additional information of 5q13 copy number loss (right).

To reveal additional molecular aberrations associated with microenvironmental heterogeneity, we integrated EDI with whole-genome mRNA expression and SNP6 DNA copy number data in grade 3 breast tumors. Whilst there were no differentially expressed genes between EDI-high and EDI-low tumors after multiple test correction, we identified a number of significant correlations of specific copy number alterations with EDI-high tumors (Fig 5A; S2 Table). These included higher frequency of loss of 1p35, 4p14, 5q13, and 10q23 and gains of 2q14 and 17q23. Akin to *TP53* mutation, copy number loss of 4p14 and 5q13 was able to substratify EDI-high, grade 3 cancer patients into aggressive subgroups (4p14 loss, *n* = 12, 9% 5-y survival; 5q13 loss, *n* = 14, 32% 5-y survival; Fig 5B and 5C). These stratifications were significant in both sample cohorts and independent of lymph node status and tumor size (Cohort 1: EDI + 4p14 loss, *p* < 0.001, HR = 4.89; EDI + 5q13 loss, *p* = 0.0033, HR = 3.02; Cohort 2: EDI + 4p14 loss, *p* = 0.014, HR = 8.13; EDI + 5q13 loss, *p* < 0.001, HR = 8.56; S3 Table). Application of a sparse regression model to fit EDI with genomic data, however, failed to generate satisfactory results, indicating that EDI cannot be explained based solely on genomic alterations (S1 Text).

## Discussion

Tumor histological grade remains one of the most powerful prognostic factors for breast cancer, and high-grade cancers (grade 3) are associated with a poor prognosis, with 10-y overall and relapse-free survival rates of around 40%–60% [31,32]. Development of additional breast cancer biomarkers to guide disease management on clinical diagnosis of this high-risk patient group is of paramount importance for effective patient management. Here we have developed a novel computational system for measuring the spatial heterogeneity of the tumor microenvironment based on high-throughput image analysis of cancer and microenvironmental cells. Our findings show that in the cohorts studied, grade 3 breast tumors that are characterized by high microenvironmental heterogeneity have a significantly worse 10-y disease-free survival rate (51%) than that of other grade 3 tumors (70%). Furthermore, this index is independent of known clinicopathological variables and prognostic molecular subtypes. In fact, a combined index of microenvironment heterogeneity and DNA alteration—namely, *TP53* mutation or copy number loss of 4p14 or 5q13—was able to improve the accuracy of prognosis in grade 3 breast cancer patients in our cohorts further, identifying subgroups of patients with disease-specific survival rates of 35%, 9%, and 32%, respectively.

Our unbiased approach thus opens the door to large-scale studies of human tumors and facilitates the integration of microenvironmental measures with cancer genomics. Our index, EDI, measures the variability of tumor composition along the spatial dimensions of a tumor and is therefore different from other methods such as the Shannon diversity index, which only measures tumor composition. Its prognostic value in grade 3 breast tumors was validated using samples stained independently at different laboratories, suggesting that the variability of H&E staining has limited impact on the prognostic associations of EDI. Diversification of the tumor ecosystem may become a new type of prognostic biomarker, in addition to cell abundance scores such as those that build on histological assessment of inflammatory cells and stromal infiltrate [33,34].

EDI as a measure of microenvironmental heterogeneity has superior prognostic value to known clinical parameters, molecular subtyping signatures, and cancer hallmarks measured in surgically resected high-grade tumors in these cohorts. Notably, none of the known cancer hallmarks, such as genomic instability, RNA expression, or genomics, could fully explain the new subtype with high diversification and its effective patient stratification in high-grade tumors. This clinical association was not observed in grade 1 and 2 cancers in the cohorts studied, suggesting that microenvironment heterogeneity may play a role in the temporal progression of high-grade, but not low-grade, breast cancers. On the other hand, *TP53* mutation was strongly associated with disease-specific deaths in both low- and high-grade cancers, consistent with a recent finding that driver mutations in *TP53* can be early and late events in breast cancer diversification [35]. Furthermore, in high-grade breast cancers, *TP53* mutation together with microenvironment heterogeneity co-defined an aggressive cancer with only a 35% 10-y survival probability. It is therefore plausible that whilst loss of *TP53* tumor suppressor functions occurs and cancer heterogeneity accumulates as disease progresses, additional microenvironment heterogeneity in advanced cancers aids further selection (Fig 4B). This hypothesis is in agreement with a recent elegant study that highlighted the importance of microenvironmental alterations that are mediated by non-cell-autonomous mechanisms that, together with genomic heterogeneity, drive tumor progression [36]. Whilst these hypotheses remain to be tested, our findings emphasize the importance of including analyses of microenvironmental heterogeneity in studies of cancer diversity.

By integrating our microenvironmental heterogeneity index with genome-wide copy number data, we found that breast cancers with heterogeneous microenvironments were enriched with specific genomic alterations, signifying the clinical implication and synergistic interaction of cancer cells and their surrounding microenvironments. Moreover, copy number loss of 4p14 and 5q13 aided further substratification of EDI-high tumors into poor prognosis subgroups in the cohorts studied. These ecosystem-associated genomic aberrations support the hypothesis of a cooperative relationship between cancer cells and their surrounding microenvironment and indicate that, consistent with mathematical modeling, their interactions may facilitate rapid cancer progression and that spatial heterogeneity of resources selects for metastasis [2,14,37]. Interestingly, we observed no differentially expressed genes between EDI-high and EDI-low tumors in these samples. This may be a consequence of whole-tumor sampling for the gene expression data, such that spatial signals are lost.

This study has a number of limitations. The motivation for our computational development was to use a data-driven model and measure the degree of spatial heterogeneity in tumor pathological specimens. In this model, only three major cell types in breast tumors were considered. Further sub-classification of the different types of stromal and immune cells by immunohistochemistry may add additional discriminatory value to our model. For dissecting spatial heterogeneity, we chose to use square regions with equal sizes. We found that EDI was correlated with the size of the region chosen for calculation of the Shannon diversity index, and as such the spatial heterogeneity is scale dependent. This phenomenon has been well described in a number of studies in ecology that show that a scale needs to be chosen that is appropriate for the ecological process under study [38,39], further highlighting the analogy between tumor studies and ecology. Similar to the recent observation that breast cancer subclonal heterogeneity is correlated with tumor size [35], we also found an association between microenvironmental heterogeneity and tumor size; hence, EDI may have more limited value in smaller tumors. However, small tumors were present in the EDI-high group, and addition of EDI within tumors grouped by size further stratified their prognosis. We found that EDI was prognostic only in grade 3 tumors in our study, which could be a limitation, given the possible discordance in grading between pathologists.

The identification of additional biomarkers in subgroups of patients that identify them as high risk is important for patient management and to avoid overtreatment for low-risk patients. We envision that the use of our measure of microenvironment heterogeneity, together with key genomic alterations, will enable the diagnosis of patients at very high risk of relapse and facilitate the enrollment of these patients into additional clinical trials for novel therapies or treatment intensification. Our novel computational approach provides a fully automated tool that is relatively easy to implement. Integration of this measure with genomic profiling provides additional prognostic information independent of known clinical parameters. The results of this study highlight the possibility of a grade-3-specific prognostic tool that may aid in further classification of high-grade breast cancer patients beyond standard assays such as ER and HER2 status.

## Supporting Information

S1 Data R package EDI. (GZ)

S1 Fig Selecting the optimal region size to measure tumor spatial variability. (A) Scatter plot of region size against average number of cells per region and average number of regions per tumor. Shadow box indicates favorable region sizes. (B) Clustering instability across different region sizes. Error bars denote standard deviation. Note region sizes of 200 and 166 μm show the highest stability. (C) Diversity scores with different region sizes in 20 randomly sampled tumors. (D) An example Q-Q plot to show how the clustering fits the distribution of data for a tumor (left); histogram of correlations of Q-Q plots for all tumors, showing a good fit of clustering for all samples (right).
(TIF)

S2 Fig Correlation plots of EDI computed with different spatial scales: *r* = 166, 200, 250, 333, 500 μm. (TIF)

S3 Fig Kaplan–Meier curves to show EDI stratification with decreasing amount of data in one of the sampling runs. (TIF)

S4 Fig Prognostic association of EDI with grade. (TIF)

S5 Fig EDI correlations with the genomic grade index. Kaplan–Meier curves to illustrate disease-specific survival differences in (A) breast cancers stratified by GGI; (B) low GGI tumors stratified by EDI; (C) high GGI tumors stratified by EDI.
(TIF)

S6 Fig Kaplan–Meier curves to compare EDI with microarray-based subtyping. Subtyping includes PAM50, IntClust, and known clinical parameters in grade 3 tumors including ER and HER2 status, node status, and tumor size. (A) Cohort 1; (B) Cohort 2.
(TIF)

S7 Fig Identifying specific subtypes enriched in the EDI-high patients with high-grade breast cancer with Fisher’s exact test. −Log *p*-values are depicted, and the solid horizontal line marks the significance threshold of *p* = 0.05.
(TIFF)

S8 Fig EDI further stratifies grade 3 patient groups defined by node status and ER status. Kaplan–Meier curves illustrating the duration of disease-specific survival according to (A) node status and (B) ER status without (left) or with (right) the addition of EDI information.
(TIF)

S9 Fig Correlation between EDI and cancer heterogeneity parameters. Boxplots show the correlation between EDI and cancer heterogeneity parameters including genomic instability (GI), APOBEC3B expression, and *TP53* mutation in grade 3 tumors (G3, first row) and in tumors of all grades (G1−3, second row); *p*-values produced using ANOVA.
(TIF)

S10 Fig Prognostic association of EDI and cancer heterogeneity parameters. Kaplan–Meier curves illustrating the duration of disease-specific survival according to EDI, genomic instability (GI), *APOBEC3B* expression, and *TP53* mutation in breast tumors of all grades (all, first column), high-grade breast tumors (grade 3, second column), and low-grade breast tumors (grade 1 and 2, third column). Genomic instability and *APOBEC3B* expression were dichotomized by their 25th and 75th percentiles. Number of patients per group is shown in the legend, together with the number of disease-specific deaths in brackets.
(TIF)

S11 Fig Correlation between EDI and clinical parameters. Boxplots show the correlation between clinical parameters, including tumor grade, tumor size, and node status, and heterogeneity measurements including EDI, genomic instability (GI), and *APOBEC3B* expression. Node status: 0, negative; 1, positive; tumor size: 1, 0–2 cm; 2, 2.1–5 cm; 3, >5 cm; *p*-values produced using ANOVA.
(TIF)

S1 Table Details of METABRIC primary tumor cohort. Node status: 0, negative; 1, positive; tumor size: 1, 0–2 cm; 2, 2.1–5 cm; 3, >5 cm; HER2 SNP6: 2, amplification; 1, gain; 0, no copy number change; -1, loss; -2, deletion; NA, not available. HER2 status was determined through HER2 SNP6 copy number data.
(PDF)

S2 Table Copy number alterations significantly enriched in EDI-high, grade 3 breast tumors. (PDF)

S3 Table Prognostic significance of 4p14 and 5q13 loss and EDI groups in independent subsets of grade 3 breast tumors in univariate and multivariate Cox regression with node status and tumor size. (DOCX)

S1 Text Supplementary methods. (DOCX)

S2 Text Sweave file for reproducibility. (PDF)

S3 Text STARD checklist. (DOCX)

## Abbreviations

EDI: ecosystem diversity index
ER: estrogen receptor
GGI: genomic grade index
H&E: hematoxylin and eosin
HR: hazard ratio
