# Supplementary figures and images for: Microenvironmental Heterogeneity Parallels Breast Cancer Progression: A Histology–Genomic Integration Analysis
Source: PLoS Med. 2016 Feb 16;13(2):e1001961. doi: 10.1371/journal.pmed.1001961 (PMC4755617; doi:10.1371/journal.pmed.1001961)

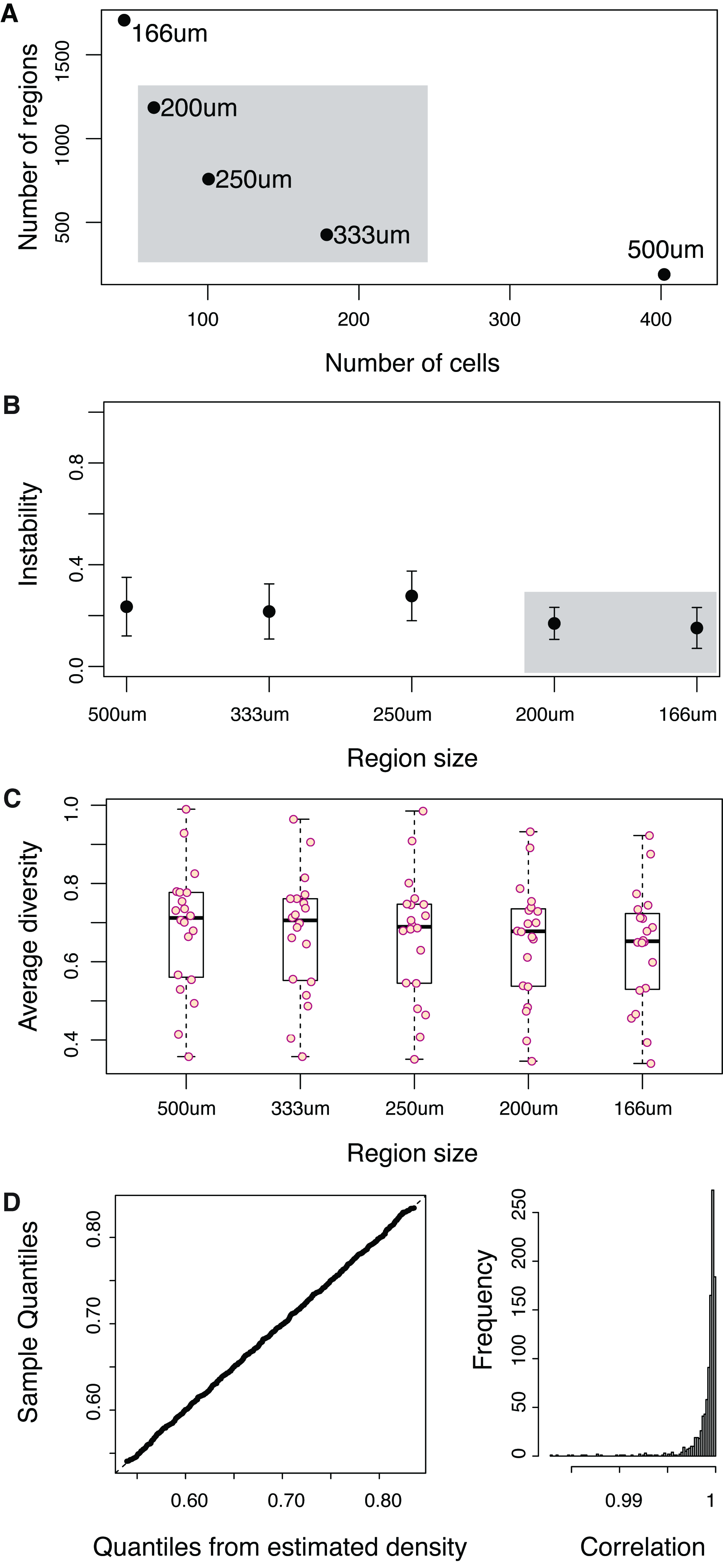

Supplement: S1 Fig — (A) Scatter plot of region size against average number of cells per region and average number of regions per tumor. Shadow box indicates favorable region sizes. (B) Clustering instability across different region sizes. Error bars denote standard deviation. Note region sizes of 200 and 166 μm show the highest stability. (C) Diversity scores with different region sizes in 20 randomly sampled tumors. (D) An example Q-Q plot to show how the clustering fits the distribution of data for a tumor (left); histogram of correlations of Q-Q plots for all tumors, showing a good fit of clustering for all samples (right). (TIF) [file pmed.1001961.s002.tif]

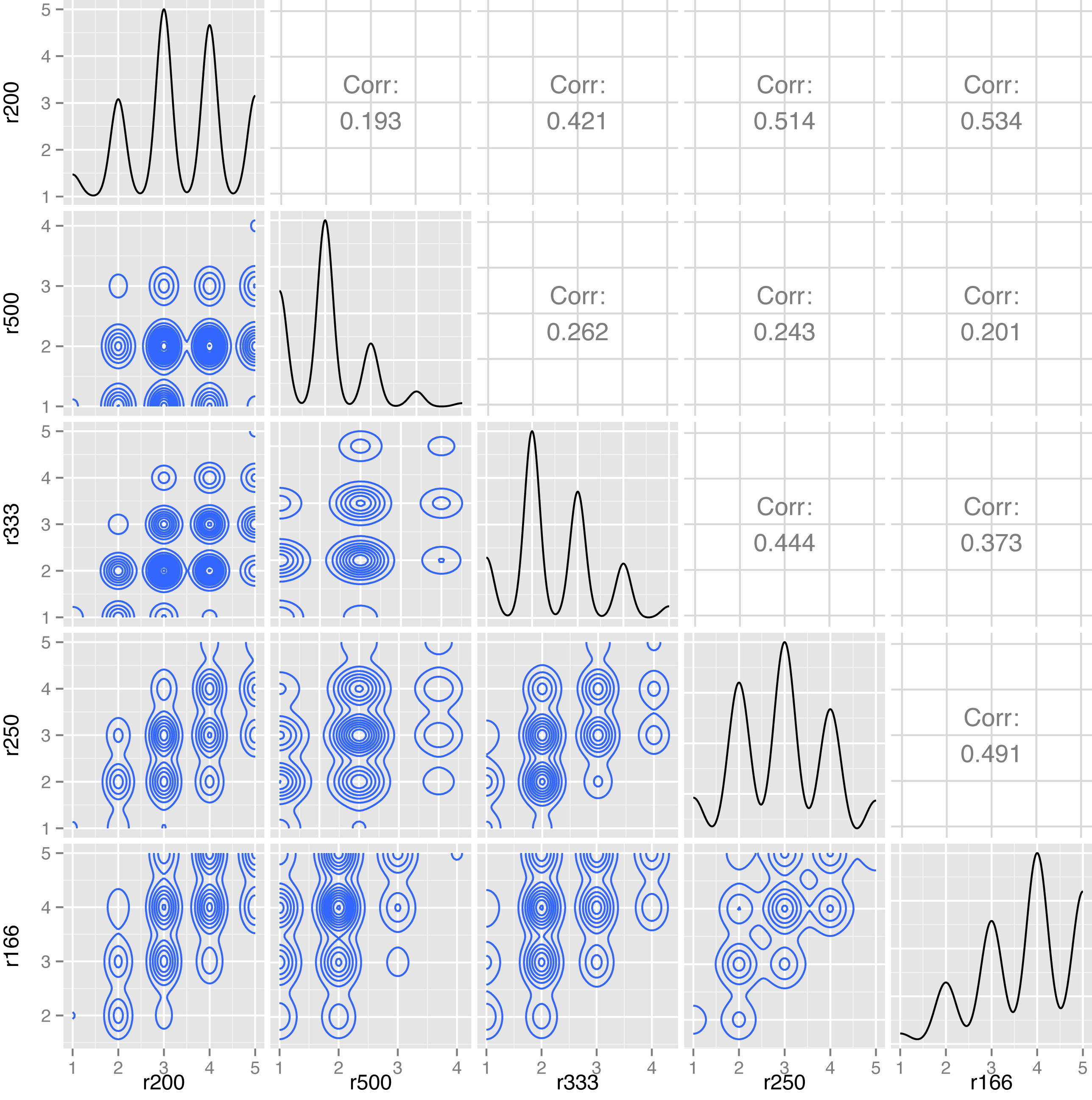

Supplement: S2 Fig — (TIF) [file pmed.1001961.s003.tif]

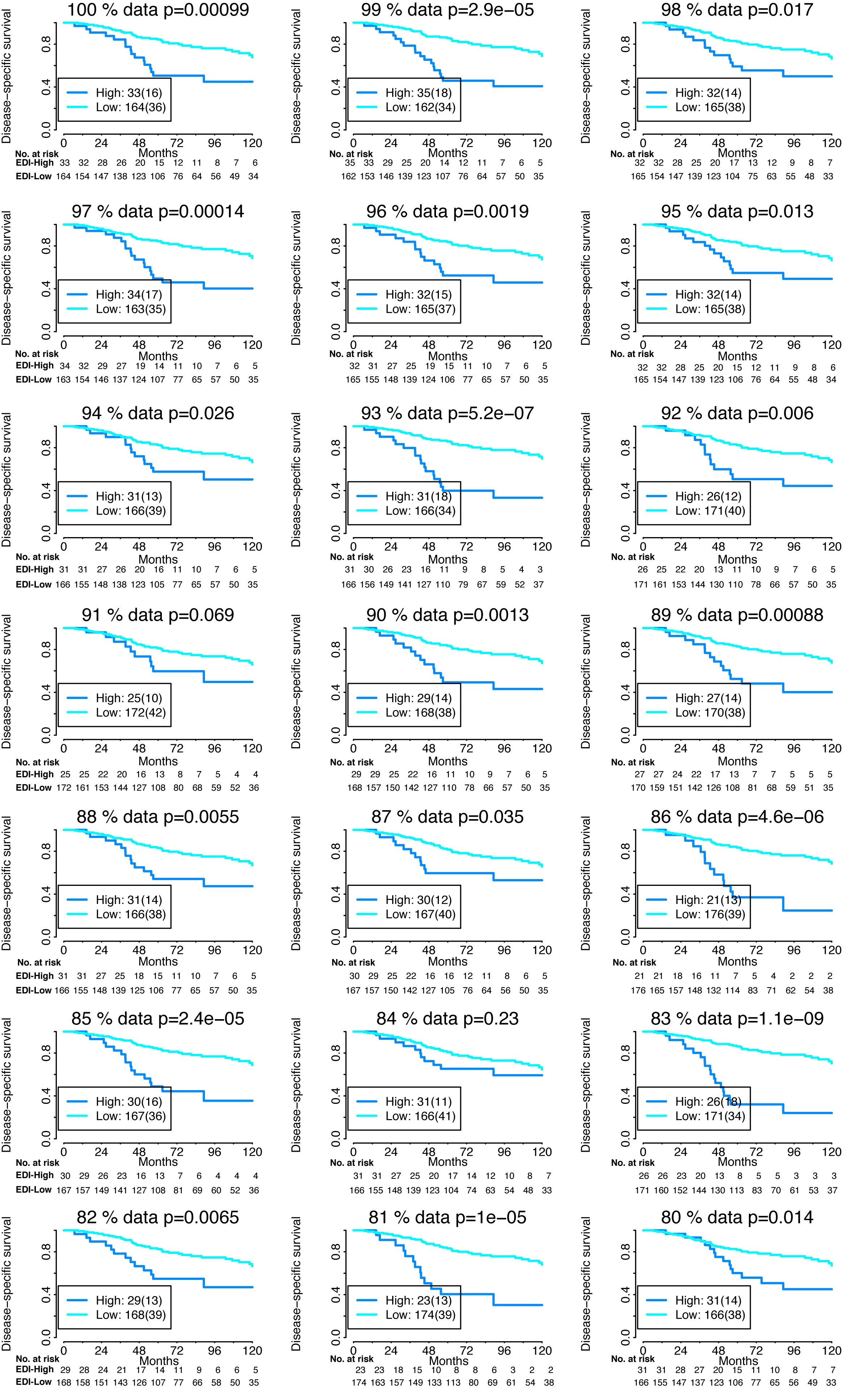

Supplement: S3 Fig — (TIF) [file pmed.1001961.s004.tif]

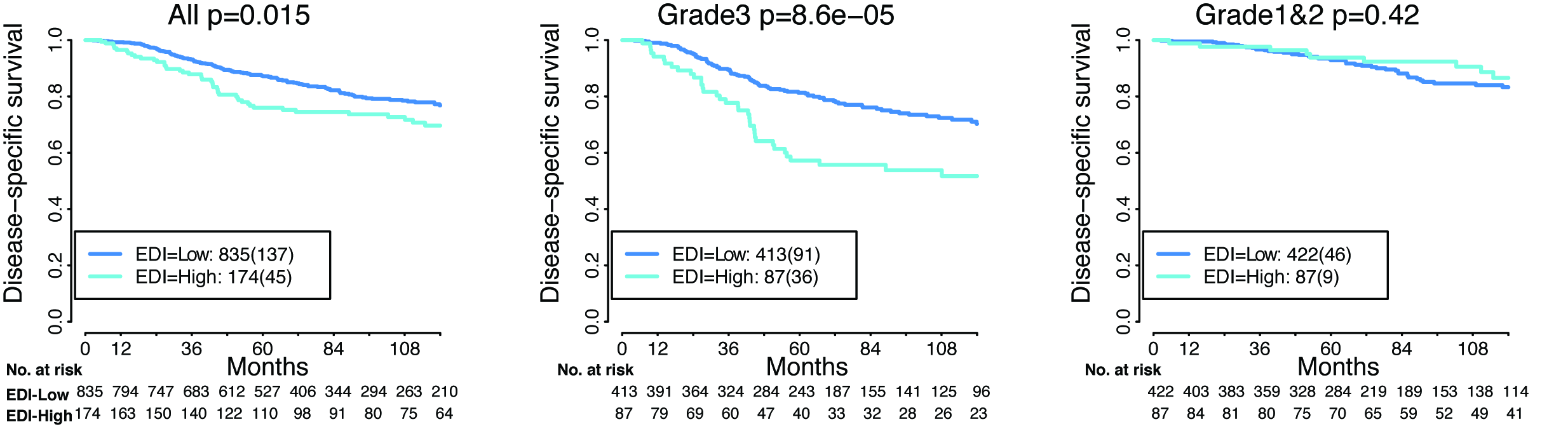

Supplement: S4 Fig — (TIF) [file pmed.1001961.s005.tif]

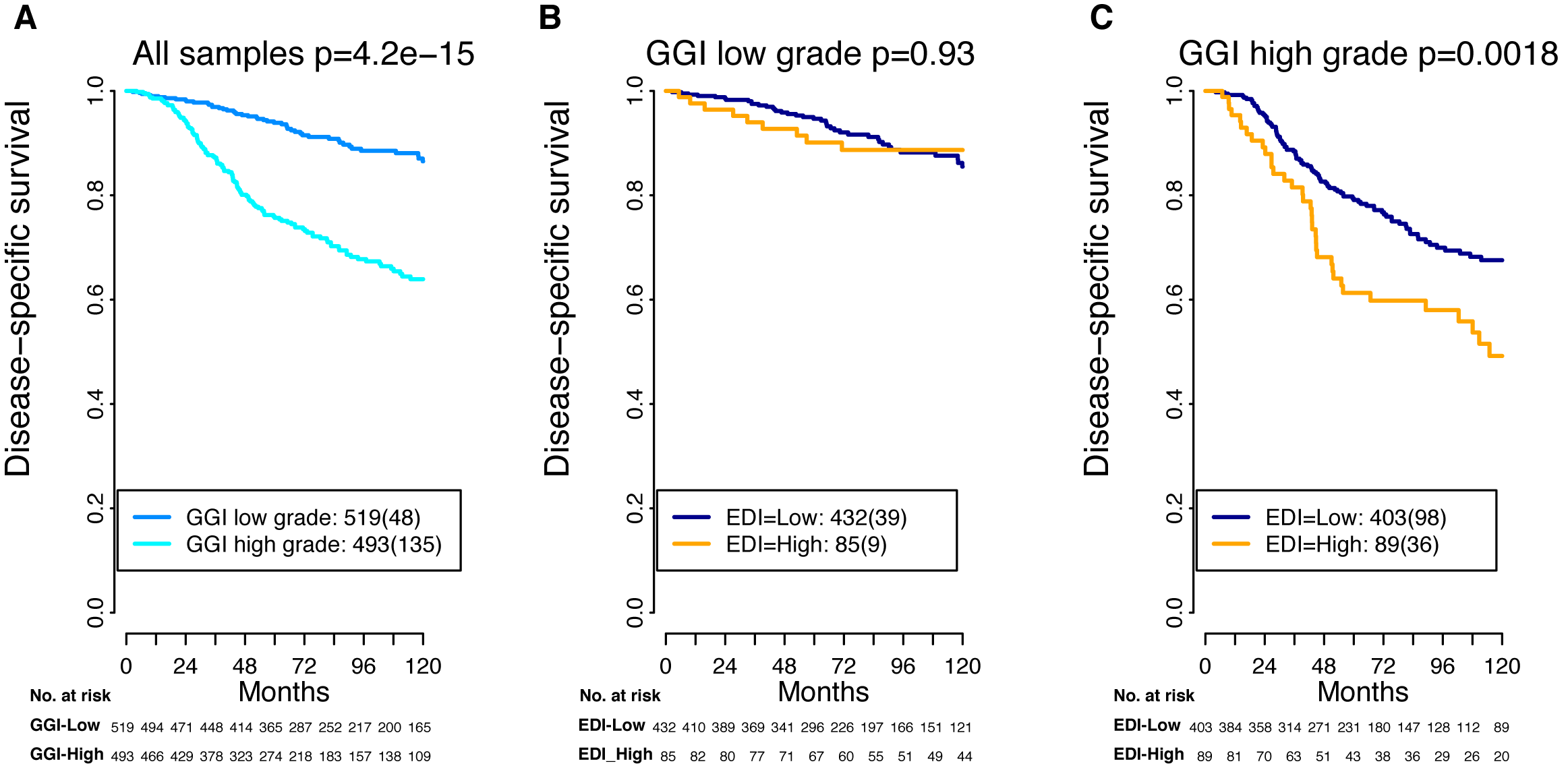

Supplement: S5 Fig — Kaplan–Meier curves to illustrate disease-specific survival differences in (A) breast cancers stratified by GGI; (B) low GGI tumors stratified by EDI; (C) high GGI tumors stratified by EDI. (TIF) [file pmed.1001961.s006.tif]

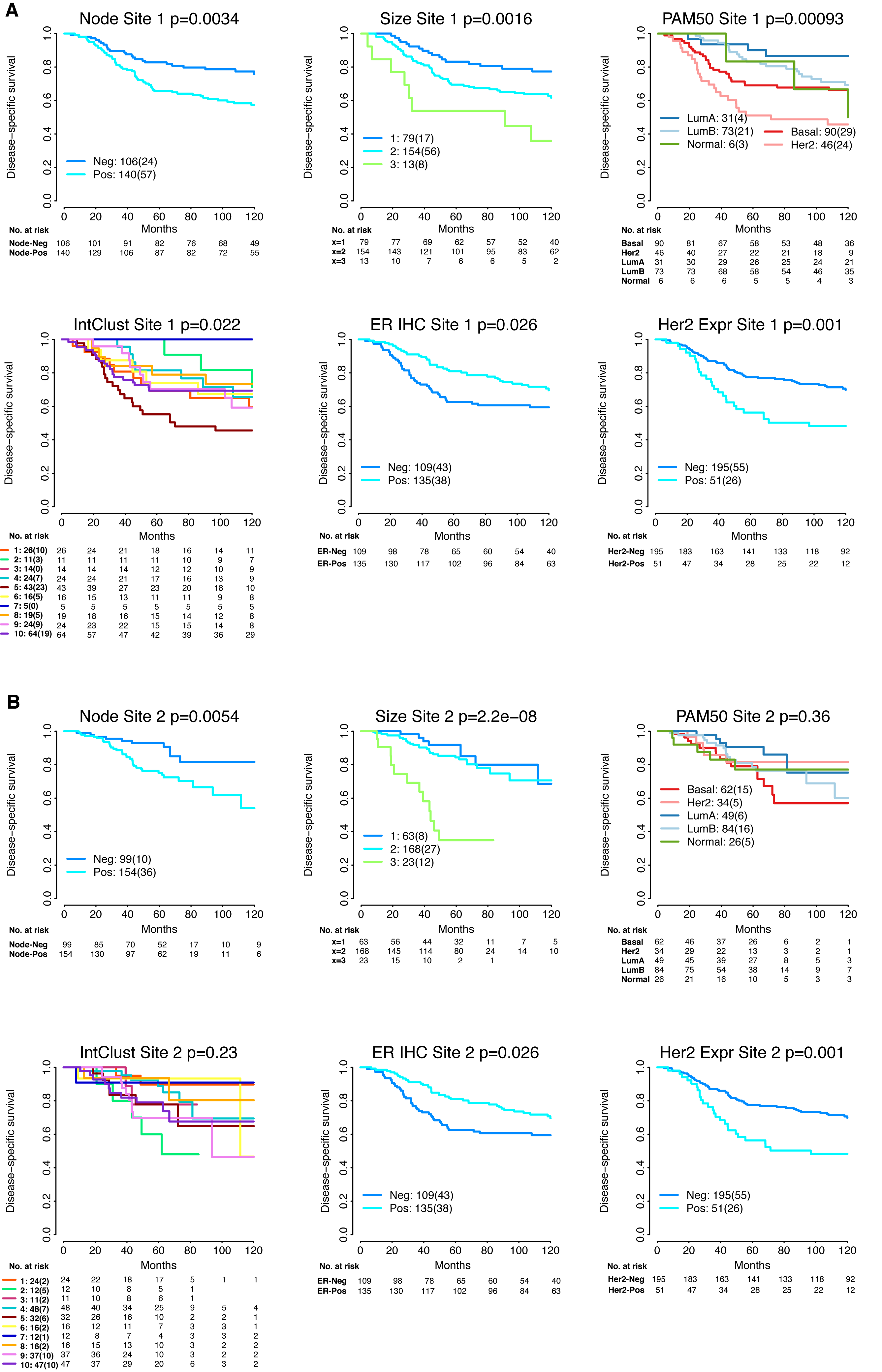

Supplement: S6 Fig — Subtyping includes PAM50, IntClust, and known clinical parameters in grade 3 tumors including ER and HER2 status, node status, and tumor size. (A) Cohort 1; (B) Cohort 2. (TIF) [file pmed.1001961.s007.tif]

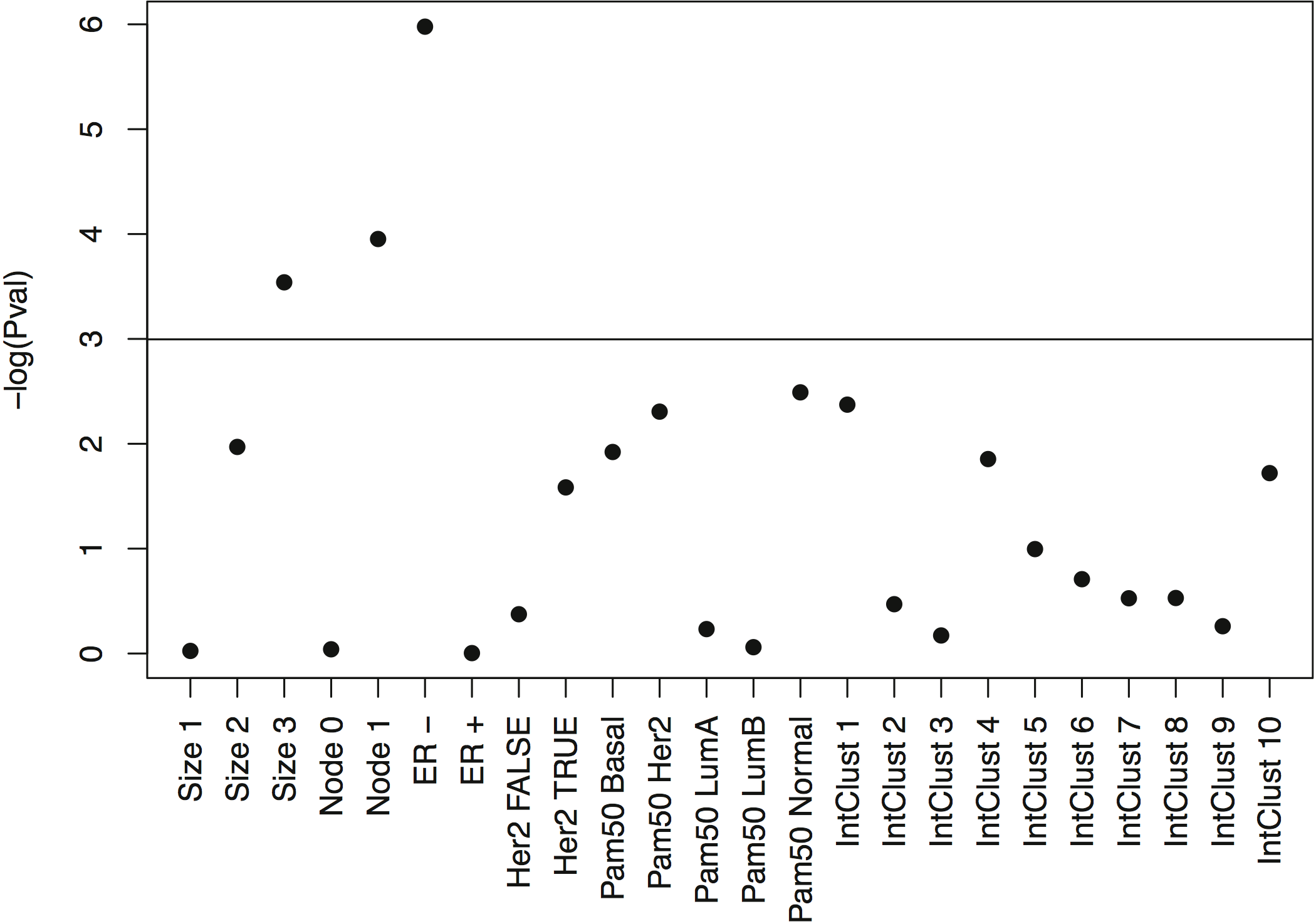

Supplement: S7 Fig — −Log p-values are depicted, and the solid horizontal line marks the significance threshold of p = 0.05. (TIFF) [file pmed.1001961.s008.tiff]

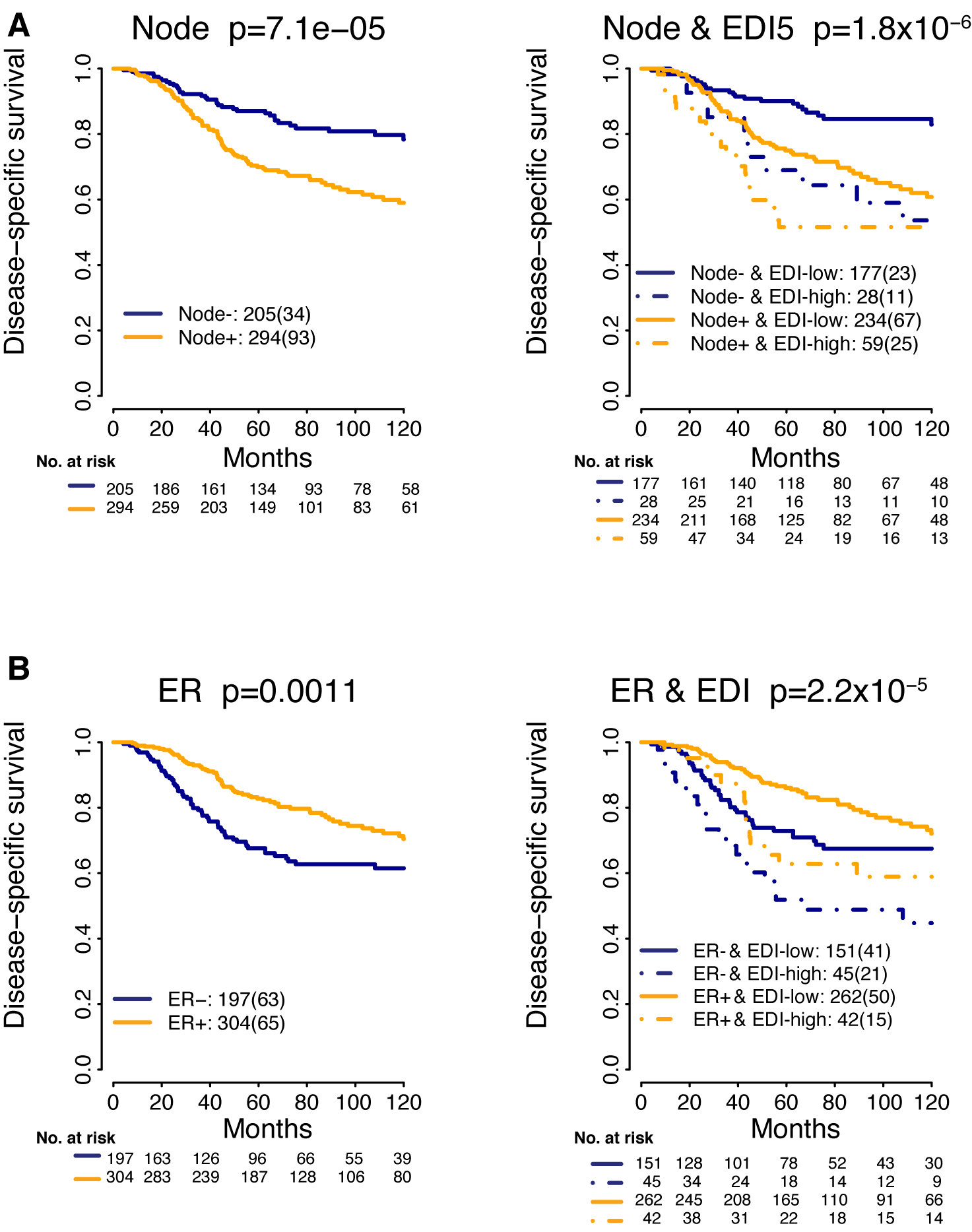

Supplement: S8 Fig — Kaplan–Meier curves illustrating the duration of disease-specific survival according to (A) node status and (B) ER status without (left) or with (right) the addition of EDI information. (TIF) [file pmed.1001961.s009.tif]

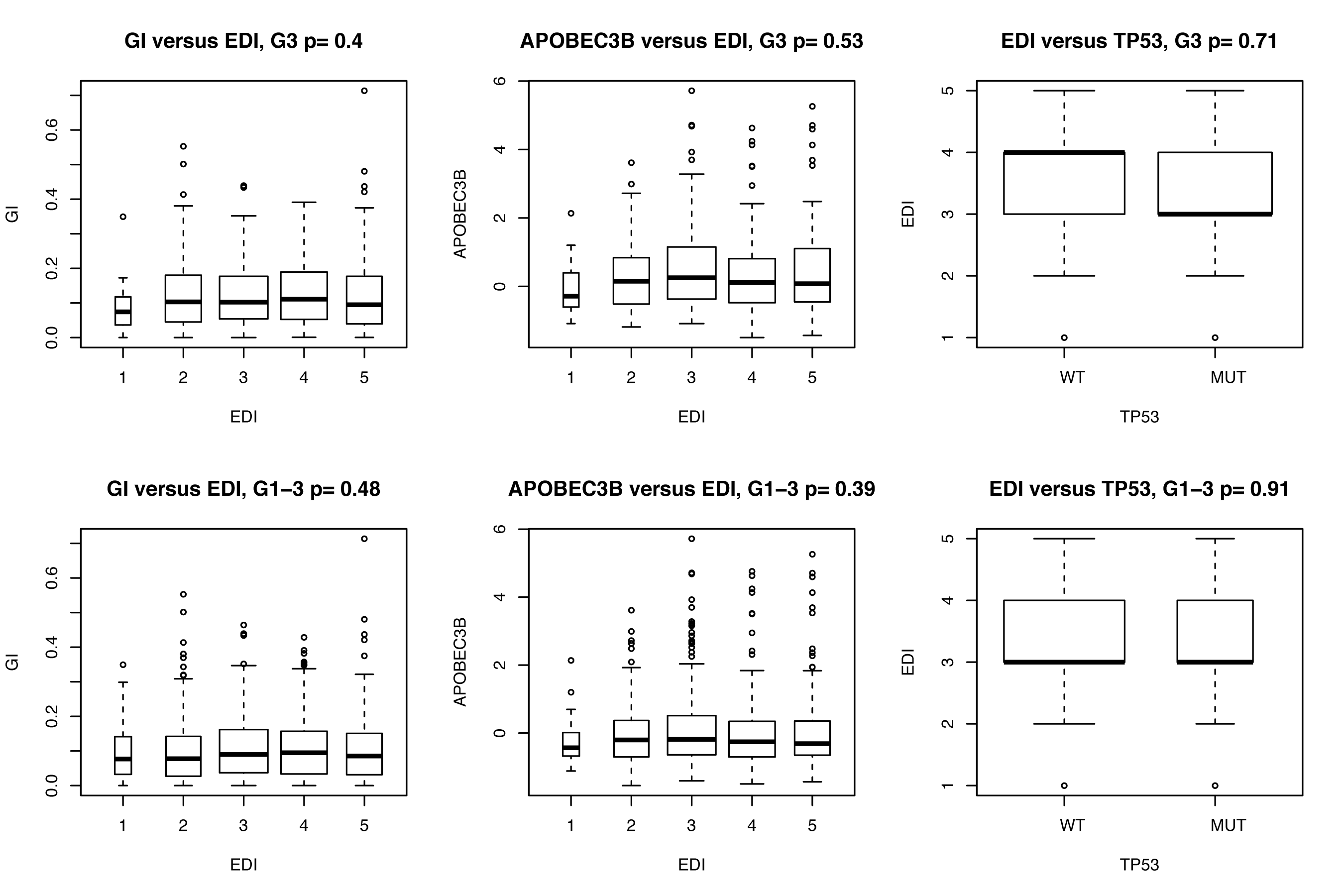

Supplement: S9 Fig — Boxplots show the correlation between EDI and cancer heterogeneity parameters including genomic instability (GI), APOBEC3B expression, and TP53 mutation in grade 3 tumors (G3, first row) and in tumors of all grades (G1−3, second row); p-values produced using ANOVA. (TIF) [file pmed.1001961.s010.tif]

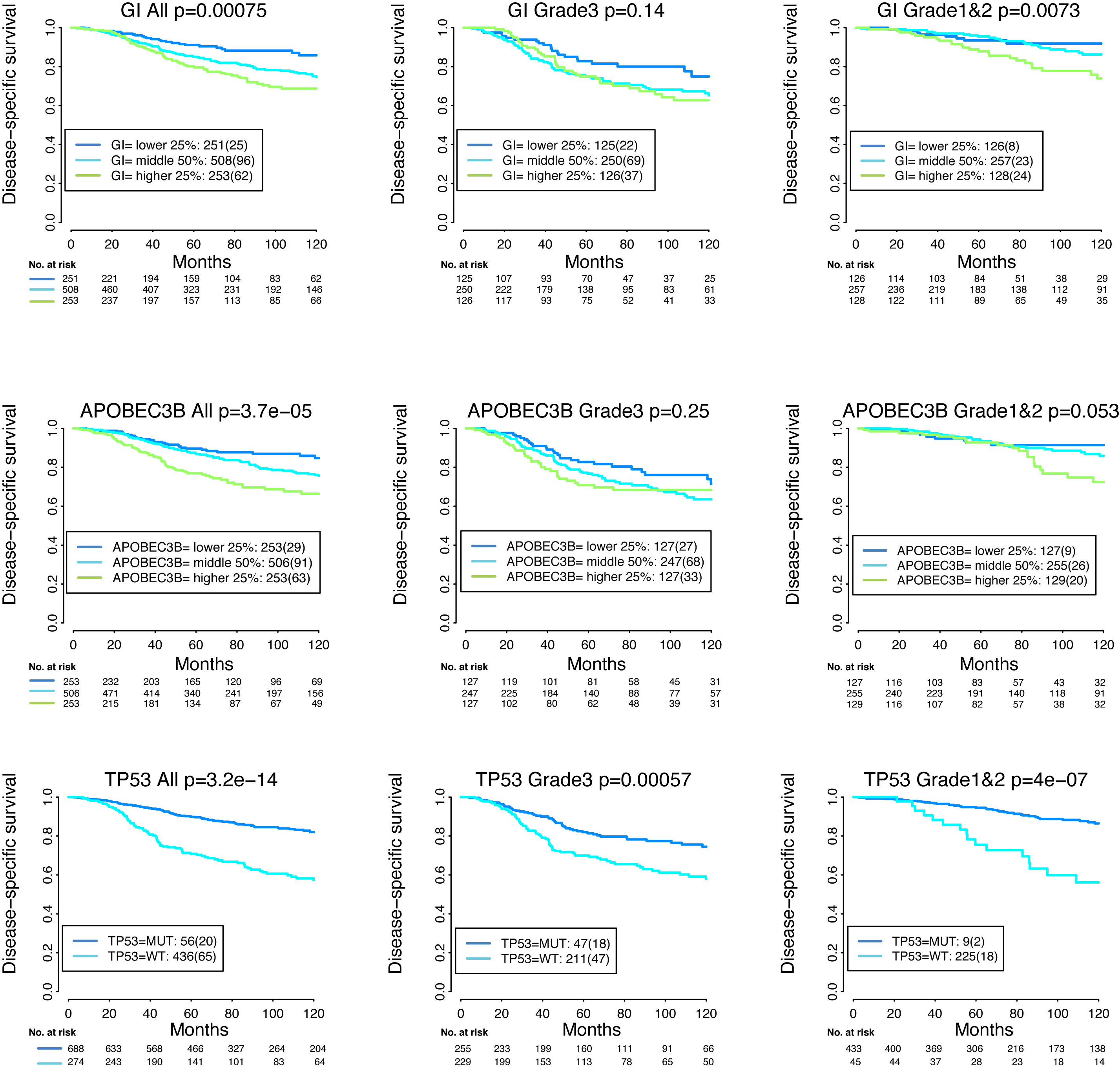

Supplement: S10 Fig — Kaplan–Meier curves illustrating the duration of disease-specific survival according to EDI, genomic instability (GI), APOBEC3B expression, and TP53 mutation in breast tumors of all grades (all, first column), high-grade breast tumors (grade 3, second column), and low-grade breast tumors (grade 1 and 2, third column). Genomic instability and APOBEC3B expression were dichotomized by their 25th and 75th percentiles. Number of patients per group is shown in the legend, together with the number of disease-specific deaths in brackets. (TIF) [file pmed.1001961.s011.tif]

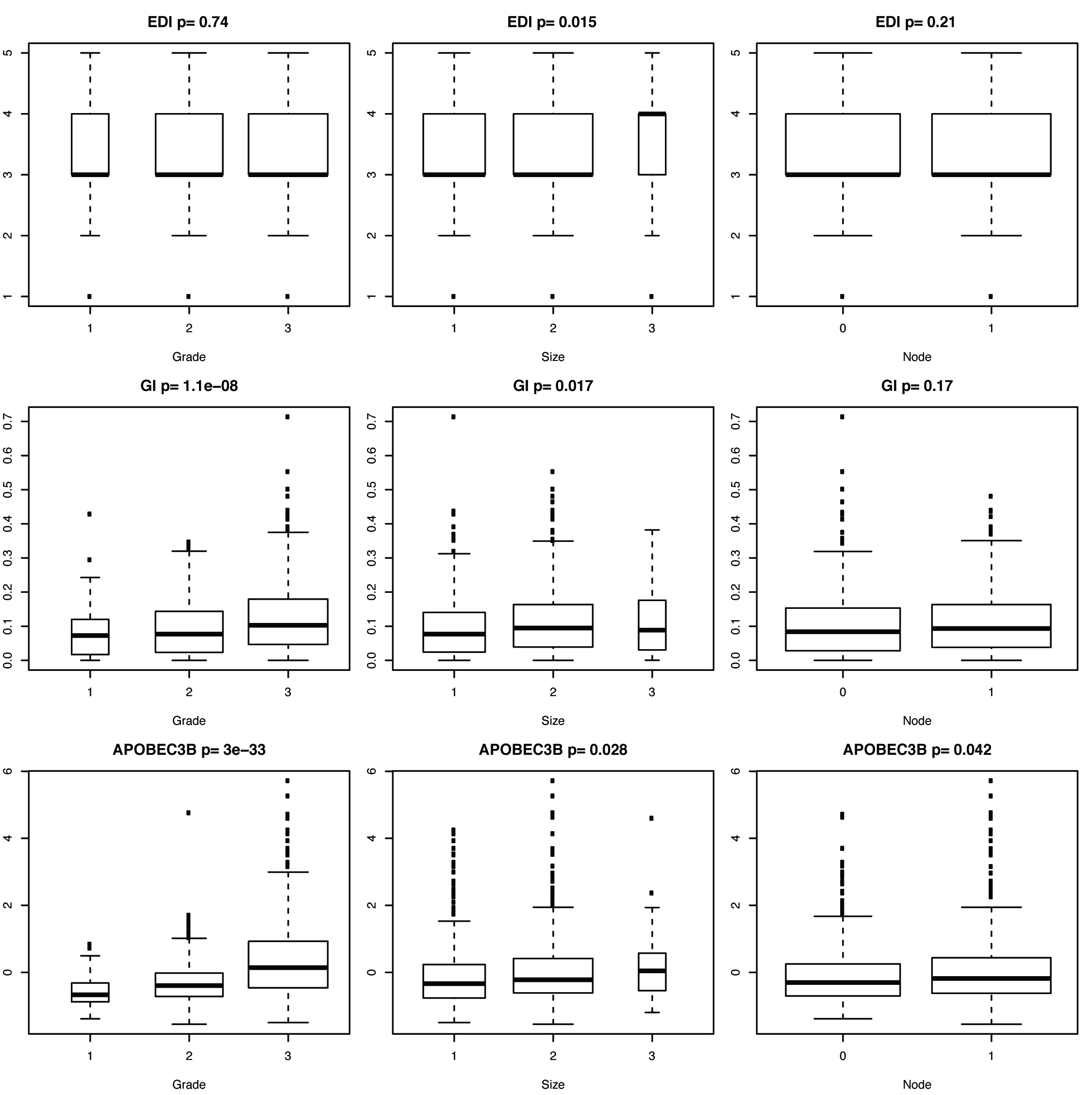

Supplement: S11 Fig — Boxplots show the correlation between clinical parameters, including tumor grade, tumor size, and node status, and heterogeneity measurements including EDI, genomic instability (GI), and APOBEC3B expression. Node status: 0, negative; 1, positive; tumor size: 1, 0–2 cm; 2, 2.1–5 cm; 3, >5 cm; p-values produced using ANOVA. (TIF) [file pmed.1001961.s012.tif]
