# Supplementary material for: Microenvironmental Heterogeneity Parallels Breast Cancer Progression: A Histology–Genomic Integration Analysis
Source: PLoS Med. 2016 Feb 16;13(2):e1001961. doi: 10.1371/journal.pmed.1001961 (PMC4755617; doi:10.1371/journal.pmed.1001961)
Supplement: S1 Table — Node status: 0, negative; 1, positive; tumor size: 1, 0–2 cm; 2, 2.1–5 cm; 3, >5 cm; HER2 SNP6: 2, amplification; 1, gain; 0, no copy number change; -1, loss; -2, deletion; NA, not available. HER2 status was determined through HER2 SNP6 copy number data. (PDF) [file pmed.1001961.s013.pdf]

Supplementary Table 1. Details of METABRIC primary tumor cohort

Sample.ID	Cohort	EDI	node	size	grade	ER_IHC	HER2_IHC	HER2_SNP6	TP53	histological_type	ER.Expr	PR.Expr	HER2.Expr	Pam50_Subtype	IntClustMemb	TP53 mutation status	Coding.description	Protein.change
MB-1000	1	2	1	1	2	pos	1	1	0	OTHER INVASIVE	+	+	-	LumB	1	0		
MB-1001	1	2	0	1	2	pos	3	2	0	IDC+ILC	+	-	+	LumA	5	0		
MB-1002	1	4	0	1	2	pos	1	-1	0	IDC	+	+	-	LumB	4	0		
MB-1003	1	3	1	2	2	pos	1	0	0	IDC	+	+	-	Normal	3	0		
MB-1004	1	3	1	2	2	pos	1	0	0	IDC	+	-	-	LumA	8	0		
MB-1005	1	4	1	2	1	pos	1	0	0	IDC	+	-	-	LumB	8	0		
MB-1006	1	4	1	1	1	pos	1	0	0	IDC-TUB	+	+	-	LumA	3	0		
MB-1007	1	4	1	1	3	neg	3	2	1	IDC	-	-	-	Basal	10	1	c.672G>C	p.E224D
MB-1008	1	4	0	2	3	neg	1	0	0	IDC	-	-	-	Basal	4	0		
MB-1009	1	4	1	2	3	neg	1	0	0	IDC	-	-	-	Basal	10	0		
MB-1010	1	4	0	2	1	pos	1	0	0	IDC	+	+	-	LumA	7	0		
MB-1011	1	4	0	2	3	pos	1	1	1	IDC	+	+	-	LumB	9	1	c.1022_1076del55	p.?
MB-1012	1	3	0	2	3	neg	1	0	1	IDC	-	-	-	Basal	10	1	c.267_268ins2	p.?
MB-1013	1	3	0	1	1	pos	3	0	0	IDC	+	+	-	LumA	8	0		
MB-1014	1	4	1	1	3	pos	1	0	1	IDC	+	+	-	Basal	1	1	c.131del1	p.?
MB-1015	1	4	0	2	3	pos	1	0	0	IDC	+	+	-	LumB	8	0		
MB-1017	1	5	1	2	3	neg	1	0	1	IDC	-	-	-	Basal	10	1	c.476C>T	p.A159V
MB-1018	1	5	1	2	2	neg	NA	0	0	IDC	+	-	-	Her2	9	0		
MB-1019	1	5	0	2	2	pos	1	0	0	IDC	+	+	-	LumB	3	0		
MB-1020	1	4	1	2	3	neg	3	2	1	IDC	-	-	+	Her2	5	1	c.1045_1068del24	p.?
MB-1022	1	4	0	2	2	pos	1	0	0	IDC	+	+	-	LumB	8	0		
MB-1023	1	5	1	2	3	neg	1	0	1	IDC	-	-	-	Basal	10	1	c.578A>G	p.H193R
MB-1024	1	4	0	1	3	pos	1	0	0	IDC	+	-	-	LumB	1	0		
MB-1025	1	4	0	1	3	neg	1	0	0	IDC	-	-	-	Basal	4	0		
MB-1026	1	4	1	2	2	pos	1	2	0	IDC	+	+	-	LumB	3	0		
MB-1027	1	3	1	1	3	neg	3	2	1	IDC	-	-	+	Her2	5	1	c.1025G>C	p.R342P
MB-1028	1	3	1	2	2	pos	3	0	0	IDC	+	-	-	LumB	1	0		
MB-1029	1	5	0	2	1	pos	1	-1	1	IDC	+	+	-	LumA	4	1	c.991C>T	p.Q331X
MB-1030	1	4	1	3	2	pos	1	0	0	IDC+ILC	-	-	-	Basal	4	0		
MB-1031	1	5	1	2	3	neg	3	0	1	IDC	-	-	-	Basal	10	1	c.517G>T	p.V173L
MB-1032	1	4	0	2	1	pos	1	0	0	IDC	+	+	-	LumA	3	0		
MB-1034	1	5	0	2	2	pos	1	0	0	IDC-MUC	+	+	-	LumA	3	0		
MB-1035	1	4	1	3	3	neg	3	2	1	IDC	-	-	+	Her2	5	1	c.772G>A	p.E258K
MB-1036	1	5	1	1	3	neg	NA	0	1	IDC	-	-	-	Basal	10	1	c.707A>C	p.Y236S
MB-1037	1	4	0	1	1	pos	3	0	0	IDC-TUB	+	+	-	LumA	8	0		
MB-1038	1	4	1	2	3	neg	1	0	0	IDC	-	-	-	Basal	10	0		
MB-1039	1	4	1	3	2	pos	1	0	0	IDC+ILC	-	-	-	Basal	4	0		
MB-1040	1	5	1	2	2	pos	1	0	0	IDC	+	-	-	LumA	4	0		
MB-1041	1	4	0	2	NA	pos	NA	0	0	ILC	+	+	-	LumA	3	0		
MB-1042	1	4	1	2	1	pos	1	0	0	IDC	+	+	-	LumB	8	0		
MB-1043	1	4	0	1	3	pos	1	0	0	IDC	+	+	-	Basal	4	0		
MB-1044	1	4	0	1	2	pos	NA	0	0	IDC	+	+	-	Normal	4	0		
MB-1045	1	4	0	2	2	neg	1	0	1	IDC	-	-	-	Her2	4	1	c.916C>T	p.R306X
MB-1046	1	5	0	2	2	pos	NA	2	0	IDC	+	-	+	Her2	6	0		
MB-1048	1	4	0	1	2	pos	NA	0	0	IDC	+	-	-	Her2	1	0		
MB-1049	1	4	0	1	3	neg	NA	0	0	IDC	-	-	-	Basal	4	0		
MB-1050	1	3	1	2	2	pos	NA	2	1	IDC	+	+	+	Her2	5	1	c.499C>T	p.Q167X
MB-1051	1	2	0	2	2	pos	NA	0	1	IDC	+	-	-	Her2	6	1	c.832C>T	p.P278S
MB-1052	1	4	0	1	3	neg	NA	0	1	IDC	-	-	-	Basal	9	1	c.529_546del18	p.?
MB-1053	1	3	0	1	3	pos	NA	0	1	IDC	+	+	-	LumA	4	1	c.916C>G	p.R306X
MB-1054	1	3	1	1	3	pos	NA	0	1	IDC	-	+	-	Basal	10	1	c.584T>C	p.I195T
MB-1055	1	4	0	2	3	neg	NA	0	1	IDC	-	-	-	Basal	10	1	c.916C>T	p.R306X
MB-1056	1	2	1	2	2	pos	NA	0	0	IDC	+	+	-	LumA	3	0		
MB-1057	1	3	1	2	2	neg	NA	0	1	ILC	-	-	-	Normal	4	1	c.722C>T	p.S241F
MB-1058	1	2	1	2	NA	pos	NA	0	0	IDC+ILC	+	+	-	Normal	3	0		
MB-1059	1	5	1	2	3	neg	NA	2	1	IDC	-	-	+	Her2	5	1	c.892G>T	p.E298X
MB-1060	1	3	1	3	1	pos	NA	-1	0	ILC	+	-	-	LumA	3	0		
MB-1061	1	4	0	2	2	pos	NA	0	0	IDC	+	-	-	Normal	4	0		
MB-1062	1	3	1	1	1	pos	NA	0	0	IDC	+	+	-	LumA	3	0		
MB-1063	1	4	0	2	3	pos	NA	0	0	IDC	+	+	-	LumA	3	0		
MB-1065	1	2	0	1	2	pos	NA	0	0	IDC	+	+	-	LumB	9	0		
MB-1066	1	4	1	1	3	neg	NA	1	0	IDC	-	-	-	Her2	3	0		
MB-1067	1	2	0	1	2	neg	NA	2	1	IDC	-	-	+	Her2	5	1	c.581T>G	p.L194R
MB-1068	1	2	0	2	3	pos	NA	2	1	IDC	+	-	+	Her2	9	1	c.817C>T	p.R273C
MB-1069	1	3	0	2	3	pos	NA	2	0	IDC	-	-	+	Her2	5	0		
MB-1070	1	3	1	1	3	pos	NA	2	1	IDC	+	+	-	Her2	9	1	c.713_718del6	p.?
MB-1071	1	4	0	1	3	pos	NA	0	0	IDC	+	+	-	Her2	3	0		
MB-1072	1	4	1	3	2	pos	NA	1	1	IDC	+	+	-	LumB	1	1	c.1045G>T	p.E349X
MB-1073	1	3	1	2	3	neg	NA	2	1	IDC	-	-	+	Basal	5	1	c.903_904ins1	p.?
MB-1074	1	2	0	1	1	pos	NA	0	0	IDC	+	-	-	Normal	4	0		
MB-1075	1	5	0	1	2	pos	NA	1	0	IDC	+	-	-	LumB	1	0		
MB-1076	1	3	0	2	2	pos	NA	0	0	IDC	+	+	-	LumA	7	0		
MB-1077	1	3	1	1	2	pos	NA	0	NA	IDC	+	+	-	LumA	3	NA	NA	NA
MB-1078	1	4	0	3	3	neg	NA	0	0	IDC	-	-	-	Basal	10	0		
MB-1079	1	4	1	2	2	pos	NA	0	0	IDC	+	-	-	Normal	4	0		
MB-1080	1	4	0	1	1	pos	NA	0	0	IDC	+	+	-	LumA	8	0		
MB-1081	1	3	0	2	3	neg	NA	0	0	IDC	-	-	-	Basal	10	0		
MB-1082	1	2	1	2	3	neg	NA	2	1	IDC	-	-	+	Her2	5	1	c.405C>G	p.C135W
MB-1084	1	2	0	3	3	neg	NA	2	1	IDC	-	-	+	Basal	10	1	c.148_149del2	p.?
MB-1085	1	2	1	2	2	pos	NA	0	0	ILC	+	+	-	Normal	7	0		
MB-1086	1	3	1	1	2	pos	NA	0	0	IDC	+	+	-	LumB	9	0		
MB-1087	1	3	1	1	3	neg	NA	0	1	IDC	-	-	-	Basal	10	1	c.783-2A>C	NA
MB-1088	1	4	1	2	3	pos	NA	2	1	IDC	+	-	+	Her2	5	1	c.736A>G	p.M246V
MB-1089	1	3	1	1	3	neg	NA	1	1	IDC	-	-	-	Basal	10	1	c.451C>G	p.P151A
MB-1090	1	2	1	1	3	neg	NA	0	1	IDC	-	-	-	Basal	10	1	c.880G>T	p.E294X
MB-1091	1	1	1	1	1	pos	NA	-1	1	IDC	+	+	-	LumA	7	1	c.707A>G	p.Y236C
MB-1092	1	2	0	2	1	neg	NA	0	0	IDC	+	+	-	Her2	1	0		
MB-1093	1	2	1	1	1	pos	NA	0	0	IDC	+	+	-	LumA	3	0		
MB-1094	1	1	0	2	3	pos	NA	0	0	IDC	+	+	-	LumB	9	0		
MB-1095	1	1	0	2	1	pos	NA	0	0	IDC	+	-	-	LumA	8	0		
MB-1096	1	4	1	1	2	pos	NA	0	0	IDC	+	+	-	LumA	3	0		
MB-1097	1	4	1	2	NA	pos	NA	0	0	ILC	+	-	-	LumB	8	0		
MB-1099	1	3	1	2	2	pos	NA	0	0	IDC	+	+	-	LumB	9	0		
MB-1100	1	4	1	2	3	pos	NA	0	0	IDC	+	+	-	LumB	8	0		
MB-1101	1	5	0	1	2	pos	NA	0	0	IDC	+	+	-	LumA	3	0		
MB-1102	1	5	1	2	1	pos	1	0	0	IDC-MUC	+	+	-	LumB	3	0		
MB-1104	1	3	1	1	3	pos	NA	0	0	IDC	+	-	-	Basal	6	0		
MB-1106	1	4	1	2	1	pos	NA	0	0	IDC	+	+	-	LumA	2	0		
MB-1107	1	5	1	2	3	pos	1	0	0	IDC	+	-	-	LumA	8	0		
MB-1109	1	4	1	1	3	pos	NA	1	NA	IDC+ILC	+	+	-	LumB	2	NA	NA	NA
MB-1111	1	3	1	3	3	pos	NA	0	1	IDC	+	+	-	Basal	1	1	c.818G>A	p.R273H
MB-1112	1	4	0	2	3	pos	NA	0	0	IDC	+	+	-	LumB	2	0		
MB-1113	1	4	0	1	1	pos	NA	0	0	IDC	+	+	-	LumA	7	0		
MB-1114	1	3	0	1	3	neg	NA	0	1	IDC	-	-	-	Basal	10	1	c.723del1	p.?
MB-1115	1	3	0	3	3	NA	3	2	0	IDC	+	-	+	Her2	5	0		
MB-1116	1	4	1	2	2	pos	NA	0	0	ILC	+	-	-	LumB	8	0		
MB-1118	1	5	0	2	2	NA	1	0	0	IDC	+	+	-	LumA	8	0		
MB-1119	1	3	1	2	3	neg	NA	0	1	IDC	-	-	-	Basal	10	1	c.323_324ins1	p.?
MB-1121	1	3	1	2	3	neg	NA	0	0	IDC	-	-	-	Basal	10	0		
MB-1122	1	3	1	2	3	pos	NA	0	0	IDC	+	-	-	Basal	4	0		
MB-1123	1	4	0	2	1	NA	1	2	0	IDC-MUC	+	+	-	LumA	1	0		
MB-1124	1	3	1	2	2	NA	1	0	0	IDC	+	+	-	LumA	7	0		
MB-1127	1	5	0	1	NA	pos	NA	0	0	IDC-MUC	+	+	-	Normal	9	0		
MB-1128	1	4	0	2	2	pos	NA	2	0	IDC	+	+	+	LumA	5	0		
MB-1130	1	3	1	2	3	pos	NA	1	0	IDC	+	-	-	LumB	9	0		
MB-1131	1	3	1	2	3	pos	NA	0	0	IDC	+	-	-	LumB	8	0		
MB-1132	1	4	0	1	2	pos	NA	0	0	IDC	+	+	-	LumB	7	0		
MB-1133	1	4	1	1	3	pos	NA	0	0	IDC	+	-	-	LumB	1	0		
MB-1135	1	5	1	2	3	neg	NA	2	0	IDC	-	-	+	Her2	5	0		
MB-1137	1	5	0	2	NA	pos	NA	0	0	IDC-MUC	+	-	-	LumA	4	0		
MB-1138	1	2	0	2	3	pos	NA	0	0	IDC	+	+	-	LumB	8	0		
MB-1140	1	2	1	3	3	pos	NA	0	0	IDC	+	-	-	LumB	8	0		
MB-1141	1	2	0	1	2	NA	1	0	0	IDC-MUC	+	+	-	LumA	7	0		
MB-1142	1	4	0	1	2	NA	1	0	0	IDC	+	+	-	LumB	2	0		
MB-1143	1	5	0	2	3	neg	NA	2	0	IDC	-	-	+	Her2	5	0		
MB-1144	1	4	0	2	3	pos	NA	0	1	IDC	+	+	-	LumB	8	1	c.844C>T	p.R282W
MB-1145	1	5	1	2	3	neg	NA	0	1	IDC	-	-	-	Basal	10	1	c.151G>A	p.E51X
MB-1147	1	4	0	2	3	pos	NA	0	0	IDC	+	+	-	LumB	6	0		
MB-1148	1	4	1	3	3	pos	NA	0	0	ILC	+	+	-	LumA	8	0		
MB-1149	1	4	1	3	3	pos	NA	0	0	IDC	+	-	-	LumB	9	0		
MB-1150	1	4	0	2	3	pos	NA	0	1	IDC	+	-	-	LumB	3	1	c.524G>A	p.R175H
MB-1151	1	4	0	2	3	pos	NA	0	1	IDC	-	-	-	Basal	10	1	c.586C>T	p.R196X
MB-1152	1	5	0	1	3	pos	NA	1	0	IDC	+	-	-	Her2	9	0		
MB-1153	1	5	0	2	2	pos	NA	0	0	ILC	+	-	-	Her2	8	0		
MB-1154	1	4	0	2	3	neg	NA	0	1	IDC	-	-	-	Basal	10	1	c.818G>A	p.R273H
MB-1155	1	4	1	1	3	neg	NA	0	1	IDC	-	-	-	Basal	10	1	c.723del1	p.?
MB-1157	1	4	1	1	2	pos	1	0	0	IDC	+	+	-	LumB	8	0		
MB-1158	1	3	1	2	3	neg	NA	0	0	IDC	-	-	-	Basal	4	0		
MB-1159	1	2	0	2	3	neg	NA	0	1	IDC	-	-	-	Basal	10	1	c.305_306del2	p.?
MB-1160	1	4	0	2	3	pos	NA	1	1	IDC	+	+	-	Her2	9	1	c.535C>G	p.H179D

MB-1161	1	3	1	2	2	pos	NA	1	0	IDC	+	+	-	LumA	6	0		
MB-1162	1	4	0	1	1	pos	NA	0	0	IDC	+	+	-	LumA	3	0		
MB-1163	1	3	1	2	2	neg	NA	0	0	ILC	+	+	-	LumA	3	0		
MB-1164	1	4	1	1	1	pos	NA	0	1	IDC	+	+	-	LumA	3	1	c.467G>A	p.R156H
MB-1165	1	4	1	3	3	neg	NA	0	1	IDC	-	-	-	Basal	10	1	c.524G>A	p.R175H
MB-1167	1	3	0	3	2	pos	NA	0	0	IDC+ILC	+	+	-	Her2	8	0		
MB-1168	1	4	0	2	3	pos	NA	0	0	IDC	+	+	-	LumA	7	0		
MB-1170	1	2	0	2	2	pos	NA	0	0	IDC	+	-	-	LumA	4	0		
MB-1171	1	3	1	2	3	pos	NA	0	0	IDC	+	+	-	LumB	9	0		
MB-1172	1	4	1	1	2	pos	NA	0	0	ILC	+	+	-	LumB	8	0		
MB-1173	1	3	0	2	2	pos	1	0	0	ILC	+	-	-	LumA	8	0		
MB-1174	1	3	0	2	3	NA	1	0	1	IDC	+	-	-	LumB	6	1	c.659A>G	p.Y220C
MB-1176	1	4	1	2	2	pos	NA	0	0	IDC	+	+	-	LumA	8	0		
MB-1181	1	3	1	2	3	pos	NA	1	0	IDC	+	-	-	LumB	1	0		
MB-1182	1	4	0	2	3	pos	NA	0	0	IDC	+	-	-	Her2	9	0		
MB-1185	1	4	1	1	2	pos	NA	0	0	IDC	+	+	-	LumA	8	0		
MB-1186	1	5	1	2	2	pos	1	0	0	IDC	+	+	-	LumB	7	0		
MB-1187	1	4	0	1	2	neg	NA	2	NA	IDC	-	-	+	Normal	5	NA	NA	NA
MB-1188	1	3	0	2	NA	pos	1	0	0	OTHER INVASIVE	+	+	-	LumB	7	0		
MB-1193	1	2	0	1	3	pos	NA	1	0	IDC	+	+	-	LumB	6	0		
MB-1194	1	4	0	2	2	pos	NA	0	0	IDC-MUC	+	-	-	LumB	2	0		
MB-1195	1	4	0	2	2	pos	1	0	0	IDC	+	+	-	LumA	8	0		
MB-1196	1	3	1	3	3	pos	NA	0	1	IDC	+	+	-	Her2	9	1	c.672+1G>C	NA
MB-1197	1	3	1	2	2	pos	NA	0	0	IDC	+	+	-	LumB	8	0		
MB-1198	1	5	0	2	3	pos	NA	0	1	IDC	+	+	-	Basal	6	1	c.871A>T	p.K291X
MB-1199	1	4	1	2	2	pos	1	0	0	IDC	+	-	-	LumB	8	0		
MB-1200	1	3	0	2	3	pos	1	0	0	IDC	+	+	-	LumB	8	0		
MB-1201	1	4	0	1	2	pos	NA	0	0	IDC+ILC	-	+	-	Basal	3	0		
MB-1205	1	4	1	2	3	neg	1	0	1	IDC	-	-	-	Basal	10	1	c.818G>A	p.R273H
MB-1207	1	3	1	2	NA	neg	NA	2	1	IDC	-	-	+	Her2	1	1	c.749C>T	p.P250L
MB-1208	1	3	1	2	3	neg	NA	0	0	IDC	-	-	-	Basal	10	0		
MB-1212	1	4	0	1	3	pos	NA	1	0	ILC	+	+	-	LumA	3	0		
MB-1215	1	5	1	1	2	pos	1	0	0	IDC+ILC	+	-	-	LumA	4	0		
MB-1216	1	4	0	2	2	pos	NA	0	0	IDC-MUC	+	+	-	LumB	4	0		
MB-1217	1	4	0	1	NA	pos	NA	0	0	ILC	+	+	-	LumA	3	0		
MB-1218	1	2	1	1	1	pos	NA	0	0	IDC	+	+	-	LumA	3	0		
MB-1219	1	3	1	1	1	pos	NA	0	0	IDC	+	+	-	LumA	8	0		
MB-1220	1	5	0	2	2	pos	NA	0	0	ILC	+	+	-	LumB	8	0		
MB-1225	1	5	1	2	3	neg	NA	1	1	IDC	-	-	-	Basal	10	1	c.833C>G	p.P278R
MB-1226	1	2	1	1	3	pos	NA	1	0	IDC	+	-	-	LumB	6	0		
MB-1227	1	4	0	1	2	pos	NA	0	0	IDC	+	-	-	Normal	3	0		
MB-1228	1	2	1	2	2	pos	1	2	0	IDC	+	+	-	Normal	7	0		
MB-1229	1	3	1	2	3	pos	NA	0	0	IDC	+	+	-	LumA	8	0		
MB-1230	1	5	0	1	2	pos	NA	0	0	IDC	+	+	-	LumA	3	0		
MB-1231	1	4	0	2	NA	pos	3	0	0	IDC-MUC	+	+	-	LumA	4	0		
MB-1232	1	1	0	1	2	pos	NA	0	0	IDC	+	+	-	LumA	8	0		
MB-1233	1	5	0	2	2	pos	1	0	0	IDC	+	+	-	Her2	8	0		
MB-1234	1	4	0	1	3	pos	NA	0	0	IDC	+	-	-	LumB	6	0		
MB-1235	1	4	1	2	2	pos	1	1	0	IDC	+	+	-	LumA	8	0		
MB-1236	1	3	1	2	2	pos	NA	1	0	IDC	+	+	-	LumB	8	0		
MB-1237	1	3	1	1	2	pos	1	1	0	IDC	+	+	-	NC	9	0		
MB-1238	1	3	0	2	3	pos	NA	0	1	IDC	+	-	-	Her2	8	1	c.672+1G>T	NA
MB-1241	1	3	1	2	2	pos	1	0	0	IDC	+	+	-	LumB	7	0		
MB-1243	1	5	0	1	1	pos	NA	0	0	IDC	+	-	-	LumA	8	0		
MB-1244	1	5	1	1	1	pos	1	0	0	IDC	+	+	-	LumA	8	0		
MB-1249	1	3	0	2	NA	pos	NA	1	0	IDC-MUC	+	+	-	LumA	8	0		
MB-1250	1	5	0	1	3	neg	3	2	0	IDC	-	-	+	Her2	5	0		
MB-1251	1	4	1	1	3	pos	NA	2	0	IDC	-	-	+	Her2	5	0		
MB-1252	1	4	1	2	3	pos	1	1	0	IDC	-	-	-	Her2	4	0		
MB-1253	1	3	0	2	2	pos	1	0	0	IDC-MUC	+	-	-	LumB	3	0		
MB-1254	1	5	1	2	2	pos	1	0	1	IDC	+	+	-	LumA	8	1	c.307T>C	p.Y103H
MB-1256	1	3	1	2	2	neg	3	2	1	IDC	-	-	+	Her2	5	1	c.401T>G	p.F134C
MB-1258	1	4	1	2	3	neg	1	0	1	IDC	-	-	-	Basal	10	1	c.581_585del5	p.?
MB-1260	1	2	1	2	3	neg	NA	2	1	IDC	+	-	+	Her2	5	1	c.973G>T	p.G325X
MB-1262	1	4	1	2	3	pos	NA	0	1	IDC	+	-	-	Her2	9	1	c.785G>T	p.G262C
MB-1263	1	5	1	2	2	pos	NA	1	1	IDC	+	+	-	LumB	9	1	c.1054G>T	p.D352Y
MB-1264	1	5	1	2	3	pos	NA	0	0	IDC	+	-	-	Normal	4	0		
MB-1266	1	4	1	2	1	pos	1	0	0	IDC	+	+	-	LumB	9	0		
MB-1267	1	3	1	2	3	neg	NA	2	1	IDC	-	-	-	Basal	10	1	c.685_686del2	p.?
MB-1268	1	5	1	3	3	pos	1	-1	0	ILC	+	+	-	LumA	8	0		
MB-1269	1	3	1	2	3	neg	NA	2	0	IDC	-	-	-	Basal	10	0		
MB-1270	1	3	1	1	3	neg	NA	0	1	ILC	-	-	-	Basal	10	1	c.393_395del3	p.?
MB-1273	1	5	1	2	3	neg	3	2	1	IDC	-	-	+	Her2	5	1	c.772G>C	p.E258Q
MB-1275	1	3	1	2	3	neg	3	2	1	IDC	-	+	+	Her2	5	1	c.512A>G	p.E171G
MB-1276	1	5	1	2	1	pos	1	0	0	IDC	+	+	-	LumA	8	0		
MB-1277	1	3	1	2	2	pos	3	0	0	IDC	+	+	-	LumA	8	0		
MB-1278	1	4	1	1	2	pos	1	0	0	IDC	+	+	-	LumA	3	0		
MB-1279	1	3	1	3	2	pos	NA	1	1	IDC	+	+	-	Her2	9	1	c.989T>G	p.L330R
MB-1280	1	5	1	2	2	pos	1	0	0	IDC-MUC	+	+	-	LumA	7	0		
MB-1281	1	NA	1	1	3	pos	NA	1	0	IDC	-	-	+	Her2	5	0		
MB-1283	1	2	1	2	2	pos	1	-1	1	ILC	+	-	-	LumB	6	1	c.1043_1045TGG>CTT	p.????X
MB-1284	1	4	1	1	3	pos	NA	0	0	ILC	+	-	-	LumA	6	0		
MB-1285	1	4	1	2	2	pos	1	1	1	OTHER INVASIVE	+	+	-	LumB	6	1	c.631A>G	p.T211A
MB-1286	1	4	1	2	3	pos	1	0	0	IDC	+	-	-	LumB	3	0		
MB-1287	1	4	1	2	3	pos	NA	2	1	IDC	+	+	+	LumB	5	1	c.818G>A	p.R273H
MB-1288	1	4	1	2	2	pos	NA	0	0	IDC	+	-	-	LumB	8	0		
MB-1289	1	4	1	3	2	pos	NA	1	0	ILC	+	+	-	Normal	4	0		
MB-1291	1	5	1	3	3	pos	NA	2	1	IDC+ILC	-	-	+	Her2	1	1	c.74+2T>C	NA
MB-1292	1	4	1	1	2	pos	NA	1	0	IDC	+	+	-	LumB	6	0		
MB-1293	1	3	1	2	3	pos	1	0	1	IDC	+	+	-	LumA	3	1	c.764T>G	p.I255S
MB-1294	1	3	1	1	2	pos	NA	1	1	IDC	+	+	-	LumB	1	1	c.993+2T>C	NA
MB-1295	1	5	1	2	3	pos	3	0	0	IDC	+	+	-	LumA	3	0		
MB-1296	1	3	1	2	3	pos	NA	2	1	IDC	+	-	+	LumB	5	1	c.711G>T	p.M237I
MB-1297	1	4	1	2	3	pos	NA	0	0	IDC	+	+	-	LumB	1	0		
MB-1298	1	3	1	2	2	pos	1	0	0	IDC	+	+	-	LumB	1	0		
MB-1299	1	3	1	2	3	pos	NA	0	0	IDC	+	-	-	LumB	10	0		
MB-2513	1	4	0	2	2	neg	3	2	1	IDC	-	-	+	LumA	5	1	c.1031T>G	p.L344R
MB-2517	1	3	0	1	3	neg	3	2	0	IDC	-	-	+	Her2	5	0		
MB-2536	1	4	0	1	1	pos	NA	1	0	IDC-TUB	+	-	-	LumA	3	0		
MB-2556	1	5	1	2	2	pos	1	0	0	ILC	-	-	-	Normal	4	0		
MB-2564	1	3	0	1	1	pos	1	0	0	IDC-TUB	+	+	-	LumA	8	0		
MB-2610	1	5	1	2	1	pos	1	0	0	IDC-TUB	+	+	-	LumA	8	0		
MB-2613	1	5	0	2	3	pos	1	0	0	IDC	+	+	-	LumB	1	0		
MB-2614	1	3	1	1	1	neg	NA	0	0	IDC-TUB	+	+	-	LumA	7	0		
MB-2616	1	4	0	1	2	pos	1	0	0	IDC+ILC	+	+	-	Normal	4	0		
MB-2617	1	5	0	1	3	pos	1	0	1	IDC	+	-	-	LumB	4	1	c.536A>G	p.H179R
MB-2618	1	4	0	1	2	pos	1	1	0	IDC	+	+	-	LumB	1	0		
MB-2624	1	2	1	1	1	pos	1	0	0	IDC-TUB	+	+	-	LumA	3	0		
MB-2626	1	3	0	1	3	pos	3	2	0	IDC	+	+	+	LumB	5	0		
MB-2629	1	3	1	1	3	pos	1	0	0	IDC	+	-	-	LumB	3	0		
MB-2632	1	4	1	1	3	neg	3	2	1	IDC	-	-	+	Her2	5	1	c.596G>T	p.G199V
MB-2634	1	5	0	1	3	pos	1	1	0	IDC	+	+	-	LumB	2	0		
MB-2642	1	5	1	2	3	neg	1	-1	0	IDC	+	+	-	LumB	1	0		
MB-2643	1	5	0	1	2	NA	NA	0	0	IDC	-	-	-	Basal	4	0		
MB-2645	1	5	1	2	3	pos	1	0	1	IDC	+	-	-	Basal	4	1	c.524G>A	p.R175H
MB-2669	1	2	0	2	2	pos	1	0	0	IDC-TUB	+	-	-	LumA	6	0		
MB-2686	1	5	1	1	3	pos	1	0	0	IDC	+	-	-	LumB	1	0		
MB-2705	1	5	0	1	2	pos	1	0	0	IDC-TUB	+	-	-	LumB	3	0		
MB-2708	1	3	0	1	2	pos	2	0	1	IDC-TUB	+	+	-	LumB	7	1	c.464C>A	p.T155N
MB-2711	1	4	0	1	2	pos	1	0	0	IDC	+	+	-	LumA	7	0		
MB-2712	1	3	1	1	1	pos	1	0	0	IDC+ILC	+	+	-	LumA	7	0		
MB-2718	1	4	1	2	2	neg	1	0	1	IDC-TUB	-	-	-	Basal	10	1	c.1024C>T	p.R342X
MB-2721	1	4	0	1	1	neg	1	-1	0	IDC-TUB	+	+	-	LumA	7	0		
MB-2724	1	5	0	1	3	neg	3	0	0	IDC	-	-	-	Basal	4	0		
MB-2725	1	5	1	1	1	pos	1	0	0	IDC	+	+	-	LumA	4	0		
MB-2728	1	5	1	2	3	neg	1	0	0	IDC	+	+	-	LumB	9	0		
MB-2730	1	4	1	2	3	pos	1	0	0	IDC	+	-	-	LumB	1	0		
MB-2735	1	3	1	1	3	neg	2	2	0	IDC	+	-	-	LumB	6	0		
MB-2742	1	3	1	1	3	neg	1	2	1	IDC	-	-	+	Her2	5	1	c.332T>C	p.L111P
MB-2744	1	2	0	2	2	pos	1	0	0	ILC	+	+	-	Normal	4	0		
MB-2745	1	2	1	2	2	pos	2	0	0	IDC+ILC	+	+	-	LumA	8	0		
MB-2747	1	4	0	1	1	pos	1	0	0	IDC-TUB	+	+	-	LumA	8	0		
MB-2749	1	5	0	1	1	pos	1	0	0	IDC-TUB	+	+	-	LumA	3	0		
MB-2750	1	5	0	2	1	pos	1	1	0	IDC+ILC	+	-	-	LumA	8	0		
MB-2752	1	3	0	2	2	pos	1	0	0	IDC+ILC	+	+	-	LumA	7	0		
MB-2753	1	4	1	2	3	neg	1	1	1	IDC	-	-	-	Basal	10	1	c.417_418del2	p.?
MB-2754	1	5	0	2	2	neg	1	0	0	ILC	-	-	-	Normal	3	0		
MB-2758	1	5	1	2	3	neg	2	2	0	IDC	-	-	+	Basal	4	0		
MB-2760	1	3	1	1	2	pos	NA	0	0	IDC	+	+	-	LumB	7	0		
MB-2763	1	5	0	2	2	pos	1	0	0	MIXED NST AND A SPECIAL TYPE	+	+	-	LumB	4	0		
MB-2764	1	4	1	1	2	pos	1	0	0	IDC	+	-	-	Normal	4	0		
MB-2765	1	3	0	1	3	pos	1	1	0	IDC	+	+	-	LumB	3	0		
MB-2767	1	5	1	2	3	pos	1	0	0	IDC	+	+	-	LumB	2	0		
MB-2769	1	4	0	1	2	pos	1	0	0	ILC	+	+	-	LumB	7	0		

MB-2770	1	3	1	2	2	pos	1	0	0	ILC	+	+	-	LumA	8	0	c.903A>G	p.P301P
MB-2771	1	5	0	1	3	pos	1	-1	1	IDC	-	-	-	Basal	10	1	c.329_330ins7	p.?
MB-2772	1	4	0	1	3	pos	1	-1	0	IDC	+	+	-	LumB	6	0		
MB-2774	1	5	1	2	2	pos	1	-2	0	IDC-TUB	+	+	-	LumB	7	0		
MB-2778	1	4	1	1	2	pos	1	0	0	IDC-TUB	+	+	-	NC	3	0		
MB-2779	1	3	0	2	2	pos	1	0	0	ILC	+	+	-	LumA	10	0		
MB-2781	1	4	1	2	2	pos	1	1	0	ILC	+	+	-	LumB	1	0		
MB-2786	1	4	1	2	3	pos	3	2	0	IDC	+	-	+	LumA	5	0		
MB-2790	1	3	0	2	2	pos	1	0	0	IDC-TUB	+	+	-	LumA	8	0		
MB-2791	1	3	0	1	2	pos	NA	0	0	IDC	+	+	-	LumA	3	0		
MB-2792	1	5	1	2	2	pos	2	1	0	IDC	+	+	-	Her2	9	0		
MB-2793	1	3	0	2	2	pos	1	0	0	IDC	+	+	-	LumB	8	0		
MB-2795	1	4	0	1	2	pos	1	0	0	IDC	+	-	-	LumB	1	0		
MB-2796	1	3	1	2	3	pos	1	0	1	IDC+ILC	+	+	-	LumB	2	1	c.659A>C	p.Y220S
MB-2797	1	4	0	1	1	pos	1	0	0	IDC-TUB	+	+	-	LumA	6	0		
MB-2801	1	3	1	1	2	pos	1	0	1	IDC-TUB	+	+	-	LumA	2	1	c.637C>T	p.R213X
MB-2803	1	3	0	1	2	pos	1	0	0	IDC	+	-	-	LumB	8	0		
MB-2814	1	3	0	1	2	pos	1	1	0	IDC	+	+	-	LumB	3	0		
MB-2815	1	5	0	2	1	pos	NA	0	0	IDC-TUB	+	+	-	Normal	3	0		
MB-2819	1	3	0	1	1	pos	1	0	0	IDC-TUB	+	+	-	LumA	7	0		
MB-2820	1	4	0	1	3	pos	1	0	NA	IDC	+	+	-	LumA	3	NA	NA	NA
MB-2821	1	4	0	1	NA	neg	1	0	0	IDC-MED	-	+	-	Basal	4	0		
MB-2823	1	4	0	1	3	pos	3	2	0	IDC	+	+	+	Normal	4	0		
MB-2827	1	3	1	1	3	neg	1	0	1	IDC	-	-	-	Basal	10	1	c.672+2T>G	NA
MB-2833	1	4	1	2	3	neg	1	0	0	IDC	-	-	-	Basal	10	0		
MB-2834	1	4	1	1	2	pos	1	0	1	IDC	-	-	-	Her2	3	1	c.695T>C	p.I232T
MB-2835	1	2	0	2	3	pos	1	0	0	IDC	+	+	-	LumA	3	0		
MB-2838	1	4	0	2	2	pos	1	-1	0	IDC+ILC	+	+	-	LumA	8	0		
MB-2840	1	3	0	1	2	pos	1	0	0	ILC	+	-	-	LumA	3	0		
MB-2842	1	4	1	2	3	neg	1	0	1	IDC	-	-	-	Basal	9	1	c.581T>G	p.L194R
MB-2843	1	2	0	1	2	pos	1	0	0	IDC	+	-	-	LumA	2	0		
MB-2844	1	4	0	1	3	pos	2	1	1	IDC	+	-	-	LumB	9	1	c.486_487del1ins7	p.?
MB-2845	1	5	0	1	2	pos	1	0	0	ILC	+	-	-	LumA	4	0		
MB-2846	1	3	0	1	2	neg	NA	0	1	IDC	+	-	-	Basal	4	1	c.700T>A	p.Y234N
MB-2847	1	4	1	2	3	pos	2	2	1	IDC	-	-	+	Her2	5	1	c.673-1 G>C	NA
MB-2848	1	3	1	1	2	pos	1	0	0	IDC-TUB	+	+	-	LumA	3	0		
MB-2849	1	5	0	2	3	neg	1	0	1	IDC	-	-	-	Basal	9	1	c.463_470del8	p.?
MB-2850	1	5	1	2	3	neg	1	0	0	IDC	-	-	-	Basal	10	0		
MB-2851	1	4	0	1	3	pos	1	0	1	IDC	+	+	-	LumA	4	1	c.880G>T	p.E294X
MB-2853	1	5	0	2	2	pos	1	0	0	IDC-MUC	+	-	-	LumB	6	0		
MB-2854	1	2	0	2	2	pos	1	0	0	ILC	+	-	-	LumA	7	0		
MB-2857	1	5	1	2	3	neg	1	0	0	IDC	-	-	-	Basal	10	0		
MB-2858	1	5	0	2	3	pos	1	0	1	MIXED NST AND A SPECIAL TYPE	+	+	-	LumB	1	1	c.800G>C	p.R267P
MB-2863	1	5	1	2	1	pos	1	0	0	MIXED NST AND A SPECIAL TYPE	+	-	-	LumA	4	0		
MB-2867	1	3	1	2	1	pos	1	0	0	IDC-TUB	+	+	-	LumA	4	0		
MB-2895	1	5	0	1	3	neg	2	2	0	IDC	-	-	+	Basal	5	0		
MB-2896	1	5	0	1	2	pos	1	0	0	IDC-TUB	+	-	-	Normal	4	0		
MB-2900	1	4	1	2	2	pos	1	0	0	ILC	+	+	-	Normal	4	0		
MB-2901	1	2	0	2	2	pos	1	0	0	IDC	+	+	-	LumB	8	0		
MB-2904	1	5	1	2	3	neg	1	0	1	IDC	-	-	-	Basal	10	1	c.329del1	p.?
MB-2912	1	3	1	2	3	neg	1	0	1	IDC	-	-	-	Basal	10	1	c.467_468ins7	p.?
MB-2916	1	5	0	1	1	pos	1	0	0	IDC-TUB	+	+	-	LumA	3	0		
MB-2917	1	5	1	2	3	neg	NA	0	1	IDC	-	-	-	Basal	10	1	c.376-2A>G	NA
MB-2919	1	4	0	2	1	pos	1	0	0	IDC-MUC	+	+	-	LumB	8	0		
MB-2922	1	5	1	2	3	neg	1	1	1	IDC	-	-	-	Her2	1	1	c.455del1	p.?
MB-2923	1	5	1	2	3	neg	3	2	0	IDC	-	-	+	Her2	5	0		
MB-2927	1	4	0	1	1	pos	1	-1	0	IDC-TUB	+	+	-	LumA	1	0		
MB-2929	1	4	1	2	2	neg	1	0	1	IDC	-	-	-	Her2	4	1	c.541C>T	p.R181C
MB-2931	1	5	0	1	2	pos	1	0	1	IDC	+	+	-	LumB	8	1	c.532_533ins1	p.?
MB-2932	1	4	0	1	3	pos	1	0	0	IDC	+	+	-	LumB	2	0		
MB-2933	1	2	0	1	1	pos	1	0	0	IDC-TUB	+	+	-	LumA	3	0		
MB-2939	1	5	1	1	3	pos	3	2	0	IDC	+	+	+	LumB	5	0		
MB-2944	1	5	0	2	3	neg	1	0	1	IDC	+	+	-	LumA	8	1	c.380C>T	p.S127F
MB-2947	1	5	0	1	2	pos	1	0	0	IDC	+	+	-	LumA	4	0		
MB-2951	1	3	0	1	2	pos	1	0	0	IDC	+	+	-	LumA	1	0		
MB-2952	1	3	0	1	1	pos	1	0	0	IDC-TUB	+	+	-	LumA	8	0		
MB-2953	1	3	0	1	2	pos	1	0	1	IDC	+	+	-	LumB	10	1	c.701A>G	p.Y234C
MB-2954	1	5	1	2	2	pos	1	0	0	IDC	+	+	-	LumB	8	0		
MB-2957	1	5	1	2	3	neg	1	0	0	IDC	-	-	-	Basal	10	0		
MB-2960	1	4	0	2	3	pos	1	0	0	IDC	+	-	-	LumA	6	0		
MB-2963	1	3	1	1	2	pos	1	0	0	IDC-TUB	-	-	-	Her2	4	0		
MB-2964	1	5	1	2	3	pos	NA	2	1	IDC	+	-	-	Her2	5	1	c.742C>T	p.R248W
MB-2966	1	3	1	1	3	pos	1	0	0	IDC	+	+	-	LumA	8	0		
MB-2969	1	3	0	1	2	pos	1	-2	0	IDC	+	+	-	LumA	3	0		
MB-2970	1	4	1	2	2	pos	1	0	1	IDC+ILC	+	+	-	LumA	4	1	c.370_371ins1	p.?
MB-2971	1	1	1	1	2	pos	1	0	0	IDC	+	+	-	LumA	3	0		
MB-2977	1	2	0	1	1	pos	1	0	0	IDC-TUB	+	+	-	LumA	7	0		
MB-2983	1	4	1	1	2	pos	NA	2	1	IDC-TUB	+	+	+	LumB	5	1	c.321C>G	p.Y107X
MB-2984	1	4	0	2	3	pos	NA	2	1	IDC	+	-	+	LumB	9	1	c.716A>G	p.N239S
MB-2993	1	5	0	2	3	neg	1	0	0	IDC	-	-	-	Basal	10	0		
MB-2994	1	4	1	1	3	pos	2	2	0	IDC	+	+	+	LumA	5	0		
MB-2996	1	4	0	1	3	pos	1	0	0	IDC	+	+	-	LumA	7	0		
MB-2999	1	4	0	1	3	pos	1	0	0	IDC	+	-	-	LumA	7	0		
MB-3001	1	5	1	1	3	neg	1	-1	1	IDC	-	-	-	Basal	10	1	c.818G>A	p.R273H
MB-3002	1	4	1	2	2	pos	1	0	0	ILC	+	-	-	LumA	3	0		
MB-3005	1	5	0	2	2	pos	1	0	0	IDC	+	+	-	LumA	8	0		
MB-3006	1	5	1	2	2	pos	1	0	0	IDC	+	-	-	Normal	4	0		
MB-3007	1	5	0	1	2	pos	1	1	0	IDC	+	+	-	LumB	2	0		
MB-3008	1	3	0	1	1	pos	1	0	0	IDC+ILC	+	+	-	LumA	8	0		
MB-3013	1	4	1	2	1	pos	1	0	0	IDC-TUB	+	-	-	LumA	4	0		
MB-3014	1	3	1	1	3	neg	1	0	1	IDC	-	-	-	Basal	1	1	c.1015_1016del2ins1	p.?
MB-3016	1	2	0	1	3	pos	1	0	1	IDC	+	+	-	LumB	2	1	c.1024del1	p.?
MB-3021	1	4	1	2	3	pos	1	0	0	ILC	+	+	-	LumA	8	0		
MB-3025	1	4	1	1	3	neg	3	2	1	IDC	-	-	+	Her2	5	1	c.714_715ins1	p.?
MB-3026	1	5	0	2	3	pos	1	0	0	IDC	+	-	-	LumA	6	0		
MB-3028	1	5	0	1	3	pos	1	2	1	IDC	+	-	+	LumB	9	1	c.743G>A	p.R248Q
MB-3031	1	2	1	2	3	neg	3	2	0	IDC-MED	+	-	+	Her2	5	0		
MB-3032	1	5	0	1	1	pos	1	0	0	IDC	+	+	-	LumA	3	0		
MB-3033	1	4	0	1	2	pos	1	0	0	IDC	+	+	-	LumA	3	0		
MB-3035	1	3	0	2	2	pos	1	-1	0	IDC	+	-	-	LumA	7	0		
MB-3037	1	5	0	1	2	pos	1	0	0	IDC-TUB	+	+	-	LumA	3	0		
MB-3046	1	4	0	2	3	neg	1	0	1	IDC	-	-	-	Basal	10	1	c.331del1	p.?
MB-3049	1	5	0	1	2	pos	1	0	0	ILC	+	+	-	LumA	7	0		
MB-3050	1	4	0	2	3	pos	1	0	0	IDC	+	-	-	LumB	2	0		
MB-3057	1	3	1	2	3	neg	1	0	1	IDC	-	-	-	Basal	10	1	c.412del1	p.?
MB-3058	1	3	1	2	3	neg	1	0	0	IDC	-	-	-	Basal	4	0		
MB-3060	1	5	1	2	3	pos	1	0	0	IDC	+	-	-	LumB	6	0		
MB-3062	1	2	0	1	3	neg	1	0	1	IDC	-	-	-	Basal	10	1	c.718_719ins3	p.?
MB-3063	1	3	1	2	3	neg	1	0	1	IDC	-	-	-	Basal	10	1	c.637C>T	p.R213X
MB-3064	1	5	0	2	1	pos	NA	0	0	IDC-TUB	+	+	-	LumA	4	0		
MB-3067	1	3	1	1	3	neg	1	0	0	IDC-MED	-	-	-	Basal	9	0		
MB-3079	1	4	0	1	2	pos	2	0	0	IDC-TUB	+	+	-	LumA	8	0		
MB-3083	1	4	1	2	3	pos	1	0	0	IDC	+	+	-	LumB	1	0		
MB-3085	1	3	0	2	2	pos	1	0	0	ILC	+	+	-	Normal	4	0		
MB-3088	1	2	0	1	2	pos	1	0	0	IDC	+	+	-	LumA	7	0		
MB-3092	1	3	1	2	3	pos	1	0	0	IDC+ILC	+	+	-	LumB	8	0		
MB-3102	1	5	1	2	2	pos	1	0	0	IDC	+	+	-	LumA	9	0		
MB-3103	1	4	0	1	3	pos	3	2	0	IDC	+	-	+	LumB	5	0		
MB-3104	1	3	0	2	2	pos	1	2	0	IDC	+	-	+	LumA	5	0		
MB-3105	1	3	1	2	1	pos	1	0	0	IDC-TUB	+	+	-	LumA	3	0		
MB-3110	1	3	1	1	2	pos	1	0	0	ILC	+	-	-	LumA	3	0		
MB-3121	1	3	0	1	1	pos	1	0	0	IDC	+	+	-	LumA	3	0		
MB-3122	1	4	0	1	3	pos	1	1	1	IDC	+	-	-	Her2	9	1	c.832C>A	p.P278T
MB-3123	1	3	1	2	3	neg	1	2	1	IDC	-	-	-	Basal	9	1	c.722C>A	p.S241Y
MB-3153	1	4	1	1	3	neg	1	0	1	IDC	-	-	-	Basal	10	1	c.722C>T	p.S241F
MB-3165	1	3	1	2	3	neg	1	-1	1	IDC	+	-	-	Basal	1	1	c.637C>T	p.R213X
MB-3167	1	1	1	1	2	pos	1	1	0	IDC	+	+	-	LumA	8	0		
MB-3171	1	3	0	1	2	pos	1	0	0	IDC	+	+	-	LumA	7	0		
MB-3181	1	2	0	1	3	pos	NA	0	1	IDC-TUB	+	+	-	LumB	2	1	c.1024C>T	p.R342X
MB-3211	1	4	0	1	3	neg	1	0	0	IDC	-	-	-	Basal	10	0		
MB-3218	1	4	1	1	3	neg	1	0	0	IDC	-	-	-	Basal	10	0		
MB-3222	1	3	0	1	1	neg	1	0	0	IDC-TUB	+	+	-	LumA	7	0		
MB-3228	1	4	0	1	2	pos	1	0	0	IDC-TUB	+	+	-	LumA	4	0		
MB-3235	1	4	0	1	3	pos	2	2	1	IDC	+	+	+	LumB	5	1	c.742C>T	p.R248W
MB-3252	1	3	0	2	1	pos	1	0	0	IDC-MUC	+	+	-	LumA	3	0		
MB-3253	1	3	1	2	3	pos	1	0	1	IDC	+	+	-	LumB	9	1	c.814G>A	p.V272M
MB-3254	1	3	0	1	1	pos	NA	0	0	IDC	+	+	-	LumA	3	0		
MB-3277	1	4	1	2	3	neg	1	0	1	IDC	-	-	-	Basal	1	1	c.817C>T	p.R273C
MB-3292	1	4	0	1	3	neg	NA	0	0	IDC-MED	-	-	-	Basal	10	0		
MB-3295	1	3	0	2	3	pos	1	0	0	IDC	+	+	-	Normal	4	0		
MB-3297	1	3	1	2	3	neg	1	0	1	IDC	-	-	-	Basal	10	1	c.1015G>C	p.E339Q
MB-3298	1	3	0	1	2	pos	1	0	0	IDC-TUB	+	+	-	LumA	3	0		
MB-3300	1	3	0	1	2	pos	1	0	0	IDC	+	-	-	LumB	7	0		
MB-3301	1	2	0	2	2	pos	NA	0	0	IDC-TUB	+	+	-	LumB	8	0		

MB-3303	1	5	1	2	3	pos	NA	0	NA	OTHER	+	+	-	LumB	4	NA	NA	NA
MB-3328	1	4	1	2	2	pos	1	0	NA	IDC-TUB	+	-	-	LumA	7	NA	NA	NA
MB-3341	1	5	0	2	2	pos	1	0	0	IDC	+	+	-	LumA	8	0		
MB-3344	1	5	0	2	2	pos	1	-2	0	IDC	+	+	-	LumA	3	0		
MB-3350	1	5	0	2	2	pos	1	0	0	IDC	+	+	-	LumB	3	0		
MB-3351	1	4	0	1	2	pos	1	0	0	IDC	+	+	-	LumA	3	0		
MB-3355	1	3	1	2	3	neg	3	2	0	IDC	-	-	+	Her2	5	0		
MB-3357	1	3	0	2	3	pos	1	1	1	IDC	+	+	-	LumB	9	1	c.734G>A	p.G245D
MB-3360	1	3	0	2	3	pos	3	2	1	IDC	+	+	+	LumB	5	1	c.749C>T	p.P250L
MB-3361	1	3	0	1	3	pos	2	2	1	IDC	+	-	+	LumB	5	1	c.997del1	p.?
MB-3363	1	4	0	2	3	pos	1	0	0	IDC	+	-	-	Basal	10	0		
MB-3365	1	3	1	2	2	pos	1	0	0	IDC	+	+	-	LumA	7	0		
MB-3367	1	4	0	2	3	neg	1	0	1	IDC-MED	+	-	-	Basal	10	1	c.572_581del10	p.?
MB-3371	1	3	0	1	2	pos	1	1	0	IDC	+	-	-	LumB	8	0		
MB-3378	1	3	0	1	2	pos	1	0	0	ILC	+	+	-	LumA	4	0		
MB-3379	1	4	0	2	3	pos	2	2	0	IDC+ILC	+	+	+	LumA	5	0		
MB-3381	1	3	1	2	3	pos	1	1	0	IDC	+	+	-	LumA	7	0		
MB-3382	1	4	0	2	3	pos	3	2	0	IDC	+	-	+	LumB	5	0		
MB-3383	1	5	1	2	3	neg	1	0	1	IDC	-	-	-	Basal	10	1	c.524G>A	p.R175H
MB-3386	1	5	1	2	3	neg	3	2	1	IDC	-	-	+	Her2	1	1	c.743G>A	p.R248Q
MB-3388	1	4	1	2	3	pos	NA	1	1	IDC	+	+	-	LumB	8	1	c.746G>T	p.R249M
MB-3389	1	3	0	2	3	pos	1	1	0	IDC	+	-	-	LumB	6	0		
MB-3395	1	2	0	1	3	neg	1	0	1	IDC	-	-	-	Basal	10	1	c.659A>G	p.Y220C
MB-3396	1	4	1	2	3	neg	1	0	1	IDC	-	-	-	Basal	3	1	c.206del1	p.?
MB-3402	1	3	1	2	3	neg	2	1	0	IDC	+	+	-	LumB	1	0		
MB-3403	1	4	1	1	2	pos	1	0	0	IDC	+	+	-	LumA	3	0		
MB-3412	1	4	1	2	3	pos	3	0	0	IDC	+	-	-	LumA	8	0		
MB-3417	1	3	0	2	2	pos	1	0	0	IDC	+	+	-	LumB	7	0		
MB-3429	1	3	1	2	2	pos	1	0	0	ILC	+	-	-	Normal	4	0		
MB-3430	1	2	1	2	3	pos	1	0	0	IDC	+	+	-	LumA	3	0		
MB-3435	1	3	1	2	3	neg	3	2	0	IDC	-	-	+	Her2	5	0		
MB-3436	1	4	1	3	3	neg	1	-1	0	IDC	+	+	-	LumB	1	0		
MB-3437	1	2	0	2	3	pos	NA	0	0	IDC	+	+	-	LumB	4	0		
MB-3439	1	5	1	1	2	pos	1	0	0	IDC-TUB	+	+	-	LumA	8	0		
MB-3450	1	4	0	1	2	pos	1	-2	0	IDC	+	+	-	LumA	8	0		
MB-3452	1	3	0	2	3	pos	1	0	1	IDC	+	-	-	Normal	2	1	c.686_687del2	p.?
MB-3453	1	4	1	2	3	neg	1	0	1	IDC	-	-	-	Basal	10	1	c.785G>T	p.G262V
MB-3459	1	3	0	2	2	pos	1	2	0	IDC	+	+	-	LumB	7	0		
MB-3462	1	5	1	1	2	pos	1	0	0	IDC	+	+	-	LumA	3	0		
MB-3466	1	2	0	1	3	pos	1	0	0	IDC	+	+	-	Her2	2	0		
MB-3467	1	4	1	2	2	neg	NA	2	1	IDC	-	-	+	Her2	5	1	c.686_687del2	p.?
MB-3470	1	4	1	3	3	neg	3	2	1	IDC	-	-	+	Her2	5	1	c.742C>G	p.R248G
MB-3476	1	3	0	1	3	neg	1	0	0	IDC-MED	-	-	-	Basal	10	0		
MB-3479	1	5	1	2	3	pos	1	0	0	IDC	+	+	-	Normal	4	0		
MB-3487	1	2	0	2	3	pos	1	1	0	IDC	+	-	-	LumB	1	0		
MB-3488	1	5	0	3	3	neg	3	2	1	IDC	+	-	+	Her2	5	1	c.155_164del10	p.?
MB-3490	1	5	0	2	2	pos	1	0	0	IDC-TUB	+	+	-	LumB	3	0		
MB-3492	1	2	1	2	3	pos	1	0	0	IDC	+	+	-	LumB	1	0		
MB-3497	1	3	1	2	3	neg	2	2	0	IDC	-	-	+	Her2	5	0		
MB-3500	1	4	0	2	3	neg	1	0	1	IDC	-	-	-	Basal	10	1	c.150_164del15ins1	p.?
MB-3502	1	2	1	2	3	neg	1	0	1	IDC	-	-	-	Basal	10	1	c.818G>A	p.R273H
MB-3525	1	2	1	2	3	pos	1	1	0	IDC	+	-	-	LumB	1	0		
MB-3526	1	2	1	2	3	pos	2	2	1	IDC	+	-	-	Normal	6	1	c.844C>T	p.R282W
MB-3528	1	3	0	2	3	neg	3	2	0	IDC	-	-	+	Her2	5	0		
MB-3530	1	4	1	2	2	pos	1	0	0	IDC-TUB	+	+	-	Her2	8	0		
MB-3536	1	3	1	1	2	pos	1	2	1	IDC	+	-	-	Normal	5	1	c.574C>T	p.Q192X
MB-3545	1	5	0	2	3	pos	2	0	1	IDC	+	+	-	LumA	7	1	c.1176del1	p.?
MB-3547	1	2	0	2	NA	pos	NA	0	NA	DCIS	+	+	-	LumA	4	NA	NA	NA
MB-3548	1	5	1	2	2	pos	1	0	0	IDC	+	+	-	LumA	3	0		
MB-3556	1	5	1	3	3	neg	3	2	0	IDC	-	-	+	Her2	1	0		
MB-3567	1	4	0	2	3	neg	1	0	0	IDC+ILC	-	-	-	Basal	1	0		
MB-3576	1	2	0	2	3	pos	1	0	1	IDC+ILC	+	-	-	Her2	6	1	c.1020_1021ins5	p.?
MB-3582	1	3	0	3	NA	neg	NA	0	NA	null	+	+	-	Normal	4	NA	NA	NA
MB-3600	1	2	1	1	2	pos	1	0	0	IDC	+	+	-	LumB	9	0		
MB-3606	1	1	0	2	3	neg	3	2	NA	IDC	-	-	+	Her2	5	NA	NA	NA
MB-3614	1	5	1	2	3	pos	1	0	0	IDC	+	+	-	LumB	1	0		
MB-3702	1	5	1	1	3	neg	NA	0	0	IDC	-	-	-	Basal	10	0		
MB-3706	1	3	0	1	3	neg	1	0	1	IDC	-	-	-	Basal	10	1	c.637C>T	p.R213X
MB-3707	1	3	1	2	3	pos	1	0	1	IDC	+	-	-	LumA	4	1	c.853G>A	p.E285K
MB-3711	1	2	0	2	1	pos	1	1	0	IDC	+	+	-	LumA	3	0		
MB-3748	1	3	0	1	3	pos	1	0	1	IDC	+	+	-	LumA	4	1	c.916C>T	p.R306X
MB-3752	1	3	0	1	3	neg	1	-1	1	IDC-MED	-	-	-	Basal	4	1	c.586C>T	p.R196X
MB-3754	1	4	0	2	3	pos	1	0	0	IDC	+	-	-	LumB	1	0		
MB-3781	1	3	0	1	2	pos	1	0	0	IDC-TUB	+	+	-	LumA	8	0		
MB-3797	1	5	0	2	NA	pos	NA	-2	NA	null	+	+	-	LumA	8	NA	NA	NA
MB-3823	1	3	0	2	2	pos	1	-1	0	IDC	+	+	-	LumA	3	0		
MB-3824	1	3	0	2	3	pos	1	0	1	IDC	+	+	-	LumB	2	1	c.485_498del14	p.?
MB-3838	1	3	0	2	3	pos	1	0	0	IDC	+	+	-	LumB	8	0		
MB-3840	1	3	0	1	2	pos	1	0	0	IDC	+	-	-	LumB	6	0	c.213C>T	p.P71P
MB-3842	1	3	1	2	3	pos	1	0	0	IDC	+	-	-	LumB	1	0		
MB-3850	1	4	0	2	2	pos	1	2	0	IDC+ILC	+	+	+	LumA	4	0		
MB-3852	1	2	0	2	3	pos	1	2	0	IDC	+	+	+	LumA	4	0		
MB-0000	2	4	1	2	3	pos	NA	0	0	IDC	+	-	-	Normal	4	0		
MB-0002	2	2	0	1	3	pos	NA	0	1	IDC	+	+	-	LumA	4	1	c.533A>C	p.H178P
MB-0005	2	4	1	1	2	pos	NA	0	1	IDC	+	+	-	LumB	3	1	c.542G>A	p.R181H
MB-0006	2	2	1	2	2	pos	0	0	0	IDC	+	+	-	LumB	9	0		
MB-0008	2	3	1	2	3	pos	0	0	1	IDC	+	+	-	LumB	9	1	c.722C>T	p.S241F
MB-0010	2	3	0	2	3	pos	NA	0	1	IDC	+	+	-	LumB	7	1	c.200del1	p.?
MB-0014	2	2	1	1	2	pos	NA	-1	0	IDC	+	+	-	LumB	3	0		
MB-0022	2	4	1	2	2	pos	NA	0	0	IDC+ILC	+	-	-	LumB	3	0		
MB-0028	2	3	1	1	3	pos	NA	1	1	IDC	+	-	-	LumB	9	1	c.724T>C	p.C242R
MB-0035	2	5	0	2	2	neg	NA	-1	0	ILC	+	-	-	Her2	3	0		
MB-0036	2	4	0	2	2	pos	NA	0	0	IDC	+	+	-	LumA	3	0		
MB-0039	2	3	0	2	1	pos	NA	2	0	OTHER INVASIVE	+	+	-	LumB	4	0		
MB-0045	2	4	1	1	3	neg	NA	0	0	IDC	-	+	-	Basal	4	0		
MB-0046	2	4	1	2	3	pos	NA	2	1	IDC	+	+	+	LumA	5	1	c.403T>C	p.C135R
MB-0048	2	4	1	2	2	pos	NA	2	0	IDC+ILC	+	+	+	Her2	4	0		
MB-0050	2	4	1	2	2	pos	0	0	1	ILC	+	+	-	Normal	8	1	c.533A>C	p.H178P
MB-0053	2	3	0	2	2	pos	NA	0	1	IDC	+	-	-	LumB	7	1	c.542G>A	p.R181H
MB-0054	2	3	0	2	3	pos	NA	1	1	IDC	+	+	-	LumB	10	1	c.164C>T	p.T55I
MB-0056	2	5	0	2	2	pos	NA	0	0	IDC+ILC	+	+	-	LumB	1	0		
MB-0059	2	3	0	1	3	pos	NA	0	0	IDC	+	+	-	LumA	8	0		
MB-0060	2	4	0	2	3	pos	NA	0	1	IDC	+	+	-	LumB	10	1	c.971A>G	p.D324G
MB-0062	2	3	0	1	3	neg	NA	0	1	IDC-MED	-	-	-	Basal	10	1	c.902_904del1ins2	p.?
MB-0064	2	2	0	1	2	pos	NA	1	0	IDC	+	+	-	LumB	3	0		
MB-0066	2	3	1	1	2	pos	NA	0	0	IDC	+	+	-	LumB	7	0		
MB-0068	2	2	1	1	1	pos	NA	-1	0	IDC-TUB	+	+	-	LumA	3	0		
MB-0071	2	4	1	3	2	pos	NA	0	0	IDC+ILC	+	-	-	LumB	8	0		
MB-0079	2	2	1	2	3	neg	0	0	1	IDC	-	-	-	Her2	10	1	c.817C>T	p.R273C
MB-0081	2	3	0	2	2	pos	NA	0	0	IDC	+	+	-	LumB	3	0		
MB-0083	2	3	0	1	2	pos	NA	0	0	ILC	+	+	-	LumB	2	0		
MB-0093	2	2	1	1	3	pos	0	0	1	IDC+ILC	+	+	-	LumB	3	1	c.835G>A	p.G279R
MB-0095	2	4	1	3	2	pos	NA	0	0	IDC	+	+	-	LumB	8	0		
MB-0097	2	5	1	2	3	pos	NA	0	0	IDC	+	+	-	LumA	8	0		
MB-0099	2	3	0	2	2	pos	0	-1	0	IDC+ILC	+	+	-	LumB	4	0		
MB-0100	2	3	0	2	3	neg	NA	0	NA	IDC	-	-	-	Basal	10	NA	NA	NA
MB-0101	2	3	0	2	2	pos	NA	0	0	ILC	+	+	-	Normal	8	0		
MB-0102	2	4	1	2	2	pos	1	0	0	ILC	+	+	-	LumB	3	0		
MB-0106	2	3	1	3	1	pos	NA	0	NA	IDC	+	+	-	LumA	8	NA	NA	NA
MB-0107	2	2	0	1	3	pos	NA	0	1	IDC	+	+	-	LumB	1	1	c.530C>G	p.P177R
MB-0108	2	3	0	1	3	pos	NA	0	0	IDC	+	-	-	LumB	4	0		
MB-0109	2	2	0	2	3	pos	NA	1	NA	IDC	+	+	-	Basal	9	NA	NA	NA
MB-0110	2	5	0	3	NA	NA	NA	0	NA	PHYL	-	-	-	Normal	3	NA	NA	NA
MB-0111	2	4	0	2	1	pos	NA	0	0	IDC	+	+	-	LumA	3	0		
MB-0112	2	5	1	3	3	pos	NA	0	0	ILC	+	-	-	LumA	3	0		
MB-0113	2	4	1	1	3	pos	3	2	0	IDC	-	-	+	Her2	5	0		
MB-0114	2	5	0	2	2	pos	NA	0	0	IDC	+	+	-	LumA	8	0		
MB-0115	2	5	0	2	3	neg	1	0	1	IDC	-	-	-	Basal	10	1	c.524G>A	p.R175H
MB-0116	2	1	1	3	2	pos	NA	0	0	ILC	+	+	-	Normal	6	0		
MB-0117	2	4	1	2	2	pos	NA	0	0	IDC+ILC	+	-	-	LumA	1	0		
MB-0119	2	5	1	2	2	pos	NA	1	0	IDC	+	-	-	LumB	7	0		
MB-0120	2	4	1	2	3	pos	3	2	0	IDC	+	-	+	LumB	9	0		
MB-0121	2	4	1	2	2	pos	NA	0	0	IDC	+	-	-	LumA	8	0		
MB-0122	2	3	1	2	1	pos	NA	0	NA	OTHER	+	+	-	LumA	8	NA	NA	NA
MB-0123	2	5	0	2	3	pos	NA	0	0	IDC	+	+	-	LumB	1	0		
MB-0124	2	2	1	3	2	pos	1	0	0	IDC+ILC	+	+	-	LumA	8	0		
MB-0125	2	3	0	1	1	pos	NA	0	0	IDC	+	+	-	LumB	8	0		
MB-0126	2	3	0	2	2	pos	NA	0	0	IDC	+	+	-	LumA	8	0		
MB-0127	2	2	1	1	2	pos	NA	0	0	IDC	-	-	-	Normal	3	0		
MB-0128	2	3	0	1	2	pos	NA	0	0	IDC	+	+	-	Normal	3	0		
MB-0129	2	4	1	2	3	neg	3	2	0	IDC	-	-	+	Her2	5	0		
MB-0130	2	3	0	1	1	pos	NA	2	0	IDC	+	-	+	LumA	5	0		
MB-0131	2	3	0	2	3	pos	NA	0	0	IDC	+	-	-	LumA	8	0		
MB-0133	2	3	1	3	1	pos	NA	0	0	IDC-TUB	+	-	-	LumA	8	0		

MB-0134	2	1	1	2	3	pos	NA	0	0	IDC	+	-	-	LumB	8	0		
MB-0135	2	4	1	2	3	pos	NA	0	0	IDC	+	+	-	LumB	2	0		
MB-0136	2	4	0	1	2	pos	NA	0	0	IDC	+	+	-	LumA	3	0		
MB-0138	2	3	0	1	2	pos	NA	-1	0	ILC	+	+	-	LumA	3	0		
MB-0139	2	2	1	2	2	pos	NA	0	0	ILC	+	+	-	LumA	3	0		
MB-0140	2	3	1	2	2	pos	NA	0	0	IDC+ILC	+	-	-	LumB	8	0		
MB-0142	2	4	0	1	2	pos	NA	-1	0	IDC-MUC	+	+	-	LumA	1	0		
MB-0143	2	4	1	2	3	pos	NA	0	0	IDC	+	-	-	LumA	9	0		
MB-0144	2	4	1	2	3	pos	NA	0	0	IDC	+	+	-	LumB	8	0		
MB-0145	2	4	0	2	2	pos	NA	0	0	ILC	+	+	-	LumA	3	0		
MB-0146	2	2	1	2	3	pos	NA	-1	0	IDC	+	-	-	LumB	1	0		
MB-0147	2	5	1	2	2	pos	NA	0	0	ILC	+	+	-	LumA	2	0		
MB-0148	2	3	0	1	3	neg	NA	2	1	IDC	-	-	+	Her2	5	1	c.574C>T	p.Q192X
MB-0149	2	2	0	2	3	neg	NA	-1	1	IDC	-	-	-	Basal	10	1	c.524G>A	p.R175H
MB-0150	2	4	0	2	2	pos	NA	0	NA	OTHER	+	-	-	Basal	4	NA	NA	NA
MB-0151	2	3	1	3	3	pos	2	0	0	IDC	+	+	-	LumB	2	0		
MB-0152	2	3	0	1	3	neg	NA	2	1	IDC	-	-	+	Her2	4	1	c.793_808del16	p.?
MB-0153	2	4	0	2	NA	NA	NA	0	NA	PHYL	-	-	-	Basal	4	NA	NA	NA
MB-0154	2	3	0	1	2	pos	NA	0	0	IDC	+	-	-	LumA	4	0		
MB-0155	2	4	0	1	1	pos	NA	0	0	IDC-TUB	+	-	-	Normal	4	0		
MB-0156	2	3	0	1	1	NA	NA	0	NA	DCIS	+	-	-	Normal	4	NA	NA	NA
MB-0157	2	3	0	2	3	neg	NA	0	1	IDC	-	-	-	Basal	4	1	c.488A>G	p.Y163C
MB-0158	2	4	0	2	3	neg	NA	0	1	IDC	-	-	-	Basal	10	1	c.1014del1	p.?
MB-0159	2	5	0	2	NA	NA	NA	2	NA	DCIS	-	-	+	Basal	5	NA	NA	NA
MB-0160	2	4	0	2	NA	NA	NA	0	NA	PHYL	-	+	-	Normal	4	NA	NA	NA
MB-0162	2	4	1	1	2	pos	NA	0	0	IDC	+	+	-	LumA	7	0		
MB-0163	2	2	1	2	3	neg	NA	0	1	IDC	-	-	-	Basal	10	1	c.316_328del13ins2	p.?
MB-0164	2	2	0	1	3	neg	NA	0	1	IDC	-	-	-	Basal	10	1	c.524G>A	p.R175H
MB-0165	2	3	1	3	3	pos	3	2	0	IDC	+	-	+	LumA	5	0		
MB-0166	2	5	1	2	3	pos	NA	0	0	IDC	+	+	-	LumA	4	0		
MB-0167	2	5	1	2	3	pos	1	0	1	IDC	+	+	-	LumB	9	1	c.844C>T	p.R282W
MB-0168	2	4	0	1	3	pos	1	0	0	IDC	+	-	-	Normal	4	0		
MB-0169	2	3	0	2	2	pos	NA	0	0	ILC	+	+	-	Normal	4	0		
MB-0170	2	5	1	2	1	pos	NA	0	NA	OTHER INVASIVE	+	-	-	LumA	3	NA	NA	NA
MB-0171	2	3	0	2	3	pos	NA	0	0	IDC+ILC	+	-	-	Normal	4	0		
MB-0172	2	NA	1	2	2	pos	1	0	0	IDC	+	+	-	LumA	3	0		
MB-0173	2	2	1	2	3	pos	NA	-1	0	IDC	+	+	-	LumB	7	0		
MB-0174	2	2	0	2	3	neg	1	0	1	IDC	-	-	-	Basal	4	1	c.153del1	p.?
MB-0175	2	1	0	2	1	pos	NA	-1	0	IDC-TUB	+	-	-	LumA	6	0		
MB-0176	2	2	1	2	3	pos	1	0	0	IDC	+	+	-	LumA	1	0		
MB-0177	2	2	1	2	2	pos	NA	0	0	IDC	+	+	-	LumA	6	0		
MB-0178	2	3	0	2	3	pos	NA	0	0	IDC	+	+	-	LumB	1	0		
MB-0179	2	4	1	2	3	neg	3	1	0	IDC	-	-	-	Basal	4	0		
MB-0180	2	3	0	1	2	pos	1	0	0	IDC	+	+	-	LumA	8	0		
MB-0181	2	3	1	2	2	pos	NA	0	0	IDC	+	+	-	LumA	7	0		
MB-0184	2	4	0	2	2	pos	1	0	0	IDC-MUC	+	+	-	LumA	4	0		
MB-0185	2	2	1	3	3	pos	NA	1	1	IDC	+	+	-	LumB	9	1	c.832C>T	p.P278S
MB-0188	2	3	1	2	2	pos	1	0	0	ILC	-	-	-	Her2	3	0		
MB-0189	2	3	1	2	3	pos	1	0	0	IDC	+	-	-	Normal	6	0		
MB-0191	2	4	0	2	3	neg	NA	0	1	IDC	-	-	-	Basal	10	1	c.578A>G	p.H193R
MB-0192	2	5	0	1	1	pos	NA	0	0	IDC	+	-	-	Normal	4	0		
MB-0193	2	3	1	1	3	pos	1	2	0	IDC	+	+	-	LumB	3	0		
MB-0194	2	3	1	2	2	pos	NA	0	0	IDC	+	+	-	LumA	3	0		
MB-0195	2	5	1	2	3	pos	2	1	0	IDC	+	-	-	LumB	6	0		
MB-0197	2	4	1	2	3	pos	1	0	0	IDC	+	+	-	LumB	9	0		
MB-0198	2	3	1	1	3	pos	1	0	0	IDC+ILC	+	+	-	LumB	8	0		
MB-0199	2	4	1	1	3	pos	NA	0	0	IDC+ILC	+	+	-	LumA	3	0		
MB-0200	2	3	0	2	3	neg	NA	0	1	IDC	-	-	-	Basal	10	1	c.586C>T	p.R196X
MB-0201	2	4	0	2	3	neg	3	2	1	IDC	-	-	+	Basal	5	1	c.734G>A	p.G245N
MB-0202	2	3	1	2	3	pos	1	0	1	IDC	+	+	-	LumA	4	1	c.841G>C	p.D281H
MB-0203	2	3	1	2	3	pos	NA	0	0	IDC	+	+	-	LumA	7	0		
MB-0204	2	4	0	1	1	pos	NA	0	0	IDC	+	-	-	LumA	3	0		
MB-0205	2	3	0	2	2	pos	NA	0	0	ILC	+	-	-	Normal	3	0		
MB-0206	2	4	1	2	3	neg	1	0	1	IDC-MED	-	-	-	Basal	10	1	c.405C>G	p.C135W
MB-0207	2	3	1	2	2	pos	NA	1	0	IDC	+	+	-	LumA	8	0		
MB-0209	2	3	0	1	2	pos	NA	0	0	IDC	+	-	-	Normal	4	0		
MB-0210	2	3	1	1	2	pos	NA	0	0	IDC	-	-	-	Basal	4	0		
MB-0211	2	3	1	2	3	pos	1	0	1	IDC	-	-	-	Basal	9	1	c.743G>A	p.R248Q
MB-0214	2	4	0	2	3	pos	NA	0	1	IDC	-	-	-	Basal	10	1	c.469G>T	p.V157F
MB-0215	2	3	0	2	2	pos	NA	0	0	IDC+ILC	+	+	-	LumB	3	0		
MB-0218	2	3	1	2	3	pos	1	0	0	IDC	+	+	-	LumB	1	0		
MB-0220	2	5	1	2	3	pos	3	1	0	IDC	-	-	-	Normal	4	0		
MB-0221	2	5	1	2	3	neg	NA	1	1	IDC	-	-	-	Her2	4	1	c.590T>G	p.V197G
MB-0222	2	2	0	2	NA	pos	NA	0	0	ILC	+	+	-	Normal	4	0		
MB-0223	2	2	1	2	3	pos	1	1	0	IDC	+	-	-	LumA	4	0		
MB-0224	2	4	0	2	3	pos	NA	0	0	IDC	+	+	-	LumA	4	0		
MB-0225	2	2	0	1	2	pos	NA	2	0	IDC	+	-	+	LumB	1	0		
MB-0226	2	3	1	2	1	pos	NA	0	0	IDC	+	+	-	LumA	8	0		
MB-0227	2	3	0	2	2	pos	NA	0	0	IDC	+	+	-	LumA	8	0		
MB-0228	2	4	1	1	1	pos	NA	0	0	ILC	+	+	-	Normal	4	0		
MB-0229	2	2	0	1	2	pos	NA	0	0	IDC	+	+	-	LumA	7	0		
MB-0230	2	3	0	2	NA	NA	NA	2	NA	DCIS	-	-	+	Her2	5	NA	NA	NA
MB-0231	2	4	0	2	NA	NA	NA	1	0	IDC-MUC	+	-	-	LumA	3	0		
MB-0232	2	4	0	1	2	pos	NA	0	0	IDC	+	+	-	LumA	8	0		
MB-0233	2	5	0	1	2	NA	NA	1	1	IDC	+	+	-	LumA	2	1	c.840A>T	p.R280S
MB-0234	2	5	0	2	NA	pos	NA	0	0	ILC	+	+	-	LumB	8	0		
MB-0235	2	5	1	2	2	pos	NA	0	0	IDC	+	+	-	LumA	8	0		
MB-0236	2	2	0	2	3	pos	NA	2	0	IDC	+	+	+	LumA	5	0		
MB-0238	2	4	1	2	3	neg	NA	0	1	IDC	-	-	-	Basal	10	1	c.743G>A	p.R248Q
MB-0239	2	2	0	2	1	pos	NA	0	0	IDC+ILC	+	+	-	LumA	3	0		
MB-0241	2	2	0	2	3	pos	1	0	1	IDC	+	-	-	Basal	4	1	c.476C>T	p.A159V
MB-0242	2	3	0	1	2	NA	NA	0	0	IDC	+	+	-	LumB	8	0		
MB-0243	2	3	1	2	2	pos	NA	0	0	IDC	+	+	-	LumB	8	0		
MB-0244	2	3	0	1	1	pos	NA	0	0	IDC	+	+	-	Normal	4	0		
MB-0245	2	1	0	1	NA	pos	NA	0	0	ILC	+	-	-	LumA	3	0		
MB-0247	2	1	1	1	2	pos	NA	0	0	IDC	+	+	-	LumA	4	0		
MB-0248	2	3	0	2	3	pos	NA	0	0	IDC	+	+	-	LumA	8	0		
MB-0249	2	2	0	1	3	neg	NA	1	1	IDC	-	-	-	Her2	10	1	c.614A>C	p.Y205S
MB-0253	2	4	0	1	2	pos	NA	0	0	IDC	+	+	-	Normal	4	0		
MB-0256	2	3	0	1	1	pos	NA	0	0	IDC	+	+	-	LumA	3	0		
MB-0257	2	4	0	2	2	pos	3	1	0	IDC	+	-	-	LumA	8	0		
MB-0258	2	3	0	1	3	pos	NA	1	0	IDC	+	+	-	LumA	7	0		
MB-0259	2	3	1	NA	3	neg	NA	2	1	IDC	-	-	-	Basal	9	1	c.818G>A	p.R273H
MB-0260	2	3	0	1	NA	pos	NA	0	1	ILC	+	+	-	LumA	3	1	c.402T>A	p.F134L
MB-0261	2	2	1	2	1	pos	NA	0	0	IDC	+	+	-	LumB	8	0		
MB-0262	2	2	0	2	3	pos	NA	0	1	IDC	+	-	-	Her2	7	1	c.393_395del3	p.?
MB-0263	2	3	0	2	NA	pos	NA	0	0	ILC	+	+	-	LumA	7	0		
MB-0264	2	4	1	2	3	NA	NA	0	0	IDC	+	-	-	Her2	9	0		
MB-0265	2	5	1	2	2	pos	NA	0	1	IDC	-	-	-	Basal	4	1	c.722C>A	p.S241Y
MB-0266	2	5	0	2	NA	NA	NA	1	0	ILC	+	+	-	LumA	8	0		
MB-0268	2	5	0	1	3	pos	NA	0	1	IDC	+	+	-	LumB	9	1	c.916C>T	p.R306X
MB-0269	2	3	1	2	3	neg	NA	0	0	IDC	-	-	-	Basal	4	0		
MB-0270	2	4	0	1	3	pos	NA	0	0	IDC	+	+	-	LumB	10	0		
MB-0272	2	2	1	2	3	pos	1	0	0	IDC	+	-	-	LumB	8	0		
MB-0273	2	1	1	2	2	pos	NA	0	0	IDC+ILC	+	-	-	LumA	8	0		
MB-0275	2	2	0	1	1	pos	NA	0	0	IDC-MUC	+	-	-	Normal	4	0		
MB-0278	2	5	1	3	3	pos	1	0	1	IDC	-	-	-	Basal	10	1	c.809T>C	p.F270S
MB-0279	2	4	0	2	1	pos	NA	-2	0	ILC	+	+	-	Normal	4	0		
MB-0280	2	3	0	2	2	pos	NA	0	0	IDC+ILC	+	+	-	Normal	7	0		
MB-0281	2	4	0	1	NA	NA	NA	0	NA	BEGIN	+	-	-	Normal	4	NA	NA	NA
MB-0282	2	3	1	1	3	pos	NA	0	NA	IDC-MUC	+	+	-	Normal	4	NA	NA	NA
MB-0283	2	5	1	2	3	pos	NA	0	0	IDC	+	-	-	LumB	1	0		
MB-0284	2	5	NA	1	NA	NA	NA	0	NA	BEGIN	-	-	-	Basal	10	NA	NA	NA
MB-0285	2	2	1	2	2	pos	NA	0	0	IDC+ILC	+	+	-	Normal	4	0		
MB-0286	2	5	0	2	3	pos	NA	0	0	IDC	+	-	-	LumA	4	0		
MB-0287	2	5	1	1	2	pos	NA	0	0	IDC	+	-	-	LumB	7	0		
MB-0288	2	5	1	2	2	pos	NA	2	0	ILC	+	-	+	Normal	1	0		
MB-0289	2	5	0	2	3	pos	NA	0	0	IDC	-	-	-	Basal	4	0		
MB-0290	2	4	0	2	1	pos	NA	0	0	IDC+ILC	+	+	-	LumA	8	0		
MB-0291	2	5	1	2	3	pos	NA	2	0	IDC	+	+	+	LumB	5	0		
MB-0292	2	4	1	3	3	neg	NA	0	1	IDC	-	-	-	Her2	9	1	c.582_583ins1	p.?
MB-0293	2	5	0	1	1	pos	NA	0	0	IDC-MUC	+	-	-	Normal	4	0		
MB-0294	2	2	0	1	2	pos	NA	1	0	IDC	-	-	-	Normal	4	0		
MB-0295	2	3	1	1	1	pos	NA	-2	0	IDC-TUB	+	+	-	LumA	4	0		
MB-0299	2	4	0	2	NA	NA	NA	0	NA	DCIS	-	-	-	Basal	10	NA	NA	NA
MB-0301	2	4	1	2	3	pos	NA	0	0	IDC	+	+	-	LumA	7	0		
MB-0302	2	4	1	2	3	pos	NA	0	0	ILC	+	-	-	LumA	3	0		
MB-0303	2	3	0	1	3	neg	NA	0	0	IDC	-	-	-	Basal	10	0		
MB-0304	2	2	1	1	3	pos	NA	0	1	IDC	+	+	-	LumB	6	1	c.578A>T	p.H193L
MB-0305	2	5	1	2	3	pos	NA	0	0	IDC+ILC	+	+	-	Normal	8	0		
MB-0306	2	3	1	2	3	pos	NA	0	1	ILC	+	+	-	LumA	3	1	c.797G>A	p.G266E
MB-0307	2	2	1	1	3	pos	NA	2	0	IDC	+	-	+	LumA	5	0		
MB-0308	2	3	0	1	1	pos	NA	0	0	IDC-TUB	+	-	-	LumA	3	0		
MB-0309	2	5	1	2	1	pos	NA	0	0	IDC	+	+	-	LumA	8	0		

MB-0310	2	4	1	1	2	pos	NA	0	0	IDC	+	+	-	LumA	3	0		
MB-0311	2	2	0	2	1	pos	NA	0	0	IDC	+	-	-	LumB	8	0		
MB-0312	2	3	0	2	2	pos	NA	0	0	IDC+ILC	+	+	-	LumB	2	0		
MB-0313	2	2	1	2	3	pos	NA	0	1	IDC	+	+	-	LumB	10	1	c.659 A>G	p.Y220C
MB-0314	2	4	1	3	3	neg	NA	2	NA	IDC	-	-	+	Her2	5	NA	NA	NA
MB-0315	2	4	1	3	2	pos	NA	1	1	IDC	+	-	-	LumA	6	1	c.572del1	p.?
MB-0316	2	4	0	2	3	neg	NA	0	1	IDC	-	-	-	Basal	10	1	c.451C>T	p.P151S
MB-0317	2	5	0	2	3	pos	NA	0	0	IDC	+	+	-	LumA	7	0		
MB-0318	2	3	0	1	NA	neg	NA	0	0	OTHER INVASIVE	-	-	-	Normal	4	0		
MB-0319	2	4	1	2	2	pos	NA	1	0	IDC+ILC	+	-	-	LumB	2	0		
MB-0320	2	3	0	1	1	pos	NA	0	0	ILC	+	+	-	LumA	3	0		
MB-0321	2	4	1	2	3	pos	NA	0	0	IDC	+	+	-	LumA	8	0		
MB-0322	2	3	1	2	2	pos	NA	0	0	IDC	+	-	-	LumA	8	0		
MB-0324	2	3	1	2	3	pos	1	1	0	IDC	+	+	-	LumA	6	0		
MB-0325	2	3	0	2	3	pos	NA	0	1	IDC	+	+	-	LumB	10	1	c.636del1	p.?
MB-0327	2	5	0	2	2	pos	NA	0	0	IDC	+	+	-	LumA	3	0		
MB-0328	2	2	1	2	3	pos	NA	0	0	IDC	+	+	-	LumB	9	0		
MB-0333	2	3	0	NA	NA	NA	NA	1	1	OTHER INVASIVE	-	-	-	Basal	10	1	c.817C>T	p.R273C
MB-0336	2	3	1	2	3	pos	NA	0	0	IDC	+	+	-	LumB	10	0		
MB-0339	2	4	0	2	2	NA	NA	0	0	IDC-MUC	+	+	-	LumB	4	0		
MB-0340	2	5	1	1	3	pos	NA	0	1	IDC	-	-	-	Basal	10	1	c.783-8_790del19	p.?
MB-0341	2	4	1	2	2	pos	NA	0	0	IDC	+	+	-	LumA	8	0		
MB-0342	2	4	0	2	2	pos	NA	0	0	ILC	+	+	-	Normal	3	0		
MB-0343	2	3	1	2	2	pos	NA	0	0	IDC	+	+	-	Normal	6	0		
MB-0344	2	4	1	2	2	pos	NA	0	0	ILC	+	+	-	Normal	3	0		
MB-0345	2	4	1	2	2	pos	NA	0	0	IDC	+	+	-	LumA	3	0		
MB-0346	2	4	1	1	3	neg	3	NA	1	IDC	-	-	+	Her2	5	1	c.817C>T	p.R273C
MB-0347	2	4	0	2	3	pos	1	0	1	IDC	+	-	-	Basal	4	1	c.1015G>T	p.E339X
MB-0348	2	3	0	2	1	pos	NA	0	0	IDC	+	+	-	LumA	8	0		
MB-0349	2	2	1	2	3	pos	NA	-1	0	IDC	+	-	-	LumB	3	0		
MB-0350	2	4	NA	3	3	neg	NA	0	1	IDC-MED	-	-	-	Basal	10	1	c.742C>G	p.R248G
MB-0351	2	4	NA	NA	NA	pos	NA	0	0	IDC-MUC	+	-	-	LumB	3	0		
MB-0352	2	5	0	1	3	pos	NA	0	0	IDC	-	-	-	Her2	4	0		
MB-0353	2	4	1	2	2	pos	NA	0	1	IDC+ILC	+	-	-	LumA	7	1	c.408A>T	p.Q136H
MB-0354	2	4	1	1	3	neg	NA	0	0	IDC	-	-	-	Basal	4	0		
MB-0356	2	4	0	1	2	pos	NA	0	0	IDC+ILC	+	+	-	LumA	8	0		
MB-0358	2	3	1	2	3	pos	NA	1	1	IDC	+	+	-	LumA	9	1	c.673-2A>G	NA
MB-0359	2	4	1	2	2	pos	NA	0	0	IDC	+	+	-	LumA	3	0		
MB-0360	2	5	1	3	1	pos	NA	0	0	IDC	+	+	-	LumB	7	0		
MB-0361	2	5	1	2	3	neg	NA	2	1	IDC	-	-	+	Her2	5	1	c.743G>A	p.R248Q
MB-0362	2	4	1	1	2	pos	1	0	0	IDC+ILC	+	+	-	LumA	2	0		
MB-0363	2	5	1	3	2	pos	NA	0	NA	OTHER	+	-	-	LumB	2	NA	NA	NA
MB-0364	2	4	1	3	2	pos	1	0	0	IDC	+	-	-	LumA	4	0		
MB-0365	2	2	0	1	2	pos	NA	2	1	IDC-MUC	+	-	+	Her2	4	1	c.994-1G>C	NA
MB-0366	2	4	0	2	2	pos	NA	0	0	IDC	+	+	-	LumA	8	0		
MB-0367	2	3	0	1	2	pos	NA	0	0	IDC	+	-	-	Normal	4	0		
MB-0368	2	3	1	2	3	pos	NA	0	0	IDC	+	+	-	LumB	2	0		
MB-0369	2	4	1	2	3	pos	NA	1	0	IDC+ILC	+	-	-	LumB	1	0		
MB-0370	2	3	1	2	3	pos	1	0	0	IDC	+	-	-	LumB	9	0		
MB-0371	2	3	1	2	3	pos	3	2	0	IDC	+	-	+	LumB	5	0		
MB-0372	2	4	0	1	3	neg	1	0	1	IDC	-	-	-	Basal	10	1	c.853G>T	p.E285X
MB-0373	2	3	1	2	3	pos	NA	2	1	IDC	+	+	+	LumB	5	1	c.700T>C	p.Y234H
MB-0374	2	4	1	2	3	pos	1	-1	0	IDC	+	-	-	LumB	1	0		
MB-0375	2	5	1	2	3	neg	NA	0	1	IDC	+	+	-	Normal	10	1	c.219_229del11	p.?
MB-0377	2	2	0	1	2	pos	NA	0	0	IDC	+	+	-	LumA	4	0		
MB-0378	2	3	1	2	3	neg	3	2	1	IDC	-	-	+	Her2	5	1	c.58del1	p.?
MB-0379	2	3	1	2	2	pos	NA	0	0	IDC+ILC	+	-	-	LumA	7	0		
MB-0380	2	3	1	2	3	pos	NA	0	1	IDC	+	+	-	LumB	9	1	c.715A>G	p.N239D
MB-0381	2	3	0	2	3	pos	3	2	NA	IDC	+	-	+	Her2	5	NA	NA	NA
MB-0382	2	1	1	2	2	pos	1	0	NA	IDC	+	-	-	LumA	3	NA	NA	NA
MB-0383	2	2	0	1	3	pos	2	0	0	IDC	+	-	-	LumB	1	0		
MB-0384	2	2	1	2	3	pos	NA	-1	0	IDC	+	+	-	LumB	8	0		
MB-0385	2	1	1	2	3	pos	1	0	NA	IDC-MUC	+	-	-	LumB	9	NA	NA	NA
MB-0386	2	4	1	1	3	pos	1	-1	1	IDC	+	+	-	LumA	9	1	c.1046_1053del8	p.?
MB-0388	2	2	1	2	3	pos	3	2	0	IDC	+	-	+	Normal	1	0		
MB-0389	2	5	1	3	3	pos	3	1	0	IDC	+	+	+	LumB	2	0		
MB-0390	2	3	1	1	3	pos	3	2	NA	IDC	-	-	+	Basal	5	NA	NA	NA
MB-0391	2	3	1	3	3	neg	3	2	0	IDC	-	-	+	Her2	1	0		
MB-0392	2	5	1	2	3	pos	1	0	0	IDC	+	+	-	Normal	1	0		
MB-0393	2	2	1	3	3	pos	1	0	0	IDC	+	+	-	Basal	2	0		
MB-0394	2	2	0	2	2	pos	NA	0	0	IDC-MUC	+	+	-	LumA	2	0		
MB-0395	2	3	1	3	3	neg	3	2	1	IDC	-	-	+	Her2	5	1	c.342_343ins1	p.?
MB-0396	2	3	0	2	3	neg	1	0	1	IDC	-	-	-	Basal	10	1	c.536A>G	p.H179R
MB-0397	2	1	0	1	1	pos	NA	0	0	IDC-TUB	+	+	-	LumA	4	0		
MB-0398	2	2	1	2	3	pos	2	0	NA	IDC	+	-	-	LumB	6	NA	NA	NA
MB-0399	2	2	1	2	3	neg	1	0	0	IDC	-	-	-	Her2	4	0		
MB-0400	2	4	0	1	3	neg	1	0	1	IDC	-	-	-	Basal	10	1	c.817C>T	p.R273C
MB-0401	2	3	0	2	3	neg	3	0	1	IDC	-	-	-	Basal	10	1	c.441-444del4	p.?
MB-0402	2	2	0	1	1	pos	NA	0	0	IDC-TUB	+	+	-	Normal	4	0		
MB-0403	2	3	0	1	NA	NA	NA	0	NA	BEGIN	-	-	-	Basal	4	NA	NA	NA
MB-0404	2	4	1	1	3	pos	NA	-1	0	IDC	+	+	-	LumA	8	0		
MB-0405	2	1	0	1	2	pos	NA	0	NA	OTHER	+	+	-	LumB	7	NA	NA	NA
MB-0406	2	2	1	3	3	pos	2	0	1	IDC	+	-	-	LumB	2	1	c.402T>A	p.F134L
MB-0408	2	4	1	2	2	pos	NA	0	0	IDC	+	+	-	LumA	7	0		
MB-0410	2	2	0	2	2	pos	NA	0	0	ILC	+	+	-	LumA	3	0		
MB-0411	2	3	1	1	2	pos	1	-1	1	IDC	+	-	-	LumA	6	1	c.733_735del3	p.?
MB-0412	2	3	1	1	3	pos	NA	0	1	IDC	+	-	-	LumB	6	1	c.304del1	p.?
MB-0413	2	3	1	2	3	pos	1	0	0	IDC	+	+	-	LumA	2	0		
MB-0414	2	2	0	2	3	neg	1	-1	1	IDC	-	-	-	Basal	10	1	c.512del1	p.?
MB-0417	2	2	1	2	2	pos	NA	0	0	IDC	+	+	-	Her2	7	0		
MB-0418	2	3	1	2	3	pos	NA	0	0	IDC	+	-	-	Her2	9	0		
MB-0419	2	3	1	2	2	pos	NA	0	0	IDC	+	+	-	LumA	8	0		
MB-0420	2	2	0	1	3	neg	1	0	1	IDC	-	-	-	Basal	10	1	c.916C>T	p.R306X
MB-0421	2	2	0	2	3	pos	NA	2	NA	IDC	+	+	+	Her2	5	NA	NA	NA
MB-0422	2	3	1	1	2	pos	NA	0	0	IDC	+	+	-	LumA	8	0		
MB-0423	2	2	0	2	2	pos	NA	0	0	ILC	+	+	-	LumA	3	0		
MB-0424	2	5	1	3	3	neg	1	0	0	IDC	-	-	-	Normal	4	0		
MB-0425	2	4	1	2	2	pos	NA	0	0	IDC	+	+	-	LumA	3	0		
MB-0426	2	4	1	2	2	pos	NA	0	0	IDC	+	+	-	Normal	3	0		
MB-0427	2	2	1	2	2	pos	NA	0	0	ILC	+	-	-	LumA	7	0		
MB-0428	2	3	1	2	3	pos	NA	0	0	IDC	+	-	-	LumA	9	0		
MB-0429	2	4	1	2	3	pos	NA	1	0	ILC	+	+	-	LumB	1	0		
MB-0431	2	4	1	3	2	pos	NA	0	0	ILC	+	-	-	LumA	3	0		
MB-0432	2	2	0	1	1	pos	NA	0	NA	OTHER	-	-	-	Normal	4	NA	NA	NA
MB-0434	2	4	1	2	3	pos	NA	2	0	IDC	+	-	+	LumB	5	0		
MB-0435	2	4	0	1	2	pos	NA	0	0	IDC-MUC	-	-	-	Basal	4	0		
MB-0436	2	3	0	1	3	neg	1	0	1	IDC	-	-	-	Her2	4	1	c.818G>A	p.R273H
MB-0437	2	3	1	1	2	pos	NA	-2	0	IDC	+	+	-	LumB	4	0		
MB-0438	2	1	1	2	3	neg	3	2	0	IDC	-	-	+	Her2	5	0		
MB-0439	2	3	0	1	2	pos	NA	0	0	IDC-MUC	+	+	-	LumB	8	0		
MB-0440	2	2	1	2	3	pos	NA	0	0	IDC	+	-	-	LumB	1	0		
MB-0442	2	4	1	1	2	pos	NA	0	0	IDC	+	-	-	Normal	4	0		
MB-0443	2	2	0	2	2	pos	NA	0	0	IDC	+	+	-	LumA	7	0		
MB-0444	2	3	1	2	2	pos	1	0	1	ILC	+	+	-	LumB	8	1	c.772G>A	p.E258K
MB-0445	2	2	1	2	2	pos	1	0	0	IDC	+	+	-	LumA	7	0		
MB-0446	2	1	0	2	3	neg	1	0	NA	IDC	-	-	-	Basal	10	NA	NA	NA
MB-0448	2	3	0	1	3	pos	NA	1	0	IDC+ILC	+	+	-	LumB	1	0		
MB-0449	2	3	1	2	1	pos	NA	0	0	IDC+ILC	+	+	-	LumA	3	0		
MB-0451	2	2	0	1	1	pos	NA	0	0	IDC	+	-	-	LumA	8	0		
MB-0452	2	3	0	2	3	pos	NA	1	1	IDC	+	-	-	LumB	9	1	c.743G>A	p.R248Q
MB-0453	2	3	1	3	3	pos	1	-2	1	IDC	-	-	-	Her2	2	1	c.493C>T	p.Q165X
MB-0454	2	4	0	2	2	pos	NA	0	0	IDC-MUC	+	+	-	LumA	3	0		
MB-0455	2	4	0	1	2	pos	NA	-1	0	IDC	+	+	-	LumB	10	0		
MB-0458	2	3	1	3	2	pos	1	0	0	IDC+ILC	+	+	-	Normal	4	0		
MB-0459	2	4	1	2	3	pos	NA	1	1	IDC	+	-	-	LumB	6	1	c.488A>G	p.Y163C
MB-0460	2	1	0	2	2	pos	NA	0	0	ILC	+	+	-	LumA	4	0		
MB-0462	2	3	0	1	3	pos	NA	2	0	IDC	-	-	+	Her2	5	0		
MB-0463	2	4	0	2	2	pos	NA	0	0	IDC	+	+	-	LumA	4	0		
MB-0464	2	5	1	1	3	neg	1	-1	1	IDC	-	-	-	Basal	9	1	c.853G>A	p.E285K
MB-0465	2	3	1	2	3	pos	NA	2	1	IDC	+	-	+	LumB	5	1	c.1009C>T	p.R337C
MB-0466	2	4	1	3	2	pos	1	0	0	IDC	+	-	-	LumA	1	0		
MB-0467	2	1	0	1	3	neg	3	2	1	IDC	-	-	+	Her2	5	1	c.268del1	p.?
MB-0468	2	1	1	2	3	pos	1	0	0	IDC	+	-	-	LumA	1	0		
MB-0469	2	3	1	2	3	pos	NA	1	1	IDC	+	+	-	LumB	6	1	c.524G>A	p.R175H
MB-0470	2	4	0	1	1	pos	NA	-1	0	ILC	-	-	-	Basal	4	0		
MB-0471	2	3	0	1	2	pos	NA	0	0	IDC	+	+	-	LumA	1	0		
MB-0474	2	1	1	3	3	pos	1	0	1	IDC+ILC	+	+	-	LumB	9	1	c.734G>T	p.G245V
MB-0475	2	3	0	2	3	pos	NA	0	0	IDC	+	+	-	LumA	9	0		
MB-0476	2	3	0	2	3	neg	3	0	0	IDC	+	-	-	Normal	4	0		
MB-0478	2	2	1	2	2	pos	NA	0	0	IDC	+	+	-	LumA	3	0		
MB-0479	2	1	0	2	3	neg	3	2	0	IDC	-	-	+	Her2	5	0		
MB-0480	2	2	0	1	3	pos	NA	0	1	IDC	+	-	-	LumA	9	1	c.1011_1012del2	p.?
MB-0481	2	4	0	1	3	neg	1	0	1	IDC	-	-	-	Basal	10	1	c.321C>A	p.Y107X
MB-0482	2	4	1	1	3	neg	3	2	0	IDC	-	-	+	Normal	5	0		

MB-0483	2	3	1	2	3	pos	2	0	0	IDC	+	+	-	LumB	1	0		
MB-0484	2	3	1	2	3	pos	3	1	1	IDC	+	+	+	Her2	6	1	c.742C>G	p.R248G
MB-0485	2	4	1	3	3	pos	1	0	0	IDC+ILC	+	-	-	LumA	9	0		
MB-0486	2	4	0	2	3	pos	1	0	0	IDC	+	+	-	LumA	3	0		
MB-0487	2	1	0	1	2	pos	NA	0	0	IDC-TUB	+	+	-	Normal	3	0		
MB-0488	2	3	0	2	1	pos	NA	0	0	IDC-MUC	+	+	-	Basal	4	0		
MB-0489	2	2	0	1	3	neg	3	1	0	IDC	-	-	-	Normal	4	0		
MB-0490	2	2	1	2	2	pos	NA	0	0	IDC	+	+	-	LumA	4	0		
MB-0491	2	3	0	2	2	pos	NA	0	0	ILC	+	+	-	LumA	4	0		
MB-0492	2	3	0	2	3	pos	NA	0	0	IDC	+	+	-	LumB	8	0		
MB-0494	2	4	1	2	3	neg	1	0	1	IDC	-	-	-	Basal	4	1	c.832C>T	p.P278S
MB-0495	2	2	1	1	3	pos	1	0	NA	IDC	-	-	-	Basal	4	NA	NA	NA
MB-0496	2	2	1	2	1	pos	1	0	0	IDC+ILC	+	+	-	Normal	3	0		
MB-0497	2	3	0	2	2	pos	NA	1	0	IDC	+	+	-	LumA	4	0		
MB-0499	2	2	NA	1	2	pos	NA	0	0	IDC	-	-	-	Basal	4	0		
MB-0500	2	5	0	2	3	pos	1	0	1	IDC	-	-	-	Basal	10	1	c.326_327ins1	p.?
MB-0501	2	3	0	2	3	pos	NA	1	1	IDC	+	+	-	LumA	8	1	c.80C>T	p.P27L
MB-0502	2	1	1	2	3	pos	1	0	0	IDC	-	-	-	Basal	4	0		
MB-0503	2	2	0	2	2	pos	NA	1	0	IDC+ILC	+	+	-	LumA	7	0		
MB-0504	2	2	0	2	3	pos	NA	0	0	IDC+ILC	+	+	-	LumA	3	0		
MB-0505	2	2	1	1	1	pos	NA	0	0	IDC	+	+	-	LumA	4	0		
MB-0506	2	3	1	2	3	neg	1	0	1	IDC	-	+	-	Her2	9	1	c.405C>G	p.C135W
MB-0507	2	3	0	1	3	pos	NA	0	0	IDC	+	+	-	LumA	3	0		
MB-0508	2	1	0	1	2	pos	NA	1	0	IDC	+	-	-	Normal	2	0		
MB-0509	2	1	0	1	3	pos	NA	0	0	IDC	+	+	-	Basal	3	0		
MB-0510	2	1	1	3	3	pos	NA	0	0	IDC	+	+	-	Normal	9	0		
MB-0511	2	3	0	2	2	pos	NA	0	0	IDC	+	+	-	LumA	3	0		
MB-0512	2	4	0	2	1	pos	NA	0	0	IDC	+	+	-	LumA	9	0		
MB-0513	2	3	1	2	2	pos	NA	0	0	IDC	+	+	-	LumA	9	0		
MB-0514	2	2	1	2	2	pos	1	0	0	IDC+ILC	+	+	-	LumB	3	0		
MB-0516	2	2	0	1	3	neg	2	0	1	IDC	-	-	-	Basal	10	1	c.524G>A	p.R175H
MB-0517	2	3	0	1	2	pos	NA	-1	0	IDC	+	+	-	LumA	9	0		
MB-0519	2	2	1	3	3	pos	1	-1	0	IDC	+	-	-	Normal	4	0		
MB-0521	2	2	1	2	2	pos	1	0	0	ILC	+	+	-	LumA	3	0		
MB-0522	2	3	0	1	NA	neg	NA	2	NA	DCIS	-	-	+	Her2	5	NA	NA	NA
MB-0524	2	2	0	1	3	pos	NA	0	1	IDC	+	-	-	Normal	4	1	c.824G>A	p.C275Y
MB-0525	2	2	0	2	3	neg	NA	0	1	IDC	-	-	-	Basal	4	1	c.329G>C	p.R110P
MB-0526	2	2	0	1	3	neg	NA	0	0	IDC	+	+	-	LumB	2	0		
MB-0527	2	5	1	1	2	pos	NA	0	0	IDC	+	+	-	LumA	4	0		
MB-0528	2	3	1	2	3	pos	NA	0	1	IDC	+	-	-	LumA	2	1	c.310C>T	p.Q104X
MB-0529	2	2	1	2	2	pos	NA	0	0	IDC	+	+	-	LumA	8	0		
MB-0531	2	2	0	1	3	neg	NA	-1	0	IDC	+	+	-	Normal	4	0		
MB-0532	2	2	1	2	3	neg	1	0	0	IDC	+	+	-	LumB	9	0		
MB-0534	2	2	1	1	3	pos	NA	0	0	IDC	+	+	-	Normal	4	0		
MB-0535	2	1	0	1	2	pos	NA	0	0	IDC	+	+	-	LumA	8	0		
MB-0536	2	1	0	2	3	pos	NA	0	1	IDC	+	-	-	LumB	1	1	c.536A>G	p.H179R
MB-0537	2	5	1	1	1	pos	NA	0	0	IDC	+	-	-	Normal	4	0		
MB-0538	2	2	1	2	3	pos	NA	0	0	IDC	+	+	-	LumA	9	0		
MB-0539	2	2	0	2	NA	NA	NA	2	NA	DCIS	-	-	+	Basal	5	NA	NA	NA
MB-0540	2	1	0	1	1	pos	NA	0	NA	IDC	-	-	-	Normal	4	NA	NA	NA
MB-0541	2	1	0	1	3	pos	NA	0	0	IDC	+	-	-	LumA	9	0		
MB-0542	2	3	1	2	3	pos	1	0	1	IDC	+	+	-	Normal	1	1	c.1011_1017del7	p.?
MB-0543	2	2	1	2	3	pos	NA	2	0	IDC	+	-	-	Basal	1	0		
MB-0544	2	3	1	3	3	pos	NA	0	0	IDC+ILC	+	+	-	LumB	8	0		
MB-0545	2	3	1	2	3	pos	NA	0	1	IDC	+	-	-	Normal	6	1	c.725G>C	p.C242S
MB-0546	2	2	1	1	2	pos	NA	0	NA	IDC	+	-	-	Normal	3	NA	NA	NA
MB-0549	2	3	1	2	3	pos	NA	2	0	IDC	+	-	+	LumB	5	0		
MB-0550	2	2	1	1	2	pos	NA	0	0	IDC	+	-	-	LumB	8	0		
MB-0551	2	2	0	2	2	pos	NA	0	0	IDC+ILC	+	+	-	Normal	4	0		
MB-0553	2	1	1	2	2	pos	NA	2	0	IDC	+	-	+	LumA	5	0		
MB-0554	2	3	0	1	NA	pos	NA	0	0	IDC	+	+	-	LumA	8	0		
MB-0559	2	4	0	2	3	pos	NA	0	0	IDC	+	+	-	LumA	7	0		
MB-0564	2	5	0	2	3	neg	NA	2	0	IDC	-	-	+	Normal	4	0		
MB-0568	2	4	1	1	2	pos	NA	0	0	IDC	+	+	-	LumA	7	0		
MB-0569	2	2	1	2	3	pos	NA	0	0	IDC	+	+	-	LumB	4	0		
MB-0570	2	2	0	2	3	pos	NA	1	1	IDC	+	+	-	LumB	6	1	c.574C>T	p.Q192X
MB-0571	2	2	0	2	1	pos	NA	0	0	IDC	+	+	-	LumA	7	0		
MB-0573	2	NA	0	3	2	pos	NA	-1	NA	OTHER	+	+	-	LumA	7	NA	NA	NA
MB-0574	2	2	1	2	3	pos	NA	0	1	IDC	+	+	-	LumA	2	1	c.707A>G	p.Y236C
MB-0575	2	2	1	3	2	pos	1	1	0	IDC+ILC	+	+	-	LumA	4	0		
MB-0576	2	4	1	2	3	pos	1	0	0	IDC	+	+	-	LumB	2	0		
MB-0577	2	2	0	1	2	pos	NA	0	0	IDC-MUC	+	-	-	LumB	7	0		
MB-0578	2	3	1	2	2	pos	NA	0	0	ILC	+	+	-	Normal	4	0		
MB-0579	2	3	1	2	2	pos	NA	1	0	IDC	+	-	-	LumB	7	0		
MB-0580	2	2	1	2	3	pos	2	1	1	IDC	+	+	-	LumA	4	1	c.641del1	p.?
MB-0581	2	3	1	2	3	neg	NA	0	1	IDC	-	-	-	Basal	7	1	c.581T>G	p.L194R
MB-0582	2	3	0	2	3	neg	NA	0	1	IDC	-	-	-	Basal	10	1	c.452C>A	p.P151H
MB-0583	2	3	0	2	2	pos	NA	0	0	IDC	+	+	-	LumA	3	0		
MB-0584	2	3	0	2	3	pos	NA	0	0	IDC	+	+	-	LumB	7	0		
MB-0585	2	3	1	2	3	pos	NA	0	1	IDC	+	-	-	LumB	6	1	c.524G>A	p.R175H
MB-0586	2	4	1	3	2	pos	NA	0	0	IDC+ILC	+	-	-	LumA	6	0		
MB-0587	2	2	1	2	2	pos	1	2	NA	IDC+ILC	+	+	-	LumA	9	NA	NA	NA
MB-0588	2	3	0	2	2	neg	NA	0	0	IDC	-	-	-	Normal	4	0		
MB-0589	2	3	1	2	2	pos	NA	1	0	IDC	+	-	+	LumB	5	0		
MB-0590	2	4	1	2	3	pos	3	1	1	IDC	+	+	-	LumB	9	1	c.1001G>A	p.G334E
MB-0591	2	4	0	1	3	pos	NA	0	0	IDC	+	-	-	LumB	8	0		
MB-0592	2	2	0	3	NA	pos	NA	0	0	OTHER INVASIVE	+	+	-	LumB	8	0		
MB-0593	2	4	1	3	2	neg	3	2	0	ILC	-	-	+	Her2	5	0		
MB-0594	2	4	1	2	3	pos	NA	-1	0	IDC	+	-	-	LumB	4	0		
MB-0596	2	2	0	1	2	pos	NA	-2	0	IDC+ILC	+	+	-	LumA	3	0		
MB-0597	2	3	1	1	2	pos	NA	0	0	IDC	+	+	-	Normal	3	0		
MB-0598	2	3	1	2	2	pos	NA	0	0	IDC	+	+	-	LumB	3	0		
MB-0599	2	3	1	2	2	pos	1	0	0	IDC	+	-	-	LumA	4	0		
MB-0600	2	3	1	2	3	pos	NA	0	NA	OTHER	+	-	-	LumA	6	NA	NA	NA
MB-0601	2	3	1	2	3	pos	1	0	1	IDC	+	-	-	LumB	10	1	c.377A>G	p.Y126C
MB-0603	2	3	1	2	2	pos	NA	0	0	IDC	+	+	-	LumA	7	0		
MB-0605	2	4	0	1	2	pos	NA	2	0	IDC+ILC	+	+	-	LumA	8	0		
MB-0606	2	4	0	1	2	pos	NA	0	0	IDC-MUC	+	-	-	LumB	3	0		
MB-0607	2	2	1	2	3	pos	3	1	0	IDC	+	+	-	LumA	4	0		
MB-0608	2	3	1	2	3	neg	1	0	1	IDC-MED	-	-	-	Basal	10	1	c.524G>A	p.R175H
MB-0609	2	3	1	1	1	pos	1	0	1	IDC	+	-	-	LumA	8	1	c.747G>T	p.R249S
MB-0611	2	3	0	2	3	pos	NA	2	1	IDC	+	+	-	LumB	9	1	c.817C>T	p.R273C
MB-0613	2	5	0	3	2	neg	1	0	1	IDC-MED	-	-	-	Basal	4	1	c.859G>T	p.E287X
MB-0614	2	4	1	1	3	pos	1	0	0	IDC	+	+	-	LumA	6	0		
MB-0615	2	3	1	1	2	neg	3	2	1	IDC	-	-	+	Normal	5	1	c.701A>G	p.Y234C
MB-0616	2	2	1	2	3	pos	NA	0	0	IDC+ILC	+	-	-	LumB	7	0		
MB-0617	2	5	0	2	1	pos	NA	0	0	IDC-MUC	-	-	-	Normal	4	0		
MB-0618	2	2	0	1	2	pos	NA	0	0	IDC	+	+	-	LumB	3	0		
MB-0619	2	1	1	2	3	pos	1	0	0	IDC-MUC	+	-	-	Normal	4	0		
MB-0620	2	2	1	1	3	pos	1	0	NA	OTHER INVASIVE	+	-	-	Normal	4	NA	NA	NA
MB-0621	2	4	1	2	2	pos	NA	0	0	ILC	+	+	-	LumA	4	0		
MB-0623	2	3	0	1	2	pos	NA	0	0	IDC	+	+	-	Normal	4	0		
MB-0624	2	5	1	1	2	pos	NA	-2	0	IDC	+	-	-	LumA	3	0		
MB-0626	2	2	0	2	3	pos	NA	0	1	IDC	+	-	-	LumB	1	1	c.400T>C	p.F134L
MB-0627	2	2	NA	2	3	neg	2	0	0	IDC	-	-	-	Basal	4	0		
MB-0628	2	4	0	1	2	pos	NA	0	0	IDC	+	-	-	Normal	7	0		
MB-0630	2	4	0	2	3	pos	NA	1	0	IDC	+	+	+	LumB	1	0		
MB-0631	2	4	1	2	2	pos	NA	0	0	IDC	+	+	-	LumA	8	0		
MB-0632	2	3	0	2	2	pos	NA	0	0	IDC	+	-	-	LumA	4	0		
MB-0634	2	3	NA	1	2	pos	NA	0	0	ILC	-	-	-	Normal	4	0		
MB-0635	2	2	0	1	3	pos	NA	0	0	IDC	-	-	-	Basal	4	0		
MB-0636	2	2	0	1	3	pos	1	0	1	IDC	+	+	-	LumA	6	1	c.818G>A	p.R273H
MB-0637	2	4	0	2	3	pos	NA	0	1	IDC	+	+	-	LumB	10	1	c.815T>G	p.V272G
MB-0638	2	3	1	2	2	pos	NA	0	0	IDC	+	-	-	Normal	4	0		
MB-0639	2	2	0	1	3	neg	1	0	1	IDC	-	-	-	Basal	10	1	c.743G>A	p.R248Q
MB-0640	2	1	0	1	2	pos	NA	0	0	IDC	+	+	-	Normal	7	0		
MB-0641	2	3	1	1	2	pos	NA	0	0	IDC	+	-	-	Normal	4	0		
MB-0642	2	1	0	2	1	pos	NA	0	0	IDC	+	+	-	LumA	8	0		
MB-0643	2	5	1	3	3	pos	NA	2	1	IDC	-	-	+	Her2	5	1	c.911_912ins2	p.?
MB-0644	2	4	1	2	2	pos	NA	0	0	IDC	+	+	-	LumA	4	0		
MB-0646	2	3	1	2	2	pos	1	0	0	IDC+ILC	+	-	-	LumB	8	0		
MB-0649	2	1	1	1	1	pos	NA	0	0	IDC	+	+	-	LumA	7	0		
MB-0650	2	3	1	2	2	pos	1	0	0	IDC	+	-	-	LumA	2	0		
MB-0652	2	3	1	2	3	pos	NA	1	1	IDC	+	+	+	Her2	5	1	c.524G>A	p.R175H
MB-0653	2	5	1	2	3	pos	NA	0	1	IDC+ILC	-	-	-	Her2	9	1	c.853G>A	p.E285L
MB-0654	2	4	0	2	1	pos	NA	0	0	IDC	+	+	-	LumA	8	0		
MB-0655	2	4	0	3	NA	pos	NA	0	0	OTHER INVASIVE	+	+	-	Normal	4	0		
MB-0656	2	5	0	3	3	neg	3	2	0	IDC	-	-	+	Basal	5	0	c.648G>T	p.V216V
MB-0657	2	5	0	3	2	pos	NA	0	0	ILC	+	-	-	Normal	4	0		
MB-0658	2	3	1	2	3	neg	2	-1	1	IDC	-	-	-	Basal	10	1	c.743G>A	p.R248Q
MB-0659	2	3	0	2	2	neg	NA	0	NA	OTHER	-	-	-	Normal	4	NA	NA	NA
MB-0660	2	3	1	3	3	pos	1	1	1	IDC-MED	-	-	-	Her2	10	1	c.422G>A	p.C141Y
MB-0661	2	3	0	1	2	pos	NA	1	0	IDC	+	+	-	LumA	7	0		
MB-0662	2	3	0	1	3	pos	NA	2	0	IDC	+	+	+	Normal	5	0		

MB-0663	2	4	0	1	3	neg	3	2	1	IDC	-	-	+	Her2	5	1	c.714del1	p.?
MB-0664	2	5	1	3	3	neg	NA	0	1	IDC	-	-	-	Basal	10	1	c.812del1	p.?
MB-0666	2	2	1	2	3	pos	1	0	0	IDC	+	+	-	LumA	9	0		
MB-0667	2	3	1	2	3	pos	NA	2	0	IDC	+	+	+	LumB	1	0		
MB-0869	2	1	0	2	3	neg	NA	0	1	IDC	-	-	-	Basal	10	1	c.637C>T	p.R213X
MB-0872	2	2	1	1	2	pos	NA	0	0	IDC	+	+	-	Normal	7	0		
MB-0874	2	3	0	2	3	neg	NA	0	1	IDC	-	-	-	Basal	10	1	c.839G>C	p.R280T
MB-0876	2	3	0	1	NA	NA	NA	0	NA	DCIS	-	-	-	Basal	4	NA	NA	NA
MB-0877	2	5	1	2	3	pos	NA	0	1	IDC	+	+	-	LumB	6	1	c.743G>A	p.R248Q
MB-0880	2	2	0	2	1	pos	NA	0	0	IDC	+	+	-	LumA	7	0		
MB-0882	2	3	0	2	3	pos	NA	0	1	IDC+ILC	+	+	-	LumB	7	1	c.637C>T	p.R213X
MB-0884	2	4	1	2	3	pos	1	0	1	IDC	+	-	-	LumB	9	1	c.651_652ins23	p.?
MB-0885	2	2	0	1	NA	NA	NA	0	NA	PHYL	-	-	-	Normal	4	NA	NA	NA
MB-0891	2	5	1	2	1	pos	NA	0	0	IDC-TUB	+	-	-	LumA	3	0		
MB-0893	2	2	1	2	3	pos	NA	0	0	IDC	-	-	-	Basal	4	0		
MB-0895	2	4	1	1	3	pos	NA	2	1	IDC	+	+	+	LumB	5	1	c.730G>A	p.G244S
MB-0897	2	3	1	2	2	NA	NA	0	0	IDC+ILC	+	+	-	Normal	4	0		
MB-0899	2	2	1	2	2	pos	NA	0	0	IDC	+	+	-	LumA	8	0		
MB-0901	2	3	1	2	3	neg	NA	0	1	IDC	-	-	-	Basal	10	1	c.390_427del37	p.?
MB-0904	2	5	1	2	2	pos	NA	0	0	IDC	+	+	-	LumA	3	0		
MB-0906	2	4	0	2	3	neg	NA	0	0	IDC	-	-	-	Normal	4	0		
MB-0907	2	3	0	2	NA	NA	NA	2	NA	DCIS	-	-	+	Normal	4	NA	NA	NA
