# Supplementary material for: Microenvironmental Heterogeneity Parallels Breast Cancer Progression: A Histology–Genomic Integration Analysis
Source: PLoS Med. 2016 Feb 16;13(2):e1001961. doi: 10.1371/journal.pmed.1001961 (PMC4755617; doi:10.1371/journal.pmed.1001961)
Supplement: S2 Table — (PDF) [file pmed.1001961.s014.pdf]

Supplementary Table 2: Copy number alterations significantly enriched in EDI-high, grade 3 breast tumors.

Symbol	Symbol2	Cytoband	npval	gain	loss	CN.and.z	description
UBE2K	HIP2	4p14b	7.20E-05	2	27	13	ubiquitin-conjugating enzyme E2K
PDS5A	PDS5A	4p14b	0.00012	2	28	13	PDS5, regulator of cohesion maintenance, homolog A (S. cerevisiae)
LIAS	LIAS	4p14b	0.00012	2	28	13	lipoic acid synthetase
UGDH	UGDH	4p14b	0.00012	2	28	13	UDP-glucose 6-dehydrogenase
C4orf34	C4orf34	4p14b	0.00012	2	28	13	chromosome 4 open reading frame 34
GPR17	GPR17	2q14.3e	2.00E-04	12	6	9	G protein-coupled receptor 17
LIMS2	LIMS2	2q14.3e	2.00E-04	12	6	9	LIM and senescent cell antigen-like domains 2
MYO7B	MYO7B	2q14.3d-q14	2.00E-04	12	6	9	myosin VIIB
hCG_17324	LOC654134		0.00029	6	4	6	
TMEM177	TMEM177	2q14.2c	0.00029	6	4	6	transmembrane protein 177
CMPK1	CMPK1	1p33d	0.00031	20	16	14	cytidine monophosphate (UMP-CMP) kinase 1, cytosolic
DKFZp313J15	LOC651436		0.00035	2	27	12	
KLB	KLB	4p14c-p14b	0.00035	2	27	12	klotho beta
RPL9	RPL9	4p14b	0.00035	2	27	12	ribosomal protein L9
	LOC647859	5q13.2b	0.00036	7	41	17	occludin pseudogene
OCLN	OCLN	5q13.2a	0.00036	7	41	17	occludin
IWS1	IWS1	2q14.3d	0.00037	8	5	7	IWS1 homolog (S. cerevisiae)
PROC	PROC	2q14.3d	0.00037	8	5	7	protein C (inactivator of coagulation factors Va and VIIIa)
MAP3K2	LOC651319		0.00037	8	5	7	mitogen-activated protein kinase kinase kinase 2
LYPD6B	LOC130576	2q23.2a	0.00037	7	6	7	LY6/PLAUR domain containing 6B
BC035647	LOC642049		0.00046	42	15	19	
LOC344967	LOC344967	4p14b	0.00053	1	29	12	acyl-CoA thioesterase 7 pseudogene
C17orf71	C17orf71	17q22d	0.00064	96	17	31	
GDPD1	GDPD1	17q22d	0.00066	101	17	32	glycerophosphodiester phosphodiesterase domain containing 1
SMA4	LOC643373	5p13.3a	0.00066	7	35	15	glucuronidase, beta pseudogene
GTF2H2D	LOC728340	5q13.2a	0.00066	7	35	15	general transcription factor IIH, polypeptide 2D
AL049987	LOC728499	5q13.2a	0.00066	7	35	15	POM121 membrane glycoprotein (rat) pseudogene
BC064850	LOC728452	5q13.2b	0.00066	7	35	15	POM121 membrane glycoprotein (rat) pseudogene
GTF2H2	LOC653238	5q13.2a	0.00066	7	35	15	general transcription factor IIH, polypeptide 2, 44kDa
GTF2H2C	LOC653866		0.00066	7	35	15	general transcription factor IIH, polypeptide 2C
DKFZp686P0	LOC653489	2q12.2b	0.00069	5	6	6	
CNTNAP5	CNTNAP5	2q14.3b-q14	0.00069	5	6	6	contactin associated protein-like 5
ARL5A	ARL5A	2q23.3c	0.00069	5	6	6	ADP-ribosylation factor-like 5A
INSIG2	INSIG2	2q14.1d	0.00074	3	3	4	insulin induced gene 2
CCDC93	FLJ10996	2q14.1d	0.00074	3	3	4	coiled-coil domain containing 93
KIAA0531	KIF5C	2q23.1b	0.00075	8	6	7	kinesin family member 5C
LYPD6	LYPD6	2q23.2a	0.00075	7	7	7	LY6/PLAUR domain containing 6
RFC1	RFC1	4p14c	0.00078	3	28	12	replication factor C (activator 1) 1, 145kDa
WDR19	WDR19	4p14c	0.00078	3	28	12	WD repeat domain 19
BRWD1	BRWD1	21q22.2a-q2.	0.00089	32	7	14	bromodomain and WD repeat domain containing 1
PCTP	PCTP	17q22b	0.00091	89	2	26	phosphatidylcholine transfer protein
AKAP1	AKAP1	17q22c	0.0011	101	5	29	A kinase (PRKA) anchor protein 1
DHX40	DHX40	17q23.1a	0.0011	111	10	32	DEAH (Asp-Glu-Ala-His) box polypeptide 40
BCAS3	BCAS3	17q23.2a-q2.	0.0011	125	6	34	breast carcinoma amplified sequence 3
RPS6KB1	RPS6KB1	17q23.1a	0.0011	117	9	33	ribosomal protein S6 kinase, 70kDa, polypeptide 1
RNFT1	LOC645294	17p11.2c	0.0011	116	10	33	ring finger protein, transmembrane 1
TUBD1	TUBD1	17q23.1a	0.0011	116	10	33	tubulin, delta 1
EDIL3	EDIL3	5q14.3a	0.0011	17	35	17	EGF-like repeats and discoidin I-like domains 3
RPL23AP12	LOC391282	21q22.2a	0.0012	29	7	13	ribosomal protein L23a pseudogene 12
FLJ45139	FLJ45139	21q22.2a	0.0012	29	7	13	FLJ45139 protein
DNAJC27	DNAJC27	2p23.3c	0.0012	25	11	13	DnaJ (Hsp40) homolog, subfamily C, member 27
MBOAT2	MBOAT2	2p25.1e	0.0012	31	5	13	membrane bound O-acyltransferase domain containing 2
CCNY	CCNY	10p11.21c	0.0012	35	9	15	cyclin Y
ACTBL3	LOC646855		0.0012	12	6	8	
CHST10	CHST10	2q11.2d	0.0012	10	8	8	carbohydrate sulfotransferase 10
C17orf67	LOC650311		0.0012	98	4	28	chromosome 17 open reading frame 67
DGKE	DGKE	17q22c	0.0012	98	4	28	diacylglycerol kinase, epsilon 64kDa
FOXE3	FOXE3	1p33d	0.0012	24	16	14	forkhead box E3
C2orf76	C2orf76	2q14.2b	0.0013	6	3	5	chromosome 2 open reading frame 76
EN1	EN1	2q14.2b	0.0013	7	2	5	engrailed homeobox 1
AFF3	AFF3	2q11.2c-q11.	0.0014	10	5	7	AF4/FMR2 family, member 3
REV1	REV1	2q11.2c	0.0014	10	5	7	REV1 homolog (S. cerevisiae)
EIF5B	EIF5B	2q11.2c	0.0014	10	5	7	eukaryotic translation initiation factor 5B
RAPGEF4	RAPGEF4	2q31.1e	0.0014	11	4	7	Rap guanine nucleotide exchange factor (GEF) 4
KIF5C	KIF5C	2q23.1b	0.0014	9	6	7	kinesin family member 5C
CGEF2	LOC643900		0.0014	11	4	7	
RBMS1	RBMS1	2q24.2b	0.0014	10	5	7	RNA binding motif, single stranded interacting protein 1
PTPN4	PTPN4	2q14.2c	0.0014	8	4	6	protein tyrosine phosphatase, non-receptor type 4 (megakaryocyte)
ERCC3	ERCC3	2q14.3d	0.0014	7	5	6	excision repair cross-complementing rodent repair deficiency, complementation group 3
ANAPC1	LOC652635		0.0014	6	6	6	anaphase promoting complex subunit 1
CACNB4	CACNB4	2q23.3c	0.0014	5	7	6	calcium channel, voltage-dependent, beta 4 subunit
RNF126P1	RNF126P1	17q22c	0.0015	99	4	28	ring finger protein 126 pseudogene 1
SCPEP1	SCPEP1	17q22c	0.0015	99	4	28	serine carboxypeptidase 1
DMRTA2	DMRTA2	1p33a	0.0016	28	21	16	DMRT-like family A2
CYP2J2	CYP2J2	1p32.1c	0.0016	30	19	16	cytochrome P450, family 2, subfamily J, polypeptide 2
FAF1	FAF1	1p33a	0.0016	28	21	16	Fas (TNFRSF6) associated factor 1
TMEM49	TMEM49	17q23.1a	0.0016	118	10	33	
DYNC2LI1	DYNC2LI1	2p21d	0.0016	17	5	9	dynein, cytoplasmic 2, light intermediate chain 1
PLEKHH2	PLEKHH2	2p21d	0.0016	17	5	9	pleckstrin homology domain containing, family H (with MyTH4 domain) member 2
HLF	HLF	17q22b	0.0016	89	5	26	hepatic leukemia factor
SH3BGR	SH3BGR	21q22.2b	0.0017	28	13	14	SH3 domain binding glutamic acid-rich protein
TRIM25	TRIM25	17q22c	0.0018	100	4	28	tripartite motif containing 25
ACSF2	FLJ20920	17q21.33b	0.0018	103	6	29	acyl-CoA synthetase family member 2
CHAD	CHAD	17q21.33b	0.0018	103	6	29	chondroadherin
RSAD1	RSAD1	17q21.33b	0.0018	103	6	29	radical S-adenosyl methionine domain containing 1
KIAA0622	CLASP1	2q14.2e-q14	0.002	12	7	8	cytoplasmic linker associated protein 1
HOOK1	HOOK1	1p32.1c	0.002	30	20	16	hook homolog 1 (Drosophila)
C1orf87	C1orf87	1p32.1b	0.002	32	18	16	chromosome 1 open reading frame 87
IDE	IDE	10q23.33a	0.0021	3	27	11	insulin-degrading enzyme
FGGY	FLJ10986	1p32.1d-p32	0.0021	29	17	15	FGGY carbohydrate kinase domain containing
TBX2	TBX2	17q23.2b	0.0022	124	6	33	T-box 2
DEFB131	DEFB131	4p16.1c	0.0022	17	25	14	defensin, beta 131
DDX18	DDX18	2q14.1d	0.0022	3	4	4	DEAD (Asp-Glu-Ala-Asp) box polypeptide 18
PSMG1	PSMG1	21q22.2a	0.0022	31	7	13	proteasome (prosome, macropain) assembly chaperone 1
ETS2	ETS2	21q22.2a	0.0022	30	8	13	v-ets erythroblastosis virus E26 oncogene homolog 2 (avian)
CIB4	CIB4	2p23.3b	0.0022	29	9	13	calcium and integrin binding family member 4
TMEM100	TMEM100	17q22b	0.0023	89	2	25	transmembrane protein 100

HS6ST1	HS6ST1	2q14.3e	0.0024	11	5	7	heparan sulfate 6-O-sulfotransferase 1
WDR33	WDR33	2q14.3e	0.0024	9	7	7	WD repeat domain 33
SFT2D3	SFT2D3	2q14.3e	0.0024	11	5	7	SFT2 domain containing 3
NMS	NMS	2q11.2d	0.0024	9	7	7	neuromedin S
MBD5	MBD5	2q23.1a	0.0024	9	7	7	methyl-CpG binding domain protein 5
ADARB1	ADARB1	21q22.3e	0.0024	50	5	17	adenosine deaminase, RNA-specific, B1
KRTAP10-9	KRTAP10-9	21q22.3d	0.0024	48	7	17	keratin associated protein 10-9
PPM1E	PPM1E	17q22d	0.0025	88	18	28	protein phosphatase, Mg2+/Mn2+ dependent, 1E
TRIM37	TRIM37	17q22d	0.0025	88	18	28	tripartite motif containing 37
DLX4	DLX4	17q21.33a	0.0025	100	6	28	distal-less homeobox 4
PRR11	PRR11	17q22d	0.0025	94	17	29	proline rich 11
MITD1	MITD1	2q11.2c	0.0027	8	5	6	MIT, microtubule interacting and transport, domain containing 1
MRPL30	MRPL30	2q11.2c	0.0027	8	5	6	mitochondrial ribosomal protein L30
EPB41L5	EPB41L5	2q14.2c	0.0027	9	4	6	erythrocyte membrane protein band 4.1 like 5
TGFBRAP1	LOC644629		0.0027	7	6	6	transforming growth factor, beta receptor associated protein 1
FHL2	FHL2	2q12.2a	0.0027	7	6	6	four and a half LIM domains 2
C2orf49	C2orf49	2q12.2a	0.0027	7	6	6	chromosome 2 open reading frame 49
TXNDC9	LOC285453	4q12e	0.0027	8	5	6	thioredoxin domain containing 9
BCL2L11	BCL2L11	2q13c	0.0027	7	6	6	BCL2-like 11 (apoptosis facilitator)
RND3	RND3	2q23.3a	0.0027	5	8	6	Rho family GTPase 3
BX119026	LOC339782		0.0027	5	8	6	
MAP2K6	MAP2K6	17q24.3a	0.0027	87	5	25	mitogen-activated protein kinase kinase 6
DBI	DBI	2q14.2b	0.0027	7	3	5	diazepam binding inhibitor (GABA receptor modulator, acyl-CoA binding protein)
GYPC	GYPC	2q14.3d	0.0027	6	4	5	glycophorin C (Gerbich blood group)
PLGLA	PLGLA	2q12.2b	0.0027	4	6	5	plasminogen-like A
CYP27C1	CYP27C1	2q14.3d	0.0027	6	4	5	cytochrome P450, family 27, subfamily C, polypeptide 1
NPEPPS	NPEPPS	17q21.32b	0.0028	35	25	18	aminopeptidase puromycin sensitive
MMD	MMD	17q22b	0.0028	91	6	26	monocyte to macrophage differentiation-associated
COIL	COIL	17q22c	0.0029	98	4	27	coilin
AKIRIN1	AKIRIN1	1p34.3a	0.0029	22	21	14	akirin 1
ELAVL4	ELAVL4	1p33a	0.0029	27	16	14	ELAV (embryonic lethal, abnormal vision, Drosophila)-like 4 (Hu antigen D)
ADCY3	ADCY3	2p23.3c	0.0029	32	11	14	adenylate cyclase 3
CENPO	CENPO	2p23.3c	0.0029	32	11	14	centromere protein O
DLX3	DLX3	17q21.33a	0.0029	101	6	28	distal-less homeobox 3
DKFZp762C1	UBE2Q2	15q24.2a	0.0029	12	19	11	ubiquitin-conjugating enzyme E2Q family member 2
RPL7A	LOC647949		0.0029	13	18	11	ribosomal protein L7a
SERTAD2	SERTAD2	2p14c	0.0029	26	5	11	SERTA domain containing 2
PCDH7	PCDH7	4p15.1e-p15	0.003	2	37	13	protocadherin 7
C2orf70	LOC339778	2p23.3b	0.003	30	9	13	chromosome 2 open reading frame 70
LRIG2	LRIG2	1p13.2c	0.003	14	21	12	leucine-rich repeats and immunoglobulin-like domains 2
DQ894659	TAF1B	2p25.1d	0.003	30	5	12	TATA box binding protein (TBP)-associated factor, RNA polymerase I, B, 63kDa
ISL2	ISL2	15q24.3a	0.003	11	24	12	ISL LIM homeobox 2
KIAA1454	SCAPER	15q24.3a	0.003	11	24	12	S-phase cyclin A-associated protein in the ER
TAF1B	TAF1B	2p25.1d	0.003	30	5	12	TATA box binding protein (TBP)-associated factor, RNA polymerase I, B, 63kDa
RSAD2	RSAD2	2p25.2a	0.003	30	5	12	radical S-adenosyl methionine domain containing 2
CMPK2	CMPK2	2p25.2a	0.003	30	5	12	cytidine monophosphate (UMP-CMP) kinase 2, mitochondrial
LONRF2	LONRF2	2q11.2d	0.0031	15	5	8	LON peptidase N-terminal domain and ring finger 2
BX648246	LOC648293		0.0031	15	5	8	
BC037862	LOC648293		0.0031	15	5	8	
FIGN	FIGN	2q24.3a	0.0031	11	9	8	fidgetin
TRIML2	TRIML2	4q35.2b	0.0033	3	49	16	tripartite motif family-like 2
TRIML1	TRIML1	4q35.2c	0.0033	3	49	16	tripartite motif family-like 1
VEZF1	VEZF1	17q22d	0.0034	99	4	27	vascular endothelial zinc finger 1
AK097513	FLJ40194	17q21.32c	0.0034	91	12	27	uncharacterized FLJ40194
TAC4	TAC4	17q21.33a	0.0035	101	7	28	tachykinin 4 (hemokinin)
COL1A1	COL1A1	17q21.33a	0.0035	102	6	28	collagen, type I, alpha 1
LRRC59	LRRC59	17q21.33b	0.0035	103	5	28	leucine rich repeat containing 59
EME1	EME1	17q21.33b	0.0035	103	5	28	essential meiotic endonuclease 1 homolog 1 (S. pombe)
CARD11	CARD11	7p22.2b	0.0035	52	9	18	caspase recruitment domain family, member 11
PTTG1IP	PTTG1IP	21q22.3d-q2	0.0035	56	5	18	pituitary tumor-transforming 1 interacting protein
YPEL2	YPEL2	17q22d	0.0035	111	12	31	yippee-like 2 (Drosophila)
PCDHA6	PCDHA8	5q31.3b	0.0036	12	36	15	protocadherin alpha 6
ID2	ID2	2p25.1e	0.0038	39	5	14	inhibitor of DNA binding 2, dominant negative helix-loop-helix protein
DPP10	DPP10	2q14.1a-q14	0.0038	6	11	7	dipeptidyl-peptidase 10 (non-functional)
LOC151121	LOC151121	2q21.1a	0.0038	12	5	7	uncharacterized LOC151121
SP3	SP3	2q31.1f	0.0038	13	4	7	Sp3 transcription factor
EPC2	EPC2	2q23.1b	0.0038	7	10	7	enhancer of polycomb homolog 2 (Drosophila)
GRB14	GRB14	2q24.3b	0.0038	9	8	7	growth factor receptor-bound protein 14
COBLL1	COBLL1	2q24.3b	0.0038	9	8	7	COBL-like 1
USP1	USP1	1p31.3d	0.0038	32	25	17	ubiquitin specific peptidase 1
NME1	NME2	17q21.33b	0.0038	87	7	25	non-metastatic cells 1, protein (NM23A) expressed in
MMRN2	MMRN2	10q23.2a	0.0039	11	17	10	multimerin 2
KIF11	KIF11	10q23.33a	0.0039	3	25	10	kinesin family member 11
CSGALNACT2	CSGALNACT2	10q11.21a	0.0039	34	6	13	chondroitin sulfate N-acetylgalactosaminyltransferase 2
C17orf82	C17orf82	17q23.2b	0.004	123	6	32	chromosome 17 open reading frame 82
SFRS1	SFRS1	17q22d	0.004	100	4	27	
STIL	STIL	1p33d	0.004	21	15	12	SCL/TAL1 interrupting locus
OTOF	OTOF	2p23.3b	0.004	23	9	11	otoferlin
BMPR1A	BMPR1A	10q23.2a	0.004	15	17	11	bone morphogenetic protein receptor, type IA
GUF1	GUF1	4p13b	0.004	7	25	11	GUF1 GTPase homolog (S. cerevisiae)
GNPDA2	GNPDA2	4p13b	0.004	7	25	11	glucosamine-6-phosphate deaminase 2
YIPF7	YIPF7	4p13b	0.004	7	25	11	Yip1 domain family, member 7
PTRH2	PTRH2	17q23.1a	0.0041	114	10	31	peptidyl-tRNA hydrolase 2
CLTC	CLTC	17q23.1a	0.0041	114	10	31	clathrin, heavy chain (Hc)
FAM33A	FAM33A	17q22d	0.0041	92	17	28	
ITGA3	ITGA3	17q21.33a	0.0041	103	6	28	integrin, alpha 3 (antigen CD49C, alpha 3 subunit of VLA-3 receptor)
XYLT2	XYLT2	17q21.33b	0.0041	104	5	28	xylosyltransferase II
MRPL27	MRPL27	17q21.33b	0.0041	104	5	28	mitochondrial ribosomal protein L27
ORC1L	ORC1L	1p32.3d	0.0042	27	26	16	
CC2D1B	CC2D1B	1p32.3d	0.0042	27	26	16	coiled-coil and C2 domain containing 1B
PRPF38A	PRPF38A	1p32.3d	0.0042	27	26	16	PRP38 pre-mRNA processing factor 38 (yeast) domain containing A
ZCCHC11	ZCCHC11	1p32.3d	0.0042	27	26	16	zinc finger, CCHC domain containing 11
MYO10	MYO10	5p15.1b	0.0043	54	8	18	myosin X
UBE2G2	UBE2G2	21q22.3d	0.0043	55	7	18	ubiquitin-conjugating enzyme E2G 2
STXBP4	STXBP4	17q22b	0.0044	84	6	24	syntaxin binding protein 4
UTP18	UTP18	17q21.33c	0.0044	84	6	24	UTP18 small subunit (SSU) processome component homolog (yeast)
TXNDC12	TXNDC12	1p32.3e-p32	0.0045	23	26	15	thioredoxin domain containing 12 (endoplasmic reticulum)
RET	RET	10q11.21a	0.0045	41	8	15	ret proto-oncogene
TSGA10	TSGA10	2q11.2c	0.0046	9	5	6	testis specific, 10
DQ895307	TSGA10	2q11.2c	0.0046	9	5	6	

LYG2	LYG2	2q11.2c	0.0046	9	5	6	lysozyme G-like 2
LYG1	LYG1	2q11.2c	0.0046	9	5	6	lysozyme G-like 1
ZAK	ZAK	2q31.1e	0.0046	10	4	6	sterile alpha motif and leucine zipper containing kinase AZK
MMADHC	C2orf25	2q23.2a	0.0046	6	8	6	methylmalonic aciduria (cobalamin deficiency) cblD type, with homocystinuria
ABCG8	ABCG8	2p21d	0.0047	16	5	8	ATP-binding cassette, sub-family G (WHITE), member 8
LRPPRC	LRPPRC	2p21d	0.0047	16	5	8	leucine-rich pentatricopeptide repeat containing
ABCG5	ABCG5	2p21d	0.0047	16	5	8	ATP-binding cassette, sub-family G (WHITE), member 5
MYOF	FER1L3	10q23.33a-q	0.0047	4	17	8	myoferlin
CCDC74A	CCDC74B	2q21.1b	0.0047	15	6	8	coiled-coil domain containing 74A
IFIH1	IFIH1	2q24.2d	0.0047	13	8	8	interferon induced with helicase C domain 1
KCNH7	KCNH7	2q24.2d	0.0047	12	9	8	potassium voltage-gated channel, subfamily H (eag-related), member 7
ZNF652	ZNF652	17q21.32c	0.0047	94	11	27	zinc finger protein 652
SGCA	SGCA	17q21.33a	0.0048	104	6	28	sarcoglycan, alpha (50kDa dystrophin-associated glycoprotein)
HILS1	HILS1	17q21.33a	0.0048	104	6	28	histone linker H1 domain, spermatid-specific 1, pseudogene
PDK2	PDK2	17q21.33a	0.0048	104	6	28	pyruvate dehydrogenase kinase, isozyme 2
PPP1R9B	PPP1R9B	17q21.33a	0.0048	104	6	28	protein phosphatase 1, regulatory subunit 9B
SAMD14	SAMD14	17q21.33a	0.0048	104	6	28	sterile alpha motif domain containing 14
AMAP-1	MYCBPAP	17q21.33b	0.0048	104	6	28	MYCBP associated protein
EIF4G3	EIF4G3	1p36.12b	0.0048	2	43	14	eukaryotic translation initiation factor 4 gamma, 3
DR1	DR1	1p22.1b	0.0048	14	31	14	down-regulator of transcription 1, TBP-binding (negative cofactor 2)
LOC1001285	LOC649813		0.0051	82	4	23	uncharacterized LOC100128571
LCA5L	LCA5L	21q22.2b	0.0051	30	11	13	Leber congenital amaurosis 5-like
WRB	WRB	21q22.2b	0.0051	30	11	13	tryptophan rich basic protein
BG000842	LOC653188	5q13.2a	0.0051	5	36	13	
MARCO	MARCO	2q14.2b	0.0051	6	2	4	macrophage receptor with collagenous structure
ANKRD57	ANKRD57	2q13a	0.0052	7	4	5	
SLC5A7	SLC5A7	2q12.3c	0.0052	4	7	5	solute carrier family 5 (choline transporter), member 7
Sep-10	Sep-10	2q13a	0.0052	7	4	5	septin 10
TEKT4	TEKT4	2q11.1c	0.0052	9	2	5	tektin 4
POU3F3	POU3F3	2q12.1d	0.0052	5	6	5	POU class 3 homeobox 3
STAM2	STAM2	2q23.3c	0.0052	4	7	5	signal transducing adaptor molecule (SH3 domain and ITAM motif) 2
ATP8A1	ATP8A1	4p13c	0.0052	2	23	9	ATPase, aminophospholipid transporter (APLT), class I, type 8A, member 1
ABCA5	ABCA5	17q24.3a	0.0052	83	8	24	ATP-binding cassette, sub-family A (ABC1), member 5
KRTAP10-8	KRTAP10-8	21q22.3d	0.0052	47	7	16	keratin associated protein 10-8
SLC1A7	SLC1A7	1p32.3c	0.0052	38	16	16	solute carrier family 1 (glutamate transporter), member 7
PODN	PODN	1p32.3c	0.0052	38	16	16	podocan
KRTAP12-3	KRTAP12-3	21q22.3d	0.0052	47	7	16	keratin associated protein 12-3
KRTAP12-2	KRTAP12-2	21q22.3d	0.0052	47	7	16	keratin associated protein 12-2
KRTAP10-10	KRTAP10-10	21q22.3d	0.0052	47	7	16	keratin associated protein 10-10
KRTAP10-11	KRTAP10-11	21q22.3d	0.0052	47	7	16	keratin associated protein 10-11
KRTAP12-4	KRTAP12-4	21q22.3d	0.0052	47	7	16	keratin associated protein 12-4
KRTAP12-1	KRTAP12-1	21q22.3d	0.0052	47	7	16	keratin associated protein 12-1
SPAG9	SPAG9	17q21.33b	0.0054	91	5	25	sperm associated antigen 9
C10orf116	C10orf116	10q23.2a	0.0054	12	17	10	chromosome 10 open reading frame 116
EU176715	SNCG	10q23.2a	0.0054	12	17	10	synuclein, gamma (breast cancer-specific protein 1)
FAM25A	KIAA1975	10q23.2a-q2	0.0054	12	17	10	family with sequence similarity 25, member A
KIAA1975	LOC643150		0.0054	12	17	10	
PPP2CA	CDKL3	5q31.1d	0.0054	12	21	11	protein phosphatase 2, catalytic subunit, alpha isozyme
BCAS2	BCAS2	1p13.2a	0.0054	15	18	11	breast carcinoma amplified sequence 2
DENND2C	DENND2C	1p13.2a	0.0054	15	18	11	DENN/MADD domain containing 2C
C2orf39	C2orf39	2p23.3b	0.0054	24	9	11	
FAM179A	LOC165186	2p23.2a	0.0054	28	5	11	family with sequence similarity 179, member A
C2orf71	FLJ34931	2p23.2a	0.0054	28	5	11	chromosome 2 open reading frame 71
TMEM92	TMEM92	17q21.33a	0.0055	101	5	27	transmembrane protein 92
PRKCA	PRKCA	17q24.2a	0.0056	105	6	28	protein kinase C, alpha
NUP155	NUP155	5p13.2b	0.0057	41	9	15	nucleoporin 155kDa
WDR70	GUSBL2	6p11.2a	0.0057	41	9	15	WD repeat domain 70
ZBTB8OS	ZBTB8OS	1p35.1b	0.0057	18	32	15	zinc finger and BTB domain containing 8 opposite strand
ZBTB8A	ZBTB8	1p35.1b	0.0057	18	32	15	zinc finger and BTB domain containing 8A
ZFP42	ZFP42	4q35.2b	0.0057	4	46	15	zinc finger protein 42 homolog (mouse)
SCP2	SCP2	1p32.3d-p32	0.0057	28	22	15	sterol carrier protein 2
KTI12	KTI12	1p32.3e	0.0057	24	26	15	KTI12 homolog, chromatin associated (S. cerevisiae)
PDE4B	PDE4B	1p31.3b	0.0059	36	23	17	phosphodiesterase 4B, cAMP-specific
ESSPL	ESSPL	4q31.3b	0.0059	4	14	7	
AK130848	LOC647606		0.0059	12	6	7	
ARHGAP15	ARHGAP15	2q22.2a	0.0059	9	9	7	Rho GTPase activating protein 15
PDCL3	PDCL3	2q11.2d	0.0059	10	8	7	phosducin-like 3
GCG	GCG	2q24.2d	0.0059	10	8	7	glucagon
DPP4	DPP4	2q24.2d	0.0059	10	8	7	dipeptidyl-peptidase 4
ITGB6	ITGB6	2q24.2b	0.0059	13	5	7	integrin, beta 6
TTC21B	TTC21B	2q24.3c-q24.	0.0059	11	7	7	tetratricopeptide repeat domain 21B
HOXD1	HOXD1	2q31.1h	0.0059	13	5	7	homeobox D1
SLC38A11	SLC38A11	2q24.3b-q24	0.0059	10	8	7	solute carrier family 38, member 11
CSRNP3	FAM130A2	2q24.3c	0.0059	11	7	7	cysteine-serine-rich nuclear protein 3
TOM1L1	TOM1L1	17q22b	0.006	80	7	23	target of myb1 (chicken)-like 1
COX11	COX11	17q22b	0.006	80	7	23	COX11 cytochrome c oxidase assembly homolog (yeast)
CREM	CREM	10p11.21c	0.0062	37	9	14	cAMP responsive element modulator
HEATR6	HEATR6	17q23.1a	0.0063	119	8	31	HEAT repeat containing 6
DHX40P	TBC1D3B	17q12b	0.0063	119	8	31	TBC1 domain family, member 3B
USP32	LOC650546		0.0064	114	8	30	ubiquitin specific peptidase 32
ABI3	ABI3	17q21.32c	0.0064	89	13	26	ABI family, member 3
PHOSPHO1	PHOSPHO1	17q21.32c	0.0064	89	13	26	phosphatase, orphan 1
TLK2	LOC641806		0.0064	109	8	29	tousled-like kinase 2
SPATA20	SPATA20	17q21.33b	0.0065	101	6	27	spermatogenesis associated 20
POFUT2	POFUT2	21q22.3e	0.0065	59	5	18	protein O-fucosyltransferase 2
C17orf57	C17orf57	17q21.32a-q	0.0065	31	24	16	chromosome 17 open reading frame 57
UNQ2783	MGC52498	1p32.3d	0.0065	30	25	16	
C1orf163	C1orf163	1p32.3d	0.0065	30	25	16	
ZYG11B	ZYG11B	1p32.3d	0.0065	28	27	16	zyg-11 homolog B (C. elegans)
FAM159A	FAM159A	1p32.3d	0.0065	30	25	16	family with sequence similarity 159, member A
GPX7	GPX7	1p32.3d	0.0065	30	25	16	glutathione peroxidase 7
ATG4C	ATG4C	1p31.3c	0.0065	30	25	16	ATG4 autophagy related 4 homolog C (S. cerevisiae)
SLC44A5	SLC44A5	1p31.1g	0.0066	15	27	13	solute carrier family 44, member 5
LHX8	LHX8	1p31.1g	0.0066	15	27	13	LIM homeobox 8
BEND5	C1orf165	1p33b	0.0066	25	17	13	BEN domain containing 5
HMGN1	HMGN1	21q22.2b	0.0066	31	11	13	high mobility group nucleosome binding domain 1
RPS7P4	LOC149224	1p31.3a	0.0066	22	20	13	ribosomal protein S7 pseudogene 4
LOC645291	LOC645291	1p31.3a	0.0066	24	18	13	
MAB21L2	MAB21L2	4q31.3a	0.0068	4	18	8	mab-21-like 2 (C. elegans)
BEND4	CCDC4	4p13d	0.0068	1	21	8	BEN domain containing 4

CYP26C1	CYP26C1	10q23.33a	0.0068	4	18	8	cytochrome P450, family 26, subfamily C, polypeptide 1
CYP26A1	CYP26A1	10q23.33a	0.0068	4	18	8	cytochrome P450, family 26, subfamily A, polypeptide 1
BC157063	CYP26A1	10q23.33a	0.0068	4	18	8	
THADA	THADA	2p21e-p21d	0.0068	17	5	8	thyroid adenoma associated
SLC30A9	SLC30A9	4p13d	0.0068	1	21	8	solute carrier family 30 (zinc transporter), member 9
SMPD4	FLJ41352	2q21.1d	0.0068	16	6	8	sphingomyelin phosphodiesterase 4, neutral membrane (neutral sphingomyelinase-3)
TUBA3D	TUBA3D	2q21.1d	0.0068	16	6	8	tubulin, alpha 3d
FLJ14346	LOC653904		0.0068	16	6	8	
FAM128A	FAM128A	2q21.1d	0.0068	16	6	8	
ACVR2A	ACVR2A	2q22.3c-q23.	0.0068	10	12	8	activin A receptor, type IIA
ORC4L	ORC4L	2q23.1a	0.0068	10	12	8	
AGBL4	AGBL4	1p33c-p33b	0.0069	22	16	12	ATP/GTP binding protein-like 4
KCTD8	KCTD8	4p13b	0.0069	6	32	12	potassium channel tetramerisation domain containing 8
C2orf18	C2orf18	2p23.3a	0.0069	30	8	12	chromosome 2 open reading frame 18
CENPA	CENPA	2p23.3a	0.0069	30	8	12	centromere protein A
ANGPTL3	ANGPTL3	1p31.3d	0.007	37	32	19	angiopoietin-like 3
DQ896418	COX11P	6p22.1b	0.0071	81	7	23	
ZYG11A	ZYG11A	1p32.3d	0.0071	29	22	15	zyg-11 homolog A (C. elegans)
ECHDC2	ECHDC2	1p32.3d	0.0071	29	22	15	enoyl CoA hydratase domain containing 2
RAB3B	RAB3B	1p32.3e	0.0071	23	28	15	RAB3B, member RAS oncogene family
NRD1	NRD1	1p32.3e	0.0071	24	27	15	nardilysin (N-arginine dibasic convertase)
SLC35D1	SLC35D1	1p31.3a	0.0071	27	24	15	solute carrier family 35 (UDP-glucuronic acid/UDP-N-acetylgalactosamine dual transporter), member D1
SELI	SELI	2p23.3b	0.0072	18	8	9	
N4BP2	N4BP2	4p14b	0.0072	1	25	9	NEDD4 binding protein 2
KLHL5	KLHL5	4p14c	0.0072	2	24	9	kelch-like 5 (Drosophila)
WDR36	WDR36	5q22.1a	0.0072	12	22	11	WD repeat domain 36
TIAL1	TIAL1	10q26.11d	0.0072	17	17	11	TIA1 cytotoxic granule-associated RNA binding protein-like 1
TUBB4Q	LOC649679		0.0072	6	28	11	
AX747733	LOC643396		0.0072	16	18	11	
DN994130	LOC391037	1p33c	0.0072	19	15	11	
SLC5A9	SLC5A9	1p33c	0.0072	19	15	11	solute carrier family 5 (sodium/glucose cotransporter), member 9
CYP4Z2P	LOC652843		0.0072	21	13	11	cytochrome P450, family 4, subfamily Z, polypeptide 2 pseudogene
CYP4A11	CYP4A11	1p33d	0.0072	21	13	11	cytochrome P450, family 4, subfamily A, polypeptide 11
KIDINS220	KIDINS220	2p25.1e	0.0072	29	5	11	kinase D-interacting substrate, 220kDa
GABRA4	GABRA4	4p12b	0.0072	12	22	11	gamma-aminobutyric acid (GABA) A receptor, alpha 4
UNQ6975	UNQ6975		0.0072	30	4	11	putative uncharacterized protein UNQ6975/PRO21958
CHRNA9	CHRNA9	4p14a	0.0073	5	25	10	cholinergic receptor, nicotinic, alpha 9 (neuronal)
Mar-05	Mar-05	10q23.32b	0.0073	4	26	10	membrane-associated ring finger (C3HC4) 5
SAP130	SAP130	2q14.3e	0.0074	8	7	6	Sin3A-associated protein, 130kDa
POLR2D	POLR2D	2q14.3e	0.0074	8	7	6	polymerase (RNA) II (DNA directed) polypeptide D
AMMECR1L	AMMECR1L	2q14.3e	0.0074	8	7	6	AMME chromosomal region gene 1-like
C2orf27B	MGC50273	2q21.2a	0.0074	11	4	6	chromosome 2 open reading frame 27B
C2orf27A	LOC642669	14q11.1d	0.0074	11	4	6	chromosome 2 open reading frame 27A
NBEA	LOC647974		0.0074	11	4	6	neurobeachin
MKI67IP	MKI67IP	2q14.3a	0.0074	9	6	6	MKI67 (FHA domain) interacting nucleolar phosphoprotein
TSN	TSN	2q14.3a	0.0074	9	6	6	translin
DQ895626	C2orf40	2q12.2b	0.0074	9	6	6	chromosome 2 open reading frame 40
NAP5	NAP5	2q21.2b-q21	0.0074	9	6	6	
GPR39	GPR39	2q21.2b	0.0074	10	5	6	G protein-coupled receptor 39
MGAT4A	MGAT4A	2q11.2c	0.0074	11	4	6	mannosyl (alpha-1,3-)-glycoprotein beta-1,4-N-acetylglucosaminyltransferase, isozyme A
CNGA3	CNGA3	2q11.2c	0.0074	11	4	6	cyclic nucleotide gated channel alpha 3
C2orf55	MGC42367	2q11.2c	0.0074	11	4	6	chromosome 2 open reading frame 55
VWA3B	MGC26733	2q11.2b-q11	0.0074	11	4	6	von Willebrand factor A domain containing 3B
CDCA7	CDCA7	2q31.1e	0.0074	11	4	6	cell division cycle associated 7
ITGA6	ITGA6	2q31.1d	0.0074	10	5	6	integrin, alpha 6
LRP2	LRP2	2q31.1a	0.0074	9	6	6	low density lipoprotein receptor-related protein 2
PLA2R1	PLA2R1	2q24.2a-q24	0.0074	9	6	6	phospholipase A2 receptor 1, 180kDa
DYNLL2	DYNLL2	17q22d	0.0075	99	4	26	dynein, light chain, LC8-type 2
AK095981	LOC648302		0.0075	35	78	28	
MRPS23	MRPS23	17q22d	0.0075	104	4	27	mitochondrial ribosomal protein S23
CUEDC1	CUEDC1	17q22d	0.0075	104	4	27	CUE domain containing 1
MYST2	MYST2	17q21.33a	0.0075	100	8	27	
SRP14	LOC644863		0.0075	100	8	27	signal recognition particle 14kDa (homologous Alu RNA binding protein)
CACNA1G	CACNA1G	17q21.33b	0.0075	102	6	27	calcium channel, voltage-dependent, T type, alpha 1G subunit
EPN3	EPN3	17q21.33b	0.0075	102	6	27	epsin 3
SNORD21	SNORD21	1p22.1c	0.0078	12	35	14	small nucleolar RNA, C/D box 21
LOC642852	LOC642852	21q22.3e	0.0078	60	5	18	uncharacterized LOC642852
DOCK7	DOCK7	1p31.3d-p31	0.0078	34	31	18	dedicator of cytokinesis 7
SMN2	SMN2	5q13.2a	0.0084	5	38	13	survival of motor neuron 2, centromeric
NAIP	LOC651112		0.0084	5	38	13	NLR family, apoptosis inhibitory protein
GUSBP1	SMA4	5q13.2a	0.0084	5	38	13	glucuronidase, beta pseudogene 1
SERF1B	SERF1B	5q13.2a	0.0084	5	38	13	small EDRK-rich factor 1B (centromeric)
SMN1	SMN1	5q13.2b	0.0084	5	38	13	survival of motor neuron 1, telomeric
ST6GALNAC3	ST6GALNAC3	1p31.1g-p31.	0.0084	18	25	13	ST6 (alpha-N-acetyl-neuraminyl-2,3-beta-galactosyl-1,3)-N-acetylgalactosaminide alpha-2,6-sialyltransferase 3
SPATA6	SPATA6	1p33c	0.0084	24	19	13	spermatogenesis associated 6
GPR177	GPR177	1p31.3a	0.0084	24	19	13	
DIRAS3	DIRAS3	1p31.3a	0.0084	23	20	13	DIRAS family, GTP-binding RAS-like 3
C2orf79	C2orf79	2p23.3c	0.0084	31	12	13	
NRP1	NRP1	10p11.22a	0.0084	38	5	13	neuropilin 1
C17orf73	C17orf73	17q21.33b	0.0087	98	6	26	
MGAT5	MGAT5	2q21.2d-q21	0.0088	13	6	7	mannosyl (alpha-1,6-)-glycoprotein beta-1,6-N-acetyl-glucosaminyltransferase
LOC1001307	LOC648682		0.0088	10	9	7	
GTDC1	GTDC1	2q22.2a-q22	0.0088	10	9	7	glycosyltransferase-like domain containing 1
PAIP2B	PAIP2B	2p13.3b	0.0088	17	2	7	poly(A) binding protein interacting protein 2B
TANK	TANK	2q24.2c	0.0088	11	8	7	TRAF family member-associated NFKB activator
FAP	FAP	2q24.2d	0.0088	11	8	7	fibroblast activation protein, alpha
TBR1	TBR1	2q24.2c	0.0088	10	9	7	T-box, brain, 1
SCN3A	SCN3A	2q24.3c	0.0088	11	8	7	sodium channel, voltage-gated, type III, alpha subunit
ZNF2	ZNF2	2q11.1c	0.0088	14	5	7	zinc finger protein 2
ZNF514	ZNF514	2q11.1c	0.0088	14	5	7	zinc finger protein 514
TTC30B	TTC30B	2q31.2a	0.0088	14	5	7	tetratricopeptide repeat domain 30B
SCN2A	SCN2A	2q24.3c	0.0088	11	8	7	sodium channel, voltage-gated, type II, alpha subunit
HLA-A	HLA-A	6p21.33b	0.0088	43	9	15	major histocompatibility complex, class I, A
BTF3L4	BTF3L4	1p32.3d	0.0088	26	26	15	basic transcription factor 3-like 4
ZFYVE9	ZFYVE9	1p32.3d	0.0088	26	26	15	zinc finger, FYVE domain containing 9
OSBPL9	OSBPL9	1p32.3e	0.0088	25	27	15	oxysterol binding protein-like 9
KRTAP10-12	KRTAP10-12	21q22.3d	0.0088	45	7	15	keratin associated protein 10-12
HS1BP3	HS1BP3	2p24.1c	0.0088	42	10	15	HCLS1 binding protein 3
ANKRD20A1	LOC730920		0.0089	10	2	5	ankyrin repeat domain 20 family, member A1
C2orf15	C2orf15	2q11.2c	0.0089	7	5	5	chromosome 2 open reading frame 15

SULT1C3	SULT1C3	2q12.3c	0.0089	5	7	5	sulfotransferase family, cytosolic, 1C, member 3
SULT1C4	SULT1C4	2q12.3c	0.0089	5	7	5	sulfotransferase family, cytosolic, 1C, member 4
LIMS3	LOC729260	2q13b	0.0089	7	5	5	LIM and senescent cell antigen-like domains 3
SULT1C2	SULT1C2	2q12.3c	0.0089	5	7	5	sulfotransferase family, cytosolic, 1C, member 2
BM720600	LOC653596		0.0089	7	5	5	
AK096170	LOC440895	2q13b	0.0089	7	5	5	two pore channel 3 pseudogene
MRPS9	MRPS9	2q12.1d	0.0089	6	6	5	mitochondrial ribosomal protein S9
AK125994	FLJ44006	2q13c	0.0089	7	5	5	uncharacterized FLJ44006
ACOXL	ACOXL	2q13c	0.0089	7	5	5	acyl-CoA oxidase-like
NEB	LOC649199		0.0089	5	7	5	nebulin
AX748125	FLJ36116	1p13.2c	0.0089	16	23	12	uncharacterized LOC388666
DSCAM	DSCAM	21q22.2b-q2	0.0089	29	10	12	Down syndrome cell adhesion molecule
CASP3	CASP3	4q35.1e	0.0089	7	32	12	caspase 3, apoptosis-related cysteine peptidase
OPN4	OPN4	10q23.2a	0.0089	22	17	12	opsin 4
LBP-32	GRHL1	2p25.1d	0.0089	34	5	12	grainyhead-like 1 (Drosophila)
POMC	POMC	2p23.3c	0.0089	29	10	12	proopiomelanocortin
DNMT3A	DNMT3A	2p23.3c	0.0089	30	9	12	DNA (cytosine-5-)-methyltransferase 3 alpha
EFR3B	KIAA0953	2p23.3c	0.0089	29	10	12	EFR3 homolog B (S. cerevisiae)
ARAP2	CENTD1	4p14f	0.0089	6	33	12	ArfGAP with RhoGAP domain, ankyrin repeat and PH domain 2
WNT2B	WNT2B	1p13.2c	0.0094	15	20	11	wingless-type MMTV integration site family, member 2B
DENND2D	DENND2D	1p13.3a	0.0094	20	15	11	DENN/MADD domain containing 2D
CTTNBP2NL	CTTNBP2NL	1p13.2c	0.0094	15	20	11	CTTNBP2 N-terminal like
FRG1	FRG1	4q35.2d	0.0094	7	28	11	FSHD region gene 1
LOC283788	LOC283788		0.0094	7	28	11	FSHD region gene 1 pseudogene
CYP4Z1	CYP4Z2P	1p33d	0.0094	22	13	11	cytochrome P450, family 4, subfamily Z, polypeptide 1
CYP4X1	CYP4X1	1p33d	0.0094	22	13	11	cytochrome P450, family 4, subfamily X, polypeptide 1
AK124409	FLJ42418	2p25.2a	0.0094	29	6	11	
ATP6V1E2	ATP6V1E2	2p21b-p21a	0.0094	32	3	11	ATPase, H+ transporting, lysosomal 31kDa, V1 subunit E2
RHOQ	RHOQ	2p21a	0.0094	32	3	11	ras homolog family member Q
GABRG1	GABRG1	4p12b	0.0094	11	24	11	gamma-aminobutyric acid (GABA) A receptor, gamma 1
C21orf93	C21orf93	21q22.3e	0.0094	61	5	18	
TBX4	TBX4	17q23.2b	0.0095	125	5	31	T-box 4
DKFZp761O0	CLIP4	2p23.2a	0.0095	20	3	8	CAP-GLY domain containing linker protein family, member 4
ALK	ALK	2p23.2a-p23	0.0095	20	3	8	anaplastic lymphoma receptor tyrosine kinase
KLF7	KLF7	2q33.3c	0.0095	17	6	8	Kruppel-like factor 7 (ubiquitous)
APC	APC	5q22.2a	0.0097	12	36	14	adenomatous polyposis coli
RPL5	FAM69A	1p22.1c	0.0097	13	35	14	ribosomal protein L5
AK091004	PPP2R2B	5q32d-q32e	0.0097	23	25	14	protein phosphatase 2, regulatory subunit B, beta
CASC4	CASC4	15q15.3c	0.0097	3	45	14	cancer susceptibility candidate 4
CPT2	CPT2	1p32.3c	0.0097	32	16	14	carnitine palmitoyltransferase 2
C1orf123	C1orf123	1p32.3c	0.0097	32	16	14	chromosome 1 open reading frame 123
USP24	USP24	1p32.3a	0.0097	35	13	14	ubiquitin specific peptidase 24
PCSK9	PCSK9	1p32.3a	0.0097	35	13	14	proprotein convertase subtilisin/kexin type 9
CUL2	CUL2	10p11.21c	0.0097	38	10	14	cullin 2
H2AFY	H2AFY	5q31.1f	0.0097	20	11	10	H2A histone family, member Y
FBXO22	FBXO22	15q24.2a	0.0097	12	19	10	F-box protein 22
NRG4	NRG4	15q24.2a	0.0097	12	19	10	neuregulin 4
DQ894431	GTF2A2	15q22.2a	0.0097	7	24	10	general transcription factor IIA, 2, 12kDa
HS3ST1	HS3ST1	4p15.33e	0.0097	5	26	10	heparan sulfate (glucosamine) 3-O-sulfotransferase 1
CEACAM20	CEACAM20	19q13.31b	0.0097	19	12	10	carcinoembryonic antigen-related cell adhesion molecule 20
CB990789	LOC649270		0.0098	37	20	16	
tcag7.935	LOC389457		0.0098	48	9	16	
SLC6A17	SLC6A17	1p13.3a	0.0098	39	18	16	solute carrier family 6, member 17
TIFAB	TIFAB	5q31.1f	0.0098	16	11	9	TRAF-interacting protein with forkhead-associated domain, family member B
SLC25A26	SLC25A26	3p14.1c	0.0098	7	20	9	solute carrier family 25, member 26
LRIG1	LRIG1	3p14.1c	0.0098	7	20	9	leucine-rich repeats and immunoglobulin-like domains 1
HADHB	HADHB	2p23.3b	0.0098	19	8	9	hydroxyacyl-CoA dehydrogenase/3-ketoacyl-CoA thiolase/enoyl-CoA hydratase (trifunctional protein), beta subunit
GPR113	GPR113	2p23.3b	0.0098	19	8	9	G protein-coupled receptor 113
PGM2	PGM2	4p14d	0.0098	2	25	9	phosphoglucomutase 2
GRXCR1	GRXCR1	4p13c	0.0098	4	23	9	glutaredoxin, cysteine rich 1
SH3RF3	SH3MD4	2q13a	0.0098	21	6	9	SH3 domain containing ring finger 3
SDHALP1	SDHALP1	3q29f	0.0099	88	7	24	
METRNL	METRNL	17q25.3h	0.0099	104	11	28	meteorin, glial cell differentiation regulator-like
ZC3H8	ZC3H8	2q13d	0.01	4	5	4	zinc finger CCCH-type containing 8
MERTK	MERTK	2q13c-q13d	0.01	4	5	4	c-mer proto-oncogene tyrosine kinase
