# Supplementary material for: Microenvironmental Heterogeneity Parallels Breast Cancer Progression: A Histology–Genomic Integration Analysis
Source: PLoS Med. 2016 Feb 16;13(2):e1001961. doi: 10.1371/journal.pmed.1001961 (PMC4755617; doi:10.1371/journal.pmed.1001961)
Supplement: S3 Table — (DOCX) [file pmed.1001961.s015.docx]

**S3 Table.** **Prognostic significance of the 4p14 and 5q13 loss and EDI groups in independent subsets of grade 3 breast tumors in univariate and multivariate Cox regression with Node and Size**

|  | **Cohort 1 (n=251)** | | | | **Cohort 2 (n=256)** | | | |
| --- | --- | --- | --- | --- | --- | --- | --- | --- |
| **Variable** | **Univariate hazard ratio (95% CI)** | **p** | **Multiivariate hazard ratio (95% CI)** | **p** | **Univariate hazard ratio (95% CI)** | **p** | **Multiivariate hazard ratio (95% CI)** | **p** |
| **4p14&EDI** | 4.89(2.42-9.86) | 9.5x10^-7^** | 4.4(2.17-8.93) | 4x10^-5^** | 8.13(1.1-60.16) | 0.014* | 17.52(2.11-145.74) | 0.0081** |
| **5q13&EDI** | 0.58 (0.37 - 0.89) | 3.02(1.39-6.58) | 2.82(1.3-6.14) | 0.0089** | 8.56(2.02-36.36) | 0.00046** | 12.18(2.78-53.26) | 9 x10^-4^** |
| **Node** | 2.01 (1.25 - 3.24) | 0.0034** | 1.83(1.13-2.97) | 0.014* | 2.61 (1.29 - 5.26) | 0.0054** | 1.92(0.92-3.98) | 0.081 |
| **Size** | 2.01 (1.33 - 3.04) | 0.00090** | 1.76(1.14-2.71) | 0.011* | 3.03 (1.73 - 5.30) | 0.0001** | 2.7(1.49-4.89) | 0.001** |
