# Supplementary material for: Microenvironmental Heterogeneity Parallels Breast Cancer Progression: A Histology–Genomic Integration Analysis
Source: PLoS Med. 2016 Feb 16;13(2):e1001961. doi: 10.1371/journal.pmed.1001961 (PMC4755617; doi:10.1371/journal.pmed.1001961)
Supplement: S1 Text — (DOCX) [file pmed.1001961.s016.docx]

Supporting Information: S1 Text

**Supplementary Methods**

**Choice of region size and stability of clusters**

To identify regional clusters within a tumor, tumor regions need to be small enough so that there are sufficient numbers of regions, yet sufficiently large to account for local characteristics of the cancer ecosystem. We based our criteria for region size selection on several factors, including 1) the effect of region size on the number of regions per tumor, 2) number of cells per region, and 3) stability of clustering. First, based on the region size *r* as a *r*-by-*r* square, we plotted the average number of regions in all tumors and average number of cells per region (S1 Fig A). Based on this, regions of 200um capture a reasonable amount of cells whilst allowing sufficient number of regions for clustering.

Another criterion to evaluate sufficient number of regions for clustering is the clustering stability. This is based on the notion that a stable clustering can be reproduced even with less data points, here we used 90% of the data. By sampling 90% regions 10 times in 20 randomly selected tumors, we can measure the instability of clustering as

$s=\frac{\sum_{i=1}^{20} sd\left( k_{i}/w \right)}{20},$Eq.3

where $k_{i}=k_{1},k_{2},\ldots,k_{10}$for a tumor *i* is a vector of the number of clusters produced using the same clustering method Eq.1 but based on random sampling, *w* is a normalizing factor of maximum amount of variability in the clustering, defined as *sd(1, 1, …, m, m)* and *m* being the maximum number of the clusters. Thus *s* has a range of [0,1], and the higher the *s,* the more unstable the clustering. According to this criterion, the most stable clustering occurs at region sizes of 200 and 166μm (S1 Fig B). In addition, we examined the diversity scores as a function of region size. The smaller the region is, the more likely that the region captures single cell types, the extreme case being one cell per region. Indeed, average diversity decreases with decreasing region size using 20 randomly sampled tumors (S1 Fig C). Therefore, we chose a region size of 200μm by 200μm, that is, 400 pixels by 400 pixels square regions in H&E images of 20x magnification. Cells in the same region are within the effective cell communication range via a cyto/chemokine, ≈250μm ^45^.

On average, a region of this size contains 64$\pm$24 cells, and a tumor contains 1,185$\pm$808 regions. Due to possible empty space in the tissue section image, regions with less than 25% of the average number of cells (16 cells) were removed prior to clustering. Good correlation in the Q-Q plots of all tumors suggests that our clustering method provides a good fit of the data (S1 Fig D-E). We also evaluated the differences in EDI computed using different spatial configurations defined by region size r=166, 200, 250, 333, 500μm. The correlation of EDI indices ranges from 0.19 to 0.53 (S2 Fig). This is not unexpected since *r* directly determines the number of cells in each region and the total number of regions.

**Robustness of the EDI subtype to sampling variability**

To test the robustness of EDI subtype against sampling issues, we performed resampling experiments to evaluate its performance against a decreasing amount of tumor regions. Each time 200 grade 3 tumors were randomly chosen. For each tumor 99%, 98%, …, 80% of tumor regions were randomly sampled and EDI scores computed based on sampled regions. These scores were then compared to the EDI scores computed using 100% regions. Agreement between the two scoring was computed as accuracy (the proportion of true positive or true negative samples). The analysis upon 200 repeated sampling showed that with only 80% of data, the agreement with result from 100% data remains at 90%. More importantly, the ability of EDI to stratify these 200 patients is stable with fewer data (S3 Fig.). 90% of the times during resampling, the log-rank *p*-values remain significant.

**Morphology-molecular association**

We used LIMMA [1] to test if there are significant correlations between EDI group and gene expression data. For test of pair-wise association between EDI group and copy number status which are dichotomized data, we used Fisher’s exact test with permutation resampling to avoid the problem of multiple testing and to calculate exact permutation probability [2]. Fisher’s exact test aims to statistically determine whether there is an association between two groups of observations. For each gene/locus *i*, we used the EDI group as one group of observation and the copy number aberrations in this gene – either gains or losses – as the other group. The probability of association is thus:

$p_{i}=\frac{\left( \begin{matrix} q \\ a_{i} \end{matrix} \right)\left( \begin{matrix} m-q \\ b_{i} \end{matrix} \right)}{\left( \begin{matrix} m \\ a_{i}+b_{i} \end{matrix} \right)}$, Eq.4

where *m* is the total number of samples/patients, *q* is the number of EDI-high samples, $a_{i}$ is the number of samples with copy number aberrations in gene *i* within the EDI-high group, $b_{i}$ is the number of samples with copy number aberrations in gene *i* but not in the EDI-high group.

For testing the explanatory effect of genome-wide copy number for the EDI subtypes, we used logistic *lasso* regression [3]. Previously we have successfully applied this method to identifying explanatory copy number variables for expression traits [4]. Here EDI is the trait or response variable *y*, and copy number data as the explanatory variables *x_i,_ i=1, 2, …, N, N* being the number of copy number loci:

$y=\sum_{i} \beta_{i}x_{i}+\varepsilon^{2}$, Eq.5

where a non-zero $\beta_{i}$ indicates that the variable $x_{i}$ contributes towards explaining the variation in *y*. Only the top ranking copy number loci determined by the pair-wise test described above were used. In our tests, *lasso* regression failed to converge with the top 50, 100, 200, 800 variables, indicating that there is no sufficient power to explain *y*/EDI with copy number variables.

**References**

1. Smyth GK. Limma: linear models for microarray data. Bioinformatics and Computational Biology Solutions using R and Bioconductor. Springer, New York; 2005. p. 397–420.

2. Brown CC, Fears TR. Exact Significance Levels for Multiple Binomial Testing with Application to Carcinogenicity Screens. Biometrics. 1981;37(4):763–74.

3. Tibshirani R. Regression shrinkage and selection via the lasso. J R Stat Soc. 1994;58(1):267–88.

4. Yuan Y, Curtis C, Caldas C, Markowetz F. A sparse regulatory network of copy-number driven gene expression reveals putative breast cancer oncogenes. IEEE/ACM Trans Comput Biol Bioinform. 2011/07/27 ed. 2012;9(4):947–54.
