# Supplementary material for: Microenvironmental Heterogeneity Parallels Breast Cancer Progression: A Histology–Genomic Integration Analysis
Source: PLoS Med. 2016 Feb 16;13(2):e1001961. doi: 10.1371/journal.pmed.1001961 (PMC4755617; doi:10.1371/journal.pmed.1001961)
Supplement: S2 Text — (PDF) [file pmed.1001961.s017.pdf]

Supplementary materials, R code and data for Microenvironmental Heterogeneity Parallels Breast Cancer Progression: A Histology-Genomic Integration Analysis

Contents

1 Load data and functions **3**

2 Distribution of EDI in breast cancer **4**
2.1 Distribution of EDI in all tumors and Grade 3 tumors 4
2.2 Correlation between EDI and clinicopathological parameters 5
2.3 Correlation between EDI and tumor composition 6

3 Association between EDI and breast cancer prognosis **7**
3.1 Survival data in METABRIC cohorts 7
3.2 Univariate analysis of prognostic significance of EDI in high- and low-grade tumors . . 8
3.3 Univariate analysis of prognostic significance of existing clinicopathological parameters and tumor composition in high-grade tumors 14
3.3.1 Clinicopathological parameters including node, size and genomic subtypes . . . 14
3.3.2 ER and HER2 status measured by IHC and microarrays 16
3.3.3 Cell proportions determined by image analysis and pathological scores 20
3.4 Multivariate analysis of EDI5 and survival-associated clinicopathological parameters in high-grade tumors 24
3.5 Further stratification for Grade 3 tumors 26
3.5.1 Further stratification for node, size and ER status 26
3.5.2 Additional stratification combining EDI5, node and size 30
3.6 EDI and the Genomic Grade Index 32
3.7 Compare EDI and Shannon entropy of the whole tumor 33

4 Generating EDI scores **36**

5 Stability of EDI **37**
5.1 Resampling tumor regions 37
5.2 Correlation with input data 42
5.3 Determining parameters 45

6 Association of EDI with genomics **46**
6.1 Synergistic association between EDI5 and 4p14, 5q13 47
6.2 Cox regression analysis for EDI5 and 4p14, 5q13 50

7 Comparison with cancer hallmarks **53**
7.1 Prognostic value of tumor heterogeneity measures 53
7.2 Correlation with grade node and size 54

7.3 Correlation among EDI5 and cancer hallmarks 55
7.4 Multivariate analysis of cancer hallmark measures 57
7.5 Association with TP53 mutation 60

8 Robustness of the Cox model **68**

9 Session Info **70**

1 Load data and functions

This document presents R codes and data for reproducing our analysis result as described in the paper. To start, first load the clinical data of METABRIC samples and R functions for subsequent analysis and plotting. The data file is available as part of the paper supplement and online ("http://yuanlab.org/software/EDI/download").

```
load("./data/trait.rdata")
source("EDIfunctions.R")
head(trait)

##          file grade node size       GI Pam50Subtype IntClustMemb S_10year
## MB-0000 1645     3    1    2 0.008151       Normal            4   99.97+
## MB-0002 1729     3    0    1 0.048145         LumA            4   49.47+
## MB-0005 1646     2    1    1 0.308214         LumB            3  101.77+
## MB-0006 1647     2    1    2 0.097780         LumB            9   57.37+
## MB-0008 1648     3    1    2 0.165905         LumB            9   41.37
## MB-0010 1649     3    0    2 0.139772         LumB            7    7.80
##          Site APOBEC3B  er her2 ER.Expr Her2.SNP6 Her2.Expr cancer stromal
## MB-0000    2  -0.9906 pos null       +         0         - 0.7121 0.13328
## MB-0002    2  -0.7038 pos null       +         0         - 0.7633 0.02527
## MB-0005    2  -0.3099 pos null       +         0         - 0.7752 0.03581
## MB-0006    2  -0.3863 pos    0       +         0         - 0.7045 0.04043
## MB-0008    2   1.0764 pos    0       +         0         - 0.7204 0.06209
## MB-0010    2   1.2308 pos null       +         0         - 0.7282 0.04939
##             lym TP53 Coding.description Codon.change Protein.change
## MB-0000 0.1546    0
## MB-0002 0.2114    1           c.533A>C      CAC>CCC        p.H178P
## MB-0005 0.1889    1           c.542G>A      CGC>CAC        p.R181H
## MB-0006 0.2550    0
## MB-0008 0.2175    1           c.722C>T      TCC>TTC        p.S241F
## MB-0010 0.2224    1          c.200del1       CCA>NA            p.?
##          Lymphocyte.infiltration      GGI    ct    rt   ht
## MB-0000                     mild -1.78398 FALSE  TRUE TRUE
## MB-0002                     mild -0.35481 FALSE  TRUE TRUE
## MB-0005                     mild  0.31357  TRUE FALSE TRUE
## MB-0006                     mild -0.09698  TRUE  TRUE TRUE
## MB-0008                     mild  0.95107  TRUE  TRUE TRUE
## MB-0010                     mild  0.72861 FALSE  TRUE TRUE
```

Each row is a sample/patient from the METABRIC database. Columns indicate: clinical grade (grade), lymph node status (node), tumor size (size), genomic instability (GI), Pam50 intrinsic subtype classification (Pam50Subtype), IntClust subtype classification (IntClustMemb), 10-year disease-free survival (S_10year), sample subsets (Site), APOBEC3B expression from microarray (APOBEC3B), ER status by IHC (er), ER status by gene expression microarray (ER.Expr), Her2 status by pathology (her2), Her2 status measured by gene expression microarray (Her2.Expr), Her2 status by SNP6 microarray (Her2.SNP6), proportion of cancer, stromal cells and lymphocytes (cancer, stromal, lym), TP53 mutation status (TP53) and description of these mutations (Coding.description, Codon.change, Protein.change), pathological scores of lymphocytic infiltration (Lymphocyte.infiltration), Genomic grade index (GGI).

Color codes for Pam50 clusters and IntClust as used in the paper are defined here.

```
pam50colors <- c("#E41A1C", "#FB9A99", "#1F78B4", "#A6CEE3", "#66A61E")
names(pam50colors) <- c("Basal", "Her2", "LumA", "LumB", "Normal")
```

```
col <- c("#FF5500", "#00EE76", "#CD3278", "#00C5CD", "#8B0000", "#FFFF40", "#0000CD",
    "#FFAA00", "#EE82EE", "#7D26CD")
```

2 Distribution of EDI in breast cancer

2.1 Distribution of EDI in all tumors and Grade 3 tumors

We load the EDI scores for 1,026 breast tumors. Details on how we computed these scores will be given in the next section. We used EDI5 to denote the EDI-high group.

```
load("./data/EDI.rdata")
par(mfrow = c(1, 2))
set2 <- grepl(3, trait$grade)
EDI5 <- EDI == 5
hist(EDI, main = "Distribution of EDI in 1026 breast cancer")
hist(EDI[set2], main = "Distribution of EDI  in high-grade breast cancer")
```

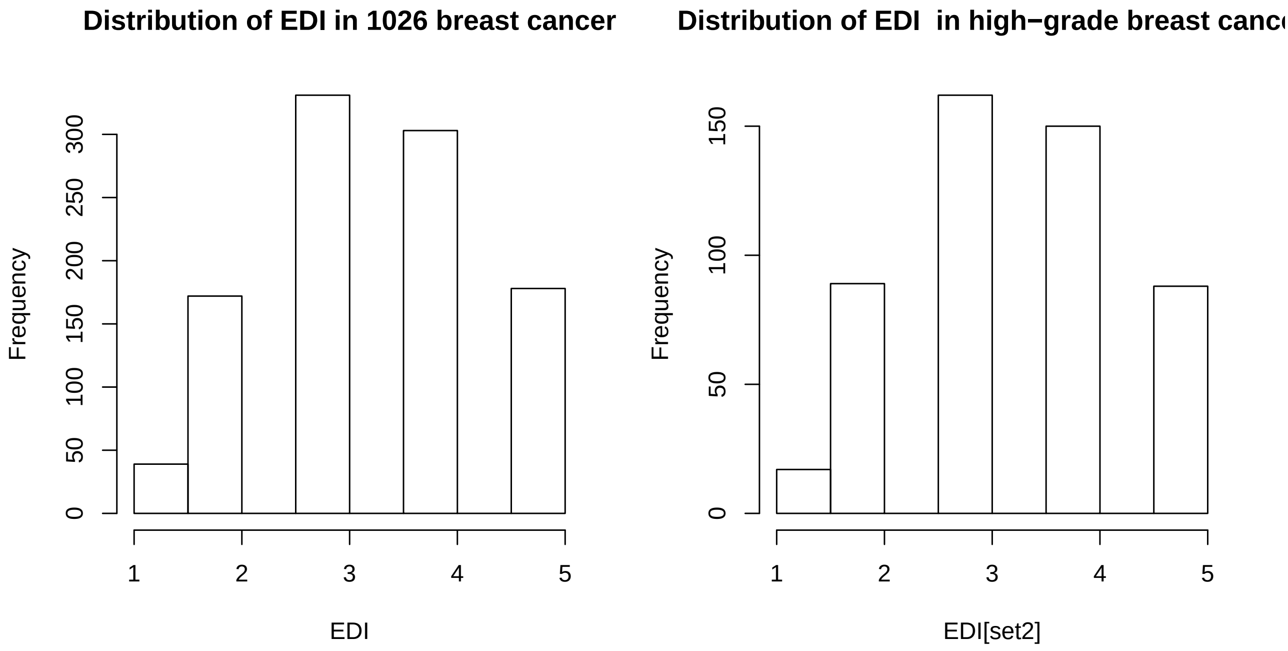

We can plot Grade 3 tumor composition as histograms:

```
Col = c("black", "white")
par(mfrow = c(2, 2))
x = prop.table(table(EDI5[set2], trait$node[set2]), 1)
x <- x[, colSums(x) > 0.05]
barplot((x), beside = T, las = 3, col = Col, ylim = c(0, 1), names.arg = colnames(x),
    main = "Node")
legend("topleft", legend = c("Others", "EDI5"), col = Col, pch = 15)
x = prop.table(table(EDI5[set2], trait$size[set2]), 1)
x <- x[, colSums(x) > 0.05]
barplot((x), beside = T, las = 3, col = Col, ylim = c(0, 1), names.arg = colnames(x),
    main = "Size")
x = prop.table(table(EDI5[set2], trait$er[set2]), 1)
x <- x[, colSums(x) > 0.05]
barplot((x), beside = T, las = 3, col = Col, ylim = c(0, 1), names.arg = colnames(x),
```

```
    main = "ER")
pam50 <- trait$Pam50Subtype
pam50[pam50 == "NC"] <- NA
x = prop.table(table(EDI5[set2], pam50[set2]), 1)
x <- x[, colSums(x) > 0.05]
barplot(t(x), beside = T, las = 3, col = pam50colors[colnames(x)], ylim = c(0,
    1), main = "PAM50", names.arg = c("EDI-low", "EDI-high"))
legend("topleft", legend = names(pam50colors), col = pam50colors, pch = 15)
```

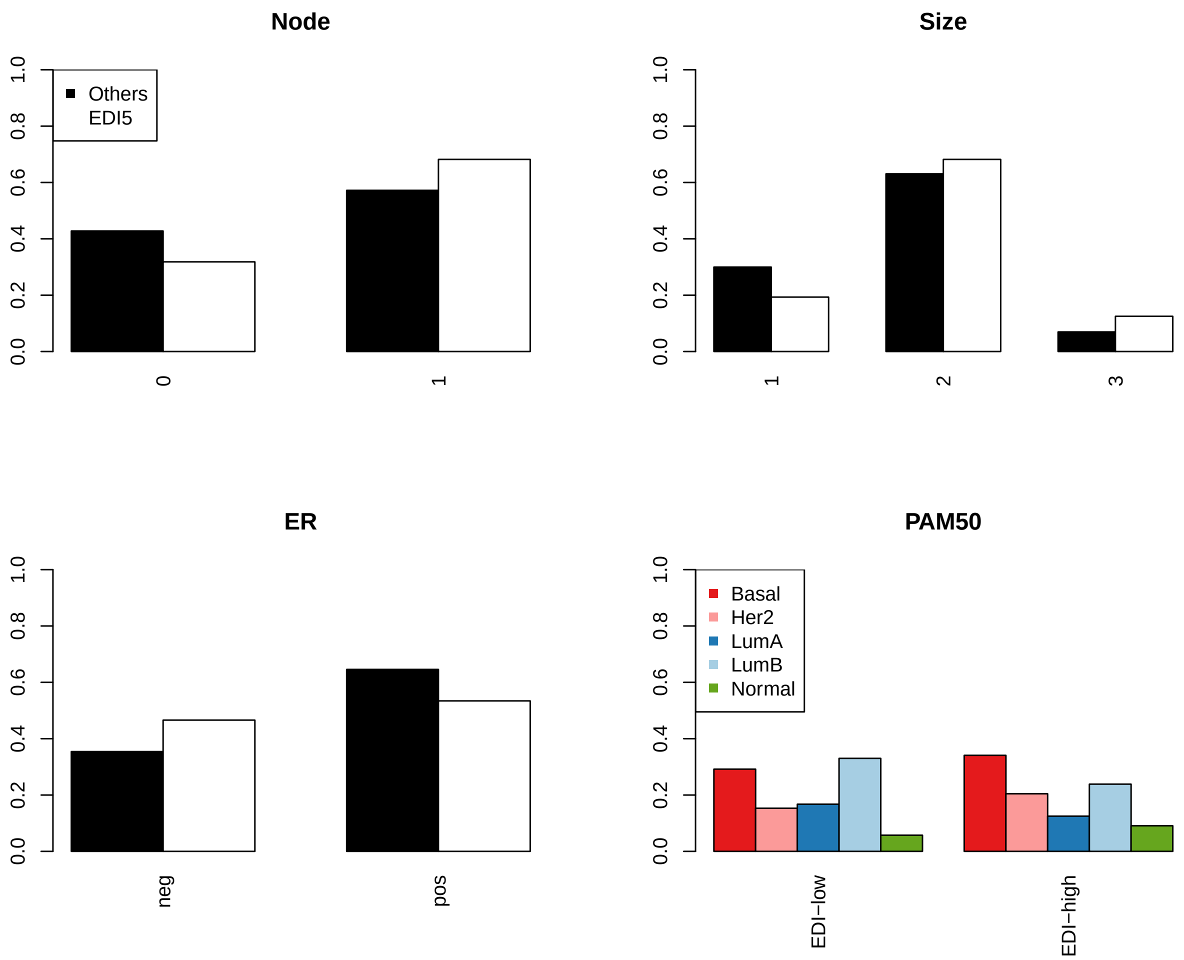

2.2 Correlation between EDI and clinicopathological parameters

Here we examine the correlation between EDI with nine different clinicopathological parameters of the breast tumors. We test each subtype individually with Fisher's test to see if there is any specific subtype enriched in EDI5.

```
Pval <- NULL
N <- NULL
x <- EDI5[set2]
dat <- data.frame(trait$size, trait$node, trait$ER.Expr, trait$TP53, trait$Her2.SNP6 ==
    2, trait$Pam50Subtype, trait$IntClustMemb)[set2, ]
colnames(dat) <- c("Size", "Node", "ER", "TP53", "Her2", "Pam50", "IntClust")
for (i in 1:ncol(dat)) {
    s <- dat[, i]
    S <- sort(unique(s))
    S <- S[!is.na(S)]
```

```r
    for (j in 1:length(S)) {
        z <- s == S[j]
        Pval <- c(Pval, phyper(sum(x & z, na.rm = T), sum(x, na.rm = T), length(x) -
            sum(x, na.rm = T), sum(z, na.rm = T), lower.tail = F))
        N <- c(N, paste(colnames(dat)[i], S[j]))
    }
}
par(mar = c(7, 4, 0, 0))
plot(-log(Pval), xaxt = "n", xlab = "", pch = 19)
abline(h = -log(0.05))
axis(1, at = 1:length(Pval), labels = N, las = 2)
```

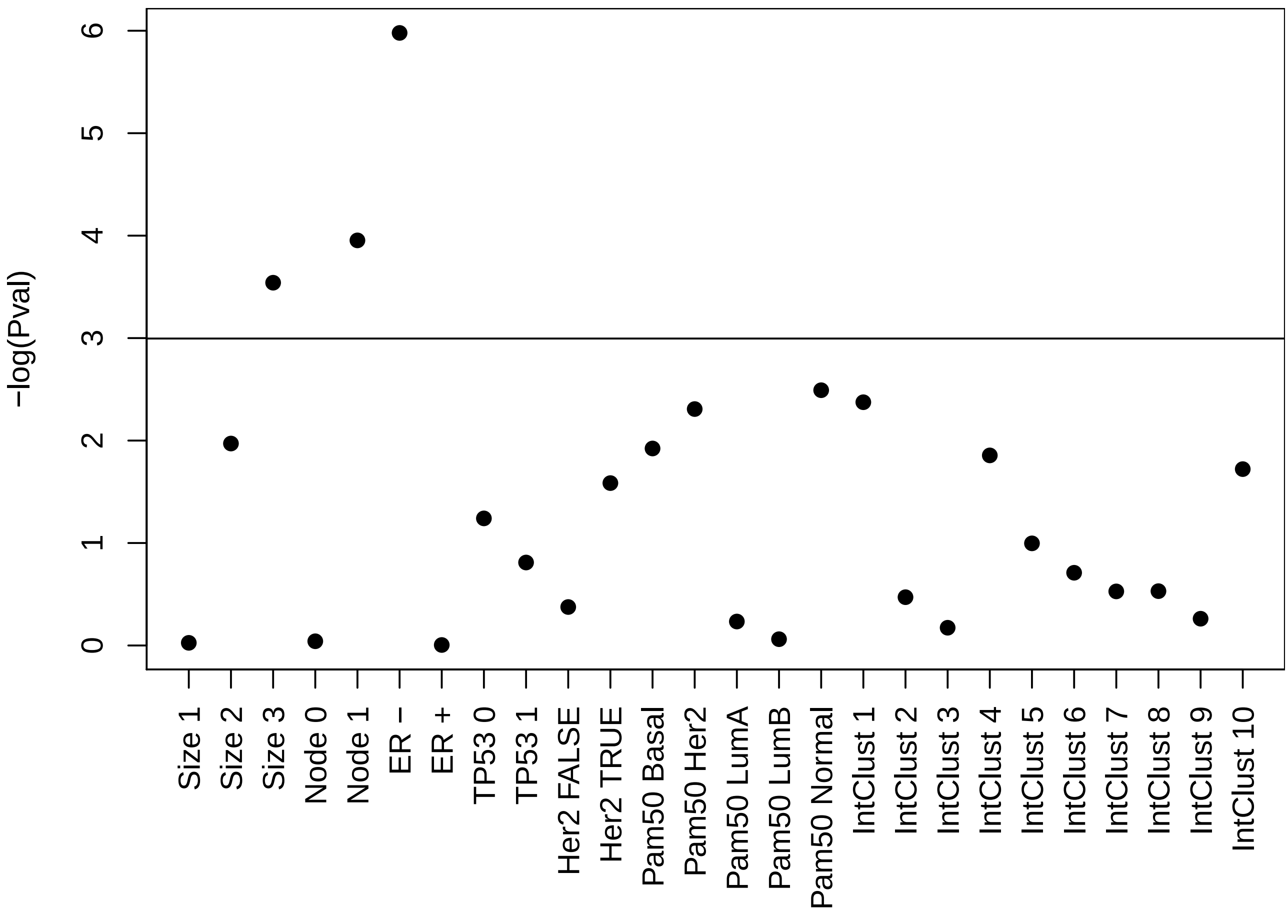

Thus, in the size 3, Node positive, ER- subtypes, here is an enrichment of EDI5 group.

2.3 Correlation between EDI and tumor composition

EDI/EDI5 is not associated with abundance of specific cell types defined by image analysis or pathological scores.

```r
par(mfrow = c(2, 2))
set2 <- grepl(3, trait$grade)
Boxplot(trait$cancer[set2] ~ EDI5[set2], ylab = "Cancer", xlab = "EDI5")
Boxplot(trait$stromal[set2] ~ EDI5[set2], ylab = "Stromal", xlab = "EDI5")
Boxplot(trait$lym[set2] ~ EDI5[set2], ylab = "Lymphocyte", xlab = "EDI5")
Boxplot(EDI[set2] ~ trait$Lymphocyte.infiltration[set2], ylab = "EDI", xlab = "Pathological Ly
```

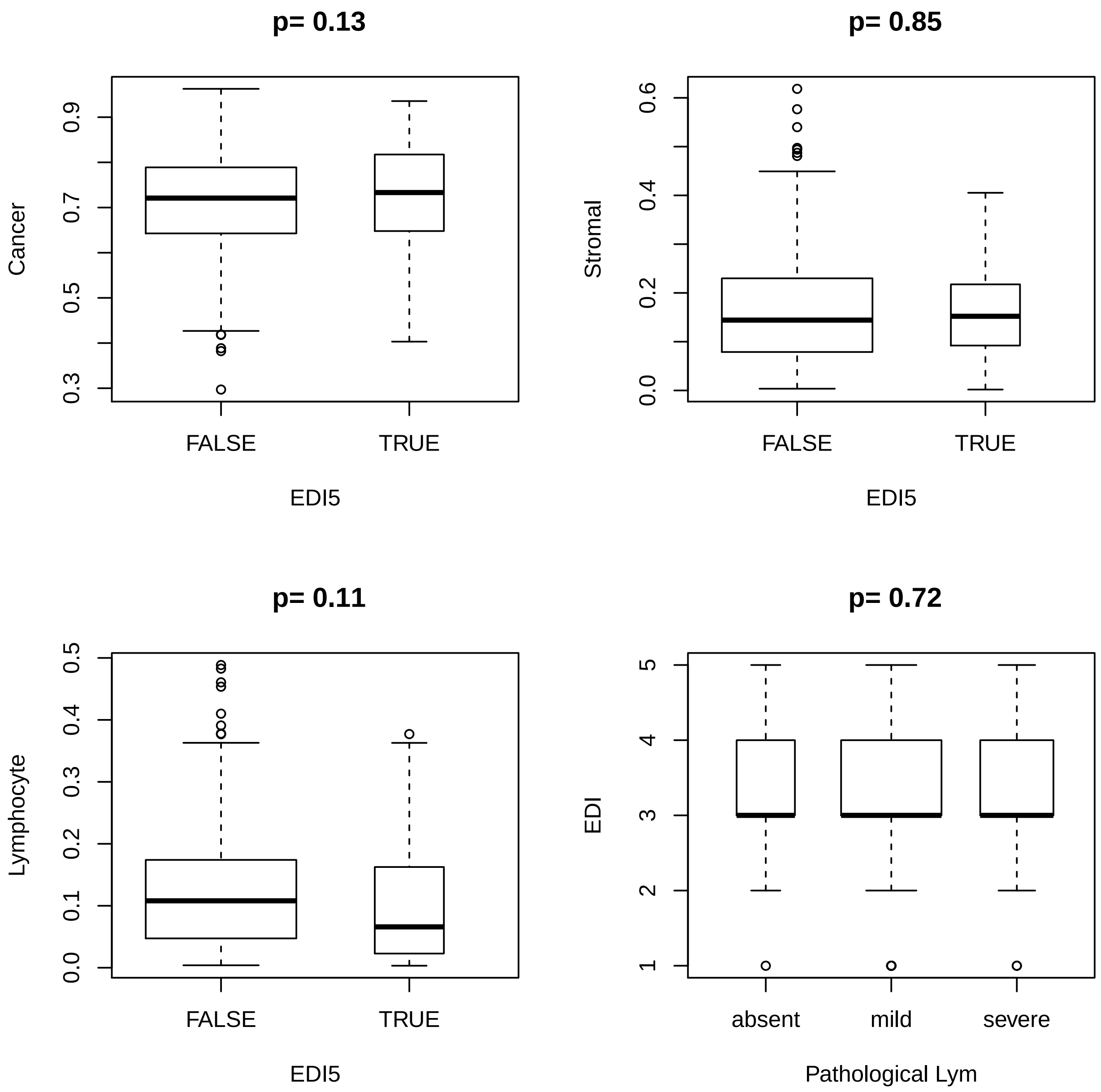

3 Association between EDI and breast cancer prognosis

3.1 Survival data in METABRIC cohorts

The summary statistics of breast tumors in our cohort are given below. For survival data we focused on disease-specific survival (DSS) within 10 years from diagnosis.

```
summary(trait$S_10year)

##       time            status     
##  Min.   :  0.27   Min.   :0.000  
##  1st Qu.: 45.52   1st Qu.:0.000  
##  Median : 72.20   Median :0.000  
##  Mean   : 75.25   Mean   :0.181  
##  3rd Qu.:120.00   3rd Qu.:0.000  
##  Max.   :120.00   Max.   :1.000  
##  NA's   :3        NA's   :13
```

The estimated median follow-up time can be calculated by the reverse Kaplan-Meier method. We invert the censoring index for death to estimate time to loss of follow up.

```
library(survival)
survfit(Surv(trait$S_10year[, 1], trait$S_10year[, 2] == 0) ~ 1)

## Call: survfit.formula(formula = Surv(trait$S_10year[, 1], trait$S_10year[,
##     2] == 0) ~ 1)
##
##    14 observations deleted due to missingness
## records   n.max n.start  events  median 0.95LCL 0.95UCL
##   1012.0  1012.0  1012.0   829.0    88.5    83.5    98.2
```

There are 14 censoring events for DSS, and median DSS (shown earlier) will closely approximate median follow up.

3.2 Univariate analysis of prognostic significance of EDI in high- and low-grade tumors

We plot Kaplan-Meier curves of DSS based on EDI5 or EDI of low- or high-grade tumors at a given cohort. Grade is a strong prognostic factor in our cohorts.

```
par(mfrow = c(1, 2))
Site <- list(trait$Site == 1, trait$Site == 2)
set2 <- Site[[1]]
plotSurv(trait$S_10year[set2, ], trait$grade[set2], fileType = "", name = "Grade",
    type = "Site1")
set2 <- Site[[2]]
plotSurv(trait$S_10year[set2, ], trait$grade[set2], fileType = "", name = "Grade",
    type = "Site2")
```

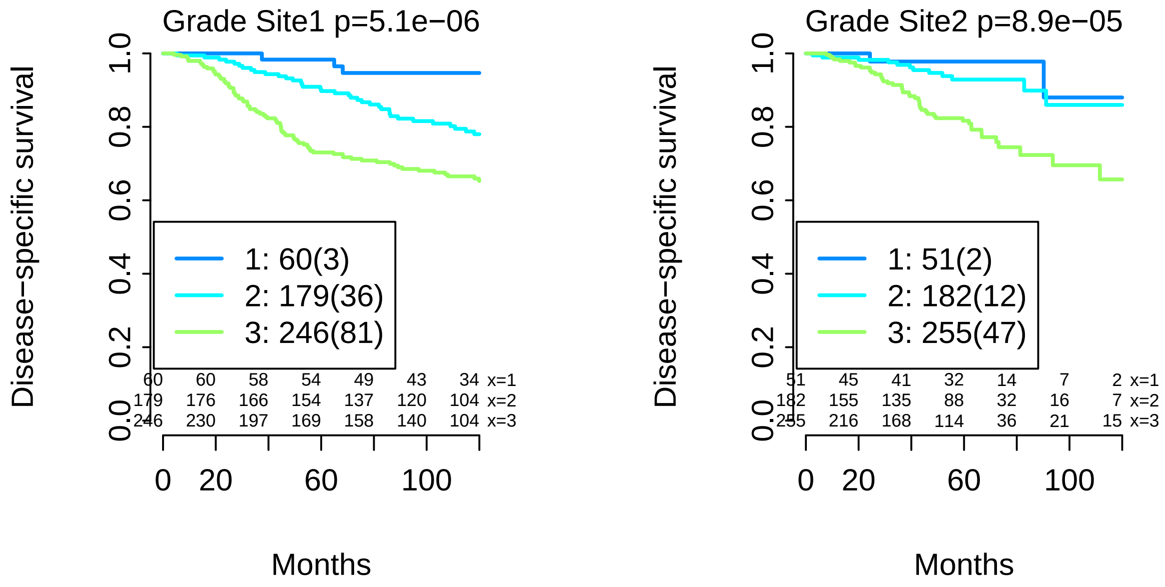

Univariate Cox regression analysis are performed on the high-grade tumors for each sample cohort.

```
set2 <- grepl(3, trait$grade) & Site[[1]]
summary(coxph(trait$S_10year[set2, ] ~ EDI5[set2]))[c(8, 10, 14)]

## $conf.int
##                  exp(coef) exp(-coef) lower .95 upper .95
## EDI5[set2]TRUE     2.011     0.4972     1.265     3.198
##
## $sctest
##    test      df  pvalue
## 9.07580 1.00000 0.00259
##
## $concordance
## concordance.concordant            se.std(c-d)
##                0.57140                0.02302

set2 <- grepl(3, trait$grade) & Site[[2]]
summary(coxph(trait$S_10year[set2, ] ~ EDI5[set2]))[c(8, 10, 14)]

## $conf.int
##                  exp(coef) exp(-coef) lower .95 upper .95
## EDI5[set2]TRUE     2.243     0.4458     1.083     4.646
##
## $sctest
##    test      df  pvalue
## 4.98806 1.00000 0.02552
##
## $concordance
## concordance.concordant            se.std(c-d)
##                0.56106                0.02349
```

Plot Kaplan-Meier (KM) curves for these groups:

```
par(mfrow = c(2, 2))
set2 <- grepl(3, trait$grade) & Site[[1]]
plotSurv(trait$S_10year[set2, ], EDI5[set2], fileType = "", name = "EDI5", type = "Grade 3 Sit
set2 <- grepl(3, trait$grade) & Site[[2]]
plotSurv(trait$S_10year[set2, ], EDI5[set2], fileType = "", name = "EDI5", type = "Grade 3 Sit
set2 <- !grepl(3, trait$grade) & Site[[1]]
plotSurv(trait$S_10year[set2, ], EDI5[set2], fileType = "", name = "EDI5", type = "Grade 1&2 S
set2 <- !grepl(3, trait$grade) & Site[[2]]
plotSurv(trait$S_10year[set2, ], EDI5[set2], fileType = "", name = "EDI5", type = "Grade 1&2 S
```

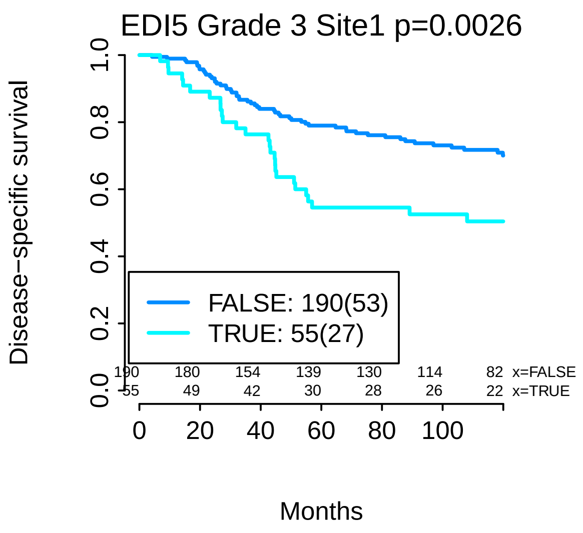

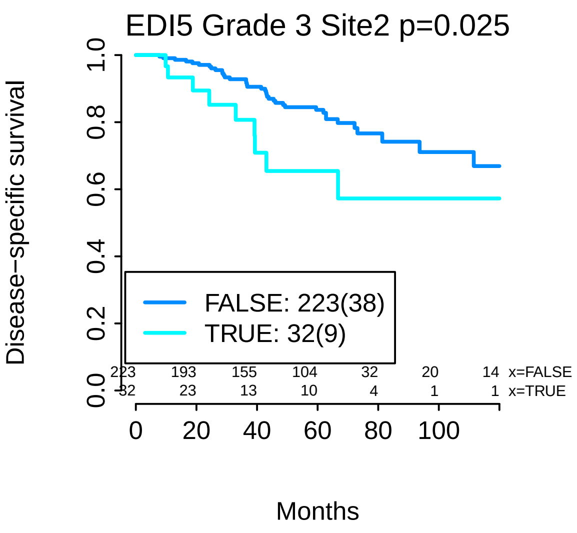

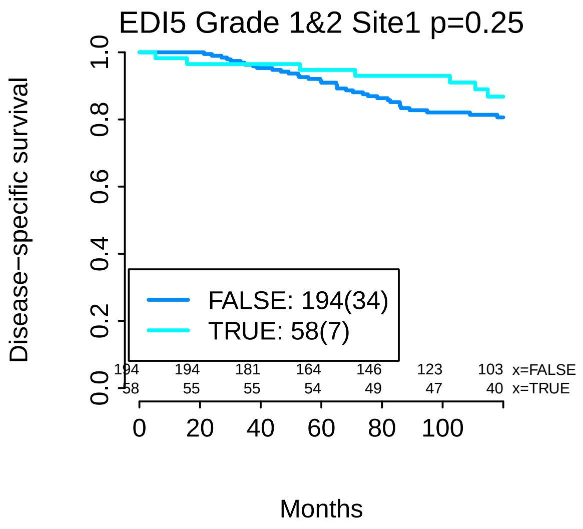

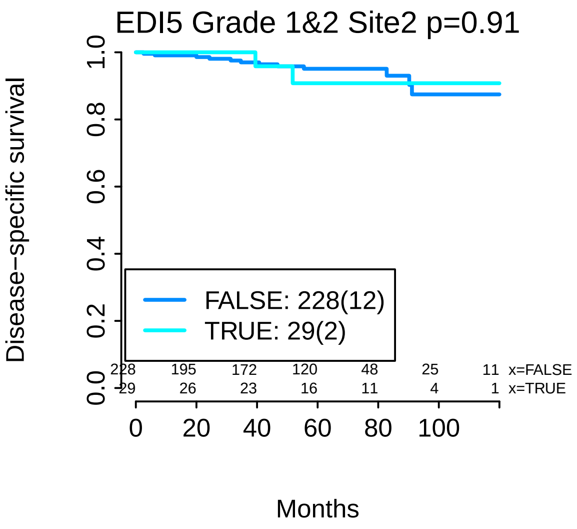


```
par(mfrow = c(2, 2))
Site <- list(trait$Site == 1, trait$Site == 2)
set2 <- grepl(3, trait$grade) & Site[[1]]
plotSurv(trait$S_10year[set2, ], EDI[set2], fileType = "", name = "EDI", type = "Grade 3 Site1
set2 <- grepl(3, trait$grade) & Site[[2]]
plotSurv(trait$S_10year[set2, ], EDI[set2], fileType = "", name = "EDI", type = "Grade 3 Site2
set2 <- !grepl(3, trait$grade) & Site[[1]]
plotSurv(trait$S_10year[set2, ], EDI[set2], fileType = "", name = "EDI", type = "Grade 1&2 Sit
set2 <- !grepl(3, trait$grade) & Site[[2]]
plotSurv(trait$S_10year[set2, ], EDI[set2], fileType = "", name = "EDI", type = "Grade 1&2 Sit
```

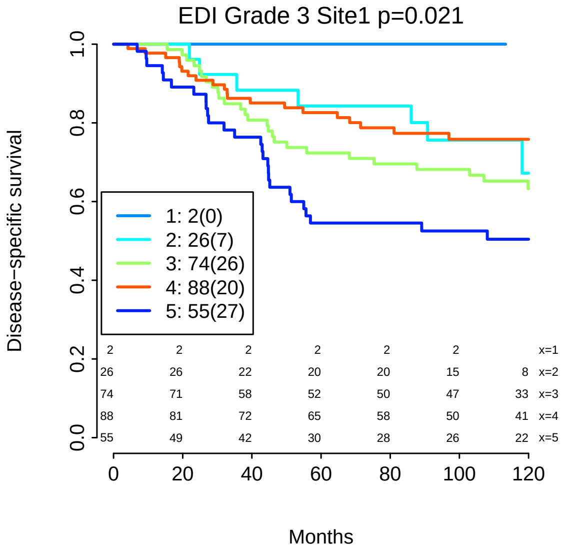

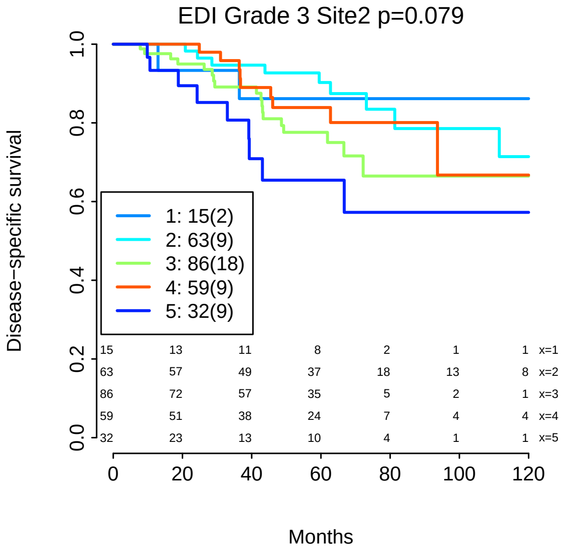

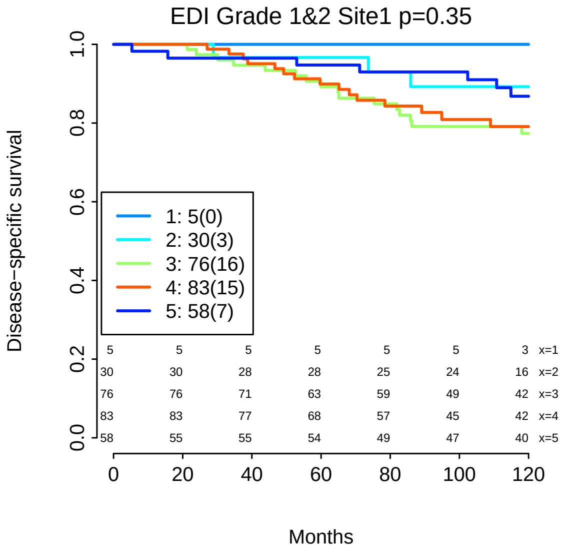

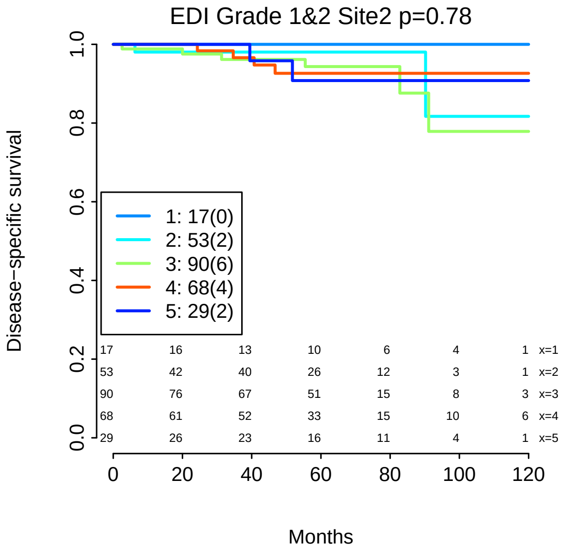

Combining two cohorts, the survival probability for each EDI5 group is:

```
set2 <- grepl(3, trait$grade)
summary(survfit(trait$S_10year[set2 & EDI5] ~ 1))

## Call: survfit.formula(formula = trait$S_10year[set2 & EDI5] ~ 1)
##
## 2 observations deleted due to missingness
##    time n.risk n.event survival std.err lower 95% CI upper 95% CI
##    6.83     86       1    0.988  0.0116        0.966        1.000
##    9.43     84       1    0.977  0.0163        0.945        1.000
##    9.60     83       1    0.965  0.0199        0.927        1.000
##    9.83     82       1    0.953  0.0229        0.909        0.999
##   10.60     81       1    0.941  0.0255        0.893        0.993
##   14.10     78       1    0.929  0.0279        0.876        0.985
##   14.40     77       1    0.917  0.0300        0.860        0.978
##   16.73     74       1    0.905  0.0320        0.844        0.970
##   18.80     73       1    0.892  0.0339        0.828        0.961
```

```
##    23.20     70       1    0.880  0.0358        0.812        0.953
##    24.23     69       1    0.867  0.0374        0.797        0.943
##    26.73     68       1    0.854  0.0390        0.781        0.934
##    26.77     67       1    0.841  0.0404        0.766        0.925
##    27.20     66       1    0.829  0.0418        0.751        0.915
##    27.47     65       1    0.816  0.0431        0.736        0.905
##    31.93     63       1    0.803  0.0443        0.721        0.895
##    32.97     62       1    0.790  0.0454        0.706        0.884
##    35.00     61       1    0.777  0.0465        0.691        0.874
##    39.17     59       1    0.764  0.0475        0.676        0.863
##    39.30     57       1    0.750  0.0485        0.661        0.852
##    42.57     55       1    0.737  0.0495        0.646        0.841
##    42.97     54       1    0.723  0.0505        0.631        0.829
##    43.10     53       1    0.710  0.0513        0.616        0.818
##    43.20     52       1    0.696  0.0521        0.601        0.806
##    44.60     51       1    0.682  0.0529        0.586        0.794
##    44.77     50       1    0.669  0.0535        0.572        0.782
##    44.83     49       1    0.655  0.0541        0.557        0.770
##    45.17     48       1    0.641  0.0547        0.543        0.758
##    51.00     47       1    0.628  0.0552        0.528        0.746
##    51.40     46       1    0.614  0.0557        0.514        0.733
##    55.00     45       1    0.600  0.0561        0.500        0.721
##    55.63     43       1    0.586  0.0565        0.486        0.708
##    56.93     42       1    0.572  0.0569        0.471        0.695
##    66.73     37       1    0.557  0.0574        0.455        0.682
##    89.10     29       1    0.538  0.0585        0.434        0.666
##   108.07     26       1    0.517  0.0598        0.412        0.649
```

```
summary(survfit(trait$S_10year[set2 & !EDI5] ~ 1))
```

```
## Call: survfit.formula(formula = trait$S_10year[set2 & !EDI5] ~ 1)
##
## 6 observations deleted due to missingness
##     time n.risk n.event survival std.err lower 95% CI upper 95% CI
##     4.17    410       1    0.998 0.00244        0.993        1.000
##     7.80    405       1    0.995 0.00346        0.988        1.000
##     9.07    401       1    0.993 0.00425        0.984        1.000
##     9.13    399       1    0.990 0.00491        0.981        1.000
##    12.93    391       1    0.988 0.00551        0.977        0.998
##    15.07    388       1    0.985 0.00606        0.973        0.997
##    15.50    384       1    0.982 0.00656        0.970        0.995
##    16.60    381       1    0.980 0.00703        0.966        0.994
##    18.67    378       1    0.977 0.00748        0.963        0.992
##    19.00    377       1    0.975 0.00789        0.959        0.990
##    19.10    376       1    0.972 0.00829        0.956        0.989
##    19.73    375       1    0.970 0.00866        0.953        0.987
##    19.83    374       1    0.967 0.00902        0.949        0.985
##    20.83    372       1    0.964 0.00936        0.946        0.983
##    21.17    370       1    0.962 0.00969        0.943        0.981
##    21.57    369       1    0.959 0.01001        0.940        0.979
##    21.93    368       1    0.957 0.01032        0.937        0.977
##    23.27    366       1    0.954 0.01061        0.933        0.975
##    23.83    365       1    0.951 0.01090        0.930        0.973
##    24.33    363       1    0.949 0.01118        0.927        0.971
```

```
##    24.87    362        2    0.943 0.01172         0.921          0.967
##    24.90    360        1    0.941 0.01198         0.918          0.965
##    25.43    357        1    0.938 0.01223         0.915          0.962
##    26.27    355        1    0.936 0.01248         0.911          0.960
##    26.77    354        1    0.933 0.01272         0.908          0.958
##    28.50    350        1    0.930 0.01296         0.905          0.956
##    28.57    349        1    0.928 0.01319         0.902          0.954
##    28.60    347        1    0.925 0.01342         0.899          0.952
##    28.73    346        1    0.922 0.01365         0.896          0.949
##    29.00    344        1    0.920 0.01387         0.893          0.947
##    29.33    340        1    0.917 0.01409         0.890          0.945
##    30.17    338        1    0.914 0.01431         0.887          0.943
##    30.43    337        1    0.911 0.01452         0.883          0.940
##    31.00    336        1    0.909 0.01473         0.880          0.938
##    32.03    334        1    0.906 0.01493         0.877          0.936
##    32.07    333        1    0.903 0.01513         0.874          0.933
##    32.83    331        1    0.901 0.01533         0.871          0.931
##    32.93    330        1    0.898 0.01553         0.868          0.929
##    35.63    326        1    0.895 0.01572         0.865          0.926
##    36.40    324        1    0.892 0.01591         0.862          0.924
##    36.43    323        1    0.890 0.01610         0.859          0.922
##    36.63    321        1    0.887 0.01629         0.855          0.919
##    36.77    320        2    0.881 0.01665         0.849          0.914
##    38.03    316        1    0.878 0.01683         0.846          0.912
##    38.80    313        1    0.876 0.01701         0.843          0.910
##    39.53    310        1    0.873 0.01719         0.840          0.907
##    41.37    308        1    0.870 0.01736         0.837          0.905
##    42.70    306        1    0.867 0.01754         0.833          0.902
##    42.97    305        1    0.864 0.01771         0.830          0.900
##    43.13    302        1    0.861 0.01788         0.827          0.897
##    43.30    299        1    0.859 0.01805         0.824          0.895
##    43.83    298        1    0.856 0.01822         0.821          0.892
##    44.40    296        1    0.853 0.01838         0.817          0.890
##    44.73    294        1    0.850 0.01855         0.814          0.887
##    45.50    292        1    0.847 0.01871         0.811          0.884
##    45.93    291        1    0.844 0.01887         0.808          0.882
##    46.07    290        1    0.841 0.01903         0.805          0.879
##    46.43    287        1    0.838 0.01919         0.801          0.877
##    48.60    282        1    0.835 0.01935         0.798          0.874
##    49.23    281        1    0.832 0.01951         0.795          0.871
##    49.47    278        1    0.829 0.01967         0.792          0.869
##    50.10    276        1    0.826 0.01982         0.788          0.866
##    53.37    266        1    0.823 0.01999         0.785          0.863
##    54.77    263        1    0.820 0.02016         0.781          0.860
##    55.83    257        1    0.817 0.02033         0.778          0.858
##    59.50    246        1    0.813 0.02052         0.774          0.855
##    61.90    234        1    0.810 0.02072         0.770          0.852
##    62.77    227        2    0.803 0.02115         0.762          0.845
##    64.70    214        1    0.799 0.02138         0.758          0.842
##    66.63    208        1    0.795 0.02162         0.754          0.839
##    68.20    200        1    0.791 0.02187         0.750          0.835
##    68.27    199        1    0.787 0.02212         0.745          0.832
##    71.43    188        1    0.783 0.02240         0.740          0.828
```

```
##   72.20    186        1    0.779 0.02267        0.736        0.825
##   73.13    182        1    0.775 0.02294        0.731        0.821
##   75.33    178        1    0.770 0.02322        0.726        0.817
##   81.10    159        1    0.765 0.02358        0.721        0.813
##   81.33    158        1    0.761 0.02392        0.715        0.809
##   86.07    152        1    0.756 0.02428        0.709        0.805
##   87.70    149        1    0.751 0.02464        0.704        0.800
##   90.80    146        1    0.745 0.02500        0.698        0.796
##   93.67    142        1    0.740 0.02537        0.692        0.792
##   96.97    141        1    0.735 0.02573        0.686        0.787
##  102.97    130        1    0.729 0.02615        0.680        0.782
##  107.10    126        1    0.723 0.02657        0.673        0.777
##  111.53    116        1    0.717 0.02706        0.666        0.772
##  118.13    100        1    0.710 0.02773        0.658        0.767
##  119.87     97        1    0.703 0.02839        0.649        0.761
```

```
summary(coxph(trait$S_10year[set2, ] ~ EDI5[set2]))[c(8, 10, 14)]
```

```
## $conf.int
##                 exp(coef) exp(-coef) lower .95 upper .95
## EDI5[set2]TRUE      2.129     0.4697     1.447     3.133
##
## $sctest
##      test        df    pvalue
## 1.543e+01 1.000e+00 8.572e-05
##
## $concordance
## concordance.concordant            se.std(c-d)
##                0.57238                0.01679
```

3.3 Univariate analysis of prognostic significance of existing clinicopathological parameters and tumor composition in high-grade tumors

3.3.1 Clinicopathological parameters including node, size and genomic subtypes

Now we examine the correlation between survival and known clinicopathological parameters and subtypes including Pam50 and IntClust in both cohorts in high grade tumors. First, we visualize the analysis with KM curves.

```
par(mfrow = c(2, 4), mar = c(4, 5, 3, 0))
set2 <- grepl(3, trait$grade)
plotSurv(trait$S_10year[set2 & Site[[1]]], trait$node[set2 & Site[[1]]], name = "Node",
    type = "Site 1")
plotSurv(trait$S_10year[set2 & Site[[1]]], trait$size[set2 & Site[[1]]], name = "Size",
    type = "Site 1")
plotSurv(trait$S_10year[set2 & Site[[1]]], trait$Pam50Subtype[set2 & Site[[1]]],
    name = "PAM50", type = "Site 1", col = pam50colors)
plotSurv(trait$S_10year[set2 & Site[[1]]], trait$IntClustMemb[set2 & Site[[1]]],
    name = "IntClust", type = "Site 1", col = col)
plotSurv(trait$S_10year[set2 & Site[[2]]], trait$node[set2 & Site[[2]]], name = "Node",
    type = "Site 2")
plotSurv(trait$S_10year[set2 & Site[[2]]], trait$size[set2 & Site[[2]]], name = "Size",
    type = "Site 2")
```

```
plotSurv(trait$S_10year[set2 & Site[[2]]], trait$Pam50Subtype[set2 & Site[[2]]],
    name = "PAM50", type = "Site 2", col = pam50colors)
plotSurv(trait$S_10year[set2 & Site[[2]]], trait$IntClustMemb[set2 & Site[[2]]],
    name = "IntClust", type = "Site 2", col = col)
```

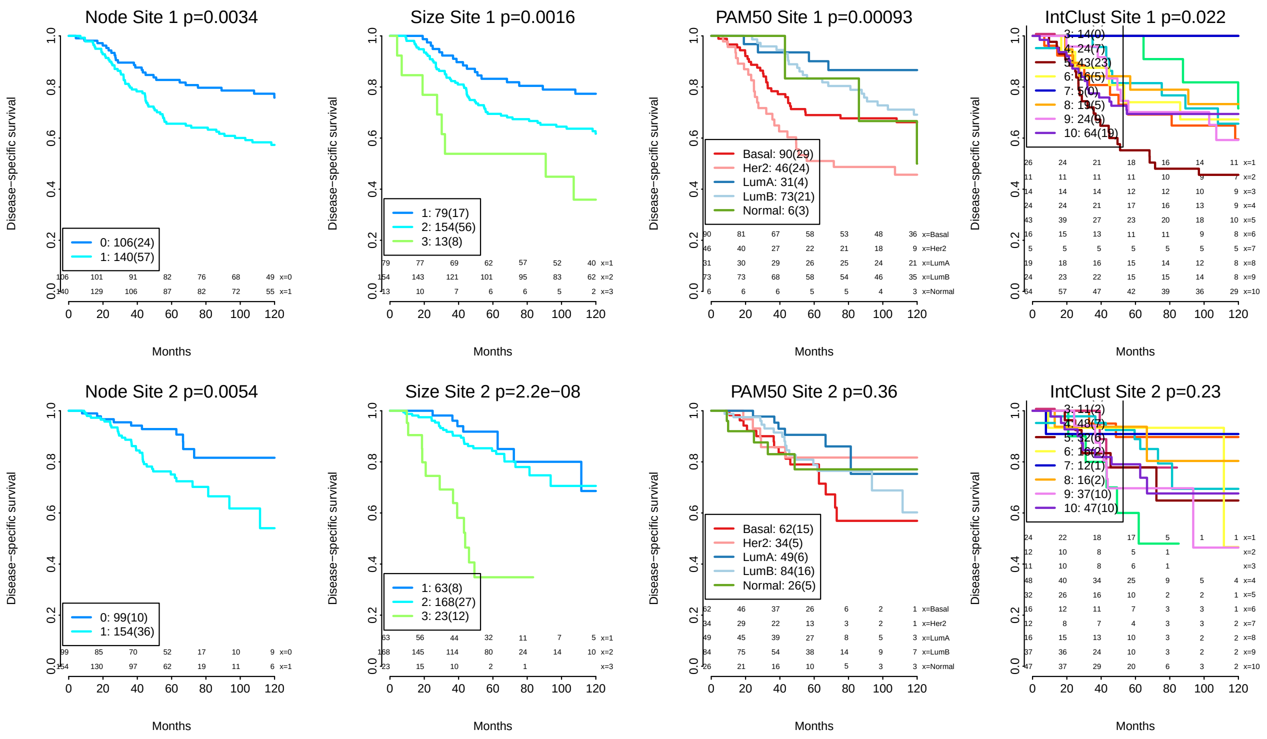

We then use univariate Cox regression analysis to test the correlation between these parameters with survival, as reported in Table 2.

```
set2 <- grepl(3, trait$grade) & Site[[1]]
summary(coxph(trait$S_10year[set2, ] ~ trait$node[set2]))[c(8, 10, 14)]

## $conf.int
##                  exp(coef) exp(-coef) lower .95 upper .95
## trait$node[set2]      2.01     0.4974     1.247      3.24
##
## $sctest
##      test       df    pvalue
## 8.564514 1.000000 0.003428
##
## $concordance
## concordance.concordant            se.std(c-d)
##                0.58006                0.02817

summary(coxph(trait$S_10year[set2, ] ~ trait$size[set2]))[c(8, 10, 14)]

## $conf.int
##                  exp(coef) exp(-coef) lower .95 upper .95
## trait$size[set2]      2.01     0.4974      1.33     3.038
##
## $sctest
##       test        df    pvalue
## 1.103e+01 1.000e+00 8.963e-04
##
```

```
## $concordance
## concordance.concordant            se.std(c-d)
##                 0.58945               0.02785

set2 <- grepl(3, trait$grade) & Site[[2]]
summary(coxph(trait$S_10year[set2, ] ~ trait$node[set2]))[c(8, 10, 14)]

## $conf.int
##                  exp(coef) exp(-coef) lower .95 upper .95
## trait$node[set2]     2.608     0.3834     1.293     5.261
##
## $sctest
##     test       df   pvalue
## 7.734070 1.000000 0.005419
##
## $concordance
## concordance.concordant            se.std(c-d)
##                 0.60249               0.03882

summary(coxph(trait$S_10year[set2, ] ~ trait$size[set2]))[c(8, 10, 14)]

## $conf.int
##                  exp(coef) exp(-coef) lower .95 upper .95
## trait$size[set2]     3.031       0.33     1.732     5.303
##
## $sctest
##      test         df    pvalue
## 1.472e+01 1.000e+00 1.247e-04
##
## $concordance
## concordance.concordant            se.std(c-d)
##                 0.64844               0.03714
```

We found that only node and size are significantly associated with survival in both cohorts in this analysis.

3.3.2 ER and HER2 status measured by IHC and microarrays

First, we compare ER and Her2 status measured by IHC or microarrays.

```
par(mfrow = c(4, 3), mar = c(4, 5, 3, 0))
Her2 <- trait$Her2.SNP6 == 2
Her2IHC <- trait$her2 == 3
Her2IHC[trait$her2 == "null"] <- NA
set2 <- grepl(3, trait$grade) & Site[[1]]
plotSurv(trait$S_10year[set2], trait$er[set2], name = "ER IHC", type = "Site 1")
plotSurv(trait$S_10year[set2], trait$ER.Expr[set2], name = "ER Expr", type = "Site 1")
plotSurv(trait$S_10year[set2], Her2IHC[set2], name = "HER2 IHC", type = "Site 1")
plotSurv(trait$S_10year[set2], Her2[set2], name = "Her2 SNP6", type = "Site 1")
plotSurv(trait$S_10year[set2], trait$Her2.Expr[set2], name = "Her2 Expr", type = "Site 1")
plot(0, 0)
set2 <- grepl(3, trait$grade) & Site[[2]]
plotSurv(trait$S_10year[set2], trait$er[set2], name = "ER IHC", type = "Site 2")
plotSurv(trait$S_10year[set2], trait$ER.Expr[set2], name = "ER Expr", type = "Site 2")
```

```
plotSurv(trait$S_10year[set2], Her2IHC[set2], name = "HER2 IHC", type = "Site 2")
plotSurv(trait$S_10year[set2], Her2[set2], name = "Her2 SNP6", type = "Site 2")
plotSurv(trait$S_10year[set2], trait$Her2.Expr[set2], name = "Her2 Expr", type = "Site 2")
```

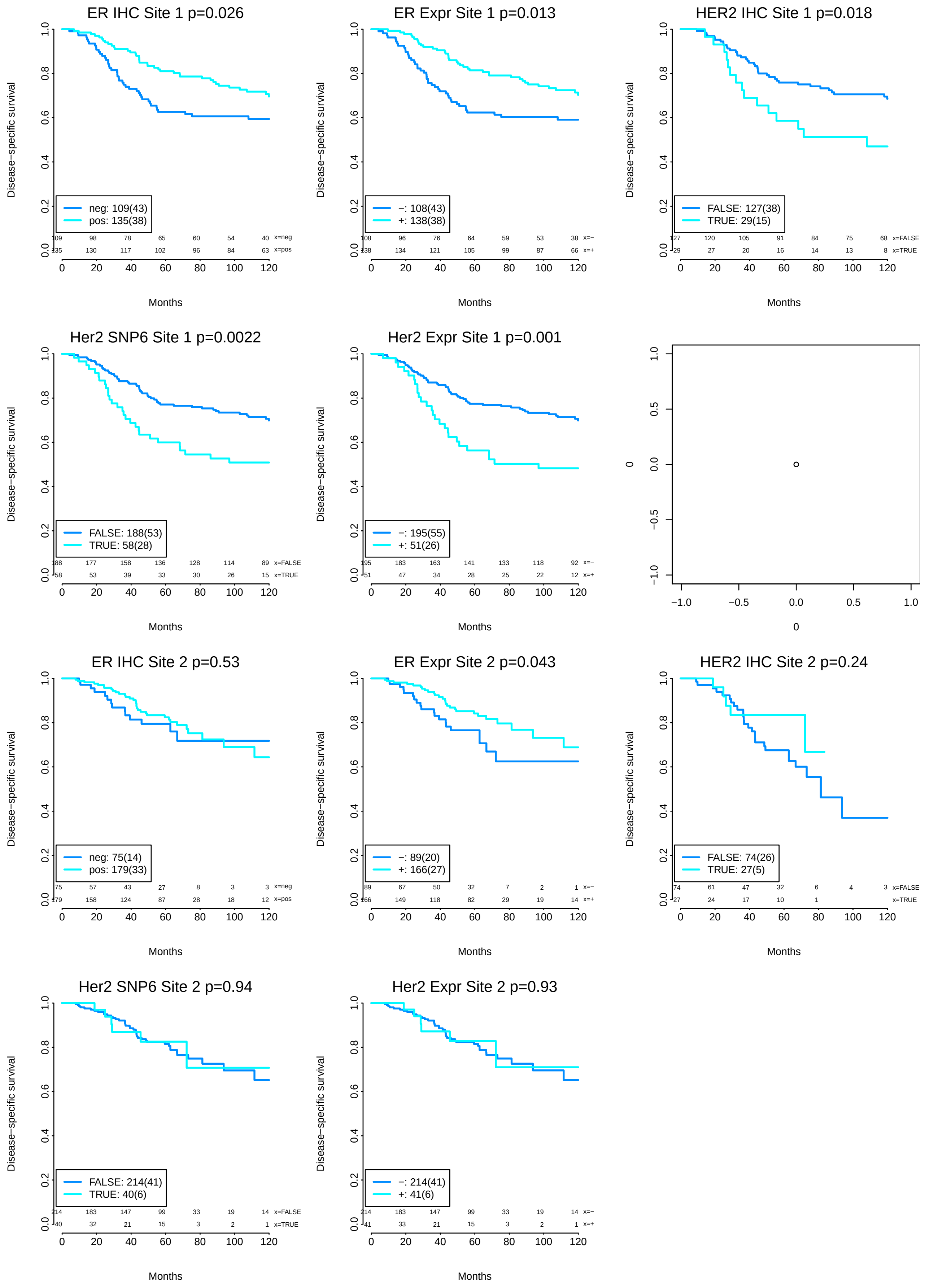

Only ER status measured by RNA expression microarray is correlated with survival in both sites. Also, due to missing data in the Her2 IHC status, we will use Her2 status measured by SNP6 microarray from now on. Next, univariate Cox regression model confirms this.

```
set2 <- grepl(3, trait$grade) & Site[[1]]
summary(coxph(trait$S_10year[set2, ] ~ trait$ER.Expr[set2]))[c(8, 10, 14)]

## $conf.int
##                      exp(coef) exp(-coef) lower .95 upper .95
## trait$ER.Expr[set2]+    0.5795      1.725    0.3745    0.8969
##
## $sctest
##   test     df pvalue
## 6.1425 1.0000 0.0132
##
## $concordance
## concordance.concordant            se.std(c-d)
##               0.58224                0.02774

summary(coxph(trait$S_10year[set2, ] ~ Her2[set2]))[c(8, 10, 14)]

## $conf.int
##                exp(coef) exp(-coef) lower .95 upper .95
## Her2[set2]TRUE     2.019     0.4952     1.277     3.194
##
## $sctest
##    test      df  pvalue
## 9.39971 1.00000 0.00217
##
## $concordance
## concordance.concordant            se.std(c-d)
##               0.57424                0.02316

set2 <- grepl(3, trait$grade) & Site[[2]]
summary(coxph(trait$S_10year[set2, ] ~ trait$ER.Expr[set2]))[c(8, 10, 14)]

## $conf.int
##                      exp(coef) exp(-coef) lower .95 upper .95
## trait$ER.Expr[set2]+    0.5515      1.813    0.3079    0.9879
##
## $sctest
##    test      df  pvalue
## 4.12124 1.00000 0.04235
##
## $concordance
## concordance.concordant            se.std(c-d)
##               0.57593                0.03617

summary(coxph(trait$S_10year[set2, ] ~ Her2[set2]))[c(8, 10, 14)]

## $conf.int
##                exp(coef) exp(-coef) lower .95 upper .95
## Her2[set2]TRUE    0.9696      1.031    0.4112     2.286
##
## $sctest
```

```
##     test       df   pvalue
## 0.004993 1.000000 0.943668
##
## $concordance
## concordance.concordant            se.std(c-d)
##                0.50086                0.02704
```

Merging these to generate supplementary figure 5.

```
par(mfrow = c(4, 3), mar = c(4, 5, 3, 0))
set2 <- grepl(3, trait$grade)
plotSurv(trait$S_10year[set2 & Site[[1]]], trait$node[set2 & Site[[1]]], name = "Node",
    type = "Site 1")
plotSurv(trait$S_10year[set2 & Site[[1]]], trait$size[set2 & Site[[1]]], name = "Size",
    type = "Site 1")
plotSurv(trait$S_10year[set2 & Site[[1]]], trait$Pam50Subtype[set2 & Site[[1]]],
    name = "PAM50", type = "Site 1", col = pam50colors)
plotSurv(trait$S_10year[set2 & Site[[1]]], trait$IntClustMemb[set2 & Site[[1]]],
    name = "IntClust", type = "Site 1", col = col)
plotSurv(trait$S_10year[set2 & Site[[1]]], trait$er[set2 & Site[[1]]], name = "ER IHC",
    type = "Site 1")
plotSurv(trait$S_10year[set2 & Site[[1]]], trait$Her2.Expr[set2 & Site[[1]]],
    name = "Her2 Expr", type = "Site 1")

plotSurv(trait$S_10year[set2 & Site[[2]]], trait$node[set2 & Site[[2]]], name = "Node",
    type = "Site 2")
plotSurv(trait$S_10year[set2 & Site[[2]]], trait$size[set2 & Site[[2]]], name = "Size",
    type = "Site 2")
plotSurv(trait$S_10year[set2 & Site[[2]]], trait$Pam50Subtype[set2 & Site[[2]]],
    name = "PAM50", type = "Site 2", col = pam50colors)
plotSurv(trait$S_10year[set2 & Site[[2]]], trait$IntClustMemb[set2 & Site[[2]]],
    name = "IntClust", type = "Site 2", col = col)
plotSurv(trait$S_10year[set2 & Site[[1]]], trait$er[set2 & Site[[1]]], name = "ER IHC",
    type = "Site 2")
plotSurv(trait$S_10year[set2 & Site[[1]]], trait$Her2.Expr[set2 & Site[[1]]],
    name = "Her2 Expr", type = "Site 2")
```

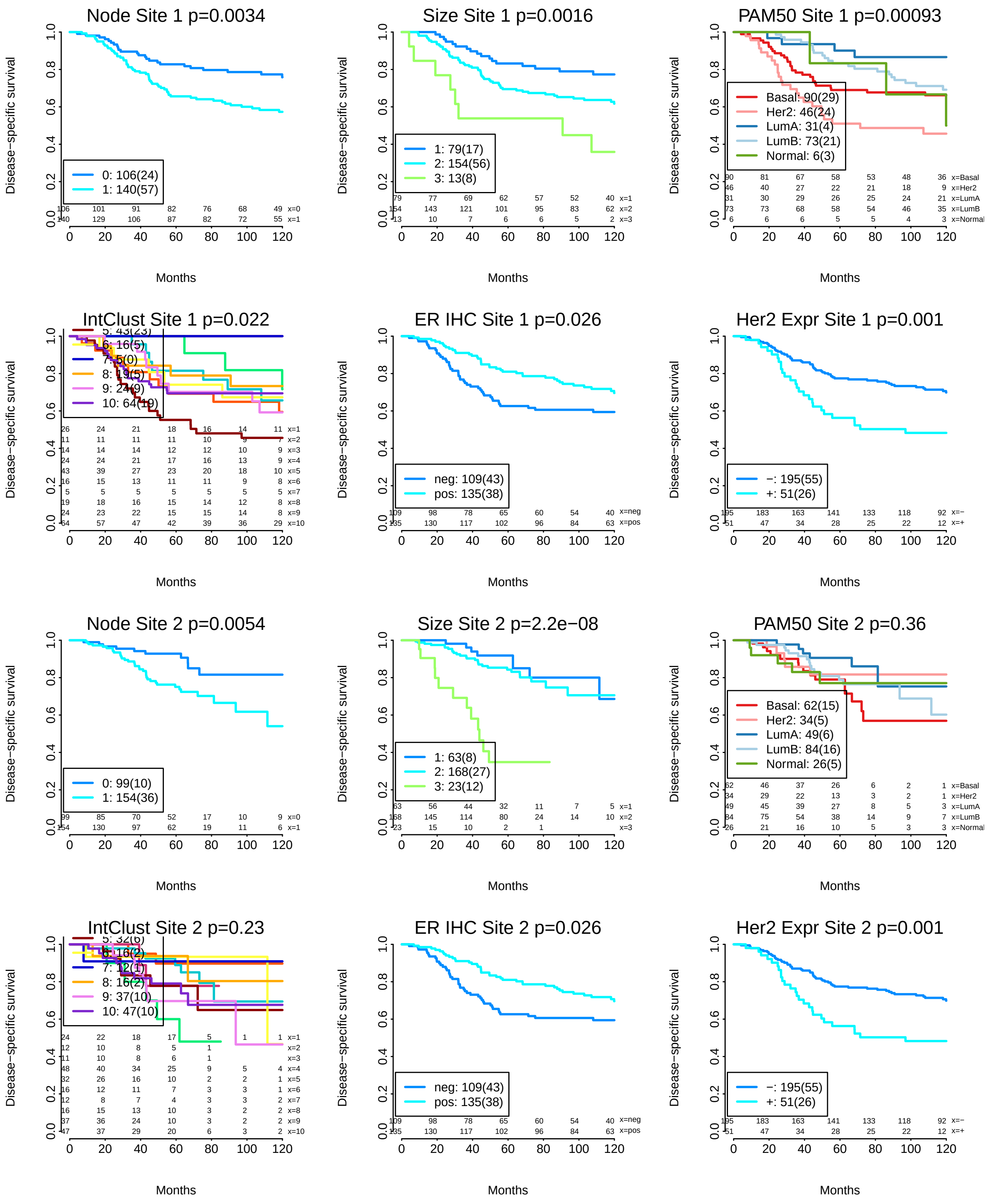

3.3.3 Cell proportions determined by image analysis and pathological scores

We first focus on the percentage of cancer and normal cells based on image analysis and pathological scores. First, for cancer cells:

```
set2 <- grepl(3, trait$grade)
summary(coxph(trait$S_10year[set2, ] ~ trait$cancer[set2]))[c(8, 10, 14)]

## $conf.int
```

```
##                      exp(coef) exp(-coef) lower .95 upper .95
## trait$cancer[set2]      1.09     0.9176    0.2331     5.096
##
## $sctest
##    test      df  pvalue
## 0.01195 1.00000 0.91295
##
## $concordance
## concordance.concordant            se.std(c-d)
##                0.51882                0.02649
```

```
summary(coxph(trait$S_10year[set2, ] ~ group2(trait$cancer[set2])))[c(8, 10,
    14)]
```

```
## $conf.int
##                              exp(coef) exp(-coef) lower .95 upper .95
## group2(trait$cancer[set2])     1.079     0.9264    0.7618      1.53
##
## $sctest
##   test     df pvalue
## 0.1851 1.0000 0.6671
##
## $concordance
## concordance.concordant            se.std(c-d)
##                0.52124                0.02292
```

```
summary(coxph(trait$S_10year[set2, ] ~ group3(trait$cancer[set2])))[c(8, 10,
    14)]
```

```
## $conf.int
##                              exp(coef) exp(-coef) lower .95 upper .95
## group3(trait$cancer[set2])     1.041     0.9609    0.8144      1.33
##
## $sctest
##   test     df pvalue
## 0.1015 1.0000 0.7500
##
## $concordance
## concordance.concordant            se.std(c-d)
##                0.52346                0.02434
```

```
cancer <- trait$cancer[set2] > sort(trait$cancer[set2], decreasing = TRUE)[88]
summary(coxph(trait$S_10year[set2, ] ~ cancer))[c(8, 10, 14)]
```

```
## $conf.int
##            exp(coef) exp(-coef) lower .95 upper .95
## cancerTRUE    0.9364      1.068    0.5957     1.472
##
## $sctest
##   test     df pvalue
## 0.0810 1.0000 0.7759
##
## $concordance
## concordance.concordant            se.std(c-d)
##                0.50016                0.01763
```

Cancer cell proportion as a continuous variable is not associated with survival. When patients are divided into two equal size groups or three groups of lower 25%, middle, 50% and higher 25% based on cancer proportion, the grouping is still not prognostic. Since there is a positive correlation between cancer and EDI, we take the patient group with highest cancer proportion of the same size as the EDI5 group (88 patients). There is still no correlation between this group and survival data. Similarly for stromal cells:

```
set2 <- grepl(3, trait$grade)
summary(coxph(trait$S_10year[set2, ] ~ trait$stromal[set2]))[c(8, 10, 14)]

## $conf.int
##                     exp(coef) exp(-coef) lower .95 upper .95
## trait$stromal[set2]     3.533     0.2831    0.7151     17.45
##
## $sctest
##   test     df pvalue
## 2.4005 1.0000 0.1213
##
## $concordance
## concordance.concordant            se.std(c-d)
##                0.52960                0.02649

summary(coxph(trait$S_10year[set2, ] ~ group2(trait$stromal[set2])))[c(8, 10,
    14)]

## $conf.int
##                             exp(coef) exp(-coef) lower .95 upper .95
## group2(trait$stromal[set2])      1.25     0.8001    0.8828      1.77
##
## $sctest
##   test     df pvalue
## 1.5867 1.0000 0.2078
##
## $concordance
## concordance.concordant            se.std(c-d)
##                0.52785                0.02291

summary(coxph(trait$S_10year[set2, ] ~ group3(trait$stromal[set2])))[c(8, 10,
    14)]

## $conf.int
##                             exp(coef) exp(-coef) lower .95 upper .95
## group3(trait$stromal[set2])      1.11     0.9005    0.8612     1.432
##
## $sctest
##   test     df pvalue
## 0.6529 1.0000 0.4191
##
## $concordance
## concordance.concordant            se.std(c-d)
##                0.51763                0.02416

stromal <- trait$stromal[set2] > sort(trait$stromal[set2], decreasing = TRUE)[88]
summary(coxph(trait$S_10year[set2, ] ~ stromal))[c(8, 10, 14)]
```

```
## $conf.int
##            exp(coef) exp(-coef) lower .95 upper .95
## stromalTRUE     1.114     0.8978    0.6967     1.781
##
## $sctest
##   test     df pvalue
## 0.2031 1.0000 0.6523
##
## $concordance
## concordance.concordant            se.std(c-d)
##                0.50555                0.01685
```

We next focus on the lymphocyte proportion and perform a similar analysis. The best index is lym as a continuous variable, but only borderline significance was found. And this was not confirmed when analysis was performed on individual cohorts.

```
summary(coxph(trait$S_10year[set2, ] ~ trait$lym[set2]))[8:9]

## $conf.int
##                exp(coef) exp(-coef) lower .95 upper .95
## trait$lym[set2]     0.161      6.213    0.0215     1.205
##
## $logtest
##    test      df   pvalue
## 3.37918 1.00000 0.06602

summary(coxph(trait$S_10year[set2, ] ~ group2(trait$lym[set2])))[8:9]

## $conf.int
##                        exp(coef) exp(-coef) lower .95 upper .95
## group2(trait$lym[set2])    0.8509      1.175    0.6009     1.205
##
## $logtest
##   test     df pvalue
## 0.8299 1.0000 0.3623

summary(coxph(trait$S_10year[set2, ] ~ group3(trait$lym[set2])))[8:9]

## $conf.int
##                        exp(coef) exp(-coef) lower .95 upper .95
## group3(trait$lym[set2])    0.8251      1.212    0.6423      1.06
##
## $logtest
##   test     df pvalue
## 2.2702 1.0000 0.1319

lym <- trait$lym[set2] > sort(trait$lym[set2], decreasing = FALSE)[88]
summary(coxph(trait$S_10year[set2, ] ~ lym))[8:9]

## $conf.int
##        exp(coef) exp(-coef) lower .95 upper .95
## lymTRUE    0.6811      1.468    0.4477     1.036
##
## $logtest
##    test      df   pvalue
## 2.98947 1.00000 0.08381
```

```r
set2 <- grepl(3, trait$grade) & Site[[1]]
summary(coxph(trait$S_10year[set2, ] ~ trait$lym[set2]))[8:9]

## $conf.int
##                 exp(coef) exp(-coef) lower .95 upper .95
## trait$lym[set2]    0.2317      4.315   0.01305     4.114
##
## $logtest
##   test     df pvalue
## 1.0507 1.0000 0.3053

set2 <- grepl(3, trait$grade) & Site[[2]]
summary(coxph(trait$S_10year[set2, ] ~ trait$lym[set2]))[8:9]

## $conf.int
##                 exp(coef) exp(-coef) lower .95 upper .95
## trait$lym[set2]    0.1145      8.734  0.005392     2.431
##
## $logtest
##   test     df pvalue
## 2.0506 1.0000 0.1521
```

As summary, from univariate analysis only ER status determined by RNA expression microarray, node status and size are significantly associated with survival in high-grade breast cancer.

3.4 Multivariate analysis of EDI5 and survival-associated clinicopathological parameters in high-grade tumors

Multivariate Cox regression analysis is performed on the high-grade tumors for each sample cohort, considering only variables shown to be associated with survival in univariate analysis.

```r
set2 <- grepl(3, trait$grade) & Site[[1]]
summary(coxph(trait$S_10year[set2, ] ~ EDI5[set2] + trait$node[set2] + trait$size[set2] +
    trait$ER.Expr[set2]))[c(7, 8, 10, 14)]

## $coefficients
##                         coef exp(coef) se(coef)      z Pr(>|z|)
## EDI5[set2]TRUE        0.5577    1.7467   0.2394  2.330 0.019813
## trait$node[set2]      0.4147    1.5140   0.2519  1.647 0.099618
## trait$size[set2]      0.7034    2.0206   0.2225  3.161 0.001573
## trait$ER.Expr[set2]+ -0.4839    0.6164   0.2292 -2.111 0.034779
##
## $conf.int
##                      exp(coef) exp(-coef) lower .95 upper .95
## EDI5[set2]TRUE          1.7467     0.5725    1.0926     2.792
## trait$node[set2]        1.5140     0.6605    0.9241     2.480
## trait$size[set2]        2.0206     0.4949    1.3063     3.125
## trait$ER.Expr[set2]+    0.6164     1.6223    0.3933     0.966
##
## $sctest
##      test        df    pvalue
## 2.829e+01 4.000e+00 1.090e-05
##
## $concordance
```

```
## concordance.concordant            se.std(c-d)
##                0.67189                0.03269
```

```
set2 <- grepl(3, trait$grade) & Site[[2]]
summary(coxph(trait$S_10year[set2, ] ~ EDI5[set2] + trait$node[set2] + trait$size[set2] +
    trait$ER.Expr[set2]))[c(7, 8, 10, 14)]
```

```
## $coefficients
##                          coef  exp(coef) se(coef)      z Pr(>|z|)
## EDI5[set2]TRUE         0.8221    2.2753   0.3767  2.182 0.029097
## trait$node[set2]       0.9020    2.4646   0.3762  2.398 0.016506
## trait$size[set2]       0.8829    2.4180   0.2860  3.087 0.002019
## trait$ER.Expr[set2]+ -0.7984    0.4501   0.3079 -2.593 0.009517
##
## $conf.int
##                      exp(coef) exp(-coef) lower .95 upper .95
## EDI5[set2]TRUE          2.2753     0.4395    1.0873    4.7614
## trait$node[set2]        2.4646     0.4058    1.1790    5.1521
## trait$size[set2]        2.4180     0.4136    1.3805    4.2353
## trait$ER.Expr[set2]+    0.4501     2.2219    0.2461    0.8229
##
## $sctest
##      test        df    pvalue
## 2.802e+01 4.000e+00 1.235e-05
##
## $concordance
## concordance.concordant            se.std(c-d)
##                0.70713                0.04482
```

Thus, in multivariate analysis EDI5, size, ER status remain correlated with survival in both cohorts but not node status. This also means that EDI5 is associated with survival independent to ER status, node and size. We then turn to treatment options: chemotherapy ct, radiotherapy rt, and hormone therapy ht.

```
set2 <- grepl(3, trait$grade) & Site[[1]]
summary(coxph(trait$S_10year[set2, ] ~ EDI5[set2] + trait$ct[set2] + trait$rt[set2] +
    trait$ht[set2]))[c(7, 8, 10, 14)]
```

```
## $coefficients
##                          coef  exp(coef) se(coef)       z  Pr(>|z|)
## EDI5[set2]TRUE       0.6387    1.8941   0.2411  2.6492 0.0080675
## trait$ct[set2]TRUE   1.1837    3.2663   0.3508  3.3744 0.0007396
## trait$rt[set2]TRUE -0.2324    0.7926   0.2695 -0.8624 0.3884428
## trait$ht[set2]TRUE  0.3881    1.4742   0.3479  1.1157 0.2645561
##
## $conf.int
##                    exp(coef) exp(-coef) lower .95 upper .95
## EDI5[set2]TRUE        1.8941     0.5280    1.1808     3.038
## trait$ct[set2]TRUE    3.2663     0.3062    1.6424     6.496
## trait$rt[set2]TRUE    0.7926     1.2616    0.4674     1.344
## trait$ht[set2]TRUE    1.4742     0.6783    0.7455     2.915
##
## $sctest
##      test        df    pvalue
```

```
## 2.529e+01 4.000e+00 4.395e-05
##
## $concordance
## concordance.concordant            se.std(c-d)
##                0.65459                0.03213
```

```
set2 <- grepl(3, trait$grade) & Site[[2]]
summary(coxph(trait$S_10year[set2, ] ~ EDI5[set2] + trait$ct[set2] + trait$rt[set2] +
    trait$ht[set2]))[c(7, 8, 10, 14)]
```

```
## $coefficients
##                         coef exp(coef) se(coef)      z Pr(>|z|)
## EDI5[set2]TRUE        1.0436    2.8396   0.3863  2.702 0.006896
## trait$ct[set2]TRUE    0.6840    1.9818   0.3265  2.095 0.036163
## trait$rt[set2]TRUE    0.6016    1.8250   0.4628  1.300 0.193641
## trait$ht[set2]TRUE   -0.3353    0.7151   0.3163 -1.060 0.289024
##
## $conf.int
##                    exp(coef) exp(-coef) lower .95 upper .95
## EDI5[set2]TRUE        2.8396     0.3522    1.3318     6.054
## trait$ct[set2]TRUE    1.9818     0.5046    1.0451     3.758
## trait$rt[set2]TRUE    1.8250     0.5479    0.7368     4.521
## trait$ht[set2]TRUE    0.7151     1.3984    0.3848     1.329
##
## $sctest
##      test        df    pvalue
## 13.922817  4.000000  0.007546
##
## $concordance
## concordance.concordant            se.std(c-d)
##                0.63435                0.04396
```

3.5 Further stratification for Grade 3 tumors

3.5.1 Further stratification for node, size and ER status

Additional value of EDI to node, size and ER status in Grade 3 samples is demonstrated here using Kaplan-Meier curves.

```
par(mfrow = c(3, 3))
set2 <- grepl(3, trait$grade)
Boxplot(EDI[set2] ~ trait$size[set2], ylab = "EDI", xlab = "Size")
plotSurv(trait$S_10year[set2], trait$size[set2] == 3, name = "Size", col = c("darkblue",
    "orange"))
plotSurv(trait$S_10year[set2], 5 * (trait$size[set2] == 3) + 1 * EDI5[set2],
    name = "Size & EDI5", col = c("darkblue", "darkblue", "orange", "orange"),
    lty = c(1, 4, 1, 4))
Boxplot(EDI[set2] ~ trait$node[set2], ylab = "EDI", xlab = "Node")
plotSurv(trait$S_10year[set2], trait$node[set2], name = "Node", col = c("darkblue",
    "orange"))
plotSurv(trait$S_10year[set2], 5 * trait$node[set2] * 1 + 1 * EDI5[set2], name = "Node & EDI5"
    col = c("darkblue", "darkblue", "orange", "orange"), lty = c(1, 4, 1, 4))
Boxplot(EDI[set2] ~ trait$ER.Expr[set2], ylab = "EDI", xlab = "ER")
```

```r
plotSurv(trait$S_10year[set2], trait$ER.Expr[set2], name = "ER", col = c("darkblue",
    "orange"))
plotSurv(trait$S_10year[set2], 5 * (trait$ER.Expr[set2] == "+") + 1 * EDI5[set2],
    name = "ER & EDI5", col = c("darkblue", "darkblue", "orange", "orange"),
    lty = c(1, 4, 1, 4))
```

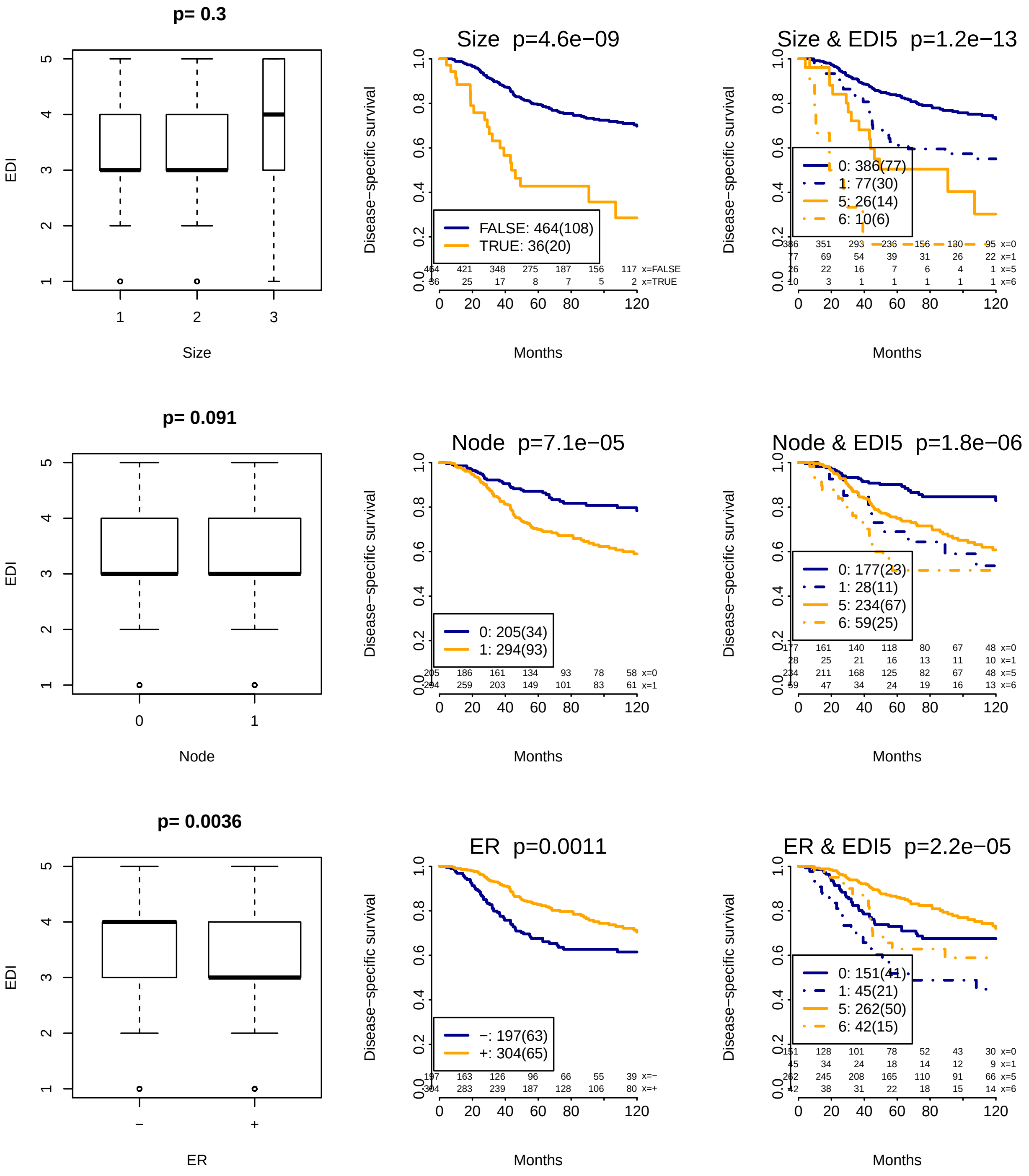

In the above plots, size 1 and 2 were merged. Here we plot all sizes:

```r
par(mfrow = c(1, 2))
plotSurv(trait$S_10year[set2], trait$size[set2], name = "Size", col = c("darkred",
    "darkgreen", "darkblue"))
plotSurv(trait$S_10year[set2], 5 * (trait$size[set2]) + 1 * EDI5[set2], name = "Size & EDI5",
    col = rep(c("darkred", "darkgreen", "darkblue"), each = 2), lty = c(1, 4,
```

```
        1, 4, 1, 4))
```

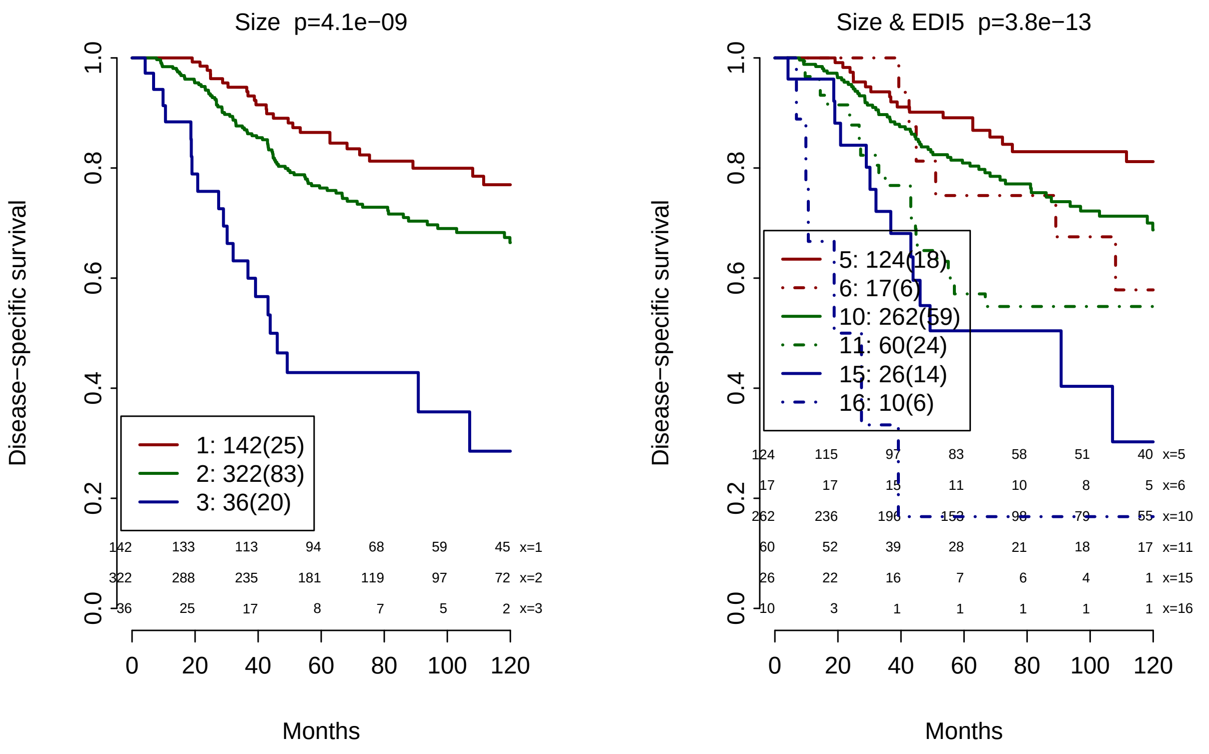

Tests for differences among tumor sizes in the EDI-high group: size 2 and 3 within EDI5; size 1 and 2 within EDI5.

```
set3 <- EDI5 & trait$size != 1
summary(coxph(trait$S_10year[set2 & set3] ~ trait$size[set2 & set3]))[c(8, 10)]

## $conf.int
##                         exp(coef) exp(-coef) lower .95 upper .95
## trait$size[set2 & set3]     4.279     0.2337     1.709     10.71
##
## $sctest
##       test        df    pvalue
## 1.136e+01 1.000e+00 7.507e-04

set3 <- EDI5 & trait$size != 3
summary(coxph(trait$S_10year[set2 & set3] ~ trait$size[set2 & set3]))[c(8, 10)]

## $conf.int
##                         exp(coef) exp(-coef) lower .95 upper .95
## trait$size[set2 & set3]     1.472     0.6792    0.6011     3.606
##
## $sctest
##   test     df pvalue
## 0.7251 1.0000 0.3945
```

Tests for differences among the subgroups defined by EDI5 revealed significant differences for all these parameters.

```
set3 <- trait$size == 3
summary(coxph(trait$S_10year[set2 & set3] ~ EDI5[set2 & set3]))[c(8, 10)]

## $conf.int
##                         exp(coef) exp(-coef) lower .95 upper .95
## EDI5[set2 & set3]TRUE     2.775     0.3603     1.034     7.451
##
## $sctest
##    test      df   pvalue
## 4.42981 1.00000 0.03532

summary(survfit(trait$S_10year[set2 & set3 & EDI5] ~ 1))

## Call: survfit.formula(formula = trait$S_10year[set2 & set3 & EDI5] ~
##     1)
##
## 1 observation deleted due to missingness
##   time n.risk n.event survival std.err lower 95% CI upper 95% CI
##   6.83      9       1    0.889   0.105       0.7056        1.000
##   9.83      8       1    0.778   0.139       0.5485        1.000
##  10.60      7       1    0.667   0.157       0.4200        1.000
##  18.80      4       1    0.500   0.186       0.2408        1.000
##  27.47      3       1    0.333   0.184       0.1128        0.985
##  39.17      2       1    0.167   0.150       0.0287        0.968

set3 <- trait$size == 2
summary(coxph(trait$S_10year[set2 & set3] ~ EDI5[set2 & set3]))[c(8, 10)]

## $conf.int
##                         exp(coef) exp(-coef) lower .95 upper .95
## EDI5[set2 & set3]TRUE     1.965     0.5088     1.222      3.16
##
## $sctest
##     test       df   pvalue
## 8.071039 1.000000 0.004498

summary(survfit(trait$S_10year[set2 & set3 & EDI5] ~ 1))

## Call: survfit.formula(formula = trait$S_10year[set2 & set3 & EDI5] ~
##     1)
##
##   time n.risk n.event survival std.err lower 95% CI upper 95% CI
##   9.43     59       1    0.983  0.0168        0.951        1.000
##   9.60     58       1    0.966  0.0236        0.921        1.000
##  14.10     57       1    0.949  0.0286        0.895        1.000
##  14.40     56       1    0.932  0.0327        0.870        0.999
##  16.73     53       1    0.915  0.0365        0.846        0.989
##  23.20     50       1    0.896  0.0401        0.821        0.979
##  24.23     49       1    0.878  0.0433        0.797        0.967
##  26.73     48       1    0.860  0.0461        0.774        0.955
##  26.77     47       1    0.841  0.0486        0.751        0.942
##  27.20     46       1    0.823  0.0509        0.729        0.929
##  31.93     45       1    0.805  0.0529        0.708        0.916
##  32.97     44       1    0.787  0.0548        0.686        0.902
```

```
## 35.00    43     1    0.768  0.0565        0.665        0.887
## 42.97    39     1    0.749  0.0584        0.642        0.872
## 43.10    38     1    0.729  0.0601        0.620        0.857
## 43.20    37     1    0.709  0.0616        0.598        0.841
## 44.60    36     1    0.689  0.0629        0.577        0.825
## 44.77    35     1    0.670  0.0642        0.555        0.808
## 45.17    34     1    0.650  0.0652        0.534        0.791
## 51.40    33     1    0.630  0.0662        0.513        0.774
## 55.00    32     1    0.611  0.0670        0.493        0.757
## 55.63    31     1    0.591  0.0676        0.472        0.740
## 56.93    30     1    0.571  0.0682        0.452        0.722
## 66.73    25     1    0.548  0.0692        0.428        0.702

set3 <- trait$size == 1
summary(coxph(trait$S_10year[set2 & set3] ~ EDI5[set2 & set3]))[c(8, 10)]

## $conf.int
##                         exp(coef) exp(-coef) lower .95 upper .95
## EDI5[set2 & set3]TRUE     2.216     0.4513     0.879     5.586
##
## $sctest
##    test      df   pvalue
## 2.99715 1.00000 0.08341

set3 <- trait$node == 1
1 - pchisq(survdiff(trait$S_10year[set2 & set3] ~ EDI5[set2 & set3])$chisq,
    1)

## [1] 0.01763

1 - pchisq(survdiff(trait$S_10year[set2 & !set3] ~ EDI5[set2 & !set3])$chisq,
    1)

## [1] 0.001232

set3 <- trait$ER == "+"
1 - pchisq(survdiff(trait$S_10year[set2 & set3] ~ EDI5[set2 & set3])$chisq,
    1)

## [1] 0.01734

1 - pchisq(survdiff(trait$S_10year[set2 & !set3] ~ EDI5[set2 & !set3])$chisq,
    1)

## [1] 0.008089
```

3.5.2 Additional stratification combining EDI5, node and size

In the left plot patients were grouped into Node-&size small (-1), Node+&size large (2) and otherwise (0). In the right panel patients were grouped into Node-&size small & EDI$\neq$5 (-1), node+&size large&EDI$\bar{5}$ (2) and otherwise (0).

```
par(mfrow = c(1, 2))
plotSurv(trait$S_10year[set2], -1 * (trait$node[set2] == 0 & trait$size[set2] ==
```

```
    1) + 2 * (trait$node[set2] == 1 & trait$size[set2] > 1), name = "Size+node")
plotSurv(trait$S_10year[set2], -1 * (trait$node[set2] == 0 & trait$size[set2] ==
    1 & (!EDI5[set2])) + 2 * (trait$node[set2] == 1 & trait$size[set2] > 1 &
    EDI5[set2]), name = "EDI+size+node")
```

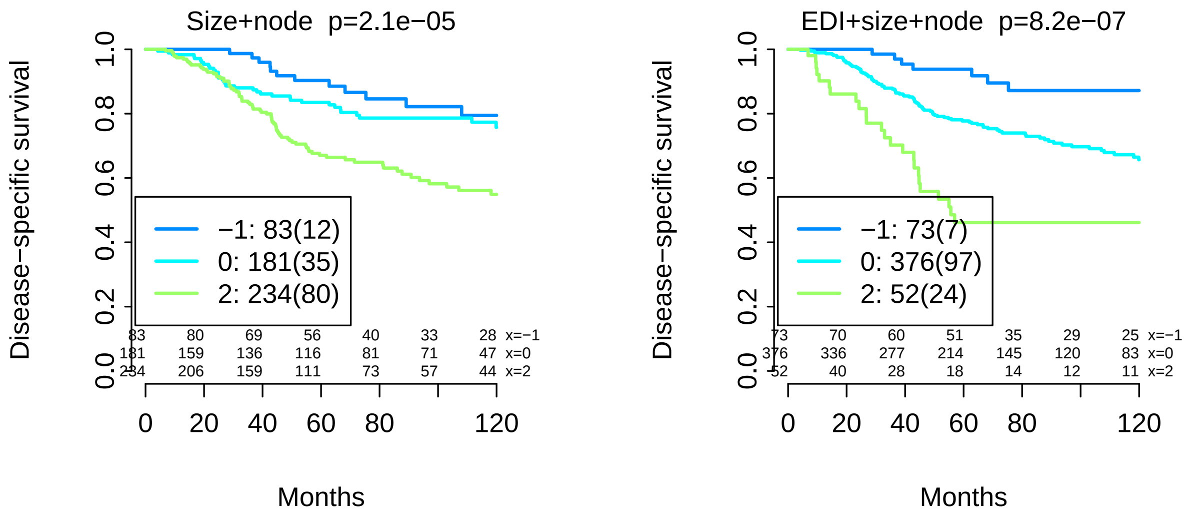


```
cl <- -1 * (trait$node[set2] == 0 & trait$size[set2] == 1) + 2 * (trait$node[set2] ==
    1 & trait$size[set2] > 1)
set3 <- cl %in% c(-1, 2)
survdiff(trait$S_10year[set2][set3] ~ cl[set3])
cl <- -1 * (trait$node[set2] == 0 & trait$size[set2] == 1 & (!EDI5[set2])) +
    2 * (trait$node[set2] == 1 & trait$size[set2] > 1 & EDI5[set2])
set3 <- cl %in% c(-1, 2)
survdiff(trait$S_10year[set2][set3] ~ cl[set3])
```

In fact, better stratification can be achieved with the addition of EDI5 as early as 5 years after diagnosis. We created an R object S for 5-year disease-specific survival data.

```
S <- trait$S_10year
S[grepl(0, S[, 2]) & S[, 1] > 60, 1] <- 60
idx <- grepl(1, S[, 2]) & S[, 1] > 60
S[idx, 1] <- 60
S[idx, 2] <- 0
cl <- -1 * (trait$node[set2] == 0 & trait$size[set2] == 1) + 2 * (trait$node[set2] ==
    1 & trait$size[set2] > 1)
set3 <- cl %in% c(-1, 2)
survdiff(S[set2][set3] ~ cl[set3])

## Call:
## survdiff(formula = S[set2][set3] ~ cl[set3])
##
## n=317, 6 observations deleted due to missingness.
##
##                  N Observed Expected (O-E)^2/E (O-E)^2/V
## cl[set3]=-1  83        7     21.9     10.17      14.5
```

```
## cl[set3]=2  234       67     52.1      4.28      14.5
##
##  Chisq= 14.5  on 1 degrees of freedom, p= 0.000142
```

```
cl <- -1 * (trait$node[set2] == 0 & trait$size[set2] == 1 & (!EDI5[set2])) +
    2 * (trait$node[set2] == 1 & trait$size[set2] > 1 & EDI5[set2])
set3 <- cl %in% c(-1, 2)
survdiff(S[set2][set3] ~ cl[set3])
```

```
## Call:
## survdiff(formula = S[set2][set3] ~ cl[set3])
##
## n=125, 2 observations deleted due to missingness.
##
##              N Observed Expected (O-E)^2/E (O-E)^2/V
## cl[set3]=-1 73        4    18.47      11.3      33.5
## cl[set3]=2  52       24     9.53      22.0      33.5
##
##  Chisq= 33.5  on 1 degrees of freedom, p= 6.97e-09
```

Size 3 and EDI5 tumors have significantly worst prognosis than the rest of high-grade tumors.

```
set3 <- trait$size[set2] > 2
survdiff(S[set2][set3] ~ EDI5[set2][set3])
```

```
## Call:
## survdiff(formula = S[set2][set3] ~ EDI5[set2][set3])
##
## n=36, 5 observations deleted due to missingness.
##
##                         N Observed Expected (O-E)^2/E (O-E)^2/V
## EDI5[set2][set3]=FALSE 26       12    15.57     0.818      6.32
## EDI5[set2][set3]=TRUE  10        6     2.43     5.237      6.32
##
##  Chisq= 6.3  on 1 degrees of freedom, p= 0.0119
```

3.6 EDI and the Genomic Grade Index

Instead of using pathological definition of grade, we tested the prognostic effect of EDI in high-grade tumors defined by the Genomic Grade Index (GGI). GGI has strong prognostic value on our cohort. EDI5 further stratifies GGI high but not GGI low group.

```
par(mfrow = c(1, 3))
plotSurv(trait$S_10year, trait$GGI > 0, name = "GGI high", type = " All samples")
set2 <- trait$GGI <= 0
plotSurv(trait$S_10year[set2], EDI5[set2], type = " in GGI low grade", name = "EDI5",
    col = c("darkblue", "orange"))
set2 <- trait$GGI > 0
plotSurv(trait$S_10year[set2], EDI5[set2], type = " in GGI high grade", name = "EDI5",
    col = c("darkblue", "orange"))
```

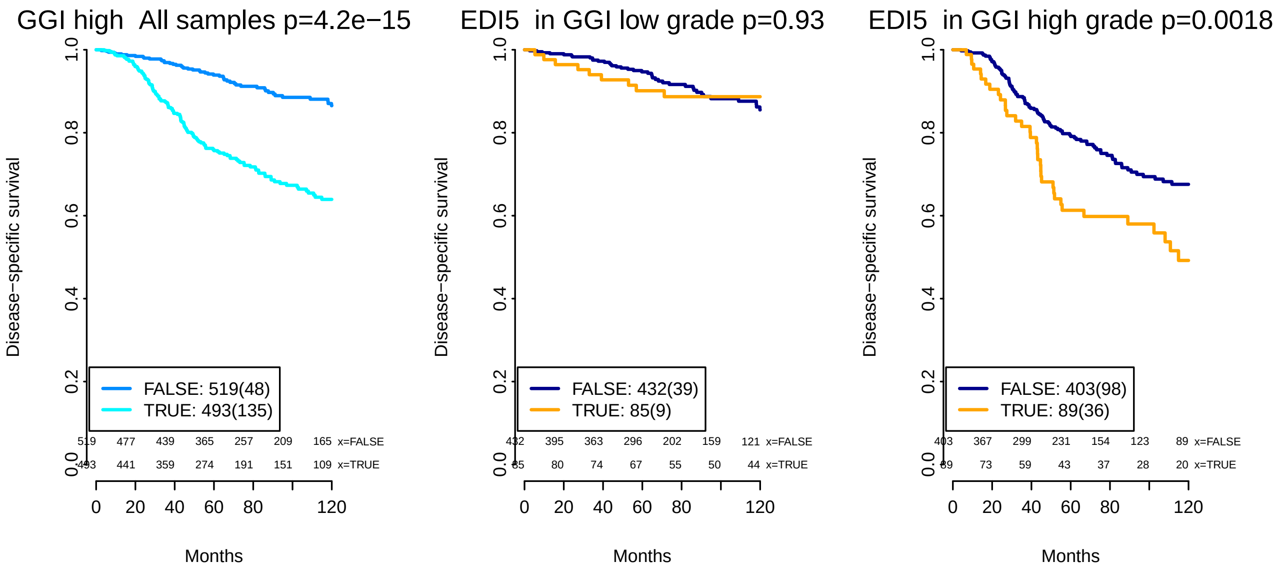

3.7 Compare EDI and Shannon entropy of the whole tumor

EDI represents the spatial variability in Shannon entropy for a tumor. Shannon entropy can be applied to the whole tumor to measure diversity in tumor composition. To test their difference, we computed the entropy using cell proportion data. First we observed a strong correlation between entropy and EDI5.

```
regStatDir <- "./data/image/"
require(vegan)
Entropy <- sapply(trait$file, function(x) {
    res <- try(read.table(paste(regStatDir, x, ".txt", sep = ""), as.is = T,
        sep = "\t", row.names = NULL))
    if (class(res) != "try-error") {
        res <- rowSums(res[, 3:5])
        dvs <- diversity(res, index = "shannon", MARGIN = 1)
    } else {
        NA
    }
})

par(mfrow = c(2, 2))
hist(Entropy, br = 100, xlab = "Entropy")
Boxplotkw(Entropy ~ trait$size, ylab = "Entropy", xlab = "size")
Boxplotkw(Entropy ~ EDI5, ylab = "Entropy", xlab = "EDI5")
summary(coxph(trait$S_10year ~ Entropy))[c(8, 10)]

## $conf.int
##         exp(coef) exp(-coef) lower .95 upper .95
## Entropy     1.347     0.7422     1.102     1.648
##
## $sctest
##     test       df    pvalue
## 8.363694 1.000000 0.003828

summary(coxph(trait$S_10year ~ EDI5))[c(8, 10)]
```

```
## $conf.int
##          exp(coef) exp(-coef) lower .95 upper .95
## EDI5TRUE     1.515     0.6599     1.082     2.123
##
## $sctest
##    test      df  pvalue
## 5.92147 1.00000 0.01496
```

```r
summary(coxph(trait$S_10year ~ Entropy + EDI5))[c(7, 8, 10)]
```

```
## $coefficients
##            coef exp(coef) se(coef)     z Pr(>|z|)
## Entropy  0.2384     1.269   0.1127 2.115  0.03443
## EDI5TRUE 0.2304     1.259   0.1914 1.204  0.22865
##
## $conf.int
##          exp(coef) exp(-coef) lower .95 upper .95
## Entropy      1.269     0.7879    1.0176     1.583
## EDI5TRUE     1.259     0.7942    0.8653     1.832
##
## $sctest
##      test        df    pvalue
## 10.191087  2.000000  0.006124
```

```r
summary(coxph(trait$S_10year ~ Entropy + trait$grade, subset = Site[[1]]))[c(7,
    8, 10)]
```

```
## $coefficients
##               coef exp(coef) se(coef)     z  Pr(>|z|)
## Entropy     0.4233     1.527   0.1528 2.769 5.616e-03
## trait$grade 0.7607     2.140   0.1638 4.645 3.404e-06
##
## $conf.int
##             exp(coef) exp(-coef) lower .95 upper .95
## Entropy         1.527     0.6549     1.132     2.060
## trait$grade     2.140     0.4674     1.552     2.949
##
## $sctest
##      test        df    pvalue
## 3.160e+01 2.000e+00 1.373e-07
```

```r
summary(coxph(trait$S_10year ~ Entropy + trait$grade, subset = Site[[2]]))[c(7,
    8, 10)]
```

```
## $coefficients
##               coef exp(coef) se(coef)     z  Pr(>|z|)
## Entropy     0.1593     1.173   0.1578 1.010 3.126e-01
## trait$grade 1.0138     2.756   0.2602 3.895 9.801e-05
##
## $conf.int
##             exp(coef) exp(-coef) lower .95 upper .95
## Entropy         1.173     0.8528    0.8608     1.598
## trait$grade     2.756     0.3628    1.6548     4.590
##
```

```
## $sctest
##      test        df    pvalue
## 1.757e+01 2.000e+00 1.529e-04
```

```
summary(coxph(trait$S_10year ~ Entropy, subset = trait$grade == 3))[c(8, 10)]
```

```
## $conf.int
##         exp(coef) exp(-coef) lower .95 upper .95
## Entropy     1.421     0.7039     1.119     1.804
##
## $sctest
##    test      df  pvalue
## 8.27047 1.00000 0.00403
```

```
summary(coxph(trait$S_10year ~ Entropy + EDI5, subset = trait$grade == 3))[c(7,
    8, 10)]
```

```
## $coefficients
##            coef exp(coef) se(coef)     z Pr(>|z|)
## Entropy  0.1941     1.214   0.1331 1.459 0.144612
## EDI5TRUE 0.5900     1.804   0.2260 2.611 0.009035
##
## $conf.int
##          exp(coef) exp(-coef) lower .95 upper .95
## Entropy      1.214     0.8235    0.9355     1.576
## EDI5TRUE     1.804     0.5543    1.1584     2.809
##
## $sctest
##      test        df    pvalue
## 1.742e+01 2.000e+00 1.647e-04
```

```
Boxplotkw(Entropy ~ trait$grade, ylab = "Entropy", xlab = "grade")
```

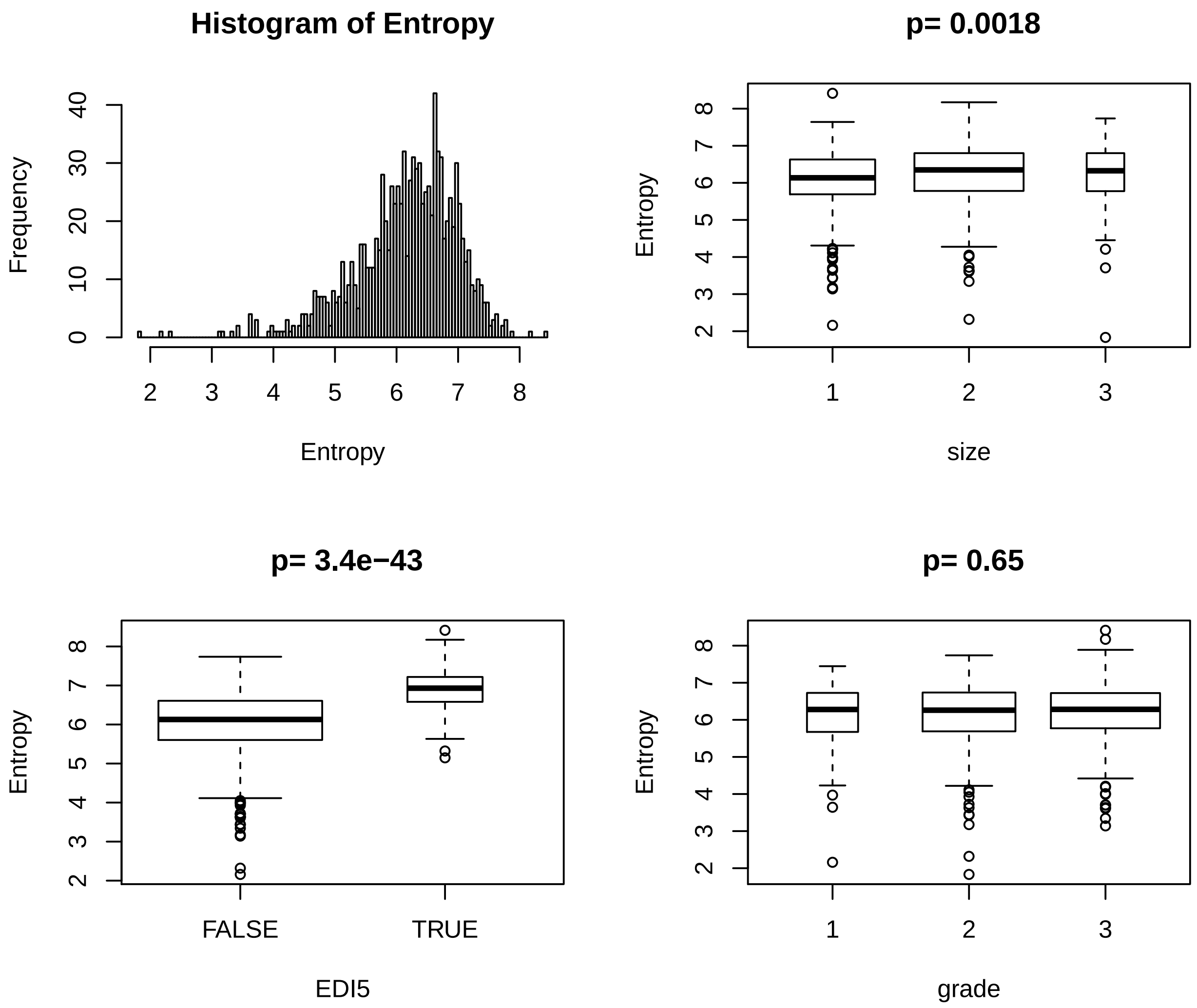

In all samples, n is a stronger prognostic factor than EDI5 as seen in univariate Cox analysis. However when restricted to high grade cancers EDI5 is the only prognostic factor in multivariate analysis together with n. In low grade cancers (grade=1 or 2), neither is prognostic. A possible explanation is that n is not independent of grade. We therefore tested a multivariate model with only n and grade, and found that n is not prognostic in the validation cohort given tumor grade.

4 Generating EDI scores

Here we describe how to generate EDI scores using function getEDI in the file EDIfunctions.R. getEDI function computes EDI using cell composition data in the “image" folder, which can be generated by function getRegionalComposition. getRegionalComposition first reads CRImage processed files for all the sub images in a tumor section slide, divides an sub image into the size of region specified by the parameter n, and writes cell count table as a csv file. The getEDI function then uses this table to calculate the Shannon Diversity Index score for each region using the R package vegan. The regional diversity scores were then used for clustering using the R package Mclust. This part of the code is not executed in the sweave file.

```
regStatDir <- "./data/image/"
EDI <- sapply(trait$file, function(x) getEDI(x, regStatDir))
```

Several parameters of the getEDI function need to be determined. By default, q is set to 0.8, which means that prior to clustering lower 20% and upper 20% quantiles of data were removed. This step removes outliers and artefacts in the frozen tumor section images. Removing the lower 20% quantile

data helps remove out-of-focus, folded tissue, stained glass, and occasionally cancerous/homogeneous regions. Removing the upper 20% quantile data helps remove fat tissue, highly fragmented tissue, stroma tumor regions. Since we are interested in the interface between cancer and microenvironment, removal of these regions will lead to more accurate detection of ecosystem heterogeneity. An example with regions annotated with the number of cluster k can be seen here. We will test the robustness of this step in subsequent section.

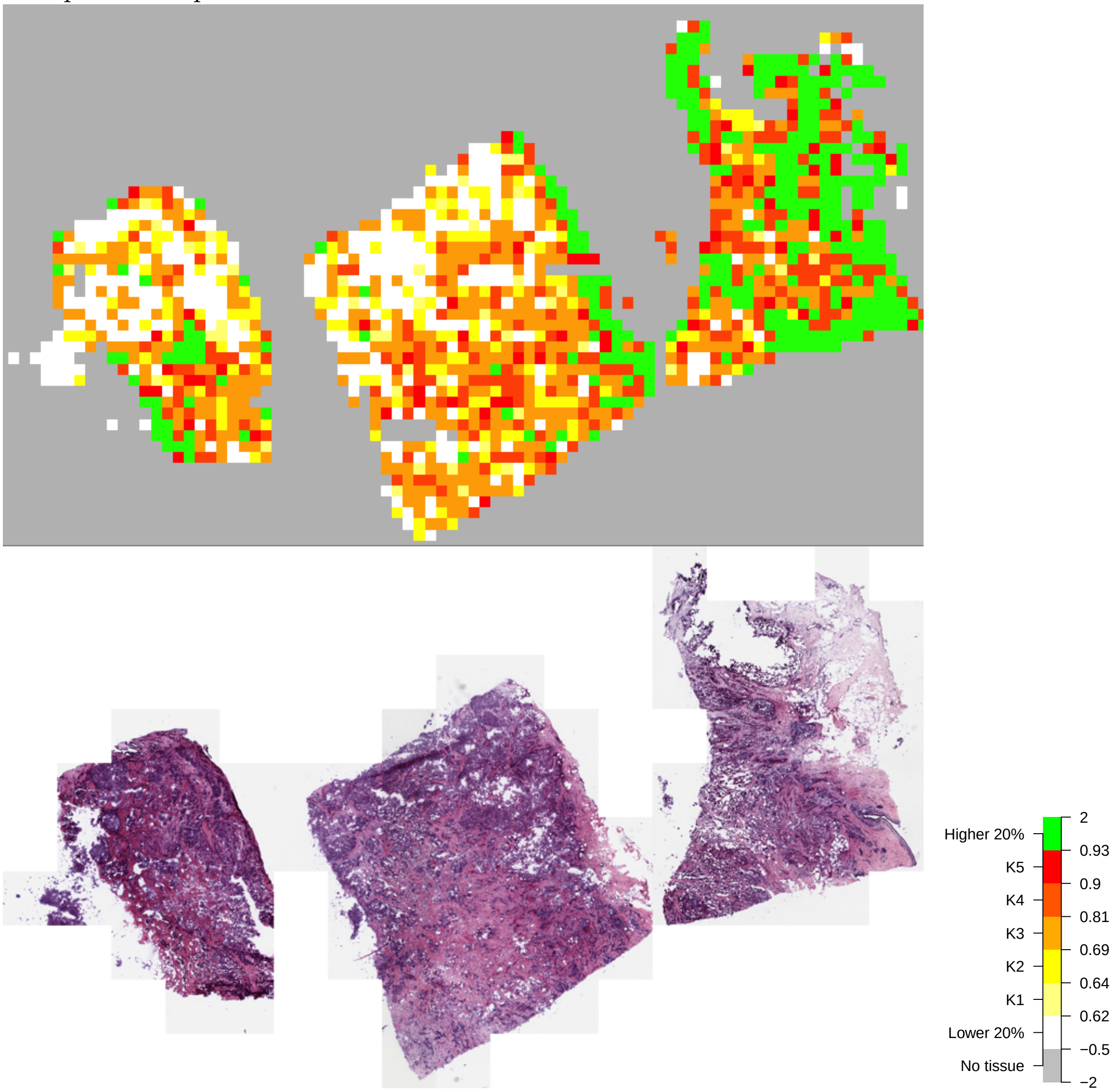

5 Stability of EDI

5.1 Resampling tumor regions

To test the stability of EDI and its dependency on number of regions, we randomly sample 200 high grade tumor images to speed up computing, and run getEDI function with resampling a fraction of the tumor regions for each tumor (80-100%). Since it takes a while to compute, we saved the result as a rdata file.

```
regStatDir <- "./data/image/"
set.seed(40)
samples <- sample(which(trait$grade == 3), 200)
testrange <- seq(1, 0.8, len = 21)
EDI0 <- sapply(testrange, function(q) {
```

```
    sapply(trait$file[samples], function(x) getEDI(x, regStatDir, sampling = q))
})
save(EDI0, file = "./data/EDI0sampling.rdata")
```

EDI0 is a matrix. The first column of EDI0 contains the results using all tumor regions, and the second column contains EDI results by sampling 99% of the tumor regions and so on. Thus we can compare them using the percentage of agreement. We can observe that for the identification of the EDI5 group, which is the focus of this paper, the analysis remains stable (90% agreement with 100% samples) even when only 80% of tumor regions were used in the calculation of EDI. Agreement was computed as $\frac{\sum(bothareEDI5)+\sum(NeitherareEDI5)}{\sum(totalnumberofsamples)}$.

```
set.seed(40)
samples <- sample(which(trait$grade == 3), 200)
testrange <- seq(1, 0.8, len = 21)
load(file = "./data/EDI0sampling.rdata")
fp2 <- sapply(1:length(testrange), function(x) {
    m <- table(EDI0[, 1] == 5, EDI0[, x] == 5)
    sum(diag(m))/sum(m)
})
plot(testrange, fp2, ylim = c(0.5, 1), xlab = "Proportion of sampled tumor regions",
    ylab = "Agreement with 100% tumor regions", main = "EDI5", pch = 19)
```

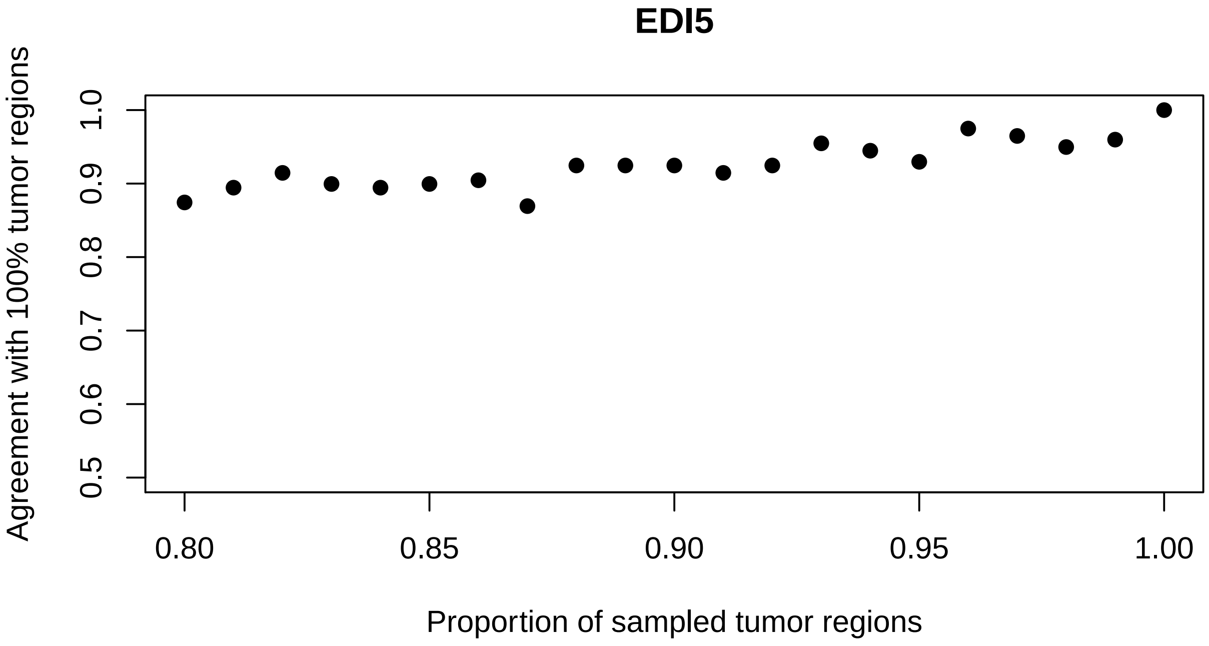

Next we ask whether the EDI5 stratification remains significant with reduced number of tumor regions by sampling.

```
par(mfrow = c(7, 3), mar = c(3, 4, 3, 0))
p <- NULL
for (i in 1:21) {
    tmp <- EDI0[, i] == 5
    tmp <- replace.vector(tmp, c(TRUE, FALSE), c("High", "Low"))
    p <- c(p, plotSurv(trait$S_10year[samples], tmp, name = "", type = paste(testrange[i] *
        100, "% data")))
}
```

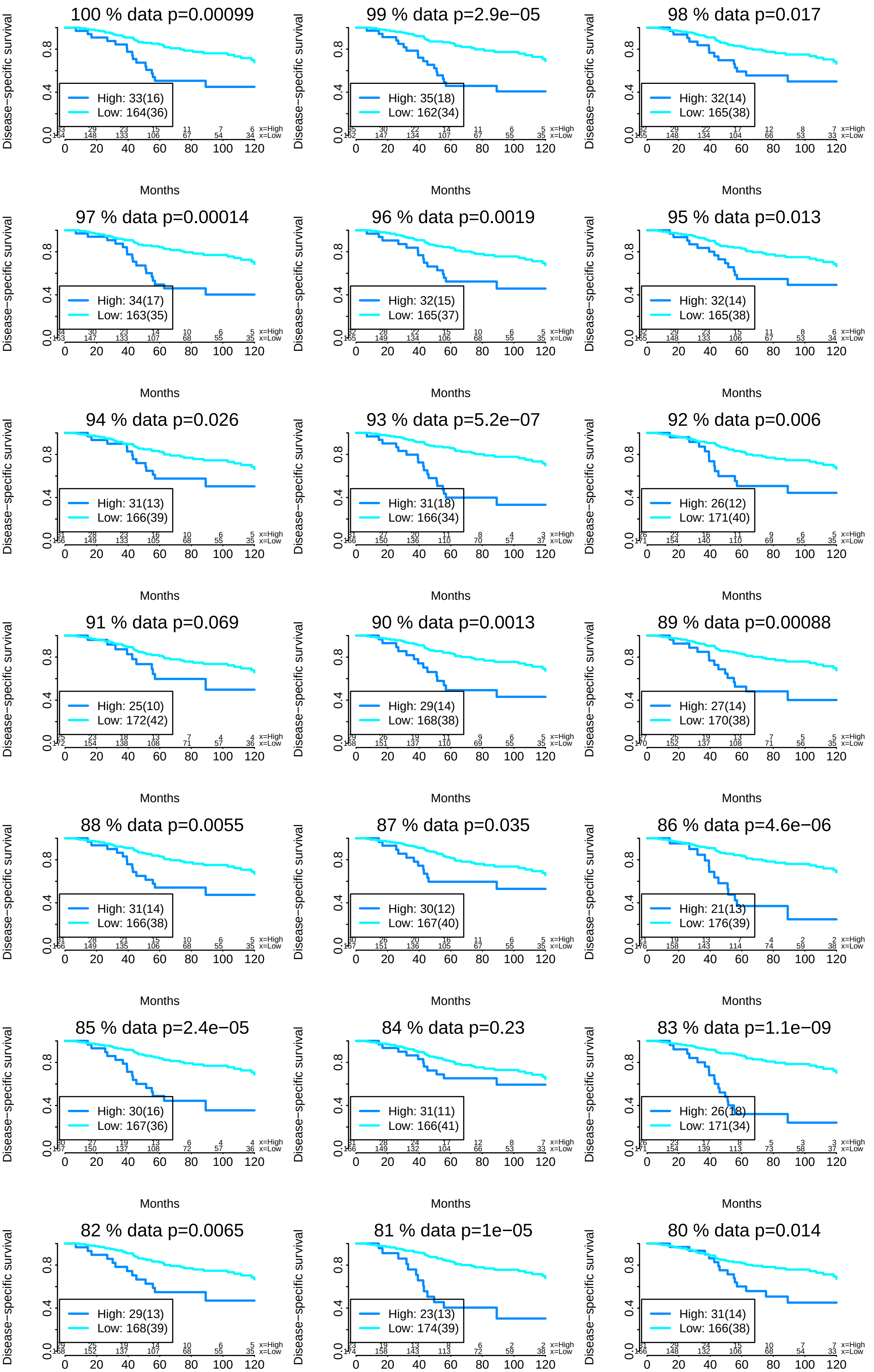
100 % data p=0.00099
High: 33(16)
Low: 164(36)
99 % data p=2.9e−05
High: 35(18)
Low: 162(34)
98 % data p=0.017
High: 32(14)
Low: 165(38)
97 % data p=0.00014
High: 34(17)
Low: 163(35)
96 % data p=0.0019
High: 32(15)
Low: 165(37)
95 % data p=0.013
High: 32(14)
Low: 165(38)
94 % data p=0.026
High: 31(13)
Low: 166(39)
93 % data p=5.2e−07
High: 31(18)
Low: 166(34)
92 % data p=0.006
High: 26(12)
Low: 171(40)
91 % data p=0.069
High: 25(10)
Low: 172(42)
90 % data p=0.0013
High: 29(14)
Low: 168(38)
89 % data p=0.00088
High: 27(14)
Low: 170(38)
88 % data p=0.0055
High: 31(14)
Low: 166(38)
87 % data p=0.035
High: 30(12)
Low: 167(40)
86 % data p=4.6e−06
High: 21(13)
Low: 176(39)
85 % data p=2.4e−05
High: 30(16)
Low: 167(36)
84 % data p=0.23
High: 31(11)
Low: 166(41)
83 % data p=1.1e−09
High: 26(18)
Low: 171(34)
82 % data p=0.0065
High: 29(13)
Low: 168(39)
81 % data p=1e−05
High: 23(13)
Low: 174(39)
80 % data p=0.014
High: 31(14)
Low: 166(38)
Disease−specific survival
Months
x=High
x=Low

Thus, for 90.4762% of the times, EDI5 stratification remains stable. This demonstrates the stability of EDI5 group in situations where less tumor regions were used for computing EDI scores.

We then repeat the same sampling procedure 25 times, and compute False Positive rate and frequency of resampling EDI being significant in survival analysis.

```
testrange <- seq(1, 0.8, len = 21)
EDI0list <- NULL
for (i in 1:25) {
    EDI0 <- sapply(testrange, function(q) {
        sapply(trait$file[samples], function(x) getEDI(x, regStatDir, sampling = q))
    })
    EDI0list <- c(EDI0list, list(EDI0))
}
save(EDI0list, file = "./data/EDI0list.rdata")
```

```
FP <- NULL
PVAL <- NULL
for (i in 1:25) {
    EDI0 <- EDI0list[[i]]
    fp2 <- sapply(1:length(testrange), function(x) {
        m <- table(EDI0[, 1] == 5, EDI0[, x] == 5)
        sum(diag(m))/sum(m)
    })
    pval <- sapply(1:length(testrange), function(x) {
        x <- EDI0[, x] == 5
        fit <- survfit(trait$S_10year[samples] ~ x, data = dat)
        test <- survdiff(trait$S_10year[samples] ~ x, data = dat, rho = 0)
        1 - pchisq(test$chisq, length(test$n) - 1)
    })
    FP <- cbind(FP, fp2)
    PVAL <- cbind(PVAL, pval)
}
save(FP, PVAL, file = "./data/FP.rdata")
```

Now visualise sampling results:

```
load("./data/FP.rdata")
library(ggplot2)
xlabel <- as.expression(expression(paste("Test range")))
ylabel <- "Proportion of  tumor region sampled"

A <- t(apply(FP, 1, quantile))
A <- data.frame(A, testrange = testrange)
p <- ggplot(data = A, aes(x = testrange, y = X50., ymin = X25., ymax = X75.,
    fill = "grey")) + geom_line() + geom_ribbon(alpha = 0.5) + xlab(xlabel) +
    ylab(ylabel) + ylim(0.5, 1) + geom_line(aes(y = X0.), linetype = "dotted") +
    geom_line(aes(y = X100.), linetype = "dotted")
plot(p)
```

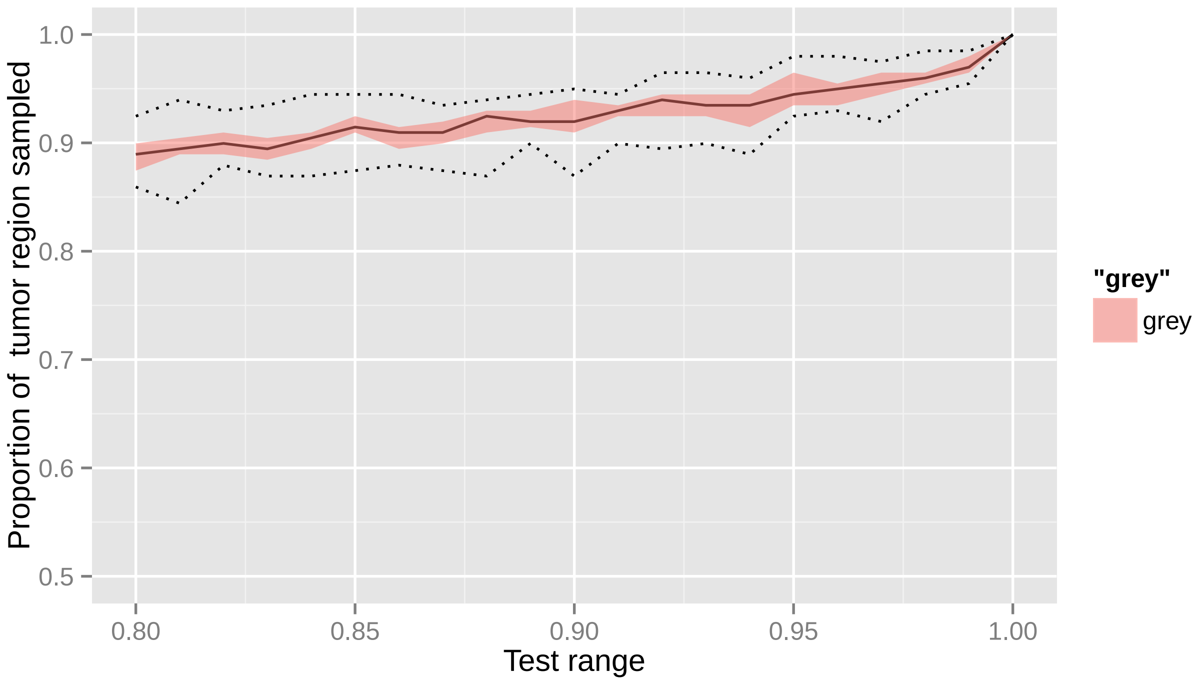


```
library(ggplot2)
xlabel <- as.expression(expression(paste("Percentage sampled")))
ylabel <- "Frequency of  prognostic significance"
PVAL0 <- 1 * (PVAL < 0.05)
plot(y = rowSums(PVAL0)/25, x = testrange, ylim = c(0, 1), ylab = "Frequency of significance",
    xlab = "Percentage of regions used", pch = 19)
abline(h = 0.9, lty = 2)
abline(h = 0.8, lty = 2)
```

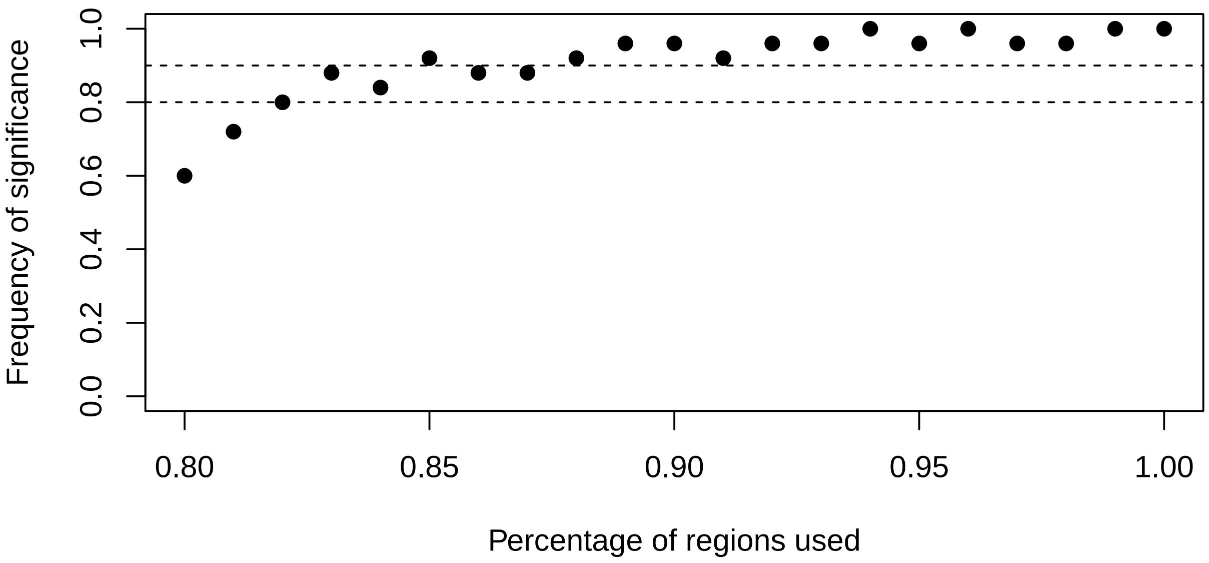

Thus 80% of the times EDI computed by our resampling procedure remain statistically significant in survival analysis when at least 82

5.2 Correlation with input data

We next asked whether EDI correlates with the number of tumor regions used as input data. Here we computed the number of tumor regions for 1,026 tumors n. Not surprisingly, n is highly correlated with the clinical parameter tumor size. EDI is also significantly correlated with tumor size (p=0.015) and n.

```
regStatDir <- "./data/image/"
minCell <- 400/25
n <- sapply(trait$file, function(x) {
    res <- try(read.table(paste(regStatDir, x, ".txt", sep = ""), as.is = T,
        sep = "\t", row.names = NULL))
    if (class(res) != "try-error") {
        res <- res[rowSums(res[, 3:5]) >= minCell, ]
        nrow(res)
    } else {
        NA
    }
})
par(mfrow = c(2, 2))
Boxplot(n ~ trait$size, ylab = "n", xlab = "size")
Boxplot(EDI ~ trait$size, ylab = "EDI", xlab = "size")
Boxplot(n ~ EDI, ylab = "n", xlab = "EDI")

load("./data/NumberOfPixels.rdata")
Boxplot(px ~ EDI, ylab = "Tissue")
```

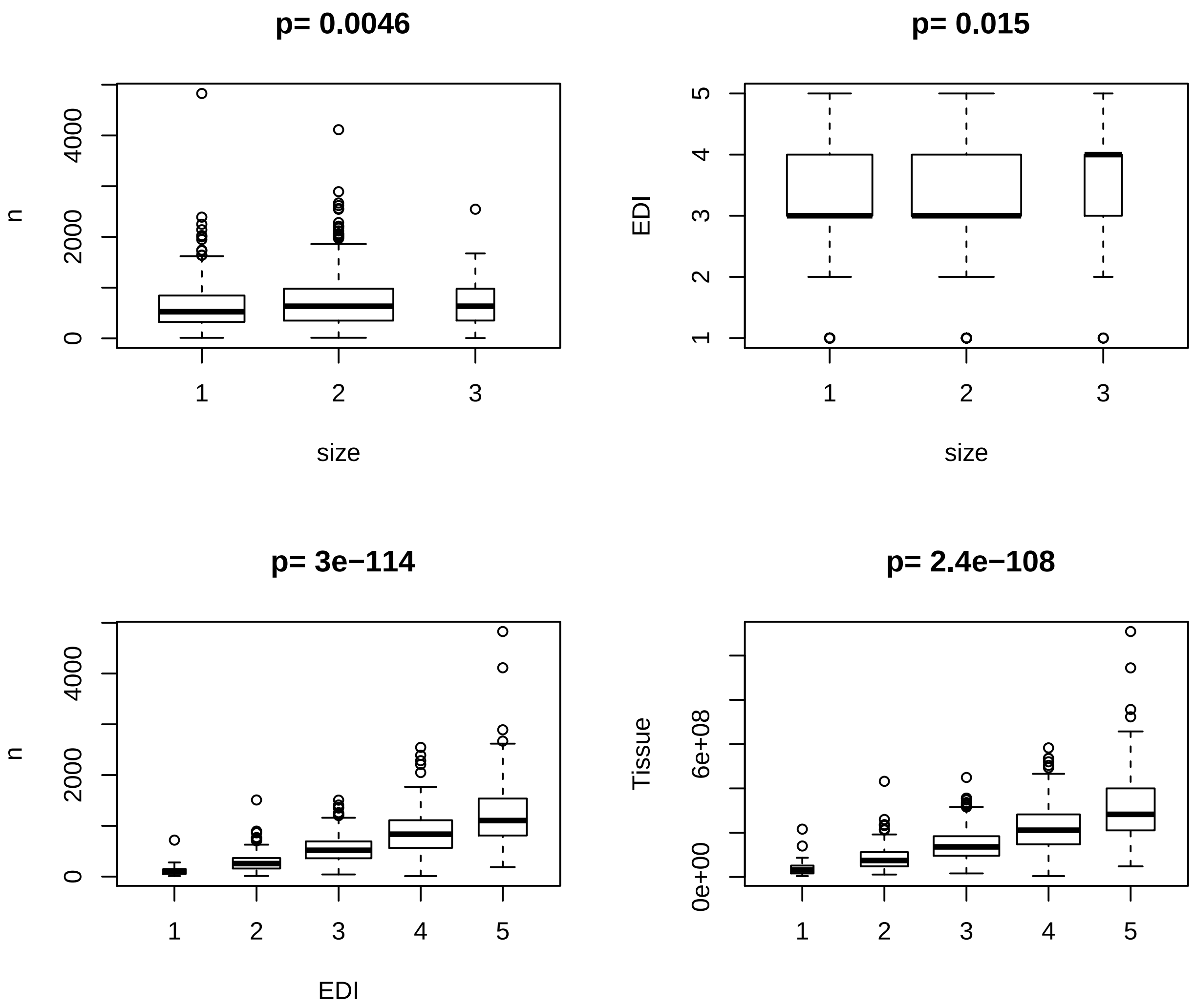

We next examine the prognostic value of n in comparison with EDI in grade 3 tumors. First n was dichotomised to two groups of same sizes as EDI5. Univariate Cox analysis shows significant correlation with DSS (p=0.005) and multivariate Cox analysis with EDI5 shows that n has no independent prognostic value over EDI5 (p-value for n 0.26, EDI5 0.004). Next, n was dichotomised by its 25 and 75 percentiles. Again, univariate Cox analysis shows its significant correlation with DSS (p=0.004) and multivariate Cox analysis with EDI5 shows that n has no independent prognostic value over EDI5 (p-value for n 0.13, EDI5 0.006). Finally, n was dichotomised by its 50 percentiles. Univariate Cox analysis shows no significant correlation with DSS (p=0.21). We tested a range of cutoffs to dichotomise n but none resulted in superior value to EDI5. Using n as a continuous variable for univariate and multivariate analysis similar conclusion can be drawn. Thus, EDI is correlated with but independent of tumor size.

```
set2 <- grepl(3, trait$grade)
n0 <- n[set2] > sort(n[set2], decreasing = T)[sum(EDI5[set2], na.rm = T)]
table(n0)

## n0
## FALSE  TRUE
##   419    87

summary(coxph(trait$S_10year[set2, ] ~ n0))$coefficients

##            coef exp(coef) se(coef)     z Pr(>|z|)
## n0TRUE 0.5549     1.742   0.2024 2.741 0.006119
```

```r
summary(coxph(trait$S_10year[set2, ] ~ n0 + EDI5[set2]))$coefficients

##                    coef exp(coef) se(coef)     z Pr(>|z|)
## n0TRUE          0.2598     1.297   0.2323 1.118 0.263364
## EDI5[set2]TRUE 0.6366     1.890   0.2260 2.816 0.004857


n0 <- group3(n[set2])
table(n0)

## n0
##   1   2   3
## 128 251 127

summary(coxph(trait$S_10year[set2, ] ~ n0))$coefficients

##     coef exp(coef) se(coef)     z Pr(>|z|)
## n0 0.359     1.432   0.1262 2.844 0.004459

summary(coxph(trait$S_10year[set2, ] ~ n0 + EDI5[set2]))$coefficients

##                    coef exp(coef) se(coef)     z Pr(>|z|)
## n0              0.2073     1.230   0.1394 1.487 0.136975
## EDI5[set2]TRUE 0.6021     1.826   0.2211 2.724 0.006458


n0 <- group2(n[set2])
table(n0)

## n0
##   1   2
## 253 253

summary(coxph(trait$S_10year[set2, ] ~ n0))$coefficients

##      coef exp(coef) se(coef)     z Pr(>|z|)
## n0 0.2199     1.246   0.1793 1.227     0.22

summary(coxph(trait$S_10year[set2, ] ~ n0 + EDI5[set2]))$coefficients

##                     coef exp(coef) se(coef)      z  Pr(>|z|)
## n0              -0.01698    0.9832   0.1974 -0.086 0.9314640
## EDI5[set2]TRUE  0.76351    2.1458   0.2169  3.520 0.0004308


summary(coxph(trait$S_10year[set2, ] ~ n[set2]))$coefficients

##               coef exp(coef)  se(coef)     z  Pr(>|z|)
## n[set2] 0.0003821         1 0.0001159 3.296 0.0009795

summary(coxph(trait$S_10year[set2, ] ~ n[set2] + EDI5[set2]))$coefficients

##                     coef exp(coef) se(coef)     z Pr(>|z|)
## n[set2]        0.0001981     1.000 0.000144 1.376  0.16885
## EDI5[set2]TRUE 0.5972733     1.817 0.232988 2.564  0.01036
```

5.3 Determining parameters

As mentioned above, getEDI function requires a parameter q. Here we test a range of q around the preselected value of 0.8. 200 of the Grade 3 samples were randomly sampled, and EDI for q=0.75, 0.77, 0.79, 0.81, 0.83, 0.85 were computed. Again due to the time for computation, results were first saved as rdata file.

```
set.seed(40)
samples <- sample(which(trait$grade == 3), 200)
testrange <- seq(0.75, 0.85, len = 6)
EDI0 <- sapply(testrange, function(q) {
    sapply(trait$file[samples], function(x) getEDI(x, regStatDir, q = q))
})
save(EDI0, file = "./data/EDI0q.rdata")
```

```
load("./data/EDI0q.rdata")
set.seed(40)
samples <- sample(which(trait$grade == 3), 200)
testrange <- seq(0.75, 0.85, len = 6)
fp2 <- sapply(1:length(testrange), function(x) {
    m <- table(EDI[samples] == 5, EDI0[, x] == 5)
    sum(diag(m))/sum(m)
})
par(mfrow = c(2, 2))
plot(testrange, fp2, ylim = c(0, 1), xlab = "Quantile parameter in getEDI function",
    ylab = "Agreement with preset parameter", main = "EDI5", pch = 19)
plotSurv(trait$S_10year[samples], EDI0[, 5] == 5, type = paste("q=", testrange[5]))
plotSurv(trait$S_10year[samples], EDI0[, 2] == 5, type = paste("q=", testrange[2]))
plotSurv(trait$S_10year[samples], EDI0[, 1] == 5, type = paste("q=", testrange[1]))
```

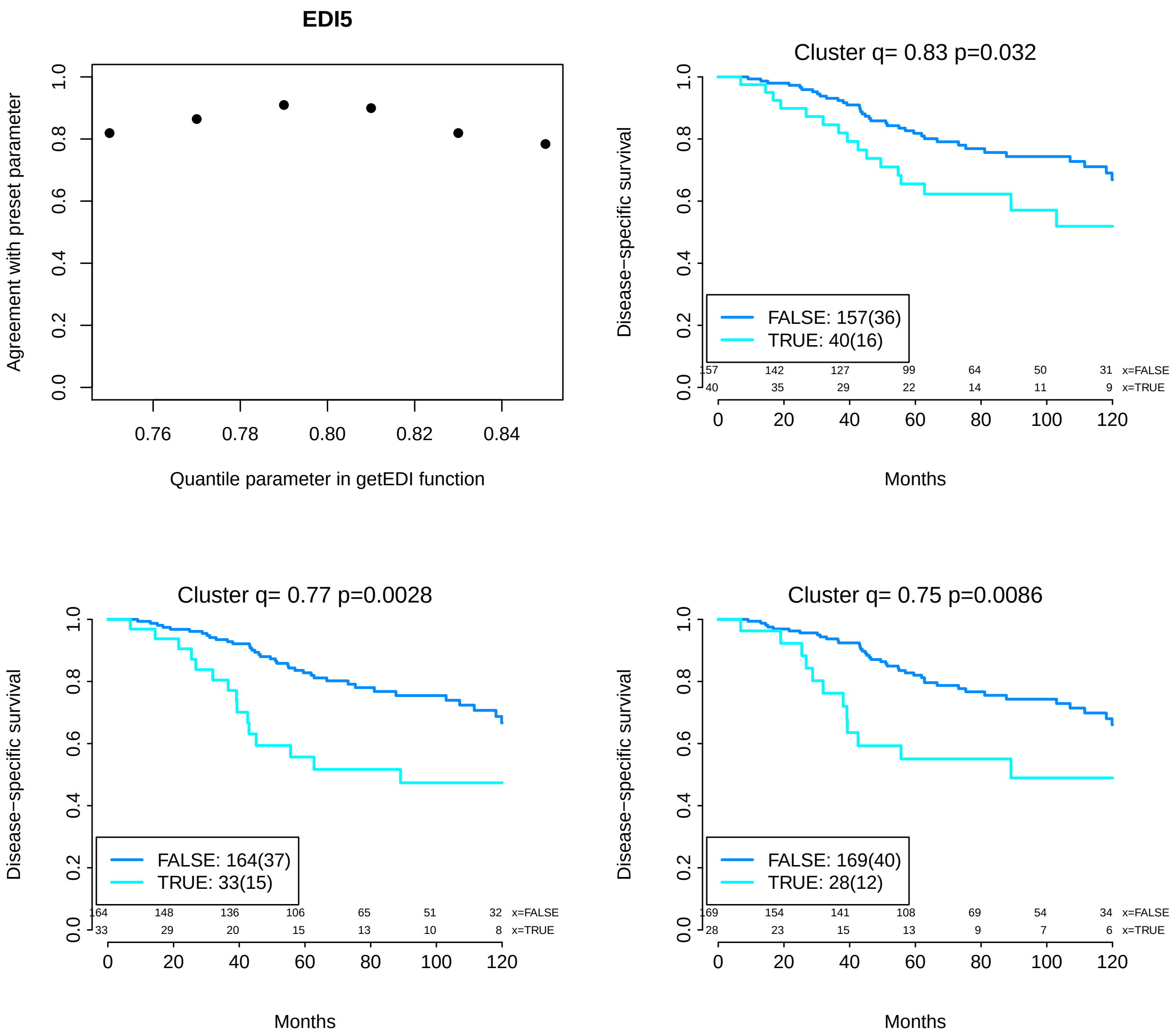

The top left panel shows how different the results are with q=0.8 (78%-90% agreement). Then based on the KM curves for these results, for most of these runs the stratification of EDI5 group remains significant.

6 Association of EDI with genomics

To access genome-wide copy number data, one needs to apply to the METABRIC consortium. Here we made the copy number profile of 4p14 and 5q13 available for reproducing our results presented in the paper. The distribution of genomic aberrations on 4p14 and 5q13 for all breast tumors is plotted.

```
load("./data/4p5q_cn.rdata")
cn4ploss <- cn4p == -1
cn5qloss <- cn5q == -1
par(mfrow = c(1, 2))
hist(cn4p)
hist(cn5q)
```

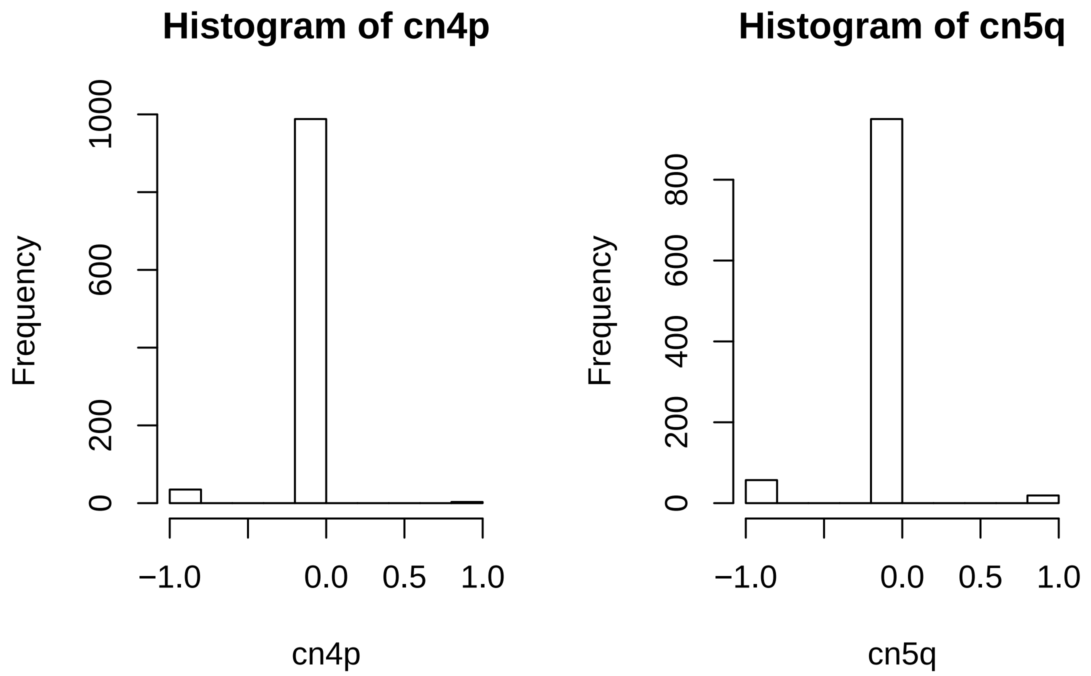

6.1 Synergistic association between EDI5 and 4p14, 5q13

The association between DSS and the synergistic effect of genomics and ecosystem heterogeneity is illustrated here:

```
par(mfrow = c(1, 2))
set2 <- grepl(3, trait$grade)
plotSurv(trait$S_10year[set2], cn4ploss[set2], name = "4p14")
plotSurv(trait$S_10year[set2], cn5qloss[set2], name = "5q13")
```

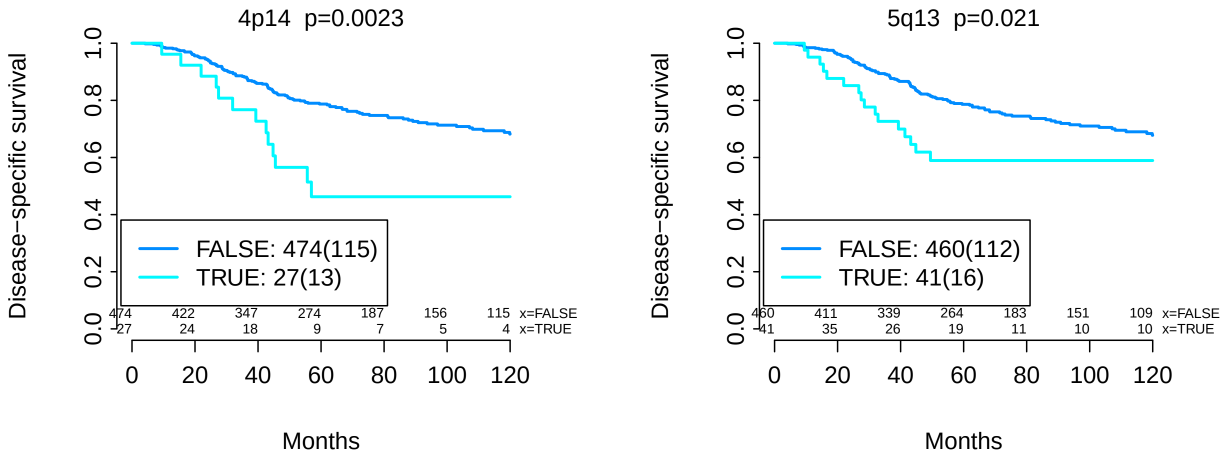

Further dividing samples into two cohorts:

```
set2 <- grepl(3, trait$grade) & Site[[1]]
summary(coxph(trait$S_10year[set2] ~ cn4ploss[set2]))[c(7, 8, 10)]

## $coefficients
```

```
##                       coef exp(coef) se(coef)     z Pr(>|z|)
## cn4ploss[set2]TRUE 1.056     2.876   0.3253 3.247 0.001167
##
## $conf.int
##                      exp(coef) exp(-coef) lower .95 upper .95
## cn4ploss[set2]TRUE     2.876     0.3478      1.52      5.44
##
## $sctest
##      test        df    pvalue
## 1.156e+01 1.000e+00 6.756e-04
```

```
set2 <- grepl(3, trait$grade) & Site[[2]]
summary(coxph(trait$S_10year[set2] ~ cn4ploss[set2]))[c(7, 8, 10)]
```

```
## $coefficients
##                        coef exp(coef) se(coef)      z Pr(>|z|)
## cn4ploss[set2]TRUE 0.1575     1.171   0.7244 0.2174   0.8279
##
## $conf.int
##                      exp(coef) exp(-coef) lower .95 upper .95
## cn4ploss[set2]TRUE     1.171     0.8543     0.283     4.842
##
## $sctest
##    test      df  pvalue
## 0.04738 1.00000 0.82769
```

On the other hand, we can also further stratify EDI by genomics.

```
par(mfrow = c(1, 3))
set2 <- grepl(3, trait$grade)
plotSurv(trait$S_10year[set2], EDI5[set2], name = "EDI5", col = c("black", "red"))
comb <- 1 * (cn4ploss[set2]) + 3 * (EDI5[set2])
comb <- replace.vector(comb, c(0, 1, 3, 4), c("Low EDI& no 4p loss", "Low EDI&4p loss",
    "EDI5&no 4p loss", "EDI5&4p loss"))
plotSurv(trait$S_10year[set2], comb, name = "", col = c("darkred", "orange",
    "darkblue", "lightblue"))
comb[comb %in% c("Low EDI& no 4p loss", "Low EDI&4p loss")] <- "Low EDI"
plotSurv(trait$S_10year[set2], comb, name = "", col = c("darkred", "orange",
    "black"))
```

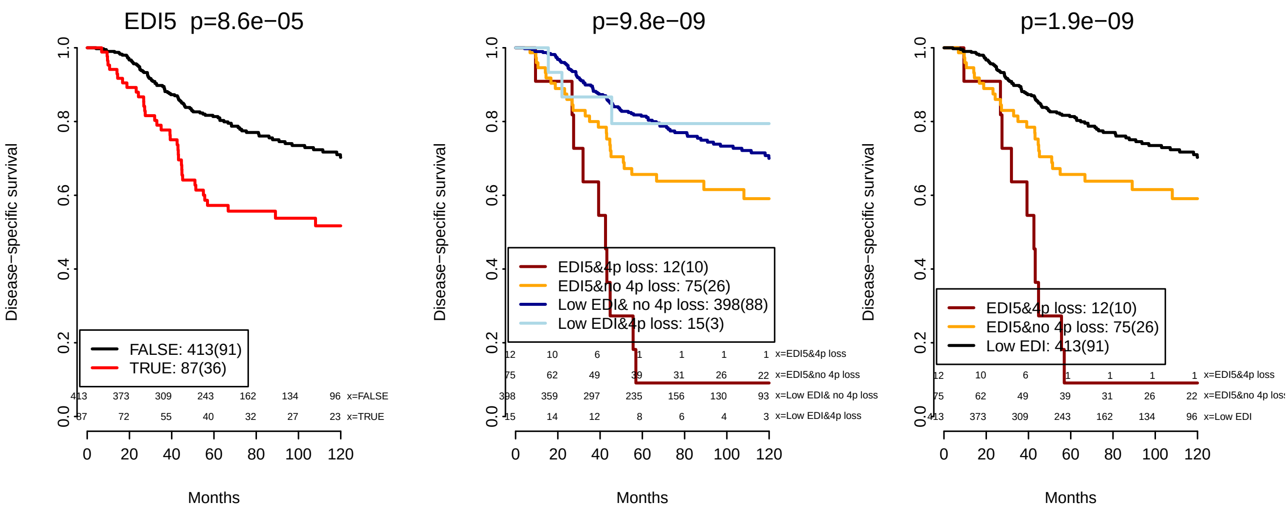


```r
summary(survfit(trait$S_10year[set2 & cn4ploss & EDI5] ~ 1))
```

This indicates that genomic changes on 4p further sub stratify EDI high group into a very poor and a relatively better outcome group. Similarly with 5q:

```r
par(mfrow = c(1, 3))
plotSurv(trait$S_10year[set2], EDI5[set2], name = "EDI5", col = c("black", "red"))
comb <- 1 * (cn5qloss[set2]) + 3 * (EDI5[set2])
comb <- replace.vector(comb, c(0, 1, 3, 4), c("Low EDI& no 5q loss", "Low EDI&5q loss",
    "EDI5&no 5q loss", "EDI5&5q loss"))
plotSurv(trait$S_10year[set2], comb, name = "", col = c("darkred", "orange",
    "darkblue", "lightblue"))
comb[comb %in% c("Low EDI& no 5q loss", "Low EDI&5q loss")] <- "Low EDI"
plotSurv(trait$S_10year[set2], comb, name = "", col = c("darkred", "orange",
    "black"))
```

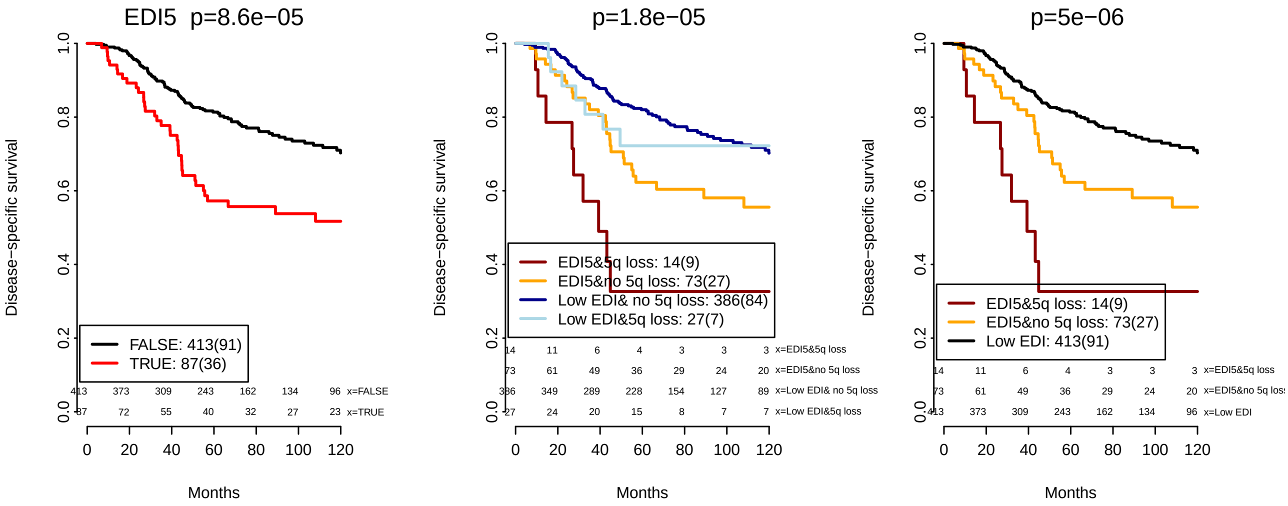


```r
summary(survfit(trait$S_10year[set2 & cn5qloss & EDI5] ~ 1))
```

6.2 Cox regression analysis for EDI5 and 4p14, 5q13

Univariate and multivariate Cox regression analysis for either: 4p14 loss on its own (cn4ploss), 4p14 loss and EDI5 (cn4ploss&EDI5) and with or without node and size in grade 3 tumors.

```
summary(coxph(trait$S_10year[set2, ] ~ cn4ploss[set2]))[c(7, 8, 10)]

## $coefficients
##                       coef exp(coef) se(coef)     z Pr(>|z|)
## cn4ploss[set2]TRUE 0.8666     2.379   0.2931 2.956 0.003112
##
## $conf.int
##                     exp(coef) exp(-coef) lower .95 upper .95
## cn4ploss[set2]TRUE     2.379     0.4204     1.339     4.225
##
## $sctest
##     test       df   pvalue
## 9.300284 1.000000 0.002291

summary(coxph(trait$S_10year[set2, ] ~ cn4ploss[set2] & EDI5[set2]))[c(7, 8,
    10)]

## $coefficients
##                                 coef exp(coef) se(coef)     z Pr(>|z|)
## cn4ploss[set2] & EDI5[set2]TRUE 1.729     5.634   0.3321 5.206 1.93e-07
##
## $conf.int
##                                 exp(coef) exp(-coef) lower .95 upper .95
## cn4ploss[set2] & EDI5[set2]TRUE     5.634     0.1775     2.939      10.8
##
## $sctest
##      test        df    pvalue
## 3.454e+01 1.000e+00 4.175e-09

summary(coxph(trait$S_10year[set2, ] ~ cn4ploss[set2] + trait$node[set2] + trait$size[set2]))[
    8, 10)]

## $coefficients
##                       coef exp(coef) se(coef)     z  Pr(>|z|)
## cn4ploss[set2]TRUE 0.8606     2.365   0.2948 2.919 0.0035069
## trait$node[set2]   0.6456     1.907   0.2056 3.140 0.0016891
## trait$size[set2]   0.6547     1.925   0.1762 3.715 0.0002031
##
## $conf.int
##                     exp(coef) exp(-coef) lower .95 upper .95
## cn4ploss[set2]TRUE     2.365     0.4229     1.327     4.214
## trait$node[set2]       1.907     0.5243     1.275     2.854
## trait$size[set2]       1.925     0.5196     1.363     2.719
##
## $sctest
##      test        df    pvalue
## 3.976e+01 3.000e+00 1.200e-08

summary(coxph(trait$S_10year[set2, ] ~ (cn4ploss[set2] & EDI5[set2]) + trait$node[set2] +
    trait$size[set2]))[c(7, 8, 10)]
```

```
## $coefficients
##                                    coef exp(coef) se(coef)     z  Pr(>|z|)
## cn4ploss[set2] & EDI5[set2]TRUE 1.7057     5.505   0.3327 5.126 2.953e-07
## trait$node[set2]                0.6358     1.889   0.2044 3.111 1.862e-03
## trait$size[set2]                0.6737     1.961   0.1764 3.820 1.337e-04
##
## $conf.int
##                                 exp(coef) exp(-coef) lower .95 upper .95
## cn4ploss[set2] & EDI5[set2]TRUE     5.505     0.1816     2.868    10.569
## trait$node[set2]                    1.889     0.5295     1.265     2.819
## trait$size[set2]                    1.961     0.5098     1.388     2.772
##
## $sctest
##      test        df    pvalue
## 6.412e+01 3.000e+00 7.749e-14
```

Same is performed for 5q13 loss.

```
summary(coxph(trait$S_10year[set2, ] ~ cn5qloss[set2]))[c(7, 8, 10)]

## $coefficients
##                       coef exp(coef) se(coef)     z Pr(>|z|)
## cn5qloss[set2]TRUE 0.6065     1.834   0.2674 2.268  0.02334
##
## $conf.int
##                     exp(coef) exp(-coef) lower .95 upper .95
## cn5qloss[set2]TRUE     1.834     0.5452     1.086     3.098
##
## $sctest
##    test      df  pvalue
## 5.30253 1.00000 0.02129

summary(coxph(trait$S_10year[set2, ] ~ cn5qloss[set2] & EDI5[set2]))[c(7, 8,
    10)]

## $coefficients
##                                   coef exp(coef) se(coef)     z  Pr(>|z|)
## cn5qloss[set2] & EDI5[set2]TRUE 1.326     3.764   0.3465 3.826 0.0001303
##
## $conf.int
##                                 exp(coef) exp(-coef) lower .95 upper .95
## cn5qloss[set2] & EDI5[set2]TRUE     3.764     0.2656     1.909     7.424
##
## $sctest
##      test        df    pvalue
## 1.691e+01 1.000e+00 3.923e-05

summary(coxph(trait$S_10year[set2, ] ~ cn5qloss[set2] + trait$node[set2] + trait$size[set2]))[
    8, 10)]

## $coefficients
##                       coef exp(coef) se(coef)     z  Pr(>|z|)
## cn5qloss[set2]TRUE 0.6434     1.903   0.2684 2.397 0.0165198
## trait$node[set2]   0.6428     1.902   0.2055 3.128 0.0017576
```

```
## trait$size[set2]   0.6703     1.955   0.1759 3.811 0.0001383
##
## $conf.int
##                     exp(coef) exp(-coef) lower .95 upper .95
## cn5qloss[set2]TRUE     1.903     0.5255     1.125     3.220
## trait$node[set2]       1.902     0.5258     1.271     2.845
## trait$size[set2]       1.955     0.5116     1.385     2.759
##
## $sctest
##      test        df    pvalue
## 3.625e+01 3.000e+00 6.630e-08
```

```
summary(coxph(trait$S_10year[set2, ] ~ (cn5qloss[set2] & EDI5[set2]) + trait$node[set2] +
    trait$size[set2]))[c(7, 8, 10)]
```

```
## $coefficients
##                                  coef exp(coef) se(coef)     z  Pr(>|z|)
## cn5qloss[set2] & EDI5[set2]TRUE 1.3259     3.766   0.3468 3.824 1.315e-04
## trait$node[set2]                0.6194     1.858   0.2042 3.033 2.419e-03
## trait$size[set2]                0.6862     1.986   0.1749 3.924 8.692e-05
##
## $conf.int
##                                 exp(coef) exp(-coef) lower .95 upper .95
## cn5qloss[set2] & EDI5[set2]TRUE     3.766     0.2656     1.908     7.430
## trait$node[set2]                    1.858     0.5382     1.245     2.772
## trait$size[set2]                    1.986     0.5035     1.410     2.798
##
## $sctest
##      test        df    pvalue
## 4.714e+01 3.000e+00 3.252e-10
```

7 Comparison with cancer hallmarks

7.1 Prognostic value of tumor heterogeneity measures

We have three measures of cancer hallmarks for comparison: EDI, genomic instability and APOBEC3B expression in low grade (Grade 1&2) and high grade (Grade 3) breast tumors tumors.

```
dat <- data.frame(EDI = EDI, GI = trait$GI, APOBEC3B = trait$APOBEC3B, TP53 = trait$TP53)
set2 <- grepl(3, trait$grade)
par(mfrow = c(4, 3))
plotSurv(trait$S_10year, EDI5, name = "EDI", type = "All")
plotSurv(trait$S_10year[set2], EDI5[set2], name = "EDI", type = "Grade3")
plotSurv(trait$S_10year[!set2], EDI5[!set2], name = "EDI", type = "Grade1&2")
plotSurv(trait$S_10year, group3(dat[, 2]), name = "GI", type = "All")
plotSurv(trait$S_10year[set2], group3(dat[set2, 2]), name = "GI", type = "Grade3")
plotSurv(trait$S_10year[!set2], group3(dat[!set2, 2]), name = "GI", type = "Grade1&2")
plotSurv(trait$S_10year, group3(dat[, 3]), name = "APOBEC3B", type = "All")
plotSurv(trait$S_10year[set2], group3(dat[set2, 3]), name = "APOBEC3B", type = "Grade3")
plotSurv(trait$S_10year[!set2], group3(dat[!set2, 3]), name = "APOBEC3B", type = "Grade1&2")
plotSurv(trait$S_10year, dat[, 4], name = "TP53", type = "All")
plotSurv(trait$S_10year[set2], dat[, 4][set2], name = "TP53", type = "Grade3")
plotSurv(trait$S_10year[!set2], dat[, 4][!set2], name = "TP53", type = "Grade1&2")
```

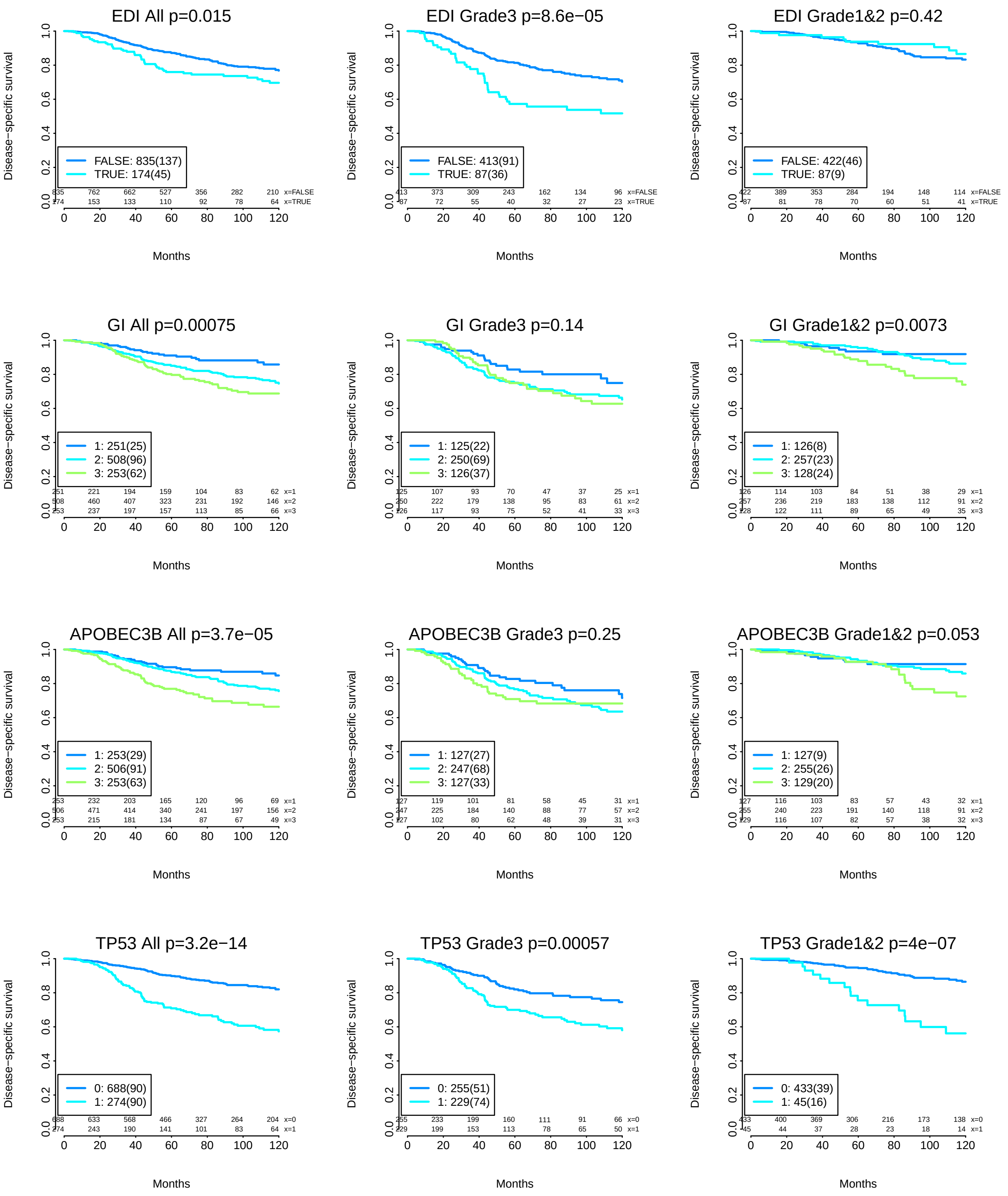

7.2 Correlation with grade node and size

We then used box plots to show their correlations with standard clinical parameters including grade, size and node.

```r
par(mfrow = c(3, 3), mar = c(4, 2, 3, 1))
for (i in 1:3) {
    Boxplot(dat[, i] ~ trait$grade, main = colnames(dat)[i], xlab = "Grade")
    Boxplot(dat[, i] ~ trait$size, main = colnames(dat)[i], xlab = "Size")
    Boxplot(dat[, i] ~ trait$node, main = colnames(dat)[i], xlab = "Node")
```

```
}
```

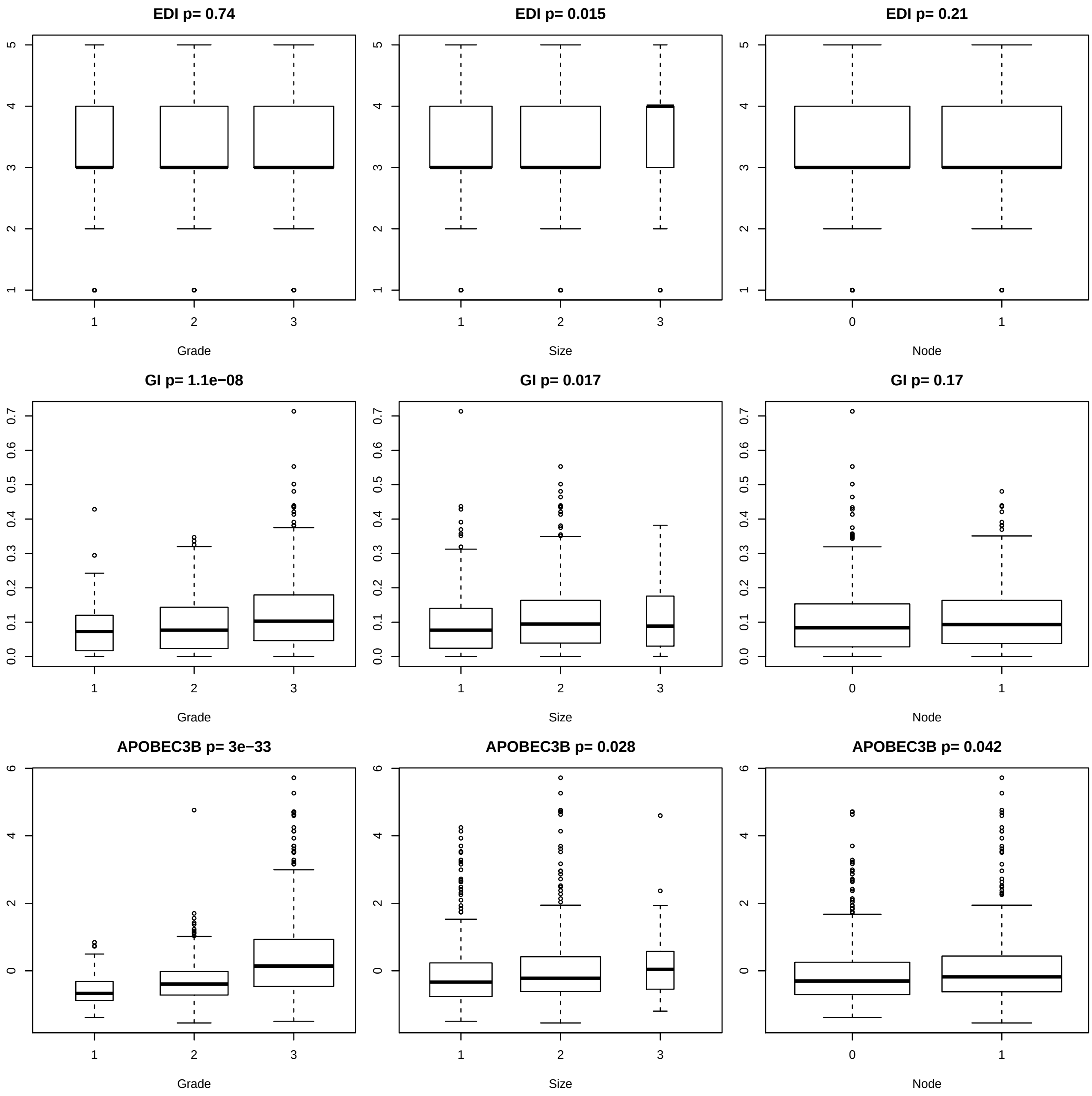

7.3 Correlation among EDI5 and cancer hallmarks

We next explore how the cancer hallmarks are correlated with each other. In high-grade cancer EDI5 is not correlated with GI and APOBEC3B expression. Same was observed in cancer of all grades.

```
set2 <- grepl(3, trait$grade)
par(mfrow = c(2, 2))
Boxplot(trait$GI[set2] ~ EDI5[set2], ylab = "GI", xlab = "EDI5", main = "GI versus EDI5, G3")
Boxplot(trait$APOBEC3B[set2] ~ EDI5[set2], ylab = "APOBEC3B", xlab = "EDI5",
    main = "APOBEC3B versus EDI5, G3")
Boxplot(trait$GI ~ EDI5, ylab = "GI", xlab = "EDI5", main = "GI versus EDI, G1-3")
Boxplot(trait$APOBEC3B ~ EDI5, ylab = "APOBEC3B", xlab = "EDI5", main = "APOBEC3B versus EDI,
```

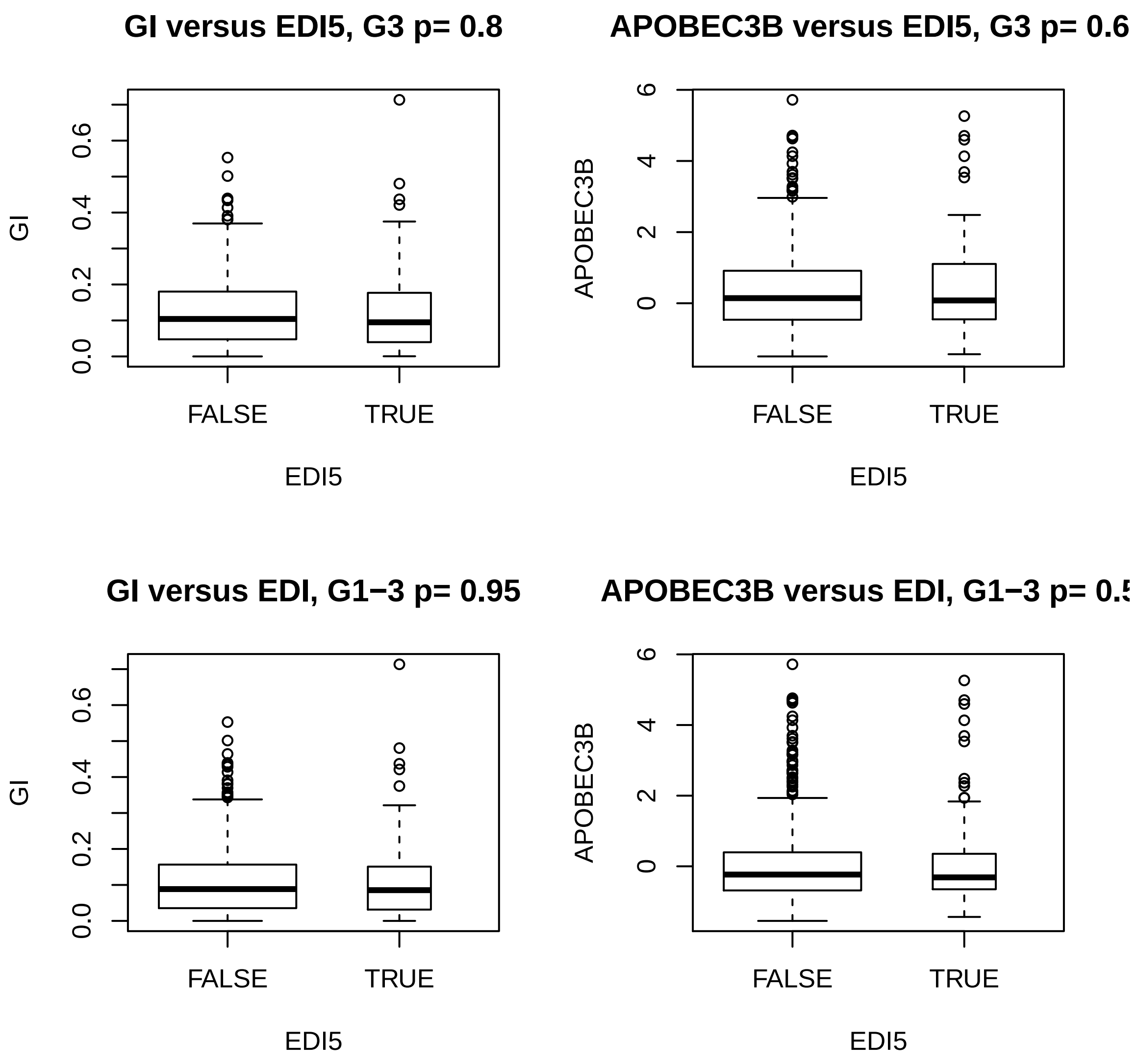

TP53 mutation status, on the other hand, is significantly associated with GI and APOBEC3B expression.

```
par(mfrow = c(2, 2))
Boxplot(trait$GI[set2] ~ trait$TP53[set2], ylab = "GI", xlab = "TP53", main = "GI versus TP53,
Boxplot(trait$APOBEC3B[set2] ~ trait$TP53[set2], ylab = "APOBEC3B", xlab = "TP53",
    main = "APOBEC3B versus TP53, G3")
Boxplot(trait$GI ~ trait$TP53, ylab = "GI", xlab = "TP53", main = "GI versus TP53, G1-3")
Boxplot(trait$APOBEC3B ~ trait$TP53, ylab = "APOBEC3B", xlab = "TP53", main = "APOBEC3B versus
```

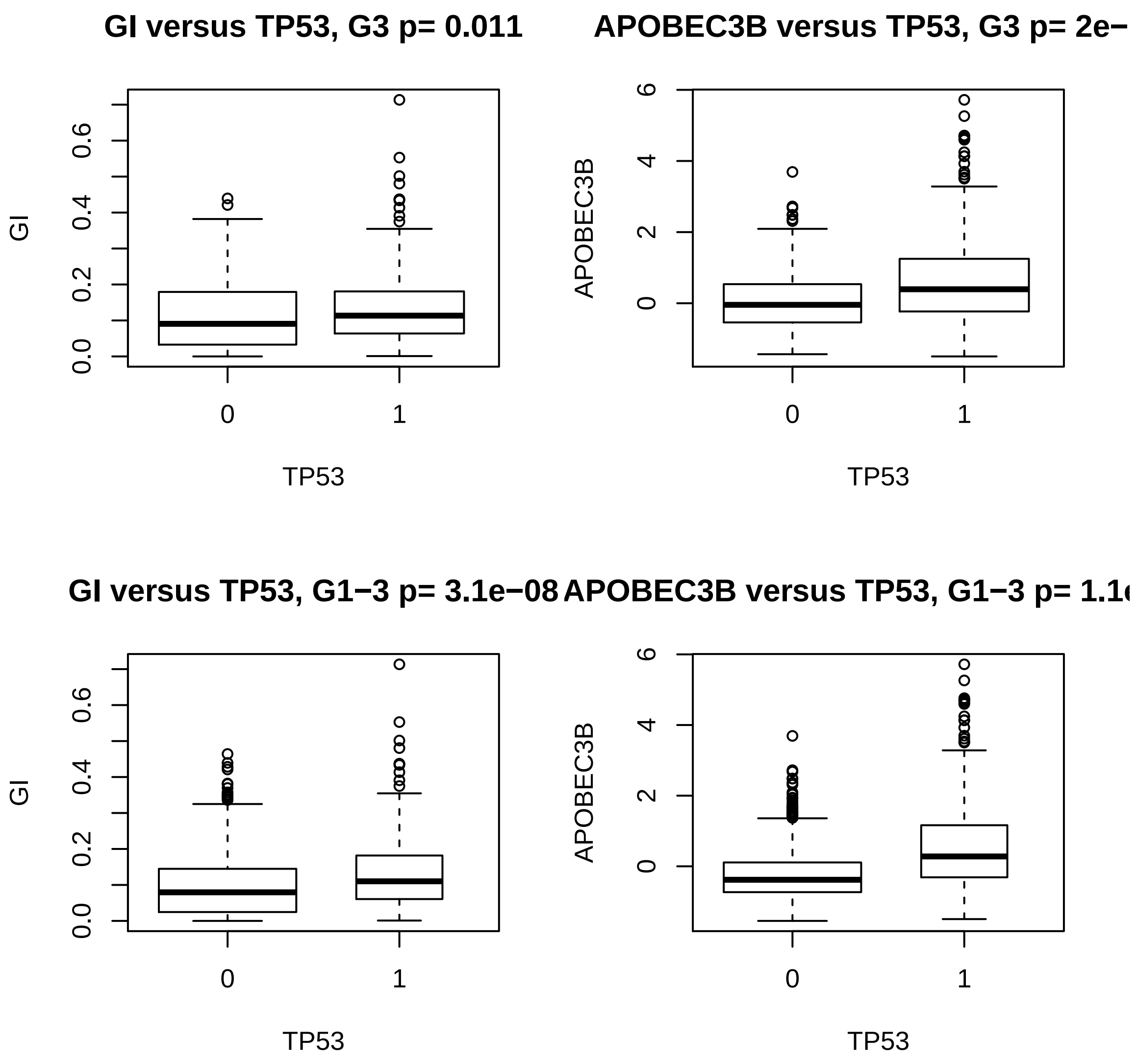

However, EDI5 and TP53 mutation are not associated.

```
x <- EDI5[set2]
z <- trait$TP53[set2] == 0
phyper(sum(x & z, na.rm = T), sum(x, na.rm = T), length(x) - sum(x, na.rm = T),
    sum(z, na.rm = T), lower.tail = F)

## [1] 0.2892

x <- EDI5
z <- trait$TP53 == 0
phyper(sum(x & z, na.rm = T), sum(x, na.rm = T), length(x) - sum(x, na.rm = T),
    sum(z, na.rm = T), lower.tail = F)

## [1] 0.2959
```

Our data showed that the three hallmark measures show significant inter-correlations, but there is no correlation between them and the image-based EDI scores.

7.4 Multivariate analysis of cancer hallmark measures

Multivariate and univariate cox regression model to test cancer and microenvironmental factors in grade 3 cancer.

```
set2 <- grepl(3, trait$grade)
summary(coxph(trait$S_10year ~ EDI5 + trait$TP53 + trait$APOBEC3B + trait$GI,
    subset = set2 & Site[[1]]))[c(8, 10, 14)]

## $conf.int
##                 exp(coef) exp(-coef) lower .95 upper .95
## EDI5TRUE           2.0977     0.4767    1.3168     3.342
## trait$TP53         1.8985     0.5267    1.2015     3.000
## trait$APOBEC3B     0.9614     1.0401    0.8083     1.144
## trait$GI           2.9982     0.3335    0.4920    18.270
##
## $sctest
##      test         df     pvalue
## 1.937e+01 4.000e+00 6.643e-04
##
## $concordance
## concordance.concordant            se.std(c-d)
##                0.62094                0.03291

summary(coxph(trait$S_10year ~ EDI5 + trait$TP53 + trait$APOBEC3B + trait$GI,
    subset = set2 & Site[[2]]))[c(7, 8, 10, 14)]

## $coefficients
##                     coef exp(coef) se(coef)        z Pr(>|z|)
## EDI5TRUE        0.939362    2.5583   0.3801  2.47165  0.01345
## trait$TP53      0.694063    2.0018   0.3375  2.05678  0.03971
## trait$APOBEC3B -0.006972    0.9931   0.1318 -0.05289  0.95782
## trait$GI        1.335386    3.8015   1.2934  1.03242  0.30187
##
## $conf.int
##                 exp(coef) exp(-coef) lower .95 upper .95
## EDI5TRUE           2.5583     0.3909    1.2147     5.388
## trait$TP53         2.0018     0.4995    1.0332     3.879
## trait$APOBEC3B     0.9931     1.0070    0.7670     1.286
## trait$GI           3.8015     0.2631    0.3013    47.966
##
## $sctest
##     test        df    pvalue
## 12.39526  4.00000  0.01464
##
## $concordance
## concordance.concordant            se.std(c-d)
##                0.62171                0.04643


summary(coxph(trait$S_10year ~ trait$TP53, subset = set2 & Site[[1]]))[c(8,
    10, 14)]

## $conf.int
##             exp(coef) exp(-coef) lower .95 upper .95
## trait$TP53      1.805     0.5541     1.158     2.812
##
## $sctest
##     test        df    pvalue
```

```
## 7.010030 1.000000 0.008105
##
## $concordance
## concordance.concordant            se.std(c-d)
##                0.57750                0.02817
```

```r
summary(coxph(trait$S_10year ~ trait$TP53, subset = set2 & Site[[2]]))[c(8,
    10, 14)]
```

```
## $conf.int
##            exp(coef) exp(-coef) lower .95 upper .95
## trait$TP53     1.929     0.5184     1.056     3.523
##
## $sctest
##    test      df  pvalue
## 4.73756 1.00000 0.02951
##
## $concordance
## concordance.concordant            se.std(c-d)
##                0.56882                0.03992
```

```r
summary(coxph(trait$S_10year ~ trait$APOBEC3B, subset = set2 & Site[[1]]))[c(8,
    10, 14)]
```

```
## $conf.int
##                exp(coef) exp(-coef) lower .95 upper .95
## trait$APOBEC3B     1.023     0.9775    0.8596     1.217
##
## $sctest
##    test      df  pvalue
## 0.06553 1.00000 0.79796
##
## $concordance
## concordance.concordant            se.std(c-d)
##                0.54860                0.03269
```

```r
summary(coxph(trait$S_10year ~ trait$APOBEC3B, subset = set2 & Site[[2]]))[c(8,
    10, 14)]
```

```
## $conf.int
##                exp(coef) exp(-coef) lower .95 upper .95
## trait$APOBEC3B     1.108     0.9027     0.877     1.399
##
## $sctest
##   test     df pvalue
## 0.7383 1.0000 0.3902
##
## $concordance
## concordance.concordant            se.std(c-d)
##                0.55115                0.04512
```

```r
summary(coxph(trait$S_10year ~ trait$GI, subset = set2 & Site[[1]]))[c(8, 10,
    14)]
```

```
## $conf.int
##          exp(coef) exp(-coef) lower .95 upper .95
## trait$GI     4.042     0.2474    0.5991     27.27
##
## $sctest
##   test     df pvalue
## 2.0542 1.0000 0.1518
##
## $concordance
## concordance.concordant            se.std(c-d)
##                0.52340                0.03269

summary(coxph(trait$S_10year ~ trait$GI, subset = set2 & Site[[2]]))[c(8, 10,
    14)]

## $conf.int
##          exp(coef) exp(-coef) lower .95 upper .95
## trait$GI     5.975     0.1674    0.4829     73.93
##
## $sctest
##   test     df pvalue
## 1.9478 1.0000 0.1628
##
## $concordance
## concordance.concordant            se.std(c-d)
##                0.54110                0.04512
```

7.5 Association with TP53 mutation

```
table(EDI5, trait$TP53)

##
## EDI5      0   1
##   FALSE 574 228
##   TRUE  124  47

set2 <- grepl(3, trait$grade)
table(EDI5[set2], trait$TP53[set2])

##
##           0   1
##   FALSE 212 190
##   TRUE   47  40
```

Next, KM curves are generated to show the phenotypic similarity between EDI5 and P53 mutated groups.

```
comb <- grepl(1, trait$TP53) * 2 + 1 * (EDI5)
comb <- replace.vector(comb, 0:3, c("Low EDI & P53 WT", "High EDI & P53 WT",
    "Low EDI & P53 MUT", "High EDI & P53 MUT"))

par(mfrow = c(1, 3))
set2 <- grepl(3, trait$grade)
```

```r
plotSurv(trait$S_10year[set2], trait$TP53[set2], col = c("darkred", "black"),
    name = "", type = "P53")

## [1] 0.0005664

plotSurv(trait$S_10year[set2], EDI5[set2], col = c("orange", "darkblue"), name = "",
    type = "EDI")

## [1] 8.562e-05

plotSurv(trait$S_10year[set2], comb[set2], col = c("lightblue", "darkred", "black",
    "darkblue"), name = "", type = "Combined")
```

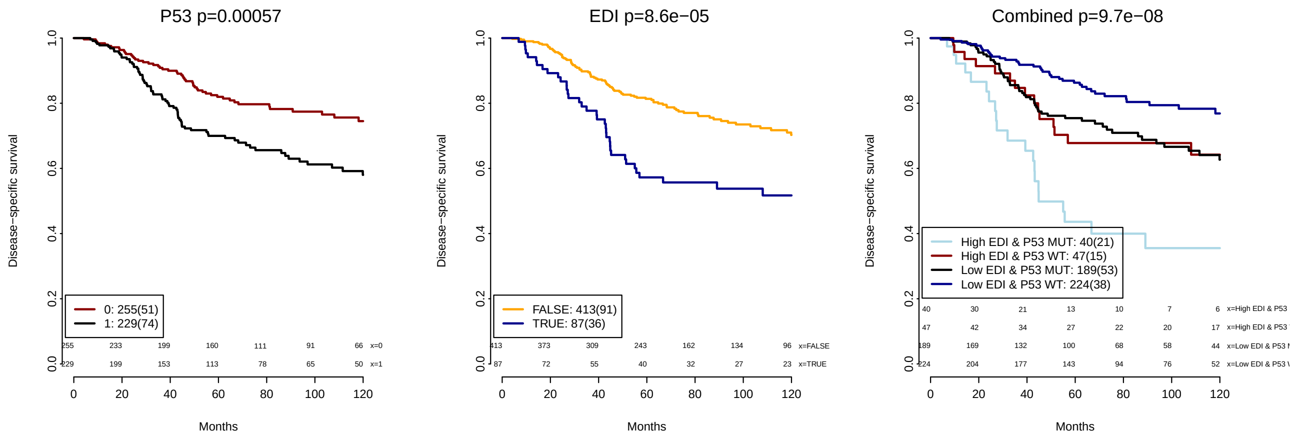


```
## [1] 9.74e-08

summary(survfit(trait$S_10year[set2 & EDI5 & trait$TP53 == 1] ~ 1))

## Call: survfit.formula(formula = trait$S_10year[set2 & EDI5 & trait$TP53 ==
##     1] ~ 1)
##
## 1 observation deleted due to missingness
##   time n.risk n.event survival std.err lower 95% CI upper 95% CI
##   6.83     39       1    0.974  0.0253        0.926        1.000
##   9.60     37       1    0.948  0.0358        0.880        1.000
##  10.60     36       1    0.922  0.0434        0.840        1.000
##  14.40     34       1    0.895  0.0499        0.802        0.998
##  16.73     31       1    0.866  0.0560        0.763        0.983
##  23.20     29       1    0.836  0.0615        0.724        0.966
##  24.23     28       1    0.806  0.0662        0.686        0.947
##  26.77     27       1    0.776  0.0701        0.650        0.927
##  27.20     26       1    0.746  0.0735        0.615        0.905
##  27.47     25       1    0.716  0.0764        0.581        0.883
##  31.93     23       1    0.685  0.0792        0.546        0.859
##  39.30     22       1    0.654  0.0815        0.512        0.835
##  42.57     21       1    0.623  0.0833        0.479        0.810
##  43.10     20       1    0.592  0.0848        0.447        0.784
##  43.20     19       1    0.561  0.0859        0.415        0.757
##  44.77     18       1    0.530  0.0866        0.384        0.730
##  44.83     17       1    0.498  0.0869        0.354        0.701
##  55.00     16       1    0.467  0.0869        0.325        0.673
```

```
##   55.63      15         1     0.436   0.0865         0.296         0.643
##   66.73      12         1     0.400   0.0866         0.262         0.611
##   89.10       9         1     0.355   0.0876         0.219         0.576
```

We create the three-grouping variable that combines the Low EDI & P53 MUT and High EDI & P53 WT groups into High EDI or P53 MUT:

```
comb0 <- grepl(1, trait$TP53) * 2 + 1 * (EDI5)
comb0[comb0 == 2] <- 1
comb <- replace.vector(comb0, 0:3, c("Low EDI & P53 WT", "High EDI or P53 MUT",
    "High EDI or P53 MUT", "High EDI & P53 MUT"))
par(mfrow = c(2, 3))
set2 <- grepl(3, trait$grade) & Site[[1]]
plotSurv(trait$S_10year[set2], trait$TP53[set2], col = c("darkred", "black"),
    name = "", type = "P53 Site1")

## [1] 0.008115

plotSurv(trait$S_10year[set2], EDI5[set2], col = c("orange", "darkblue"), name = "",
    type = "EDI5")

## [1] 0.002592

plotSurv(trait$S_10year[set2], comb[set2], col = c("lightblue", "darkred", "black",
    "darkblue"), name = "", type = "Combined")

## [1] 3.458e-05

summary(coxph(trait$S_10year[set2, ] ~ (comb[set2] == "High EDI & P53 MUT")))

## Call:
## coxph(formula = trait$S_10year[set2, ] ~ (comb[set2] == "High EDI & P53 MUT"))
##
##   n= 245, number of events= 80
##    (6 observations deleted due to missingness)
##
##                                             coef exp(coef) se(coef)    z
## comb[set2] == "High EDI & P53 MUT"TRUE 1.057     2.879    0.281 3.77
##                                        Pr(>|z|)
## comb[set2] == "High EDI & P53 MUT"TRUE  0.00016 ***
## ---
## Signif. codes:  0 '***' 0.001 '**' 0.01 '*' 0.05 '.' 0.1 ' ' 1
##
##                                        exp(coef) exp(-coef) lower .95
## comb[set2] == "High EDI & P53 MUT"TRUE      2.88      0.347      1.66
##                                        upper .95
## comb[set2] == "High EDI & P53 MUT"TRUE      4.99
##
## Concordance= 0.564  (se = 0.016 )
## Rsquare= 0.045   (max possible= 0.968 )
## Likelihood ratio test= 11.4  on 1 df,   p=0.000734
## Wald test            = 14.2  on 1 df,   p=0.000164
## Score (logrank) test = 15.6  on 1 df,   p=7.98e-05
```

```
set2 <- grepl(3, trait$grade) & Site[[2]]
plotSurv(trait$S_10year[set2], trait$TP53[set2], col = c("darkred", "black"),
    name = "", type = "P53 Site2")

## [1] 0.02951

plotSurv(trait$S_10year[set2], EDI5[set2], col = c("orange", "darkblue"), name = "",
    type = "EDI5")

## [1] 0.02544

plotSurv(trait$S_10year[set2], comb[set2], col = c("lightblue", "darkred", "black",
    "darkblue"), name = "", type = "Combined")
```

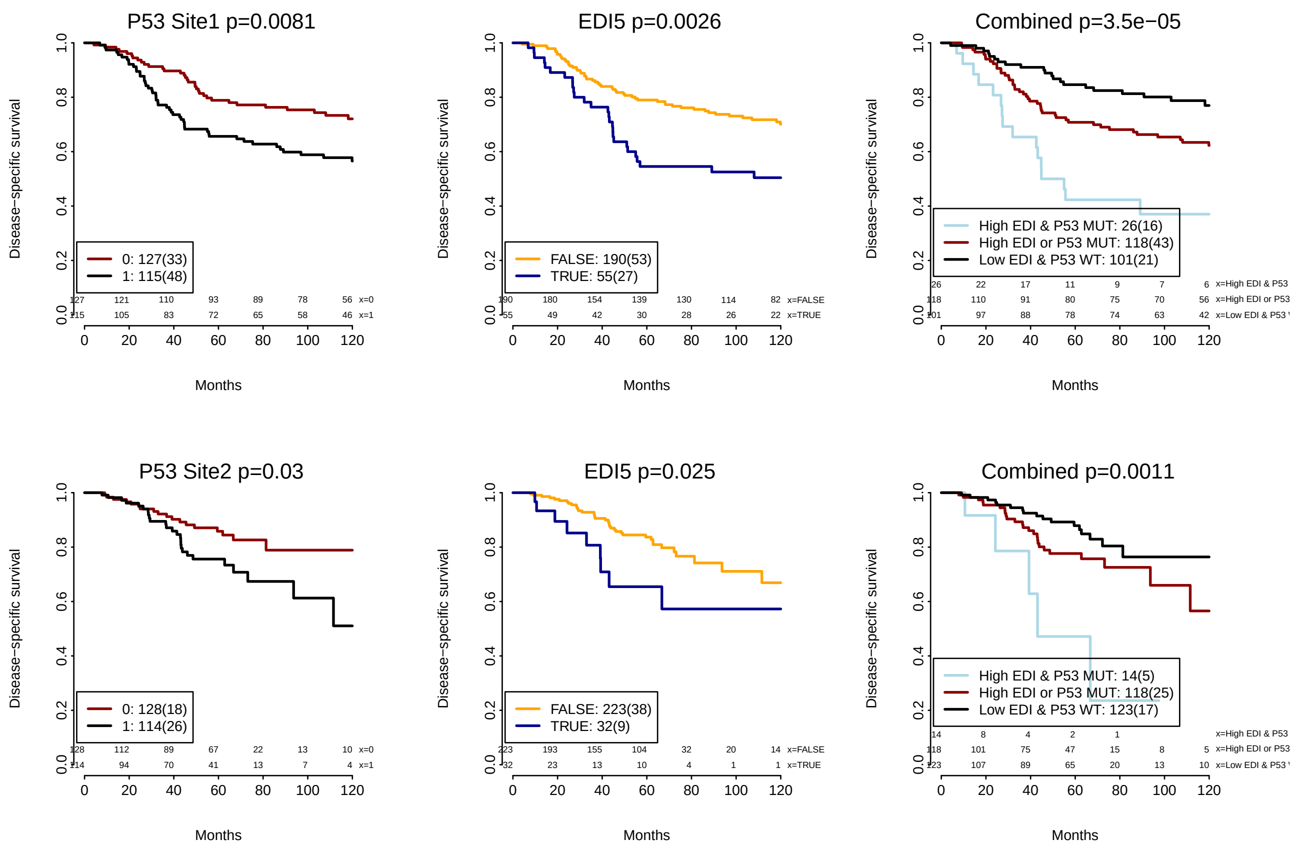


```
## [1] 0.001061

summary(coxph(trait$S_10year[set2, ] ~ (comb[set2] == "High EDI & P53 MUT")))

## Call:
## coxph(formula = trait$S_10year[set2, ] ~ (comb[set2] == "High EDI & P53 MUT"))
##
##   n= 255, number of events= 47
##    (1 observation deleted due to missingness)
##
##                                          coef exp(coef) se(coef)     z
## comb[set2] == "High EDI & P53 MUT"TRUE 1.435     4.201    0.476 3.01
##                                        Pr(>|z|)
## comb[set2] == "High EDI & P53 MUT"TRUE   0.0026 **
```

```
## ---
## Signif. codes:  0 '***' 0.001 '**' 0.01 '*' 0.05 '.' 0.1 ' ' 1
##
##                                         exp(coef) exp(-coef) lower .95
## comb[set2] == "High EDI & P53 MUT"TRUE       4.2      0.238      1.65
##                                         upper .95
## comb[set2] == "High EDI & P53 MUT"TRUE      10.7
##
## Concordance= 0.542  (se = 0.014 )
## Rsquare= 0.025   (max possible= 0.84 )
## Likelihood ratio test= 6.37  on 1 df,   p=0.0116
## Wald test            = 9.09  on 1 df,   p=0.00257
## Score (logrank) test = 10.8  on 1 df,   p=0.00104
```

Multivariate Cox regression analysis is performed on the high-grade tumors for each sample cohort, considering only variables shown to be associated with survival in univariate analysis. Three patient groups are considered here: Low EDI&P53 WT, EDI5 or P53 MUT but not both, EDI5 & P53 MUT.

```
set2 <- grepl(3, trait$grade) & Site[[1]]
summary(coxph(trait$S_10year[set2, ] ~ comb0[set2]))

## Call:
## coxph(formula = trait$S_10year[set2, ] ~ comb0[set2])
##
##   n= 245, number of events= 80
##    (6 observations deleted due to missingness)
##
##               coef exp(coef) se(coef)    z Pr(>|z|)
## comb0[set2] 0.468     1.597    0.106 4.41    1e-05 ***
## ---
## Signif. codes:  0 '***' 0.001 '**' 0.01 '*' 0.05 '.' 0.1 ' ' 1
##
##              exp(coef) exp(-coef) lower .95 upper .95
## comb0[set2]       1.6      0.626       1.3      1.97
##
## Concordance= 0.62  (se = 0.03 )
## Rsquare= 0.066   (max possible= 0.968 )
## Likelihood ratio test= 16.8  on 1 df,   p=4.06e-05
## Wald test            = 19.4  on 1 df,   p=1.04e-05
## Score (logrank) test = 20.3  on 1 df,   p=6.51e-06

summary(coxph(trait$S_10year[set2, ] ~ comb0[set2] + trait$node[set2] + trait$size[set2] +
    trait$ER.Expr[set2]))

## Call:
## coxph(formula = trait$S_10year[set2, ] ~ comb0[set2] + trait$node[set2] +
##     trait$size[set2] + trait$ER.Expr[set2])
##
##   n= 245, number of events= 80
##    (6 observations deleted due to missingness)
##
##                         coef exp(coef) se(coef)     z Pr(>|z|)
## comb0[set2]            0.382     1.465    0.115  3.32   0.0009 ***
```

```
## trait$node[set2]      0.402     1.495    0.254  1.58   0.1135
## trait$size[set2]      0.679     1.973    0.223  3.05   0.0023 **
## trait$ER.Expr[set2]+ -0.285     0.752    0.245 -1.16   0.2454
## ---
## Signif. codes:  0 '***' 0.001 '**' 0.01 '*' 0.05 '.' 0.1 ' ' 1
##
##                      exp(coef) exp(-coef) lower .95 upper .95
## comb0[set2]              1.465      0.682     1.169      1.84
## trait$node[set2]         1.495      0.669     0.909      2.46
## trait$size[set2]         1.973      0.507     1.275      3.05
## trait$ER.Expr[set2]+     0.752      1.330     0.465      1.22
##
## Concordance= 0.692  (se = 0.033 )
## Rsquare= 0.125   (max possible= 0.968 )
## Likelihood ratio test= 32.8  on 4 df,   p=1.33e-06
## Wald test            = 33.6  on 4 df,   p=8.99e-07
## Score (logrank) test = 35.6  on 4 df,   p=3.56e-07
```

```
set2 <- grepl(3, trait$grade) & Site[[2]]
summary(coxph(trait$S_10year[set2, ] ~ comb0[set2]))
```

```
## Call:
## coxph(formula = trait$S_10year[set2, ] ~ comb0[set2])
##
##   n= 255, number of events= 47
##    (1 observation deleted due to missingness)
##
##              coef exp(coef) se(coef)   z Pr(>|z|)
## comb0[set2] 0.575     1.777    0.169 3.4  0.00067 ***
## ---
## Signif. codes:  0 '***' 0.001 '**' 0.01 '*' 0.05 '.' 0.1 ' ' 1
##
##             exp(coef) exp(-coef) lower .95 upper .95
## comb0[set2]      1.78      0.563      1.28      2.48
##
## Concordance= 0.605  (se = 0.04 )
## Rsquare= 0.037   (max possible= 0.84 )
## Likelihood ratio test= 9.55  on 1 df,   p=0.002
## Wald test            = 11.6  on 1 df,   p=0.000669
## Score (logrank) test = 11.8  on 1 df,   p=0.000607
```

```
summary(coxph(trait$S_10year[set2, ] ~ comb0[set2] + trait$node[set2] + trait$size[set2] +
    trait$ER.Expr[set2]))
```

```
## Call:
## coxph(formula = trait$S_10year[set2, ] ~ comb0[set2] + trait$node[set2] +
##     trait$size[set2] + trait$ER.Expr[set2])
##
##   n= 252, number of events= 46
##    (4 observations deleted due to missingness)
##
##                         coef exp(coef) se(coef)     z Pr(>|z|)
## comb0[set2]            0.579     1.784    0.178  3.25   0.0011 **
## trait$node[set2]       0.941     2.564    0.376  2.51   0.0122 *
```

```
## trait$size[set2]      0.936     2.549    0.286  3.28   0.0011 **
## trait$ER.Expr[set2]+ -0.573     0.564    0.319 -1.79   0.0727 .
## ---
## Signif. codes:  0 '***' 0.001 '**' 0.01 '*' 0.05 '.' 0.1 ' ' 1
##
##                        exp(coef) exp(-coef) lower .95 upper .95
## comb0[set2]                1.784      0.560     1.259      2.53
## trait$node[set2]           2.564      0.390     1.228      5.35
## trait$size[set2]           2.549      0.392     1.456      4.46
## trait$ER.Expr[set2]+       0.564      1.773     0.302      1.05
##
## Concordance= 0.722  (se = 0.045 )
## Rsquare= 0.122   (max possible= 0.837 )
## Likelihood ratio test= 32.8  on 4 df,   p=1.34e-06
## Wald test            = 33  on 4 df,   p=1.2e-06
## Score (logrank) test = 33.9  on 4 df,   p=7.68e-07
```

Combining TP53 and EDI5 over treatment options:

```
par(mfrow = c(2, 3))
set2 <- grepl(3, trait$grade) & trait$ct
plotSurv(trait$S_10year[set2], EDI5[set2], type = "CT-treated", name = "EDI5")

## [1] 0.007368

plotSurv(trait$S_10year[set2], trait$TP53[set2], type = "CT-treated", name = "TP53")

## [1] 0.9354

plotSurv(trait$S_10year[set2], comb[set2], type = "CT-treated", name = "Combined")

## [1] 0.2023

set2 <- grepl(3, trait$grade) & !trait$ct
plotSurv(trait$S_10year[set2], EDI5[set2], type = "CT-null", name = "EDI5")

## [1] 0.002825

plotSurv(trait$S_10year[set2], trait$TP53[set2], type = "CT-null", name = "TP53")

## [1] 6.878e-06

plotSurv(trait$S_10year[set2], comb[set2], type = "CT-null", name = "Combined")
```

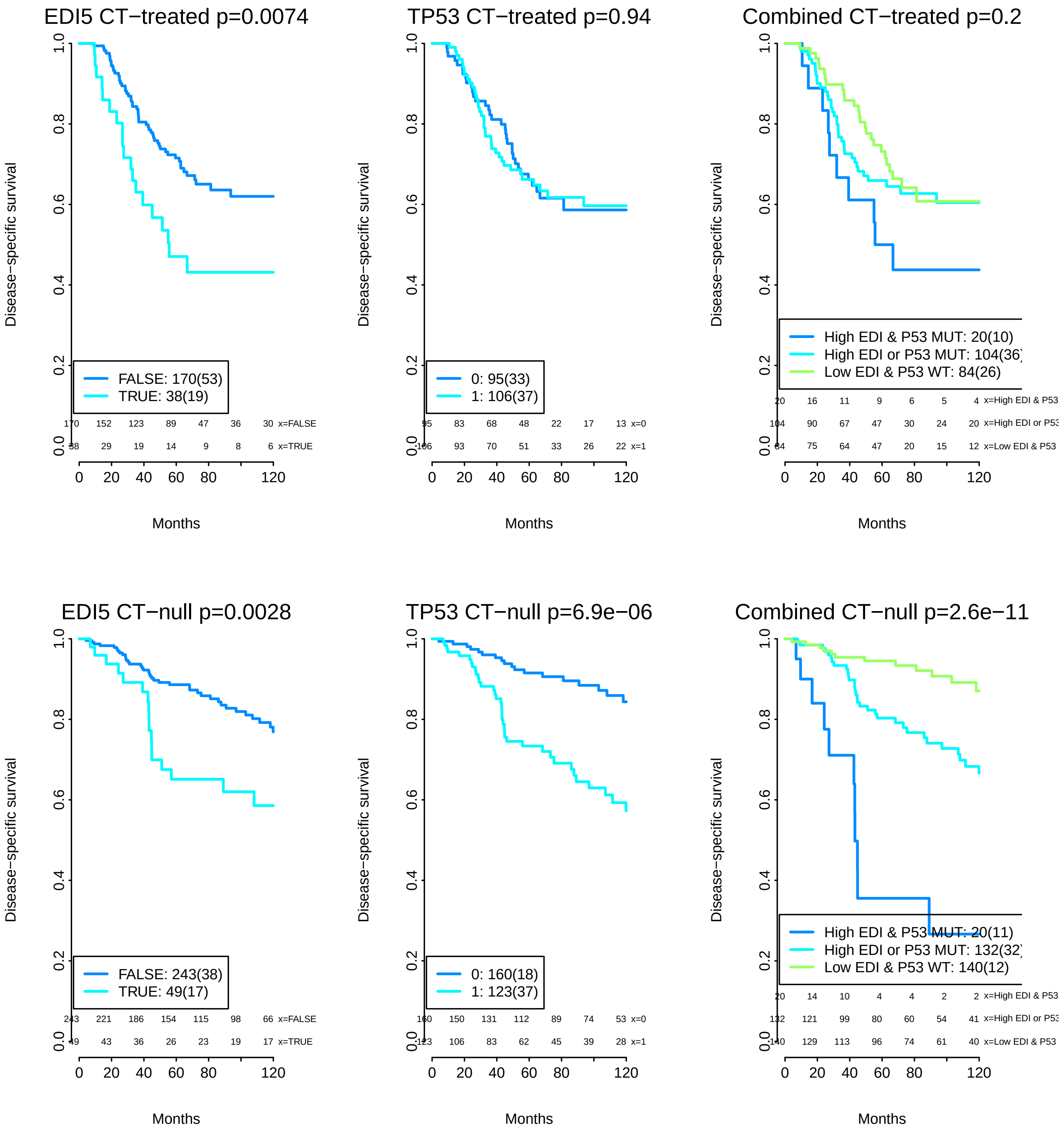


```
## [1] 2.644e-11

summary(coxph(trait$S_10year[set2, ] ~ comb[set2] + trait$node[set2] + trait$size[set2] +
    trait$ER.Expr[set2]))[c(7, 8, 10, 14)]

## $coefficients
##                                  coef exp(coef) se(coef)      z  Pr(>|z|)
## comb[set2]High EDI or P53 MUT -1.5158   0.21963   0.3633 -4.172 3.014e-05
## comb[set2]Low EDI & P53 WT    -2.5873   0.07522   0.4338 -5.964 2.454e-09
## trait$node[set2]               0.8249   2.28167   0.3014  2.737 6.200e-03
## trait$size[set2]               0.7204   2.05529   0.2576  2.796 5.171e-03
## trait$ER.Expr[set2]+          -0.4972   0.60825   0.3330 -1.493 1.355e-01
##
```

```
## $conf.int
##                              exp(coef) exp(-coef) lower .95 upper .95
## comb[set2]High EDI or P53 MUT   0.21963     4.5530   0.10776    0.4476
## comb[set2]Low EDI & P53 WT      0.07522    13.2938   0.03215    0.1760
## trait$node[set2]                2.28167     0.4383   1.26389    4.1190
## trait$size[set2]                2.05529     0.4865   1.24041    3.4055
## trait$ER.Expr[set2]+            0.60825     1.6441   0.31667    1.1683
##
## $sctest
##      test        df    pvalue
## 6.870e+01 5.000e+00 1.911e-13
##
## $concordance
## concordance.concordant            se.std(c-d)
##                0.76075                0.04083
```

8 Robustness of the Cox model

We estimate the univariate hazard ratio for EDI5 on high grade breast cancer DSS using a Cox proportional hazards model

```
set2 <- grepl(3, trait$grade)
summary(coxph(trait$S_10year[set2] ~ EDI5[set2]))

## Call:
## coxph(formula = trait$S_10year[set2] ~ EDI5[set2])
##
##   n= 500, number of events= 127
##    (7 observations deleted due to missingness)
##
##                   coef exp(coef) se(coef)    z Pr(>|z|)
## EDI5[set2]TRUE 0.756     2.129    0.197 3.84  0.00013 ***
## ---
## Signif. codes:  0 '***' 0.001 '**' 0.01 '*' 0.05 '.' 0.1 ' ' 1
##
##                exp(coef) exp(-coef) lower .95 upper .95
## EDI5[set2]TRUE      2.13       0.47      1.45      3.13
##
## Concordance= 0.572  (se = 0.017 )
## Rsquare= 0.026   (max possible= 0.948 )
## Likelihood ratio test= 13  on 1 df,   p=0.000308
## Wald test            = 14.7  on 1 df,   p=0.000125
## Score (logrank) test = 15.4  on 1 df,   p=8.57e-05
```

The hazard ratio for EDI5 is 2.12 and the lower 95% confidence interval is lower 95% 1.45, higher lower 95% 3.13). We confirmed the robustness of our results using bootstrap analysis. Data were sampled with replacement 1,000 times and the log-rank survival analysis was performed for each resampling.

```
set.seed(45)  ## Random seed set to make the result reproducible
resB2 <- replicate(1000, 1 - pchisq(survdiff(trait$S_10year[set2] ~ EDI5[set2],
    subset = sample(1:sum(set2), replace = TRUE))$chisq, 1))
mean(resB2 < 0.05)
```

```
## [1] 0.953
```

This means: In 95% our univariate analysis results stay significant in the perturbed data.

```
set.seed(45)
resB2 <- replicate(1000, summary(coxph(trait$S_10year[set2] ~ EDI5[set2] + trait$node[set2] +
    trait$size[set2], subset = sample(1:sum(set2), replace = TRUE)))$coef[1,
    5])
mean(resB2 < 0.05)

## [1] 0.909
```

This means in 91% our results of multivariate analysis stay significant. This demonstrated the stability of EDI as a cancer heterogeneity marker and prognostic factor in high-grade breast tumors.

9 Session Info

```
sessionInfo()

## R version 2.15.1 (2012-06-22)
## Platform: x86_64-apple-darwin9.8.0/x86_64 (64-bit)
##
## locale:
## [1] en_GB.UTF-8/en_GB.UTF-8/en_GB.UTF-8/C/en_GB.UTF-8/en_GB.UTF-8
##
## attached base packages:
## [1] grid      splines   stats     graphics  grDevices utils     datasets
## [8] methods   base
##
## other attached packages:
##  [1] ggplot2_0.9.2.1 vegan_2.0-5     permute_0.7-0   knitr_1.1
##  [5] rms_4.1-3       SparseM_1.03    Hmisc_3.14-3    Formula_1.1-1
##  [9] lattice_0.20-10 survival_2.37-7
##
## loaded via a namespace (and not attached):
##  [1] cluster_1.14.2      colorspace_1.2-0    dichromat_1.2-4
##  [4] digest_0.5.2        evaluate_0.4.3      formatR_0.7
##  [7] gtable_0.1.1        labeling_0.1        latticeExtra_0.6-24
## [10] MASS_7.3-22         memoise_0.1         munsell_0.4
## [13] plyr_1.7.1          proto_0.3-9.2       RColorBrewer_1.0-5
## [16] reshape2_1.2.1      scales_0.2.2        stringr_0.6.1
## [19] tools_2.15.1
```
