# Supplementary material for: Microenvironmental Heterogeneity Parallels Breast Cancer Progression: A Histology–Genomic Integration Analysis
Source: PLoS Med. 2016 Feb 16;13(2):e1001961. doi: 10.1371/journal.pmed.1001961 (PMC4755617; doi:10.1371/journal.pmed.1001961)
Supplement: S3 Text — (DOCX) [file pmed.1001961.s018.docx]

**STARD Checklist**

1. Identification as a study of diagnostic accuracy using at least one measure of accuracy (such as sensitivity, specificity, predictive values, or AUC)

**Yes- association with disease-specific survival as described in the abstract**

1. Structured summary of study design, methods, results, and conclusions

**Yes as per journal guidelines**

1. Scientific and clinical background, including the intended use and clinical role of the index test

**Yes- page 5 in the introduction “to define the clinical implication of microenvironmental heterogeneity”**

1. Study objectives and hypotheses

**Yes- Page 5 “Our aims were i) to develop a computational system for quantifying microenvironmental heterogeneity based on tumor morphology in routine histological sections; ii) to define the clinical implication of microenvironmental heterogeneity and iii) to integrate this histology-based index with RNA gene expression and DNA copy number profiling data to identify molecular changes associated with microenvironmental heterogeneity”.**

1. Whether data collection was planned before the index test and reference standard were performed (prospective study) or after (retrospective study)

**Retrospective study- Page 5: “and an independent validation set of 516 samples (hospital 3) for retrospective analysis (Fig 1A, S1 Table)”.**

1. Eligibility criteria

**Availability of H&E images. Page 5 Methods- Clinical samples and CONSORT diagram Figure 1**

1. On what basis potentially eligible participants were identified

**As above**

1. Where and when potentially eligible participants were identified (setting, location and dates)

**From a retrospective analysis of the METABRIC study. See Methods as above on page 5**

1. Whether participants formed a consecutive, random or convenience series

**Consecutive See Page 5: “1,992 untreated primary breast tumors from consecutive series from five contributing hospitals”**

1. a and b. Index test, in sufficient detail to allow replication

**Yes- see Sweave. EDI holds independent prognostic value over the reference Grade 3**

1. Rationale for choosing the reference standard (if alternatives exist)

**N/A**

1. a. Definition of and rationale for test positivity cut-offs or result categories of the index test, distinguishing pre-specified from exploratory

**Yes- see Methods and S1 Text**

b. Definition of and rationale for test positivity cut-offs or result categories of the reference standard, distinguishing pre-specified from exploratory

**Grade was predefined as per METABRIC**

1. a Whether clinical information and reference standard results were available to the performers/readers of the index test

**Yes given the retrospective nature of the study**

b Whether clinical information and index test results were available to the assessors of the reference standard

**No given the retrospective nature of the study**

1. Methods for estimating or comparing measures of diagnostic accuracy

**Univariate and Multivariate analysis to compare test index and other measures were performed (Page 11-16 Results)**

1. How indeterminate index test or reference standard results were handled
   **N/A**
2. How missing data on the index test and reference standard were handled
   **Missing data were specified (Page 10 Table 1)**
3. Any analyses of variability in diagnostic accuracy, distinguishing pre-specified from exploratory

**All exploratory**

1. Intended sample size and how it was determined

**This is retrospective analysis and sample size was determined based on sample availability**

1. Flow of participants, using a diagram

**CONSORT diagram Figure 1A**

1. Baseline demographic and clinical characteristics of participants

**Table 1**

1. a Distribution of severity of disease in those with the target condition

**Table 1 and Figure 2**

b. Distribution of alternative diagnoses in those without the target condition

**Table 1 and Figure 2**

1. Time interval and any clinical interventions between index test and reference standard

**N/A**

1. Cross tabulation of the index test results (or their distribution) by the results of the reference standard

**Table 1 Page 10**

1. Estimates of diagnostic accuracy and their precision (such as 95% confidence intervals)

**95% confidence intervals are given in Table 2**

1. Any adverse events from performing the index test or the reference standard

**None as samples used were routinely collected, specified on page 5 in study aims**

1. Study limitations, including sources of potential bias, statistical uncertainty, and generalizability

**Page 20-21**

1. Implications for practice, including the intended use and clinical role of the index test

**Page 21-22**

1. Registration number and name of registry

**N/A**

1. Where the full study protocol can be accessed

**N/A**

1. Sources of funding and other support; role of funders

**Page 24-25**
